# Risk Factors Affecting Development and Persistence of Preschool Wheezing: Consensus Document of the Emilia-Romagna Asthma (ERA) Study Group

**DOI:** 10.3390/jcm11216558

**Published:** 2022-11-04

**Authors:** Roberto Grandinetti, Valentina Fainardi, Carlo Caffarelli, Gaia Capoferri, Angela Lazzara, Marco Tornesello, Aniello Meoli, Barbara Maria Bergamini, Luca Bertelli, Loretta Biserna, Paolo Bottau, Elena Corinaldesi, Nicoletta De Paulis, Arianna Dondi, Battista Guidi, Francesca Lombardi, Maria Sole Magistrali, Elisabetta Marastoni, Silvia Pastorelli, Alessandra Piccorossi, Maurizio Poloni, Sylvie Tagliati, Francesca Vaienti, Giuseppe Gregori, Roberto Sacchetti, Sandra Mari, Manuela Musetti, Francesco Antodaro, Andrea Bergomi, Lamberto Reggiani, Fabio Caramelli, Alessandro De Fanti, Federico Marchetti, Giampaolo Ricci, Susanna Esposito, on behalf of the Emilia-Romagna Asthma (ERA) Study Group

**Affiliations:** 1 Pediatric Clinic, Department of Medicine and Surgery, University of Parma, 43126 Parma, Italy; 2 Paediatric Unit, Department of Medical and Surgical Sciences of Mothers, Children and Adults, University of Modena and Reggio Emilia, 41125 Modena, Italy; 3 Pediatric Clinic, Scientific Institute for Research and Healthcare (IRCCS) Azienda Ospedaliero-Universitaria di Bologna, 40138 Bologna, Italy; 4 Paediatrics and Neonatology Unit, Ravenna Hospital, AUSL Romagna, 48121 Ravenna, Italy; 5 Paediatrics Unit, Imola Hospital, 40026 Imola, Italy; 6 Paediatric Unit, Carpi Hospital, 41012 Carpi, Italy; 7 Paediatrics and Neonatology Unit, Guglielmo da Saliceto Hospital, 29121 Piacenza, Italy; 8 Hospital and Territorial Paediatrics Unit, Pavullo, 41026 Pavullo Nel Frignano, Italy; 9 Paediatrics Unit, Maggiore Hospital, 40133 Bologna, Italy; 10 Paediatrics Unit, Santa Maria Nuova Hospital, AUSL-IRCCS of Reggio Emilia, 42123 Reggio Emilia, Italy; 11 Paediatrics Unit, Sassuolo Hospital, 41049 Sassuolo, Italy; 12 Paediatrics and Paediatric Intensive Care Unit, Cesena Hospital, AUSL Romagna, 47521 Cesena, Italy; 13 Paediatrics Unit, Rimini Hospital, AUSL Romagna, 47921 Rimini, Italy; 14 Paediatric Clinic, Ferrara Hospital, 44124 Ferrara, Italy; 15 Paediatrics Unit, G.B. Morgagni—L. Pierantoni Hospital, AUSL Romagna, 47121 Forlì, Italy; 16 Primary Care Pediatricians, AUSL Piacenza, 29121 Piacenza, Italy; 17 Primary Care Pediatricians, AUSL Parma, 43126 Parma, Italy; 18 Primary Care Pediatricians, AUSL Modena, 41125 Modena, Italy; 19 Primary Care Pediatricians, AUSL Imola, 40026 Imola, Italy; 20 Pediatric Intensive Care Unit, IRCCS Azienda Ospedaliero-Universitaria di Bologna, 40138 Bologna, Italy

**Keywords:** allergen sensitization, episodic viral wheezing, multiple trigger wheezing, paediatric pulmonology, wheezing

## Abstract

Wheezing at preschool age (i.e., before the age of six) is common, occurring in about 30% of children before the age of three. In terms of health care burden, preschool children with wheeze show double the rate of access to the emergency department and five times the rate of hospital admissions compared with school-age asthmatics. The consensus document aims to analyse the underlying mechanisms involved in the pathogenesis of preschool wheezing and define the risk factors (i.e., allergy, atopy, infection, bronchiolitis, genetics, indoor and outdoor pollution, tobacco smoke exposure, obesity, prematurity) and the protective factors (i.e., probiotics, breastfeeding, vitamin D, influenza vaccination, non-specific immunomodulators) associated with the development of the disease in the young child. A multidisciplinary panel of experts from the Emilia-Romagna Region, Italy, addressed twelve key questions regarding managing preschool wheezing. Clinical questions have been formulated by the expert panel using the PICO format (Patients, Intervention, Comparison, Outcomes). Systematic reviews have been conducted on PubMed to answer these specific questions and formulate recommendations. The GRADE approach has been used for each selected paper to assess the quality of the evidence and the degree of recommendations. Based on a panel of experts and extensive updated literature, this consensus document provides insight into the pathogenesis, risk and protective factors associated with the development and persistence of preschool wheezing. Undoubtedly, more research is needed to improve our understanding of the disease and confirm the associations between certain factors and the risk of wheezing in early life. In addition, preventive strategies must be promoted to avoid children’s exposure to risk factors that may permanently affect respiratory health.

## 1. Introduction

Wheezing at preschool age (i.e., before age six) is common, occurring in about 30% of children before age three [1]. In terms of health care burden, preschool children with wheeze show a doubled rate of emergency department access and five times the rate of hospital admissions compared with school-age asthmatics [2]. An audit performed in the UK among children with acute wheezing/asthma admitted to the hospital showed that wheezing only with colds was common in younger children peaking at around three years, whereas children between 12 and 24 months of age accounted for a quarter of admissions [3]. 

Preschool wheezing can be described as a multifactorial disease influenced by various genetic and environmental factors. At present, the understanding of the pathophysiology and risk factors that contribute to the onset and persistence of wheezing in preschool children is limited. Increasing evidence shows that it is a combination of different factors that contribute to the development of wheezing. Early viral infections [4], bacterial colonisation [5] and allergen sensitisation [6] are among the most important in causing wheeze and the subsequent development of asthma. These early life exposures, coupled with genetically determined susceptibility, can affect the immune system in the early stages of life and have a major impact on the natural history of the disease. In particular, early and multiple sensitisation predicts a severe asthma trajectory [7]. Identifying risk factors may allow the identification of measures and interventions to prevent the development of preschool wheezing and, eventually, its evolution into childhood asthma. 

This consensus document aims to analyse the underlying mechanisms involved in the pathogenesis of preschool wheezing and define the risk factors (i.e., allergy, atopy, infection, bronchiolitis, genetics, indoor and outdoor pollution, tobacco smoke exposure, obesity, prematurity) and the protective factors (i.e., breastfeeding, vitamin D, probiotics) associated with the development of the disease in the young child. 

## 2. Materials and Methods

We set up a multidisciplinary panel of experts that included all the Heads of the Paediatric Units of Emilia-Romagna Region, Italy, the Heads of the outpatient clinics for pulmonology and allergology, a sample of primary care paediatricians (identified in each province based on the number of the paediatric population according to ISTAT 2018 data) and a patients’ association (Respiro Libero, Parma, Italy). This study group (named Emilia-Romagna Asthma Study Group and described in detail in a previous publication on the management of children with acute asthma attacks [8] included members with previous experience in the development of documents and recommendations with the Grading of Recommendations Assessment, Development, and Evaluation [9,10].

In order to assess risk factors and protective factors influencing the development of preschool wheezing, clinical questions have been formulated by the expert panel using the PICO format (Patients, Intervention, Comparison, Outcomes). Systematic reviews have been conducted on PubMed from January 2008 to December 2021 using different search strategies focused on wheezing pathogenesis (in particular atopy and respiratory infections), risk factors associated with preschool wheeze onset or persistence and protective factors that may reduce the risk of developing the disease. Prospective or retrospective cohort and case-control studies were included. Included studies investigated patients <6 years of age (preschool age). Letters, comments, editorials and case reports were excluded. Only full manuscripts published in the English language were included. Search strategies and extended evidence tables are available in Supplementary Material S1. 

Clinical questions were divided into 3 sections:

(1) Pathogenesis of preschool wheezing

Question 1. What is the role of infection in the pathogenesis of preschool wheezing?

Question 2. What is the role of atopy in the pathogenesis of preschool wheezing?

(2) Risk factors for wheeze development

Question 3. Does the presence of risk factors such as allergy/atopy influence the onset and the evolution of preschool wheezing?

Question 4. Does the presence of risk factors such as previous respiratory tract infection/bronchiolitis influence the onset and evolution of preschool wheezing?

Question 5. Does pollution influence the onset and evolution of preschool wheezing?

Question 6. Does genetics influence the onset and the evolution of preschool wheezing?

Question 7. Does obesity influence the onset and the evolution of preschool wheezing?

Question 8. Do prematurity and other perinatal factors influence the onset and the evolution of preschool wheezing?

Question 9. Does smoke exposure influence the onset and the evolution of preschool wheezing?

Question 10. Is immunodeficiency a risk factor for the onset and the evolution of preschool wheezing?

(3) Protective factors for wheeze development:

Question 11. Are probiotics protective for preschool wheezing development?

Question 12. Is vitamin D supplementation protective for preschool wheezing development?

Question 13. Is breastfeeding protective for preschool wheezing development?

Question 14. Is influenza vaccination protective for preschool wheezing development?

Question 15. Are non-specific immunomodulators protective for preschool wheezing development?

The GRADE approach has been used for each selected paper to assess the confidence in the evidence (quality) and the degree of recommendations [11]. Recommendations are graded as strong or weak after considering the quality of the evidence, the balance of desirable and undesirable consequences of compared management options, the assumptions about the relative importance of outcomes, the implications for resource use, and the acceptability and feasibility of implementation [12]. The panel then decided on the strength of the recommendations. A dedicated voting process (collection of voting forms through individual email messages) was developed for the present guidelines, and an online meeting with the participation of the full voting panel was organized. More specifically, voting panel members were provided with the results of the various literature searches, the evidence summaries, the proposed recommendations, and the related GRADE tables. Each voting member was then allowed to individually vote in favour or against each recommendation, propose possible modifications, and judge each recommendation as strong or weak according to GRADE rules. For recommendations with an agreement of <75%, further voting rounds were conducted after implementing dedicated amendments based on the provided comments. After reaching an agreement of ≥75% for all recommendations, all the authors reviewed and approved the final manuscript and Supplementary Material S1.

## 3. Results

### 3.1. Pathogenesis of Preschool Wheezing

#### 3.1.1. PICO Question 1. What Is the Role of Infection in the Pathogenesis of Preschool Wheezing?

##### Executive Summary

In young children, viral infections are the most important triggers of wheezing, particularly for the phenotype called episodic viral wheezing (EVW). These viral infections are mostly due to human rhinovirus (HRV), respiratory syncytial virus (RSV), human metapneumovirus (HMPV) and influenza virus (IV) [13,14,15,16]. If respiratory tract infections (RTIs) are the main reason for wheeze development is unclear. Some studies suggested that certain children are more prone to be severely infected because of dysregulation of the innate immune response, such as low antiviral interferon-gamma (IFN-y) production in response to viral infection or structural airway pathologies [17]. Interestingly, Chawes et al. showed that bronchial hyperresponsiveness in at-risk neonates precedes severe acute bronchiolitis in response to respiratory viral infections [18].

HRV is the most commonly identified virus involved in wheezing exacerbations in preschool and school-aged children [19]. HRV is usually predominant in patients with no history of wheezing [14]. HRV infection at one year of age has been proven to be the strongest predictor for wheezing episodes at three years of age [14,20,21,22] and recurrent wheezing development [13,15,16,23,24,25]. HRV infections may increase airway sensitisation and inflammation by different mechanisms: altering the epithelial barrier [26], inducing the release of epithelial cytokines (i.e., interleukin [IL]-25 and IL-33) with T-helper (Th2) inflammation and production of IL-4, IL-5 and IL-13 [27], stimulating the production of granulocyte-macrophage colony-stimulating factor (GM-CSF), IL-6, IL-8, IL-1α, IL-1β and eventually contributing to airway remodelling by stimulating angiogenesis and differentiation of myofibroblasts with the release of extracellular matrix proteins [28,29].

On the other hand, RSV is the most frequent cause of lower RTI, mainly bronchiolitis, in infants [19]. Several epidemiological studies demonstrated that a history of severe RSV bronchiolitis is associated with subsequent persistent wheezing, childhood asthma or both [10,26]. RSV infection causes necrosis of the bronchiolar epithelium with subsequent submucosal oedema, recruitment of polymorphonuclear leukocytes and massive release of pro-inflammatory mediators, increased mucus secretion and bronchoconstriction. The disproportionate release of pro-inflammatory mediators induces a massive infiltration of monocytes and polymorphonuclear cells that may impair the immune response with a switch towards a Th1 response and a reduced IFN-γ-dependent viral clearance [30].

Respiratory viral infections may precede bacterial colonisation, as shown by positive nasopharyngeal culture for at least one bacterium among *Moraxella catarrhalis*, *Haemophilus influenzae* or *Streptococcus pneumoniae* in 60% of patients with respiratory symptoms lasting more than ten days [31]. The evidence on the role of airway bacterial colonisation and airway microbiome in wheeze and asthma pathogenesis is constantly increasing, with several studies showing that children with preschool wheeze have lower airway infection or colonisation with pathogens like *M. catarrhalis*, *H. influenzae* or *S. pneumoniae* [5,32,33,34,35,36].

In the prospective cohort Childhood Asthma Study (CAS), viral RTIs in the first two years of life have been associated with the prevalence of *M. catarrhalis*, *S. pneumoniae* and *H. influenzae* and persistent wheeze at five years old in those with early allergic sensitisation [36]. Schwerk and colleagues demonstrated with a retrospective analysis that patients with recurrent or persistent wheeze presented chronic bacterial colonisation with bronchoalveolar lavage (BAL) cultured positive for *H. influenzae, S. pneumoniae* and *M. catarrhalis* and airway neutrophilia [33]. This finding suggested that children with severe wheezing may be early colonised and may be particularly susceptible to these pathogens, leading to neutrophilia and chronic infection unresponsive to inhaled corticosteroids (ICS) [37]. However, randomised controlled trials showed no difference in the number of wheeze episodes and the need for oral corticosteroids in children treated with antibiotics [38,39].

Moreover, a significant relationship between *Mycoplasma pneumoniae* and *Chlamydophila pneumoniae* with wheezing in preschool children, particularly in subjects with a history of recurrent episodes, has been reported [40]. Children with wheezing and acute *M. pneumoniae* infection seem to have a specific cytokine profile characterised by a significant increase in serum levels of IL-5 [41].

Aberrant respiratory microbiota was evident since birth in children experiencing more than two episodes of respiratory tract infections suggesting a trajectory starting from the first month of life. These patients showed a decreased microbial community stability, a prolonged reduction of *Corynebacterium* and *Dolosigranulum*, and enrichment of *Moraxella* spp. [42]. A complex interplay between environmental factors and genetic predisposition is considered to shape the lung and gut microbiome in early life [43,44].

**Recommendation 1.** There is evidence that mainly viruses can trigger wheezing in young children. RSV and HRV are the main viruses involved in wheezing pathogenesis.

Quality of evidence: Moderate.

#### 3.1.2. PICO Question 2. What Is the Role of Atopy in the Pathogenesis of Preschool Wheezing?

##### Executive Summary

Compared to school-age asthma, which is typically allergic and characterised by type 2 inflammation, little is known about the immunopathology of preschool wheeze. Many children with EVW are not atopic, show bacterial or viral infection in the airways and do not benefit significantly from corticosteroids that are usually effective in allergic asthma, suggesting allergy is not the main driver of disease [44]. On the other hand, children with severe recurrent multi-trigger wheezing may be atopic and have evidence of increased numbers of eosinophils in BAL and endobronchial biopsies [44,45,46,47]. These children might be at higher risk of developing asthma at a later age [46]. Asthma and atopy share many genetic risk variants that dysregulate the expression of immune-related genes [48,49].

Lower airway eosinophilia suggests corticosteroid responsiveness, and in support of this, persistent wheezers show some benefit while taking ICS [7]. A prospective cross-sectional study performed by Guiddir et al. analysed airway inflammation with BAL and characterized a phenotype of severe recurrent wheezers with sensitisation to aeroallergen and response to ICS [50], suggesting that preschool wheezers with aeroallergen sensitization may also have lower airway eosinophilia. Three-year-old children with multi-trigger wheezing showed the classic pathological features of asthma, like submucosal eosinophilia and reticular basement membrane (RBM) thickening [37,47,50,51].

In a prospective study, Just et al. investigated the critical thresholds of common biological markers of atopy in persistent wheezy infants [52]. A cohort of infants (*n* = 219, <30 months old) with recurrent wheezing were enrolled and followed up until the age of 6. Blood eosinophilia (blood count ≥470/mm^3^) and elevated IgE (total serum IgE level ≥45 IU/mL) during infancy were associated with persistent wheezing at six years of age. The main discriminative parameter of wheezing persistence was eosinophilia: lack of eosinophilia in infancy could account for 91% of subjects in remission when combined with the absence of allergic sensitisation remission correctly predicted in 96.9% of the study population [52]. Children with aeroallergen sensitisation and/or blood eosinophils >300 cells/mm^3^ showed the greatest response to daily ICS in the Individualized Therapy for Asthma in Toddlers (INFANT) [53]. 

The role of IgE was also assessed during wheezing exacerbation in an observational study by Jartti et al. [54]. In 247 children hospitalised for wheezing (median age 1.6 years), atopy and number of exacerbations were closely related to HRV etiology (OR 4.59; 95% CI 1.78–11.8), followed by aeroallergen sensitisation (OR 4.18; 95% CI 2.00, 8.72), total IgE level (OR 2.06; 95% CI 1.32–3.21), food allergen sensitisation (OR 2.02; 95% CI 1.08, 3.78) and nasal eosinophil count (OR 1.52; 95% CI 1.08–2.13) [54]. Nasal eosinophils were found to increase during RTI in a cohort of 35 young children (age range, 6–33 months) and, after adjustment for age, sex, family history, and allergies were predictive of further episodes of wheezing over the subsequent two months (adjusted OR: 27.618, *p* = 0.016) [55]. Eosinophils were found to also increase in the sputum of preschoolers with severe wheeze and were associated with high blood eosinophil count, high serum total IgE and high allergen detection rate [56].

On the other hand, Ater et al. did not show any difference in sputum eosinophilia between preschoolers with wheezing and healthy peers but demonstrated that wheezers had higher asthma predictive index (S-API) and greater bronchial hyperresponsiveness [57]. Similarly, a positive API was associated with a higher risk of recurrent wheezing in infants (OR: 5.57; 95%; CI 2.23–7.96) [58].

The role of atopy may start early in utero, as suggested by an interesting case-control study where the cord blood of newborns at risk of atopy (family history of asthma) was stimulated for T cell cytokine production. At two years of age, children with wheezing presented increased production of T-cell cytokines IL-2 and IL-5, with IL-5 being the strongest risk factor associated with the development of wheeze (OR: 35; 95% CI, 5.0–246.7) [59].

**Recommendation 2.** Recurrent multi-trigger wheezing often presents a severe clinical spectrum, can be associated with atopy more frequently than EVW and might expose the child to a higher risk of developing asthma at a later age. Aeroallergen sensitisation and blood eosinophils can be used as biomarkers to identify responses to ICS in a recurrent preschool wheeze. 

Quality of evidence: Moderate.

### 3.2. Risk Factors for Wheeze Development

#### 3.2.1. PICO Question 3. Does the Presence of Risk Factors Such as Allergy/Atopy Influence the Onset and the Evolution of Preschool Wheezing?

##### Executive Summary

Multiple aeroallergen sensitisations are associated with persistent wheezing and progression to asthma [60,61]. Atopy and allergy have a pivotal role as risk factors in preschool wheezing, as shown by many studies conducted all over the past years.

In the Urban Environment and Childhood Asthma (URECA) longitudinal birth cohort study, cumulative exposure over the first three years of life to cockroach, mouse and house dust mite allergens in the home environment was associated with sensitisation to those allergens at age three and sensitisation was associated with recurrent wheeze at three years [62]. Particularly, a strong association has been found between atopy and the intermediate onset of wheezing (onset after 18 months of life) [63]. Furthermore, high blood eosinophil count (≥470/mm^3^), allergic sensitisation and a family member (father) with asthma have been implicated in the persistence of wheezing [64]. A sex-dependent association between parental allergic conditions (asthma) and the prevalence of wheezing in offspring has been found: maternal asthma seems to be associated with asthma in girls but not in boys, while the opposite is seen for paternal asthma [65].

Wheeze, allergic rhinitis and atopic eczema were more frequent in children with high total and specific IgE [66]. In particular, anti-cockroach and anti-mouse IgE were specifically linked with the risk of developing both wheezing and atopy [67]. Interestingly, good control of allergic rhinitis reduced the risk of acute exacerbation of wheezing in the first six years of age [68].

A further correlation has been suggested between eczema and wheezing: eczema, and especially early eczema, has been associated with an increased risk of childhood asthma [69,70] but also with an increased risk of developing allergic airway diseases later in life [71]. Furthermore, the risk of developing asthma increases when eczema is combined with wheezing episodes in infancy [72]. Another study has combined eczema and sensitisation to inhaled allergens with hospital admissions for wheezing in the first three years of life, thus resulting in eczema as a strong predictor of asthma [73]. The combination of eczema and food allergy was associated with an increased risk of asthma at age four years, and this risk was higher if the allergy was proven for two or more foods [74].

Interestingly, Illi et al. investigated the pattern of atopic sensitisation typically associated with the development of asthma in childhood, showing that sensitisation to any allergen early in life and sensitised to inhalant allergens by the age of seven years was associated with a significantly increased risk of being asthmatic and maternal transmission may determine both a certain pattern of sensitisation and the expression of asthma [75]. Moreover, they showed that the chronic course of asthma characterised by airway hyper-responsiveness and impairment of lung function at school age is determined by continuing allergic airway inflammation beginning in the first three years of life [76]. However, children with a non-atopic wheezing phenotype lose their symptoms over school age and retain normal lung function at puberty.

**Recommendation 3.** Young children with recurrent wheezing and atopic eczema, sensitised to allergens or blood eosinophilia, are at higher risk of asthma at a later age.

Quality of evidence: High.

#### 3.2.2. PICO Question 4. Does the Presence of Risk Factors Such as Previous Respiratory Tract Infection or Bronchiolitis Influence the Onset and the Evolution of Preschool Wheezing?

##### Executive Summary

By applying metatranscriptomic, transcriptomic, and metabolomic approaches to infants with bronchiolitis, recent studies found an interplay between major pathogens, their function, and host response in the airway, and their longitudinal relationship with asthma development [42,43].

Viral RTIs occurring early in life have been accounted for as important triggers of wheezing episodes in infancy, and many studies have shown an association with the subsequent development of persistent wheezing in preschool age. A meta-analysis including 22 cohort studies assessed the association between wheezing and lower RTIs (LRTIs) in childhood, demonstrating that LRTIs before three years of age increased the risk of wheezing development not only in childhood but also in adolescence and adulthood [77]. Other observational studies showed that LRTIs occurring during the first three years of life were associated with increased wheezing episodes and overall respiratory morbidity (i.e., bronchitis, pneumonia) in the following two years [18,78,79,80]. This was particularly evident in children with a family history of atopy or allergy. The risk of developing wheezing following an LRTI like bronchiolitis increases for children with congenital heart disease [81] or children exposed to tobacco smoke [82]. 

Bronchiolitis is one of the commonest LRTIs in infancy. Epidemiological data show that 3–5% of infants develop severe bronchiolitis requiring hospitalization during their first year of life and that more than 30% will develop recurrent wheezing and asthma [83]. Being hospitalized for bronchiolitis in the first year of life can be a risk factor for preschool wheezing [84,85], particularly in children with respiratory comorbidities and atopy [86]. The need for intensive care for RSV-induced bronchiolitis or severe infections RSV-related were associated with an increased risk of recurrent wheezing episodes by age three years and asthma by age four years [87,88]. Two systematic reviews and other observational studies confirmed the association between RSV infection requiring hospitalization in infancy and wheezing or asthma development [89,90,91,92]. Similar results were obtained when studying children born preterm [93,94,95]. Recently, a large retrospective study on 68,130 infants reported that those (30.7%) hospitalized for RSV-bronchiolitis had more than a 2-fold risk of developing recurrent in the first year of age [96]. A recent meta-analysis of 35 studies estimated the effect of RSV infections on later wheezing as OR 4.17 (95% CI 2.36–7.37), but after adjustment for genetic and environmental influences, the effect size reduced by about 50% (aOR 2.45 (95% CI 1.23–4.88), suggesting that the association was non-causal [97].

Despite the results of one study where no association was found between other viruses different from RSV and recurrent wheezing at five years [94], increasing evidence shows that HRV is deeply implicated in the development of wheezing and asthma later in life. HRV infection is the most common cause of LRTI and wheezing after six months of age [98] and the most common trigger of acute preschool wheeze episodes [99]. A prospective cohort study comparing the clinical differences between HRV and RSV-induced wheezing showed that children infected by HRV experienced wheezing more often and earlier than children infected by RSV [100]. HRV infection increases the risk of wheezing requiring hospital admission [101], and the risk of wheezing episodes in the following year [25]. If HRV results in bronchiolitis in atopic subjects, there is a major risk of developing recurrent wheezing and subsequent asthma, compared to RSV-infected atopic subjects [102,103]. A recent meta-analysis summarized this evidence and showed that children with HRV-bronchiolitis were more likely to develop recurrent wheeze than subjects affected by RSV-bronchiolitis (OR 4.11; 95% CI 2.24–7.56) [101].

A single prospective cohort study by Reimerink et al. investigated the relationship between early intestinal viral infections and the development of eczema, wheeze and atopic sensitization during the first and the second year of life [104]. Seropositivity for immunoglobulin (Ig)G for Rota- and Norovirus (GGI.1 and GGII.4) at one year of age was related to early allergic sensitization (specific IgE), parental reported eczema and wheeze in the first two years of life. Furthermore, Rotavirus seropositivity was associated with an unexpectedly higher risk of recurrent wheeze in the first and second year of life and persistent and new recurrent wheeze (adjusted OR 2.7 and 95% CI 1.1–6.2) [104].

**Recommendation 4.** Infants with bronchiolitis represent a high-risk group for recurrent wheezing. 

Quality of evidence: High.

#### 3.2.3. PICO Question 5. Does Pollution Influence the Onset and the Evolution of Preschool Wheezing?

##### Executive Summary

Environmental pollution has been consistently associated with negative effects on the respiratory health of children [105]. Children are particularly sensitive to the damages of inhaled pollution since they undergo rapid lung growth, have higher respiratory rates and have the greatest exposure since they spend most time outdoor [105]. However, most of the studies on the association between pollution and wheeze development consist of observational studies. 

Increasing attention has been paid to traffic-related air pollution (TRAP) as a risk factor for respiratory diseases. Some studies suggested that exposure to moderate levels of TRAP can favour the development of persistent wheezing [106,107], but the duration of the exposition seems crucial. Exposition to pollutants in the first four years of life leads to an increased risk of wheezing exacerbations, although only a continuous exposition in the first seven years may lead to the development of asthma [108]. Other studies confirmed that a certain degree of correlation exists between TRAP and the incidence of preschool wheezing but not with the incidence of asthma [109]. TRAP exposure may be implicated in more frequent and severe exacerbations in children already suffering from recurrent wheezing, whereas living in green areas may be protective [110].

Some studies on ex vivo bronchial epithelial cells confirm a direct correlation between air pollution, viral infections and wheezing exacerbations. Short-term exposure to high levels of nitrogen dioxide (NO_2_) and particulate matter (PM)10 may favour wheezing since this exposure seems associated with reduced interferon-β response to viral infections [111]. The same group demonstrated that in children with wheezing, prolonged exposure to PM10 correlated with RBM thickness and airway eosinophilia, likely contributing to the development of asthma by airway remodelling and inflammation [112].

Other studies have focused particularly on the type and levels of TRAP, trying to deduce differences between different pollutants. Some studies have not found specificity considering PM2.5, PM10 or NO_2_ levels involved in wheezing exacerbations [113], while others have found certain differences. Particles with lower diameters (especially PM1 and PM2.5) are associated with higher risk than particles with bigger diameters [114,115]. The role of pollutants deriving from industrial emissions (oil refineries, metals smelters or others types of industry) has been assessed in an interesting study where the exposure has been linked to hospital admission for wheezing diseases [116]. 

Since defining the burden of a single pollutant on wheeze development is very difficult, studies often take into account the role of co-variables. In a wide case-control study [114], the combination of parental asthma, parental education, maternal smoking during pregnancy, and TRAP has been linked to an increased risk of developing persistent wheezing and asthma. Moreover, RNA-based epigenetic mechanisms—mainly microRNA post-transcriptional regulation—could serve as key epigenetic mediators of the link between air pollution and asthma [117,118,119]. Heterogeneity in the definitions of TRAP exposure and asthma outcomes has led to confusion in the field [118,119]. However, novel information regarding the molecular characterization of asthma phenotypes, TRAP exposure assessment methods, and epigenetics are revolutionizing the field.

The so-called indoor pollution also has a role in affecting respiratory health. Some studies have investigated household indicators of dampness (visible mould spots, window pane condensation or damp stains) and found they were associated with preschool wheezing [113,120], in particular with early onset wheezing and delayed remission. In a comprehensive prospective cohort Japanese study, mould growth and wood stove/fireplace were related with significantly higher ORs for wheezing (mould growth: 1.13; 95% CI, 1.06–1.22; wood stove/fireplace: 1.23; 95% CI, 1.03–1.46) [121]. The use of household chemicals and other cleaning products may also have a role in respiratory outcomes, but the evidence is still scant [122,123]. 

Particular attention should be paid to the prenatal and perinatal periods. Urbanization levels (according to various indicators), alongside sex, age and geographic region, have been significantly associated with prematurity and wheezing exacerbations [124]. Furthermore, prenatal exposure to certain molecules (high-molecular-weight phthalates, bisphenol-A) might give rise to wheezing symptoms and respiratory tract infections throughout childhood and asthma later in life [125]. 

**Recommendation 5.** Traffic-related air pollution may favour wheezing via a reduced response to viral infections. Both outdoor and indoor pollution can influence the respiratory health of young children from conception and birth. 

Quality of evidence: Moderate.

#### 3.2.4. PICO Question 6. Does Genetics Influence the Onset and the Evolution of Preschool Wheezing?

##### Executive Summary

Genome-wide association studies (GWAS) on preschool wheezing have been gradually published over the years, and evidence has begun to emerge regarding specific loci of susceptibility involved in this disease. Single nucleotide polymorphisms (SNPs) have been identified, and a specific locus of chromosome 17 (17q12-q21) has been the object of many studies [126,127,128,129,130,131,132,133].

One study has linked an SNP, rs7216389, with an increased risk of recurrent wheezing but also with asthma and asthma exacerbations [126]. This correlation is present from preschool to school age, but its role has been ascertained only for early-onset disease and not late-onset. A case-control study goes in the same direction, linking rs7216389 SNP with early-onset wheezing (until five years of age) but not with adult-onset asthma [127]. 

Many studies have focused on the association of SNPs of inflammatory cytokines with preschool wheezing. IL-10 rs1800896 SNP (in heterozygosity state) is significantly associated with the development of preschool wheezing after a severe LRTI in early infancy [128]. On the other hand, homozygosity for rs1800896 allele G (genotype G/G) represents a protective factor from asthma development [128]. Another SNP regarding IL-4, rs2070874 and specifically the genotype T/T, has been associated with a severe phenotype of viral-induced wheeze [129]. 

Studies on IL receptors have also been conducted. The homozygous variant of IRAK4 (IL-1 receptor-associated kinase-4), rs4251513, has been constantly associated with post-bronchiolitis wheezing episodes and asthma medication use at school-age [130]. IL-10, IL-4, IRAK-4 and many others are implicated in the inflammation pathways leading to viral respiratory infections.

Some genetic studies have also investigated the role of Toll-like receptors (TLR) after bronchiolitis. In particular, TLR-1 SNP rs5743618 has been associated with increased asthma prevalence during the first six years of life if bronchiolitis had been contracted within the first six years of life [131]. However, TLR-2 SNP rs5743708 did not show any correlation with wheezing [131]. In addition, an SNP in TLR-10, rs4129009, has been associated with preschool wheezing after an episode of bronchiolitis in infancy [132].

Other molecules have been investigated to better explain wheezing development in certain subjects. A case-control study conducted in preschool wheezers prospectively followed until six years of age found that intercellular adhesion molecule 1 (ICAM-1) SNP rs5498 was positively associated with asthma development [133]. A study on filaggrin (FLG) loss-of-function mutations highlighted its role in the pathogenesis of asthma and food sensitization [134].

However, although numerous studies investigated genetic susceptibility, the evidence of important genes being responsible for preschool asthma is low. 

**Recommendation 6.** Some individuals have a genetic susceptibility and are predisposed to develop preschool wheezing at first and eventually asthma later in life. At present, little can be done to modify genetic susceptibility, but environmental exposures can be adjusted to reduce this risk and potentially work on primary asthma prevention.

Quality of evidence: Low.

#### 3.2.5. PICO Question 7. Does Obesity Influence the Onset and the Evolution of Preschool Wheezing?

##### Executive Summary

Obesity is an important risk factor and a disease modifier for many respiratory diseases, including childhood asthma. This condition increases susceptibility to respiratory infections and hospitalization [135].

Excessive weight gain in early life is considered a risk factor for wheezing in preterm and term babies. Preterm babies are at particular risk for this condition since they often experience the so-called catch-up growth, an increased growth rate following low birth weight and intrauterine growth retardation. In a cohort of children born preterm, accelerated foetal growth between the first trimester of pregnancy and birth was associated with increased wheeze-ever [136]. Compared to term-born children without weight gain in the first nine months of life, children born preterm (≤32 weeks of gestational age) with rapid weight gain had a fivefold higher risk of wheeze-ever (OR 5.04; 95% CI 3.36–7.54) [136].

Kotecha et al. specifically studied the effect of catch-up growth on wheezing phenotype, which was associated with early wheezing but not with persistent or late wheezing, suggesting that different mechanisms, such as atopy, may be more important in later age [137]. Rapid weight gain in early life may lead to impaired growth of the lungs, a condition known as dysanapsis, whereby somatic growth exceeds that of lung growth [137]. In addition, the adipose tissue may release pro-inflammatory mediators like leptin, which have been associated with airway remodelling [138].

A similar risk and lower lung function were also found in children born at term with rapid weight gain in early life [139,140]. Some longitudinal studies have investigated the relationship between overweight and respiratory diseases in toddlers and older children [141,142,143,144]. A prospective cohort study of 731 children aged three to eight found that increased body mass index (BMI) at three and five years of age was associated with a higher risk of recurrent wheezing with no differences between girls and boys [142]. This was not true in the eight-year-old group, where an increased BMI was associated with an increased risk of recurrent wheezing in girls but not boys [142]. Data analysis from the combination of eight different prospective birth cohort studies, including 12,050 children, demonstrated that children with rapid BMI gain in the first two years of life were at higher risk for incident asthma up to age 6 compared with children with less pronounced weight gain slope in early childhood [143]. In the CHILD Cohort Study including 3154 children followed up from three months to five years of age, higher BMI at one year of age remained an independent risk factor for all the wheeze trajectories (transient wheeze, intermediate-onset wheeze and infantile-onset persistent wheeze) after adjustment for other factors [144]. 

**Recommendation 7.** Rapid weight gain in infancy and high BMI is associated with an increased risk of wheezing in preschool age.

Quality of evidence: Low.

#### 3.2.6. PICO Question 8. Do Prematurity and Other Perinatal Factors Influence the Onset and the Evolution of Preschool Wheezing?

##### Executive Summary

In addressing birth-related risk factors for wheezing and asthma, the impact of premature birth must be considered. The prevalence of premature birth (<37 weeks gestation) is about 10% worldwide [145]. Over the years, many studies investigated the association between gestational age and childhood wheezing disorders [146,147,148,149], showing that preterm birth was associated with an increased risk of wheezing [147,148,150]. The population is usually stratified in very preterm (<32 weeks of gestation), late preterm (32–36 weeks of gestation), early term (36–38 weeks of gestation) and term (38–41 weeks of gestation) infants and findings show that the risk of wheezing is proportional to prematurity with the highest risk in children born very preterm, particularly if they develop bronchopulmonary dysplasia [147,148,150,151]. 

When preterm birth is combined with a personal history of atopy and living with two or more children, the risk of recurrent wheezing increases [152]. However, when interpreting these findings as suggested by a systematic review and meta-analysis on this topic, several factors (i.e., sex, maternal smoking, restricted growth and parental atopy or asthma) may confound the association between preterm birth and wheezing disorders since for example, maternal smoking may be a trigger both for preterm birth and for wheezing as well as intrauterine growth restriction [148].

A growing body of literature has highlighted the importance of prenatal and perinatal factors on the subsequent development of recurrent wheezing and asthma. Considering prenatal factors, many studies sustained that an increased risk of childhood wheezing disorders may begin as early as an in utero exposure to maternal ascending infection [153], chorioamnionitis [154], mother’s quality of life (QoL) [155], stress [156,157,158] or depression [155]. Furthermore, a correlation between caesarean delivery (CD) and the risk of developing wheezing in the first three years and asthma in the first four years has been hypothesized [159,160]. Moreover, asthma exacerbations during pregnancy in women with asthma showed an increased risk of early childhood respiratory disorders in their children [161]. All these factors can result in smaller airways, altered foetal immune responses and low birth weight. Birth weight is one of the main predictors of lung function in infants [162], children [163] and also in later age as demonstrated by the linear relationship with lung function at age 45–50 years found in the Aberdeen Birth cohort [164]. Furthermore, low birth weight, particularly if associated with intrauterine growth restriction, is a well-known risk factor for wheezing and asthma [165]. Perinatal factors, including infections, hyperoxia, and mechanical ventilation, are all associated with premature birth and negatively impact lung development and airway reactivity [148]. 

Overall, children born preterm experience disruptions in lung development and may experience significant shifts from the physiologic lung function trajectory and are at higher risk for respiratory morbidity throughout life [166]. Especially children born very preterm (<28 weeks of gestational age) are at the highest risk for bronchopulmonary dysplasia and can show obstructive patterns in lung function, eventually developing chronic obstructive pulmonary disease in adult age [166].

**Recommendation 8.** Preterm birth and low birth weight are important early life risk factors for wheezing disorders in childhood. Extremely preterm infants are at the highest risk for respiratory problems and may have lower lung function trajectories across all ages.

Quality of evidence: Moderate.

#### 3.2.7. PICO Question 9. Does Smoke Exposure Influence the Onset and the Evolution of Preschool Wheezing?

##### Executive Summary

The largest body of evidence on environmental risk factors affecting wheeze development relates to cigarette smoking. Tobacco smoke exposure antenatally in utero was associated with reduced lung function at birth [158] and later in life [167], with wheezing and asthma in childhood [168] and more globally with negative interference in lung function and life trajectories [169,170].

Two large independent meta-analyses documented maternal smoking and passive smoke exposure confer the risk of wheezing and asthma in preschool children, particularly children with a family history of allergy [171]. The risk was highest in children exposed to both passive smoking and mothers smoking actively during pregnancy [171,172]. In a recent French study, 129 infants under two years admitted to the hospital for acute wheezing were assessed for prenatal smoke exposure [173]. Children exposed (36.4%) had a longer length of hospital stay, and the authors estimated that smoking one cigarette/day during pregnancy was associated with an increase in hospitalization duration of 0.055 days/month (r = 0.238, *p* = 0.006) [173]. Consequences of prenatal and postnatal smoke exposure on wheezing in childhood have also been confirmed in two recent case-control studies [174,175]. A prospective birth cohort study found that the combination of prematurity and maternal smoking during pregnancy synergistically increases recurrent wheezing and the number of episodes in early childhood [176]. However, future studies are needed to define better and quantify the exposure level since heavy parental smoking may be associated with different phenotypes of wheezing [177]. In a study conducted on 150 children with recurrent wheezing, 91 had been exposed to lower-level second-hand tobacco smoke, 24 were exposed to higher-level second-hand tobacco smoke, and 35 were not exposed to cigarette smoke. Wheezing symptom scores were higher in highly exposed children (*p* = 0.03) [178]. An interesting study has investigated one possible mechanism underlying the relationship between cigarette smoke exposure and preschool wheezing, suggesting an interaction between the genome and this environmental factor. Children with a specific polymorphism of IL-13 and in utero exposure to smoke were at higher risk of wheezing at age four and persisting asthma at age ten years [179].

Passive smoke also includes third-hand smoke, which is the smoke that stays on every surface in the area where someone has been smoking, including on skin, hair, clothing, furniture and flooring [180]. Due to particular habits of children like hand-to-mouth eating, crawling and sucking, these subjects may be particularly vulnerable to the effects of third-hand smoke. At present, there are no studies on the effect of third-hand smoke on wheezing preschool development, but we can speculate that this exposure can contribute to increasing airway reactivity in young children.

**Recommendation 9.** Maternal smoking during uterine foetal life and subsequent second and third-hand smoke exposure increase the risk of wheezing in preschool children, particularly those with a family history of allergy.

Quality of evidence: Moderate.

#### 3.2.8. PICO Question 10. Is Immunodeficiency a Risk Factor for the Onset and the Evolution of Preschool Wheezing?

##### Executive Summary

Primary immunodeficiency disorder (PID) is a heterogeneous group of disorders resulting from innate or adaptive immunity defects. The clinical spectrum presented by patients with PID is variable but increased susceptibility to infections is a common feature [181]. PID must be suspected in case of persistent wheeze refractory to therapies and a history of pulmonary or systemic infections with unusual organisms [182], although some data did not show an association between PIDs and recurrent wheezing [183]. 

Selective IgA deficiency is one of the most common PIDs, occurring in approximately 1 in 300 people [181]. An undetectable level of IgA defines IgA deficiency in the blood and secretions but no other immunoglobulin deficiencies. The IgA antibodies present in the secretions play a major role in protecting from respiratory airways and gastrointestinal tract infections. In children, the main infections associated with IgA deficiency are pharyngotonsillitis, otitis, bronchitis, sinusitis and, less frequently, pneumonia [182].

In a cohort of Swedish children with positive skin prick test to at least one allergen and with circulating IgE antibodies to egg or cat, development of late-onset wheezing (at four years of age) was reduced by high levels of secretory salivary IgA levels (*p* = 0.04) [184]. On the other hand, low levels of IgA in the respiratory mucosa might predispose to develop bronchial hyperresponsiveness and asthma [185]. In addition, IgA deficiency may predispose subjects to allergy, but underlying mechanisms need to be clarified [186]. 

Transient dysfunctions of the humoral immune system include reduced IgG subclasses (IgG1, IgG2, IgG3, IgG4). The most common IgG1 deficiency results from a generalized deficiency of antibodies, IgG2 deficiency is associated with recurrent viral and bacterial infections, both IgG2 and IgG3 deficiency predisposes to recurrent respiratory tract infections, and IgG4 deficiency has been found in chronic bronchial and lung diseases [187]. Two studies showed that low levels of the IgG4 subclass were associated with recurrent wheezing, requiring hospitalization in infants and young children [188,189].

**Recommendation 10.** PID must be suspected in case of persistent wheeze refractory to therapies and a history of pulmonary or systemic infections with unusual organisms. IgA deficiency can predispose the child to recurrent respiratory infections, including wheezing in predisposed subjects.

Quality of evidence: Moderate.

### 3.3. Protective Factors for Wheeze Development

#### 3.3.1. PICO Question 11. Are Probiotics Protective for Preschool Wheezing Development?

##### Executive Summary

Diversity and maturity of gut and lung microbiota can influence the onset and progression of allergic diseases, including asthma, since early life [190,191,192,193]. Bacterial colonisation starts in utero [194]. Then, at birth, skin, gut and lung microbiome start establishing. In the gut with dysbiosis, bacteria of the *Bacteroidetes* phyla are more present than *Firmicutes* phyla, resulting in a reduction of short-chain fatty acids (SCFA) production that may result in the secretion of pro-inflammatory cytokines such as IL-6, IL-8 and TNF-α and reduction of Treg lymphocytes favouring the development of inflammatory disease such as asthma [191]. 

In the Copenhagen Prospective Studies on Asthma in Childhood 2010 (COPSAC2010), particular bacterial colonisation of the infant’s gut in the first month of life was related to the risk of wheezing and asthma at six years of age [195]. In another study, the increase of opportunistic pathogens like *Enterococcus* and the decrease of *Eubacterium*, *Faecalibacterium* and *Bifidobacterium* in early life may increase the risk of respiratory infection [196]. Similarly, in the Asthma Detection and Monitoring (ADEM) study, 202 wheezing children and 50 healthy children aged 2–4 years were studied until the age of six years, showing that bacterial composition and maturation of intestinal microbiota in the first months of life may influence the risk of asthma at school age, especially in children born to asthmatic mothers [197]. In the CHILD (Canadian Healthy Infant Longitudinal Development) birth cohort study, wheezing and atopy at one year of age were associated with decreased abundance of *Faecalibacterium*, *Lachnospira*, *Rothia*, and *Veillonella* in faecal microbiota at three months [198]. On the other hand, in a Swedish study, higher microbial diversity during the first month of life was associated with protection against the development of asthma at seven years of age [199]. 

Over the last decade, several studies reported on the use of probiotics in order to modulate the gut microbiome composition and development of respiratory disease. The potential beneficial effects of probiotic immunomodulation include increased synthesis of IgA and IL-10, suppression of tumour necrosis factor (TNF)-alfa and the inhibition of casein-induced T-cell activation [200].

Jensen et al., in a randomised controlled trial of 123 five-year-old children, demonstrated that early-life supplementation with probiotics did not change allergic disease prevalence [201]. On the contrary, there was instead a subtle trend in the probiotic group towards developing more recurrent wheezing episodes [201]. Similar results were reported in high-risk infants with oral supplementation of *Lactobacillus rhamnosus* GG during the first six months of life [202]. Further research on the relationship between probiotic supplementation and wheezing risk in three-year-old children was the trial by Berni Canani et al. [203]. In this randomised controlled trial, authors demonstrated that probiotics associated with extensively hydrolysed casein formula (EHCF) could reduce allergic manifestation, including asthma, in children with cow’s milk allergy (CMA) and, therefore at high risk of atopy [203]. These studies were included in a recent meta-analysis including 13 trials for a total of 4021 children [204], showing that, despite some effectiveness in eczema prevention, probiotic supplementation during pregnancy or in early life did not reduce the incidence of asthma or wheezing in infants, except for the subgroup of atopic infants [201,202,203]. Further systematic reviews and meta-analyses did not confirm the protective effect of probiotic administration in the first years of life on the risk of childhood wheezing and asthma [204,205,206]. The meta-analysis by Wei et al., including 2521 children, showed that compared to placebo, probiotic supplementation reduced wheeze episodes only in a small subgroup of atopic infants [204]. The prophylactic effect was not influenced by other factors (i.e., asthma risk factors such as personal medical history or positive family history, type of probiotic used, timing and duration of intervention and follow-up) [204]. Similar results were obtained when administered antenatally to the mother and postnatally to the child [207].

Studies show controversial results when probiotics are administered in children with wheezing or asthma. A recent meta-analysis found an association with fewer exacerbations but no difference in asthma control test (ACT), respiratory symptoms or lung function [208]. Rose et al. conducted a double-blind, randomised controlled trial, which included 131 infants (aged 6–24 months) with at least two wheezing episodes and a family history of atopy [209]. The supplementation of *Lactobacillus rhamnosus* GG (LGG) did not prevent asthma or wheezing over a six-month follow-up. On the other hand, the study showed that in the subgroup sensitised to aeroallergens, the asthma symptoms score was higher in the LGG-supplemented subgroup than in the placebo group (22.9 vs. 42.5) [209]. The same authors in another trial studied the effects of probiotic supplementation on asthma exacerbations in a longer follow-up (44 months) and demonstrated no difference in terms of exacerbations between the probiotic and placebo group [210]. A meta-analysis of 25 studies demonstrated that probiotics do not influence asthma or wheeze development but may reduce IgE blood levels and the risk of atopic sensitisation when administered early in life [211]. 

**Recommendation 11.** Probiotic administration to reduce wheezing development is not recommended. 

Quality of evidence: High.

#### 3.3.2. PICO Question 12. Is Vitamin D Supplementation Protective for Preschool Wheezing Development?

##### Executive Summary

Vitamin D is mainly formed in the skin from 7-dehydrocholesterol after UVB exposure. Many foods are rich in vitamin D, such as plants, fish, eggs and liver [211,212]. The vitamin is then activated, firstly in the liver and then in the kidneys. Vitamin D seems to play a role in asthma control, given its effects on immune cell function, oxidative stress, airway remodelling and corticosteroid responsiveness through various pathways, including those involving IL-10 and IL-17 [213,214,215,216,217,218].

The potential preventive role of vitamin D starts in utero. Mothers with asthma and vitamin D sufficiency had a lower risk of offspring with asthma or recurrent wheezing by age three years [219,220,221]. On the contrary, low levels of vitamin D in mothers during gestation are important risk factors for decreased lung function at ages four, five, and six years [222]. In a recent meta-analysis including 6068 subjects, there was an inverse relationship between the intake of vitamin D during pregnancy, and the occurrence of wheezing in offspring (pooled OR: 0.68; 95% CI: 0.55–0.83) found [223]. However, it has been argued that supplementation may not work not for all the mothers since only the presence of maternal 17q21 functional SNP rs12936231 genotype confers a protective effect of high-dose prenatal vitamin D3 supplementation against offspring asthma/recurrent wheeze at age 0–3 years [224].

Considering vitamin D levels in children, higher levels are associated with lower frequency, duration and severity of wheezing attacks [225,226,227,228,229,230,231,232]. In 131 young children with recurrent wheezing, a suboptimal vitamin D status increased the risk of asthma exacerbation in the previous month, and a recent exacerbation was associated with low levels despite oral supplementation [233]. 

However, conflicting results have been reported for vitamin D supplementation and the prevention of RTIs and wheezing. Ducharme and colleagues investigated the effects of vitamin D supplementation on upper RTIs, asthma exacerbations, OCS use and emergency care evaluation in children between one and five years of age [234]. No difference was found between subjects supplemented with vitamin D and the placebo group. Similarly, oral supplementation for the first six months of life did result in the prevention of asthma, wheezing, and other atopic diseases (food allergy, rhinitis, eczema and allergen sensitization) at 2.5 years [235]. A systematic review and meta-analysis on nutrients included six studies performed in children and found no significant decreased incidence of RTIs with vitamin D supplementation (RR 0.88; 95% CI: 0.66–1.11, *p* < 0.0001) [236].

On the contrary, in a large meta-analysis including both children and adults, vitamin D’s overall effect is positive in reducing RTIs, particularly in deficient subjects [237,238]. Interestingly, patients with moderate to severe asthma treated with ICS and with vitamin D supplementation had a reduced risk of asthma exacerbation (pooled RR 0.70; 95% CI, 0.59–0.83; *p* < 0.05) [239].

**Recommendation 12.** Vitamin D supplementation during the winter season may decrease the risk of RTIs and wheezing exacerbations. 

Quality of evidence: Moderate.

#### 3.3.3. PICO Question 13. Is Breastfeeding Protective for Preschool Wheezing Development? 

##### Executive Summary

In a prospective cohort study conducted on 8499 children, breastfeeding for at least three months was associated with a lower risk of asthma between the ages of two and five [240]. Compared to exclusive breastfeeding, any other method of infant feeding was associated with an increased risk of asthma [241]. However, when considering hospitalization of asthma exacerbations in school-aged children, the data failed to demonstrate a protective effect of breastfeeding [242].

In addition to maternal antibodies, breast milk contains other immunomodulatory mediators, including activated T cells and memory T cells, secretory IgA, oligosaccharides, antibacterial proteins such as lactoferrin, lysozyme, beta-lactoglobulin, casein and pro- and anti-inflammatory factors that confer passive protection against incidence and severity of respiratory infections [241].

The protective effect of breastfeeding is particularly evident in the first years of life, as demonstrated by a recent meta-analysis where a lower risk of asthma is seen in children younger than seven years [243]. In the large birth cohort study, ALSPAC (Avon Longitudinal Study of Parents and Children), breastfeeding for at least six months reduced the risk of asthma in the first three years of life but not between the ages of 7 and 8 [244]. These findings were also confirmed by the metanalysis from Dogaru et al., which demonstrated that breastfeeding for more than six months reduced the risk of asthma and wheezing only for children younger than two years of age and not for older children [245].

**Recommendation 13.** Maternal breastfeeding protects from preschool wheezing. 

Quality of evidence: High.

#### 3.3.4. Question 14. Is Influenza Vaccination Protective for Preschool Wheezing Development?

##### Executive Summary

Influenza virus was detected in 8% of young children with wheeze hospitalized for an LRTI [15]. The risk of influenza infection and wheezing in children with EVW can be reduced through influenza vaccination [246]. All health authorities recommend annual influenza vaccination for subjects ≥6 months of age with asthma [247,248]. 

In children, the most commonly administered influenza vaccines are inactivated influenza vaccines (IIV), the efficacy of which has also been proven in children with recurrent wheezing on steroids [249]. The quadrivalent live-attenuated influenza vaccine (LAIV4) can be a suitable alternative for children ≥2 years of age [250]. LAIV4 is an intranasally administered vaccine that is very popular among parents and healthcare professionals due to the non-invasive route of administration. However, a diagnosis of asthma or a wheezing episode in the previous 12 months represents a contraindication for LAIV4 in children aged two to four years because of a reported higher incidence of wheezing in LAIV recipients compared with IIV recipients [250,251]. This contraindication has been questioned and many studies also demonstrated the vaccine’s safety in the population of preschool wheezers and severe asthmatic patients [252,253,254]. In a recent UK multicentre, open-label, phase IV intervention study in preschool children with recurrent wheezing, a follow-up of asthma symptoms 72 hours and four weeks later showed no significant change after administration of LAIV in the presence of efficacy against influenza [255,256]. LAIV4 has also been investigated concerning long-term effects, and in a prospective cohort of vaccinated children who were 12 through 35 months of age, there was no evidence of increased risk of subsequent asthma diagnosis at 14 years of age [252]. 

**Recommendation 14.** Influenza vaccination is recommended for its efficacy and safety in young children ≥6 months of age with wheezing.

Quality of evidence: High.

#### 3.3.5. Question 15. Are Immunomodulators Protective for Preschool Wheezing Development?

##### Executive Summary

Recurrent RTIs are one of the most common diseases in children [257,258,259]. Despite being generally mild, recurrent RTIs may disrupt children’s growth, contribute to antibiotic misuse, generate a significant burden of care and be a significant risk factor for asthma in later life [257]. The underlying pathogenesis of recurrent RTIs is complex. Immunological immaturity, genetic characteristics and environmental factors, such as exposure to air pollutants, attendance of daycare and lack of breastfeeding, are considered the most important factors promoting RTIs [258]. Boosting the immune system activity with non-specific immunomodulators can be an option to improve protection against infections [259]. 

Over the years, clinical evidence for the role of non-specific immunomodulators in reducing recurrent RTIs in the paediatric population has consistently increased [259]. Once administered orally, non-specific immunomodulators stimulate innate and adaptive immunity through different mechanisms. OM-85 is a non-specific lyophilised bacterial lysate of common pathogenic bacteria of the respiratory tract. In the mechanism of action of OM-85, the gut–lung immune axis plays a crucial role. OM-85 induces the maturation of human dendritic cells in gastrointestinal Peyer’s patches, stimulates macrophages in the production of proinflammatory cytokines and anti-viral chemokines, up-regulates the Th1 specific cytokine IFN-γ and down-regulate the Th2 specific cytokine IL-4 and increases the activity of B cells with the secretion of salivary IgA, bronchoalveolar IgA, and serum IgA and IgG [259]. A double-blind, randomised, controlled study involving 288 children aged between one and six years with a history of recurrent RTIs (at least six episodes in the previous year) showed that OM-85 reduced the number of new RTI episodes when administered for the first ten days of each month for three months (33% vs. 65.1% in children with placebo, *p* < 0.0001). This was associated with fewer days of absence from daycare for children and working days lost by parents [260]. So far, several meta-analyses have been published on the effect of OM-85, demonstrating that it reduces the frequency and length of RTIs and eventually limits the use of antibiotics. Overall, data suggest that the effect is greater in patients at increased risk of recurrent RTIs. Some authors recently speculated that this non-specific immuno-modulator might also be useful against COVID-19 infection [261,262,263].

Interestingly, since non-specific immunomodulators correct Th1/Th2 imbalance through activation of T regulatory (Treg) cells and promote the immune system’s maturation in children, their use can be associated with the reduction of Th2 atopic responses associated with wheezing and asthma [264]. Razi et al.’s study showed that prophylaxis with OM-85 reduced the duration and incidence of exacerbations in children with a history of recurrent wheezing [265].

**Recommendation 15.** Prophylaxis with non-specific immunomodulators can be considered in children with recurrent EVW to reduce the number of episodes during the winter season.

Quality of evidence: Moderate.

## 4. Discussion

This study shows that atopy and respiratory infections are pivotal in preschool wheezing pathogenesis. Multi-trigger wheezing is often associated with atopy and recurrent severe clinical phenotype. The evidence of blood eosinophilia and allergen sensitisation may guide the treatment in favour of ICS. HRV and RSV are the most common viruses associated with a higher risk of developing asthma at later ages. Bacterial colonisation and airway neutrophilia may characterise a unique phenotype of preschool wheezing not responsive to steroids.

Genetics and modifiable risk factors such as obesity, pollution or smoke exposure can affect the onset and evolution of wheezing phenotypes. The first 1000 days of life, including the intrauterine period, are crucial in the organism’s physiological growth and the development of respiratory diseases. Prematurity can significantly affect lung development, risk of wheezing and obstructive respiratory disease across all ages. Breastfeeding and vitamin D supplementation may contribute to protecting the child from the development of wheezing. 

Table 1 summarises the 15 statements. An extended evidence summary is available in the Supplementary Material.

## 5. Conclusions

Based on a panel of experts and extensive updated literature, this consensus document provides insights on the pathogenesis, the risk and protective factors associated with the development and persistence of preschool wheezing. Undoubtedly, more research is needed to improve our understanding of the disease and confirm the associations between certain factors and the risk of wheezing in early life. In addition, preventive strategies must be promoted to avoid children’s exposure to risk factors that may permanently affect respiratory health. 

## Figures and Tables

**Table 1:** Statements on risk factors affecting development and persistence of preschool wheezing.

Question	Answer
**Section 1.** Pathogenesis of preschool wheezing	
Q1. What is the role of infection in the pathogenesis of preschool wheezing?	There is evidence that mainly viruses can trigger wheezing in young children. RSV and HRV are the main viruses involved in wheezing pathogenesis.
Q2. What is the role of atopy in the pathogenesis of preschool wheezing?	Recurrent multi-trigger wheezing often presents a severe clinical spectrum, can be associated with atopy more frequently than EVW and might expose the child to a higher risk of developing asthma at a later age. Aeroallergen sensitization and blood eosinophils can be used as biomarkers to identify responses to ICS in a recurrent preschool wheeze.
**Section 2.** Risk factors for wheeze development	
Q3. Does the presence of risk factors such as allergy/atopy influence the onset and the evolution of preschool wheezing?	Young children with recurrent wheezing with atopic eczema, sensitized to allergens or blood eosinophilia, are at higher risk of asthma at a later age.
Q4. Does the presence of risk factors such as previous respiratory tract infection/bronchiolitis influence the onset and evolution of preschool wheezing?	Infants with bronchiolitis represent a high-risk group for recurrent wheezing.
Q5. Does pollution influence the onset and evolution of preschool wheezing?	Traffic-related air pollution may favour wheezing, likely via a reduced response to viral infections. Both outdoor and indoor pollution can influence the respiratory health of young children from conception and birth.
Q6. Does genetics influence the onset and the evolution of preschool wheezing?	Some individuals have a genetic susceptibility and are predisposed to develop preschool wheezing at first and eventually asthma later in life. At present, little can be done to modify genetic susceptibility, but environmental exposures can be adjusted to reduce this risk and potentially work on primary asthma prevention.
Q7. Does obesity influence the onset and the evolution of preschool wheezing?	Rapid weight gain in infancy and high BMI is associated with an increased risk of wheezing in preschool age.
Q8. Do prematurity and other perinatal factors influence the onset and the evolution of preschool wheezing?	Preterm birth and low birth weight are important early life risk factors for wheezing disorders in childhood. Extremely preterm infants are at the highest risk for respiratory problems and may have lower lung function trajectories across all ages.
Q9. Does smoke exposure influence the onset and the evolution of preschool wheezing?	Maternal smoking during uterine fetal life and subsequent second and third-hand smoke exposure increase the risk of wheezing in preschool children, particularly those with a family history of allergy.
Q10. Is immunodeficiency a risk factor for the onset and the evolution of preschool wheezing?	PID must be suspected in case of persistent wheeze refractory to therapies and a history of pulmonary or systemic infections with unusual organisms. IgA deficiency can predispose the child to recurrent infections, including wheezing.
**Section 3.** Protective factors for wheeze development	
Q11. Are probiotics protective for preschool wheezing development?	Probiotic administration to reduce wheezing development is not recommended.
Q12. Is vitamin D supplementation protective for preschool wheezing development?	Vitamin D supplementation during the winter season may decrease the risk of RTIs and wheezing exacerbations.
Q13. Is breastfeeding protective for preschool wheezing development?	Maternal breastfeeding protects from preschool wheezing.
Q14. Is influenza vaccination protective for preschool wheezing development?	Influenza vaccination is recommended for its efficacy and safety in young children ≥6 months of age with wheezing.
Q15. Are non-specific immunomodulators protective for preschool wheezing development?	Prophylaxis with non-specific immunomodulators can be considered in children with recurrent EVW to reduce the number of episodes during the winter season.

## Data Availability

Not applicable.

## Supplementary Materials

The following supporting information can be downloaded at: https://www.mdpi.com/article/10.3390/jcm11216558/s1, Supplementary Material S1: PICO questions and review of the literature.

## References

[1] Martinez F.D., Wright A.L., Taussig L.M., Holberg C.J., Halonen M., Morgan W.J. (1995). Asthma and wheezing in the first six years of life. The Group Health Medical Associates. N. Engl. J. Med..

[2] Moorman J.E., Akinbami L.J., Bailey C.M., Zahran H.S., King M.E., Johnson C.A., Liu X. (2012). National Surveillance of Asthma: United States, 2001–2010. Vital Health Statistics. Ser. 3 Anal. Epidemiol. Stud..

[3] Paton J. Paediatric Asthma 2015—British Thoracic Society. Audit-Reports. https://www.brit-thoracic.org.uk.

[4] Principi N., Daleno C., Esposito S. (2014). Human rhinoviruses and severe respiratory infections: Is it possible to identify at-risk patients early?. Expert Rev. Anti Infect. Ther..

[5] Robinson P.F.M., Pattaroni C., Cook J., Gregory L., Alonso A.M., Fleming L.J., Lloyd C.M., Bush A., Marsland B.J., Saglani S. (2019). Lower airway microbiota associates with inflammatory phenotype in severe preschool wheeze. J. Allergy Clin. Immunol..

[6] Caffarelli C., Garrubba M., Greco C., Mastrorilli C., Dascola C.P. (2016). Asthma and Food Allergy in Children: Is There a Connection or Interaction?. Front. Pediatr..

[7] Deliu M., Fontanella S., Haider S., Sperrin M., Geifman N., Murray C., Simpson A., Custovic A. (2020). Longitudinal trajectories of severe wheeze exacerbations from infancy to school age and their association with early-life risk factors and late asthma outcomes. Clin. Exp. Allergy.

[8] Fainardi V., Caffarelli C., Bergamini B.M., Biserna L., Bottau P., Corinaldesi E., Dondi A., Fornaro M., Guidi B., Lombardi F. (2021). Management of Children with Acute Asthma Attack: A RAND/UCLA Appropriateness Approach. Int. J. Environ. Res. Public Health.

[9] Brozek J.L., Akl E.A., Jaeschke R., Lang D.M., Bossuyt P., Glasziou P., Helfand M., Ueffing E., Alonso-Coello P., Meerpohl J. (2009). Grading quality of evidence and strength of recommendations in clinical practice guidelines: Part 2 of 3. The GRADE approach to grading quality of evidence about diagnostic tests and strategies. Allergy.

[10] Fainardi V., Caffarelli C., Deolmi M., Skenderaj K., Meoli A., Morini R., Bergamini B.M., Bertelli L., Biserna L., Bottau P. (2022). Management of Preschool Wheezing: Guideline from the Emilia-Romagna Asthma (ERA) Study Group. J. Clin. Med..

[11] Krzysztofiak A., Chiappini E., Venturini E., Gargiullo L., Roversi M., Montagnani C., Bozzola E., Chiurchiu S., Vecchio D., Castagnola E. (2021). Italian consensus on the therapeutic management of uncomplicated acute hematogenous osteomyelitis in children. Ital. J. Pediatr..

[12] Andrews J.C., Schünemann H.J., Oxman A.D., Pottie K., Meerpohl J.J., Coello P.A., Rind D., Montori V.M., Brito J.P., Norris S. (2013). GRADE guidelines: 15. Going from evidence to recommendation-determinants of a recommendation’s direction and strength. J. Clin. Epidemiol..

[13] Lemanske R.F., Jackson D.J., Gangnon R.E., Evans M.D., Li Z., Shult P.A., Kirk C.J., Reisdorf E., Roberg K.A., Anderson E.L. (2005). Rhinovirus illnesses during infancy predict subsequent childhood wheezing. J. Allergy Clin. Immunol..

[14] Fujitsuka A., Tsukagoshi H., Arakawa M., Goto-Sugai K., Ryo A., Okayama Y., Mizuta K., Nishina A., Yoshizumi M., Kaburagi Y. (2011). A molecular epidemiological study of respiratory viruses detected in Japanese children with acute wheezing illness. BMC Infect. Dis..

[15] Takeyama A., Hashimoto K., Sato M., Sato T., Tomita Y., Maeda R., Ito M., Katayose M., Kawasaki Y., Hosoya M. (2014). Clinical and epidemiologic factors related to subsequent wheezing after virus-induced lower respiratory tract infections in hospitalized pediatric patients younger than 3 years. Eur. J. Pediatr..

[16] Leino A., Lukkarinen M., Turunen R., Vuorinen T., Söderlund-Venermo M., Vahlberg T., Camargo C.A., Bochkov Y.A., Gern J.E., Jartti T. (2019). Pulmonary function and bronchial reactivity 4 years after the first virus-induced wheezing. Allergy.

[17] Stern D.A., Guerra S., Halonen M., Wright A.L., Martinez F.D. (2007). Low IFN-gamma production in the first year of life as a predictor of wheeze during childhood. J. Allergy Clin. Immunol..

[18] Chawes B.L., Poorisrisak P., Johnston S.L., Bisgaard H. (2012). Neonatal bronchial hyperresponsiveness precedes acute severe viral bronchiolitis in infants. J. Allergy Clin. Immunol..

[19] Heymann P.W., Carper H.T., Murphy D.D., Platts-Mills T.A., Patrie J., McLaughlin A.P., Erwin E.A., Shaker M.S., Hellems M., Peerzada J. (2004). Viral infections in relation to age, atopy, and season of admission among children hospitalized for wheezing. J. Allergy Clin. Immunol..

[20] De Winter J.J., Bont L., Wilbrink B., van der Ent C.K., Smit H.A., Houben M.L. (2015). Rhinovirus wheezing illness in infancy is associated with medically attended third year wheezing in low risk infants: Results of a healthy birth cohort study. Immun. Inflamm. Dis..

[21] Van der Gugten A.C., van der Zalm M.M., Uiterwaal C.S., Wilbrink B., Rossen J.W., van der Ent C.K. (2013). Human rhinovirus and wheezing: Short and long-term associations in children. Pediatr. Infect. Dis. J..

[22] Liu L., Pan Y., Zhu Y., Song Y., Su X., Yang L., Li M. (2017). Association between rhinovirus wheezing illness and the development of childhood asthma: A meta-analysis. BMJ Open.

[23] Midulla F., Pierangeli A., Cangiano G., Bonci E., Salvadei S., Scagnolari C., Moretti C., Antonelli G., Ferro V., Papoff P. (2012). Rhinovirus bronchiolitis and recurrent wheezing: 1-year follow-up. Eur. Respir. J..

[24] O’Callaghan-Gordo C., Bassat Q., Díez-Padrisa N., Morais L., Machevo S., Nhampossa T., Quintó L., Alonso P.L., Roca A. (2013). Lower respiratory tract infections associated with rhinovirus during infancy and increased risk of wheezing during childhood. A cohort study. PLoS ONE.

[25] Midulla F., Nicolai A., Ferrara M., Gentile F., Pierangeli A., Bonci E., Scagnolari C., Moretti C., Antonelli G., Papoff P. (2014). Recurrent wheezing 36 months after bronchiolitis is associated with rhinovirus infections and blood eosinophilia. Acta Paediatr..

[26] Rossi G.A., Colin A.A. (2015). Infantile respiratory syncytial virus and human rhinovirus infections: Respective role in inception and persistence of wheezing. Eur. Respir. J..

[27] Feldman A.S., He Y., Moore M.L., Hershenson M.B., Hartert T.V. (2015). Toward primary prevention of asthma. Reviewing the evidence for early-life respiratory viral infections as modifiable risk factors to prevent childhood asthma. Am. J. Respir. Crit. Care Med..

[28] Lee W.M., Lemanske R.F., Evans M.D., Vang F., Pappas T., Gangnon R., Jackson D.J., Gern J.E. (2012). Human rhinovirus species and season of infection determine illness severity. Am. J. Respir. Crit. Care Med..

[29] Skevaki C.L., Psarras S., Volonaki E., Pratsinis H., Spyridaki I.S., Gaga M., Georgiou V., Vittorakis S., Telcian A.G., Maggina P. (2012). Rhinovirus-induced basic fibroblast growth factor release mediates airway remodeling features. Clin. Transl. Allergy.

[30] Legg J.P., Hussain I.R., Warner J.A., Johnston S.L., Warner J.O. (2003). Type 1 and type 2 cytokine imbalance in acute respiratory syncytial virus bronchiolitis. Am. J. Respir. Crit. Care Med..

[31] Esposito S., Daleno C., Tagliabue C., Scala A., Tenconi R., Borzani I., Fossali E., Pelucchi C., Piralla A., Principi N. (2012). Impact of rhinoviruses on pediatric community-acquired pneumonia. Eur. J. Clin. Microbiol. Infect. Dis..

[32] Ballarini S., Rossi G.A., Principi N., Esposito S. (2021). Dysbiosis in Pediatrics Is Associated with Respiratory Infections: Is There a Place for Bacterial-Derived Products?. Microorganisms.

[33] Schwerk N., Brinkmann F., Soudah B., Kabesch M., Hansen G. (2011). Wheeze in preschool age is associated with pulmonary bacterial infection and resolves after antibiotic therapy. PLoS ONE.

[34] Esposito S., Ballarini S., Argentiero A., Ruggiero L., Rossi G.A., Principi N. (2022). Microbiota profiles in pre-school children with respiratory infections: Modifications induced by the oral bacterial lysate OM-85. Front. Cell. Infect. Microbiol..

[35] De Schutter I., Dreesman A., Soetens O., De Waele M., Crokaert F., Verhaegen J., Piérard D., Malfroot A. (2012). In young children, persistent wheezing is associated with bronchial bacterial infection: A retrospective analysis. BMC Pediatr..

[36] Teo S.M., Mok D., Pham K., Kusel M., Serralha M., Troy N., Holt B.J., Hales B.J., Walker M.L., Hollams E. (2015). The infant nasopharyngeal microbiome impacts severity of lower respiratory infection and risk of asthma development. Cell Host Microbe.

[37] Robinson P.F.M., Fontanella S., Ananth S., Alonso A.M., Cook J., Kaya-de Vries D., Polo Silveira L., Gregory L., Lloyd C., Fleming L. (2021). Recurrent Severe Preschool Wheeze: From Prespecified Diagnostic Labels to Underlying Endotypes. Am. J. Respir. Crit. Care Med..

[38] Bacharier L.B., Guilbert T.W., Mauger D.T., Boehmer S., Beigelman A., Fitzpatrick A.M., Jackson D.J., Baxi S.N., Benson M., Burnham C.D. (2015). Early Administration of Azithromycin and Prevention of Severe Lower Respiratory Tract Illnesses in Preschool Children with a History of Such Illnesses: A Randomized Clinical Trial. JAMA.

[39] Stokholm J., Chawes B.L., Vissing N.H., Bjarnadóttir E., Pedersen T.M., Vinding R.K., Schoos A.M., Wolsk H.M., Thorsteinsdóttir S., Hallas H.W. (2016). Azithromycin for episodes with asthma-like symptoms in young children aged 1–3 years: A randomised, double-blind, placebo-controlled trial. Lancet Respir. Med..

[40] Esposito S., Blasi F., Arosio C., Fioravanti L., Fagetti L., Droghetti R., Tarsia P., Allegra L., Principi N. (2000). Importance of acute Mycoplasma pneumoniae and Chlamydia pneumoniae infections in children with wheezing. Eur. Respir. J..

[41] Esposito S., Droghetti R., Bosis S., Claut L., Marchisio P., Principi N. (2002). Cytokine secretion in children with acute Mycoplasma pneumoniae infection and wheeze. Pediatr. Pulmonol..

[42] Liu C., Makrinioti H., Saglani S., Bowman M., Lin L.L., Camargo C.A., Hasegawa K., Zhu Z. (2022). Microbial dysbiosis and childhood asthma development: Integrated role of the airway and gut microbiome, environmental exposures, and host metabolic and immune response. Front. Immunol..

[43] Zhu Z., Camargo C.A., Raita Y., Freishtat R.J., Fujiogi M., Hahn A., Mansbacj J.M., Spergel J.M., Pérez-Losada M., Hasegawa K. (2022). Nasopharyngeal airway dual-transcriptome of infants with severe bronchiolitis and risk of childhood asthma: A multicenter prospective study. J. Allergy Clin. Immunol..

[44] Bosch A.A.T.M., de Steenhuijsen Piters W.A.A., van Houten M.A., Chu M.L.J.N., Biesbroek G., Kool J., Pernet P., de Groot P.C.M., Eijkemans M.J.C., Keijser B.J.F. (2017). Maturation of the Infant Respiratory Microbiota, Environmental Drivers, and Health Consequences. A Prospective Cohort Study. Am. J. Respir. Crit. Care Med..

[45] Thavagnanam S., Williamson G., Ennis M., Heaney L.G., Shields M.D. (2010). Does airway allergic inflammation pre-exist before late onset wheeze in children?. Pediatr. Allergy Immunol..

[46] Lezmi G., Deschildre A., Abou Taam R., Fayon M., Blanchon S., Troussier F., Mallinger P., Mahut B., Gosset P., de Blic J. (2018). Remodelling and inflammation in preschoolers with severe recurrent wheeze and asthma outcome at school age. Clin. Exp. Allergy.

[47] Saglani S., Payne D.N., Zhu J., Wang Z., Nicholson A.G., Bush A., Jeffery P.K. (2007). Early detection of airway wall remodeling and eosinophilic inflammation in preschool wheezers. Am. J. Respir. Crit. Care Med..

[48] Ferreira M.A., Vonk J.W., Baurecht H., Marenholz I., Tian C., Hoffman J.D., Helmer Q., Tillander A., Ullemar V., van Dongen J. (2017). Shared genetic origin of asthma, hay fever and eczema elucidates allergic disease biology. Nat. Genet..

[49] Zhu Z., Lee P.H., Chaffin M.D., Chung W., Loh P.O., Lu Q., Christiani D.C., Liang L. (2018). A genome-wide cross-trait analysis from UK Biobank highlights the shared genetic architecture of asthma and allergic diseases. Nat. Genet..

[50] Guiddir T., Saint-Pierre P., Purenne-Denis E., Lambert N., Laoudi Y., Couderc R., Gouvis-Echraghi R., Amat F., Just J. (2017). Neutrophilic Steroid-Refractory Recurrent Wheeze and Eosinophilic Steroid-Refractory Asthma in Children. J. Allergy Clin. Immunol. Pract..

[51] Turato G., Barbato A., Baraldo S., Zanin M.E., Bazzan E., Lokar-Oliani K., Calabrese F., Panizzolo C., Snijders D., Maestrelli P. (2008). Nonatopic children with multitrigger wheezing have airway pathology comparable to atopic asthma. Am. J. Respir. Crit. Care Med..

[52] Just J., Nicoloyanis N., Chauvin M., Pribil C., Grimfeld A., Duru G. (2008). Lack of eosinophilia can predict remission in wheezy infants?. Clin. Exp. Allergy.

[53] Fitzpatrick A.M., Jackson D.J., Mauger D.T., Boehmer S.J., Phipatanakul W., Sheehan W.J., Moy J.N., Paul I.M., Bacharier L.B., Cabana M.D. (2016). Individualized therapy for persistent asthma in young children. J. Allergy Clin. Immunol..

[54] Esposito S., Principi N. (2014). Pharmacological approach to wheezing in preschool children. Expert Opin. Pharmacother..

[55] Shinohara M., Wakiguchi H., Saito H., Matsumoto K. (2008). Presence of eosinophils in nasal secretion during acute respiratory tract infection in young children predicts subsequent wheezing within two months. Allergol. Int..

[56] Guo Y., Zou Y., Zhai J., Li J., Liu J., Ma C., Jin X., Zhao L. (2019). Phenotypes of the inflammatory cells in the induced sputum from young children or infants with recurrent wheezing. Pediatr. Res..

[57] Ater D., Bar B.E., Fireman N., Fireman E., Shai H., Tasher D., Dalal I., Mandelberg A. (2014). Asthma-predictive-index, bronchial-challenge, sputum eosinophils in acutely wheezing preschoolers. Pediatr. Pulmonol..

[58] De Sousa R.B., Medeiros D., Sarinho E., Rizzo J.Â., Silva A.R., Bianca A.C. (2016). Risk factors for recurrent wheezing in infants: A case-control study. Rev. Saude Publica.

[59] Quah P.L., Huang C.H., Shek L.P., Chua K.Y., Lee B.W., Kuo I.C. (2014). Hyper-responsive T-cell cytokine profile in association with development of early childhood wheeze but not eczema at 2 years. Asian Pac. J. Allergy Immunol..

[60] Sly P.D., Boner A.L., Björksten B., Bush A., Custovic A., Eigenmann P.A., Gern J.E., Gerritsen J., Hamelmann E., Helms P.J. (2008). Early identification of atopy in the prediction of persistent asthma in children. Lancet.

[61] Lloyd C.M., Saglani S. (2017). Development of allergic immunity in early life. Immunol. Rev..

[62] Lynch S.V., Wood R.A., Boushey H., Bacharier L.B., Bloomberg G.R., Kattan M., O’Connor G.T., Sandel M.T., Calatroni A., Matsui E. (2014). Effects of early-life exposure to allergens and bacteria on recurrent wheeze and atopy in urban children. J. Allergy Clin. Immunol..

[63] Henderson J., Granell R., Heron J., Sherriff A., Simpson A., Woodcock A., Strachan D.P., Shaheen S.O., Sterne J.A. (2008). Associations of wheezing phenotypes in the first 6 years of life with atopy, lung function and airway responsiveness in mid-childhood. Thorax.

[64] Just J., Belfar S., Wanin S., Pribil C., Grimfeld A., Duru G. (2010). Impact of innate and environmental factors on wheezing persistence during childhood. J. Asthma.

[65] Arshad S.H., Karmaus W., Raza A., Kurukulaaratchy R.J., Matthews S.M., Holloway J.W., Sadeghnejad A., Zhang H., Roberts G., Ewart S.L. (2012). The effect of parental allergy on childhood allergic diseases depends on the sex of the child. J. Allergy Clin. Immunol..

[66] Schmidt F., Hose A.J., Mueller-Rompa S., Brick T., Hämäläinen A.M., Peet A., Tillmann V., Niemelä O., Siljander H., Knip M. (2019). Development of atopic sensitization in Finnish and Estonian children: A latent class analysis in a multicenter cohort. J. Allergy Clin. Immunol..

[67] Donohue K.M., Al-alem U., Perzanowski M.S., Chew G.L., Johnson A., Divjan A., Kelvin E.A., Hoepner L.A., Perera F.P., Miller R.L. (2008). Anti-cockroach and anti-mouse IgE are associated with early wheeze and atopy in an inner-city birth cohort. J. Allergy Clin. Immunol..

[68] Yu C.L., Huang W.T., Wang C.M. (2019). Treatment of allergic rhinitis reduces acute asthma exacerbation risk among asthmatic children aged 2–18 years. J. Microbiol. Immunol. Infect..

[69] Von Kobyletzki L.B., Bornehag C.G., Hasselgren M., Larsson M., Lindström C.B., Svensson Å. (2012). Eczema in early childhood is strongly associated with the development of asthma and rhinitis in a prospective cohort. BMC Dermatol..

[70] Saunes M., Øien T., Dotterud C.K., Romundstad P.R., Storrø O., Holmen T.L., Johnsen R. (2012). Early eczema and the risk of childhood asthma: A prospective, population-based study. BMC Pediatr..

[71] Chiu C.Y., Yang C.H., Su K.W., Tsai M.H., Hua M.C., Liao S.L., Lai S.H., Chen L.C., Yeh K.W., Huang J.L. (2020). Early-onset eczema is associated with increased milk sensitization and risk of rhinitis and asthma in early childhood. J. Microbiol. Immunol. Infect..

[72] Ekbäck M., Tedner M., Devenney I., Oldaeus G., Norrman G., Strömberg L., Fälth-Magnusson K. (2014). Severe eczema in infancy can predict asthma development. A prospective study to the age of 10 years. PLoS ONE.

[73] Boersma N.A., Meijneke R.W.H., Kelder J.C., van der Ent C.K., Balemans W.A.F. (2017). Sensitization predicts asthma development among wheezing toddlers in secondary healthcare. Pediatr. Pulmonol..

[74] Vermeulen E.M., Koplin J.J., Dharmage S.C., Gurrin L.C., Peters R.L., McWilliam V., Ponsonby A.L., Dwyer T., Lowe A.J., Tang M.L.K. (2018). Food Allergy Is an Important Risk Factor for Childhood Asthma, Irrespective of Whether It Resolves. J. Allergy Clin. Immunol. Pract..

[75] Illi S., von Mutius E., Lau S., Nickel R., Niggemann B., Sommerfeld C., Wahn U., Multicenter Allergy Study Group (2001). The pattern of atopic sensitization is associated with the development of asthma in childhood. J. Allergy Clin. Immunol..

[76] Illi S., von Mutius E., Lau S., Niggemann B., Grüber C., Wahn U., Multicentre Allergy Study (MAS) Group (2006). Perennial allergen sensitisation early in life and chronic asthma in children: A birth cohort study. Lancet.

[77] Kenmoe S., Bowo-Ngandji A., Kengne-Nde C., Ebogo-Belobo J.T., Mbaga D.S., Mahamat G., Demeni Emoh C.P., Njouom R. (2021). Association between early viral LRTI and subsequent wheezing development, a meta-analysis and sensitivity analyses for studies comparable for confounding factors. PLoS ONE.

[78] Kovesi T.A., Cao Z., Osborne G., Egeland G.M. (2011). Severe early lower respiratory tract infection is associated with subsequent respiratory morbidity in preschool Inuit children in Nunavut, Canada. J. Asthma.

[79] Lin H.W., Lin S.C. (2012). Environmental factors association between asthma and acute bronchiolitis in young children—A perspective cohort study. Eur. J. Pediatr..

[80] Mikalsen I.B., Halvorsen T., Eide G.E., Øymar K. (2013). Severe bronchiolitis in infancy: Can asthma in adolescence be predicted?. Pediatr. Pulmonol..

[81] Jeng M.J., Lee Y.S., Tsao P.C., Yang C.F., Soong W.J. (2015). A longitudinal study on early hospitalized airway infections and subsequent childhood asthma. PLoS ONE.

[82] Nicolai A., Frassanito A., Nenna R., Cangiano G., Petrarca L., Papoff P., Pierangeli A., Scagnolari C., Moretti C., Midulla F. (2017). Risk Factors for Virus-Induced Acute Respiratory Tract Infections in Children Younger Than 3 Years and Recurrent Wheezing at 36 Months Follow-Up After Discharge. Pediatr. Infect. Dis. J..

[83] Díez-Domingo J., Pérez-Yarza E.G., Melero J.A., Sánchez-Luna M., Aguilar M.D., Blasco A.J., Alfaro N., Lázaro P. (2014). Social, economic, and health impact of the respiratory syncytial virus: A systematic search. BMC Infect. Dis..

[84] Rinawi F., Kassis I., Tamir R., Kugelman A., Srugo I., Miron D. (2017). Bronchiolitis in young infants: Is it a risk factor for recurrent wheezing in childhood?. World J. Pediatr..

[85] Skirrow H., Wincott T., Cecil E., Bottle A., Costelloe C., Saxena S. (2019). Preschool respiratory hospital admissions following infant bronchiolitis: A birth cohort study. Arch. Dis. Child..

[86] Dumas O., Hasegawa K., Mansbach J.M., Sullivan A.F., Piedra P.A., Camargo C.A. (2019). Severe bronchiolitis profiles and risk of recurrent wheeze by age 3 years. J. Allergy Clin. Immunol..

[87] Bacharier L.B., Cohen R., Schweiger T., Yin-Declue H., Christie C., Zheng J., Schechtman K.B., Strunk R.C., Castro M. (2012). Determinants of asthma after severe respiratory syncytial virus bronchiolitis. J. Allergy Clin. Immunol..

[88] Mansbach J.M., Hasegawa K., Geller R.J., Espinola J.A., Sullivan A.F., Camargo C.A., MARC-35 Investigators (2020). Bronchiolitis severity is related to recurrent wheezing by age 3 years in a prospective, multicenter cohort. Pediatr. Res..

[89] Régnier S.A., Huels J. (2013). Association between respiratory syncytial virus hospitalizations in infants and respiratory sequelae: Systematic review and meta-analysis. Pediatr. Infect. Dis. J..

[90] Coutts J., Fullarton J., Morris C., Grubb E., Buchan S., Rodgers-Gray B., Thwaites R. (2020). Association between respiratory syncytial virus hospitalization in infancy and childhood asthma. Pediatr. Pulmonol..

[91] Nguyen-Van-Tam J., Wyffels V., Smulders M., Mazumder D., Tyagi R., Gupta N., Gavart S., Fleischhackl R. (2020). Cumulative incidence of post-infection asthma or wheezing among young children clinically diagnosed with respiratory syncytial virus infection in the United States: A retrospective database analysis. Influenza Other Respir. Viruses.

[92] Shi T., Ooi Y., Zaw E.M., Utjesanovic N., Campbell H., Cunningham S., Bont L., Nair H., RESCEU Investigators (2020). Association Between Respiratory Syncytial Virus-Associated Acute Lower Respiratory Infection in Early Life and Recurrent Wheeze and Asthma in Later Childhood. J. Infect. Dis..

[93] Romero J.R., Stewart D.L., Buysman E.K., Fernandes A.W., Jafri H.S., Mahadevia P.J. (2010). Serious early childhood wheezing after respiratory syncytial virus lower respiratory tract illness in preterm infants. Clin. Ther..

[94] Escobar G.J., Masaquel A.S., Li S.X., Walsh E.M., Kipnis P. (2013). Persistent recurring wheezing in the fifth year of life after laboratory-confirmed, medically attended respiratory syncytial virus infection in infancy. BMC Pediatr..

[95] Tan S., Szatkowski L., Moreton W., Fiaschi L., McKeever T., Gibson J., Sharkey D. (2020). Early childhood respiratory morbidity and antibiotic use in ex-preterm infants: A primary care population-based cohort study. Eur. Respir. J..

[96] Van Wijhe M., Johannesen C.K., Simonsen L., Jørgensen I.M., Fischer T.K., RESCEU Investigators (2022). A retrospective cohort study on infant respiratory tract infection hospitalizations and recurrent wheeze and asthma risk: Impact of respiratory syncytial virus. J. Infect. Dis..

[97] Brunwasser S.M., Snyder B.M., Driscoll A.J., Fell D.B., Savitz D.A., Feikin D.R., Skidmore B., Bhat N., Bont L.J., Dupont W.D. (2020). Assessing the strength of evidence for a causal effect of respiratory syncytial virus lower respiratory tract infections on subsequent wheezing illness: A systematic review and meta-analysis. Lancet Respir. Med..

[98] Korppi M., Kotaniemi-Syrjänen A., Waris M., Vainionpää R., Reijonen T.M. (2004). Rhinovirus-associated wheezing in infancy: Comparison with respiratory syncytial virus bronchiolitis. Pediatr. Infect. Dis. J..

[99] Jartti T., Gern J.E. (2011). Rhinovirus-associated wheeze during infancy and asthma development. Curr. Respir. Med. Rev..

[100] Sun H., Sun Q., Jiang W., Chen Z., Huang L., Wang M., Ji W., Shao X., Yan Y. (2016). Prevalence of rhinovirus in wheezing children: A comparison with respiratory syncytial virus wheezing. Braz. J. Infect. Dis..

[101] Cox D.W., Bizzintino J., Ferrari G., Khoo S.K., Zhang G., Whelan S., Lee W.M., Bochkov Y.A., Geelhoed G.C., Goldblatt J. (2013). Human rhinovirus species C infection in young children with acute wheeze is associated with increased acute respiratory hospital admissions. Am. J. Respir. Crit. Care Med..

[102] Jartti T., Kuusipalo H., Vuorinen T., Söderlund-Venermo M., Allander T., Waris M., Hartiala J., Ruuskanen O. (2010). Allergic sensitization is associated with rhinovirus-, but not other virus-, induced wheezing in children. Pediatr. Allergy Immunol..

[103] Hasegawa K., Mansbach J.M., Bochkov Y.A., Gern J.E., Piedra P.A., Bauer C.S., Teach S.J., Wu S., Sullivan A.F., Camargo C.A. (2019). Association of Rhinovirus C Bronchiolitis and Immunoglobulin E Sensitization During Infancy with Development of Recurrent Wheeze. JAMA Pediatr..

[104] Reimerink J., Stelma F., Rockx B., Brouwer D., Stobberingh E., van Ree R., Dompeling E., Mommers M., Thijs C., Koopmans M. (2009). Early-life rotavirus and norovirus infections in relation to development of atopic manifestation in infants. Clin. Exp. Allergy.

[105] Li S., Williams G., Jalaludin B., Baker P. (2012). Panel studies of air pollution on children’s lung function and respiratory symptoms: A literature review. J. Asthma.

[106] Nordling E., Berglind N., Melén E., Emenius G., Hallberg J., Nyberg F., Pershagen G., Svartengren M., Wickman M., Bellander T. (2008). Traffic-related air pollution and childhood respiratory symptoms, function and allergies. Epidemiology.

[107] Freid R.D., Qi Y.S., Espinola J.A., Cash R.E., Aryan Z., Sullivan A.F., Camargo C.A. (2021). Proximity to Major Roads and Risks of Childhood Recurrent Wheeze and Asthma in a Severe Bronchiolitis Cohort. Int. J. Environ. Res. Public Health.

[108] Brunst K.J., Ryan P.H., Brokamp C., Bernstein D., Reponen T., Lockey J., Hershey G.K.K., Levin L., Grinshpun S.A., LeMasters G. (2015). Timing and Duration of Traffic-related Air Pollution Exposure and the Risk for Childhood Wheeze and Asthma. Am. J. Respir. Crit. Care Med..

[109] Hasunuma H., Sato T., Iwata T., Kohno Y., Nitta H., Odajima H., Ohara T., Omori T., Ono M., Yamazaki S. (2016). Association between traffic-related air pollution and asthma in preschool children in a national Japanese nested case-control study. BMJ Open.

[110] Esposito S., Galeone C., Lelii M., Longhi B., Ascolese B., Senatore L., Prada E., Montinaro V., Malerba S., Patria M.F. (2014). Impact of air pollution on respiratory diseases in children with recurrent wheezing or asthma. BMC Pulm. Med..

[111] Bonato M., Gallo E., Turrin M., Bazzan E., Baraldi F., Saetta M., Gregori D., Papi A., Contoli M., Baraldo S. (2021). Air Pollution Exposure Impairs Airway Epithelium IFN-β Expression in Pre-School Children. Front. Immunol..

[112] Bonato M., Gallo E., Bazzan E., Marson G., Zagolin L., Cosio M.G., Barbato A., Saetta M., Gregori D., Baraldo S. (2021). Air Pollution Relates to Airway Pathology in Children with Wheezing. Ann. Am. Thorac. Soc..

[113] Norbäck D., Lu C., Zhang Y., Li B., Zhao Z., Huang C., Zhang X., Qian H., Sun Y., Sundell J. (2019). Onset and remission of childhood wheeze and rhinitis across China—Associations with early life indoor and outdoor air pollution. Environ. Int..

[114] Holst G.J., Pedersen C.B., Thygesen M., Brandt J., Geels C., Bønløkke J.H., Sigsgaard T. (2020). Air pollution and family related determinants of asthma onset and persistent wheezing in children: Nationwide case-control study. BMJ.

[115] Zhang Y., Wei J., Shi Y., Quan C., Ho H.C., Song Y., Zhang L. (2021). Early-life exposure to submicron particulate air pollution in relation to asthma development in Chinese preschool children. J. Allergy Clin. Immunol..

[116] Brand A., McLean K.E., Henderson S.B., Fournier M., Liu L., Kosatsky T., Smargiassi A. (2016). Respiratory hospital admissions in young children living near metal smelters, pulp mills and oil refineries in two Canadian provinces. Environ. Int..

[117] Makrinioti H., Camargo C.A., Zhu Z., Freishtat R.J., Hasegawa K. (2022). Air pollution, bronchiolitis, and asthma: The role of nasal microRNAs. Lancet Respir. Med..

[118] Ji H., Myers J.M.B., Brandt E.B., Brokamp C., Ryan P.H., Hershey G.K.K. (2016). Air pollution, epigenetics, and asthma. Allergy Asthma Clin. Immunol..

[119] Sharma S., Yang I.V., Schwartz D.A. (2022). Epigenetic regulation of immune function in asthma. J. Allergy Clin. Immunol..

[120] Cai J., Li B., Yu W., Wang H., Du C., Zhang Y., Huang C., Zhao Z., Deng Q., Yang X. (2019). Household dampness-related exposures in relation to childhood asthma and rhinitis in China: A multicentre observational study. Environ. Int..

[121] Saijo Y., Yoshioka E., Sato Y., Azuma H., Tanahashi Y., Ito Y., Kobayashi S., Minatoya M., Bamai Y.A., Yamazaki K. (2022). Relations of mold, stove, and fragrance products on childhood wheezing and asthma: A prospective cohort study from the Japan Environment and Children’s Study. Indoor Air.

[122] Mikeš O., Vrbová M., Klánová J., Čupr P., Švancara J., Pikhart H. (2019). Early-life exposure to household chemicals and wheezing in children. Sci. Total Environ..

[123] Parks J., McCandless L., Dharma C., Brook J., Turvey S.E., Mandhane P., Becker A.B., Kozyrskyj A.L., Azad M.B., Moraes T.J. (2020). Association of use of cleaning products with respiratory health in a Canadian birth cohort. CMAJ.

[124] Lin S.C., Lin H.W. (2015). Urbanization factors associated with childhood asthma and prematurity: A population-based analysis aged from 0 to 5 years in Taiwan by using Cox regression within a hospital cluster model. J. Asthma.

[125] Gascon M., Casas M., Morales E., Valvi D., Ballesteros-Gómez A., Luque N., Rubio S., Monfort N., Ventura R., Martínez D. (2015). Prenatal exposure to bisphenol A and phthalates and childhood respiratory tract infections and allergy. J. Allergy Clin. Immunol..

[126] Bisgaard H., Bønnelykke K., Sleiman P.M., Brasholt M., Chawes B., Kreiner-Møller E., Stage M., Kim C., Tavendale R., Baty F. (2009). Chromosome 17q21 gene variants are associated with asthma and exacerbations but not atopy in early childhood. Am. J. Respir. Crit. Care Med..

[127] Halapi E., Gudbjartsson D.F., Jonsdottir G.M., Bjornsdottir U.S., Thorleifsson G., Helgadottir H., Williams C., Koppelman G.H., Heinzmann A., Boezen H.M. (2010). A sequence variant on 17q21 is associated with age at onset and severity of asthma. Eur. J. Hum. Genet..

[128] Koponen P., Nuolivirta K., Virta M., Helminen M., Hurme M., Korppi M. (2014). Polymorphism of the rs1800896 IL10 promoter gene protects children from post-bronchiolitis asthma. Pediatr. Pulmonol..

[129] Amat F., Louha M., Benet M., Guiddir T., Bourgoin-Heck M., Saint-Pierre P., Paluel-Marmont C., Fontaine C., Lambert N., Couderc R. (2017). The IL-4 rs2070874 polymorphism may be associated with the severity of recurrent viral-induced wheeze. Pediatr. Pulmonol..

[130] Korppi M., Teräsjärvi J., Lauhkonen E., Törmänen S., He Q., Nuolivirta K. (2021). Interleukin-1 receptor-associated kinase-4 gene variation may increase post-bronchiolitis asthma risk. Acta Paediatr..

[131] Koponen P., Vuononvirta J., Nuolivirta K., Helminen M., He Q., Korppi M. (2014). The association of genetic variants in toll-like receptor 2 subfamily with allergy and asthma after hospitalization for bronchiolitis in infancy. Pediatr. Infect. Dis. J..

[132] Törmänen S., Korppi M., Teräsjärvi J., Vuononvirta J., Koponen P., Helminen M., He Q., Nuolivirta K. (2017). Polymorphism in the gene encoding toll-like receptor 10 may be associated with asthma after bronchiolitis. Sci. Rep..

[133] Klaassen E.M., van de Kant K.D., Jöbsis Q., Penders J., van Schooten F.J., Quaak M., den Hartog G.J., Koppelman G.H., van Schayck C.P., van Eys G. (2014). Integrative genomic analysis identifies a role for intercellular adhesion molecule 1 in childhood asthma. Pediatr. Allergy Immunol..

[134] Marenholz I., Kerscher T., Bauerfeind A., Esparza-Gordillo J., Nickel R., Keil T., Lau S., Rohde K., Wahn U., Lee Y.A. (2009). An interaction between filaggrin mutations and early food sensitization improves the prediction of childhood asthma. J. Allergy Clin. Immunol..

[135] Dixon A.E., Peters U. (2018). The effect of obesity on lung function. Expert Rev. Respir. Med..

[136] Lowe J., Kotecha S.J., Watkins W.J., Kotecha S. (2018). Effect of fetal and infant growth on respiratory symptoms in preterm-born children. Pediatr. Pulmonol..

[137] Kotecha S.J., Lowe J., Granell R., Watkins W.J., Henderson A.J., Kotecha S. (2020). The effect of catch-up growth in the first year of life on later wheezing phenotypes. Eur. Respir. J..

[138] Mensink-Bout S.M., Santos S., van Meel E.R., Oei E.H.G., de Jongste J.C., Jaddoe V.W.V., Duijts L. (2020). General and Organ Fat Assessed by Magnetic Resonance Imaging and Respiratory Outcomes in Childhood. Am. J. Respir. Crit. Care Med..

[139] Sonnenschein-van der Voort A.M., Jaddoe V.W., Raat H., Moll H.A., Hofman A., de Jongste J.C., Duijts L. (2012). Fetal and infant growth and asthma symptoms in preschool children: The Generation R Study. Am. J. Respir. Crit. Care Med..

[140] Pike K.C., Crozier S.R., Lucas J.S., Inskip H.M., Robinson S., Roberts G., Godfrey K.M., Southampton Women’s Survey Study Group (2010). Patterns of fetal and infant growth are related to atopy and wheezing disorders at age 3 years. Thorax.

[141] Zhang Z., Lai H.J., Roberg K.A., Gangnon R.E., Evans M.D., Anderson E.L., Pappas T.E., Dasilva D.F., Tisler C.J., Salazar L.P. (2010). Early childhood weight status in relation to asthma development in high-risk children. J. Allergy Clin. Immunol..

[142] Murray C.S., Canoy D., Buchan I., Woodcock A., Simpson A., Custovic A. (2011). Body mass index in young children and allergic disease: Gender differences in a longitudinal study. Clin. Exp. Allergy.

[143] Rzehak P., Wijga A.H., Keil T., Eller E., Bindslev-Jensen C., Smit H.A., Weyler J., Dom S., Sunyer J., Mendez M. (2013). Body mass index trajectory classes and incident asthma in childhood: Results from 8 European Birth Cohorts—A Global Allergy and Asthma European Network initiative. J. Allergy Clin. Immunol..

[144] Dai R., Miliku K., Gaddipati S., Choi J., Ambalavanan A., Tran M.M., Reyna M., Sbihi H., Lou W., Parvulescu P. (2022). Wheeze trajectories: Determinants and outcomes in the CHILD Cohort Study. J. Allergy Clin. Immunol..

[145] Crump C. (2020). Preterm birth and mortality in adulthood: A systematic review. J. Perinatol..

[146] Abe K., Shapiro-Mendoza C.K., Hall L.R., Satten G.A. (2010). Late preterm birth and risk of developing asthma. J. Pediatr..

[147] Boyle E.M., Poulsen G., Field D.J., Kurinczuk J.J., Wolke D., Alfirevic Z., Quigley M.A. (2012). Effects of gestational age at birth on health outcomes at 3 and 5 years of age: Population based cohort study. BMJ.

[148] Been J.V., Lugtenberg M.J., Smets E., van Schayck C.P., Kramer B.W., Mommers M., Sheikh A. (2014). Preterm birth and childhood wheezing disorders: A systematic review and meta-analysis. PLoS Med..

[149] Voge G.A., Katusic S.K., Qin R., Juhn Y.J. (2015). Risk of Asthma in Late Preterm Infants: A Propensity Score Approach. J. Allergy Clin. Immunol. Pract..

[150] Leps C., Carson C., Quigley M.A. (2018). Gestational age at birth and wheezing trajectories at 3–11 years. Arch. Dis. Child..

[151] Pike K.C., Lucas J.S. (2015). Respiratory consequences of late preterm birth. Paediatr. Respir. Rev..

[152] Simões M.C.R.D.S., Inoue Y., Matsunaga N.Y., Carvalho M.R.V., Ribeiro G.L.T., Morais E.O., Ribeiro M.A.G.O., Morcillo A.M., Ribeiro J.D., Toro A.A.D.C. (2019). Recurrent wheezing in preterm infants: Prevalence and risk factors. J. Pediatr..

[153] Algert C.S., Bowen J.R., Lain S.L., Allen H.D., Vivian-Taylor J.M., Roberts C.L. (2011). Pregnancy exposures and risk of childhood asthma admission in a population birth cohort. Pediatr. Allergy Immunol..

[154] Kumar R., Yu Y., Story R.E., Pongracic J.A., Gupta R., Pearson C., Ortiz K., Bauchner H.C., Wang X. (2008). Prematurity, chorioamnionitis, and the development of recurrent wheezing: A prospective birth cohort study. J. Allergy Clin. Immunol..

[155] Yamamoto-Hanada K., Pak K., Saito-Abe M., Sato M., Ohya Y., Japan Environment and Children’s Study (JECS) Group (2021). Better maternal quality of life in pregnancy yields better offspring respiratory outcomes: A birth cohort. Ann. Allergy Asthma Immunol..

[156] Liu X., Olsen J., Agerbo E., Yuan W., Sigsgaard T., Li J. (2015). Prenatal stress and childhood asthma in the offspring: Role of age at onset. Eur. J. Public Health.

[157] Smejda K., Polanska K., Merecz-Kot D., Krol A., Hanke W., Jerzynska J., Stelmach W., Majak P., Stelmach I. (2018). Maternal Stress During Pregnancy and Allergic Diseases in Children During the First Year of Life. Respir. Care.

[158] Stick S.M., Burton P.R., Gurrin L., Sly P.D., LeSouëf P.N. (1996). Effects of maternal smoking during pregnancy and a family history of asthma on respiratory function in newborn infants. Lancet.

[159] Lin J., Yuan S., Dong B., Zhang J., Zhang L., Wu J., Chen J., Tang M., Zhang B., Wang H. (2021). The Associations of Caesarean Delivery with Risk of Wheezing Diseases and Changes of T Cells in Children. Front. Immunol..

[160] Huang L., Chen Q., Zhao Y., Wang W., Fang F., Bao Y. (2015). Is elective cesarean section associated with a higher risk of asthma? A meta-analysis. J. Asthma.

[161] Abdullah K., Zhu J., Gershon A., Dell S., To T. (2020). Effect of asthma exacerbation during pregnancy in women with asthma: A population-based cohort study. Eur. Respir. J..

[162] Gonçalves D.M.M., Wandalsen G.F., Scavacini A.S., Lanza F.C., Goulart A.L., Solé D., Dos Santos A.M.N. (2018). Pulmonary function in former very low birth weight preterm infants in the first year of life. Respir. Med..

[163] Den Dekker H.T., Jaddoe V.W.V., Reiss I.K., de Jongste J.C., Duijts L. (2018). Fetal and Infant Growth Patterns and Risk of Lower Lung Function and Asthma. The Generation R Study. Am. J. Respir. Crit. Care Med..

[164] Edwards C.A., Osman L.M., Godden D.J., Campbell D.M., Douglas J.G. (2003). Relationship between birth weight and adult lung function: Controlling for maternal factors. Thorax.

[165] Den Dekker H.T., Sonnenschein-van der Voort A.M.M., de Jongste J.C., Anessi-Maesano I., Arshad S.H., Barros H., Beardsmore C.S., Bisgaard H., Phar S.C., Craig L. (2016). Early growth characteristics and the risk of reduced lung function and asthma: A meta-analysis of 25,000 children. J. Allergy Clin. Immunol..

[166] Principi N., Di Pietro G.M., Esposito S. (2018). Bronchopulmonary dysplasia: Clinical aspects and preventive and therapeutic strategies. J. Transl. Med..

[167] Hollams E.M., de Klerk N.H., Holt P.G., Sly P.D. (2014). Persistent effects of maternal smoking during pregnancy on lung function and asthma in adolescents. Am. J. Respir. Crit. Care Med..

[168] Gilliland F.D., Li Y.F., Peters J.M. (2001). Effects of maternal smoking during pregnancy and environmental tobacco smoke on asthma and wheezing in children. Am. J. Respir. Crit. Care Med..

[169] Indinnimeo L., Porta D., Forastiere F., De Vittori V., De Castro G., Zicari A.M., Tancredi G., Melengu T., Duse M. (2016). Prevalence and risk factors for atopic disease in a population of preschool children in Rome: Challenges to early intervention. Int. J. Immunopathol. Pharmacol..

[170] Selby A., Munro A., Grimshaw K.E., Cornelius V., Keil T., Grabenhenrich L., Clausen M., Dubakiene R., Fiocchi A., Kowalski M.L. (2018). Prevalence estimates and risk factors for early childhood wheeze across Europe: The EuroPrevall birth cohort. Thorax.

[171] Burke H., Leonardi-Bee J., Hashim A., Pine-Abata H., Chen Y., Cook D.G., Britton J.R., McKeever T.M. (2012). Prenatal and passive smoke exposure and incidence of asthma and wheeze: Systematic review and meta-analysis. Pediatrics.

[172] Vardavas C.I., Hohmann C., Patelarou E., Martinez D., Henderson A.J., Granell R., Sunyer J., Torrent M., Fantini M.P., Gori D. (2016). The independent role of prenatal and postnatal exposure to active and passive smoking on the development of early wheeze in children. Eur. Respir. J..

[173] Collet C., Fayon M., Francis F., Galode F., Bui S., Debelleix S. (2021). The First 1000 Days: Impact of Prenatal Tobacco Smoke Exposure on Hospitalization Due to Preschool Wheezing. Healthcare.

[174] Bolat E., Arikoglu T., Sungur M.A., Batmaz S.B., Kuyucu S. (2017). Prevalence and risk factors for wheezing and allergic diseases in preschool children: A perspective from the Mediterranean coast of Turkey. Allergol. Immunopathol..

[175] Sahiner U.M., Buyuktiryaki B., Cavkaytar O., Yılmaz E.A., Soyer O., Sackesen C., Tuncer A., Sekerel B.E. (2013). Recurrent wheezing in the first three years of life: Short-term prognosis and risk factors. J. Asthma.

[176] Robison R.G., Kumar R., Arguelles L.M., Hong X., Wang G., Apollon S., Bonzagni A., Ortiz K., Pearson C., Pongracic J.A. (2012). Maternal smoking during pregnancy, prematurity and recurrent wheezing in early childhood. Pediatr. Pulmonol..

[177] Lodge C.J., Zaloumis S., Lowe A.J., Gurrin L.C., Matheson M.C., Axelrad C., Bennett C.M., Hill D.J., Hosking C.S., Svanes C. (2014). Early-life risk factors for childhood wheeze phenotypes in a high-risk birth cohort. J. Pediatr..

[178] Yilmaz O., Turkeli A., Onur E., Bilge S., Yuksel H. (2018). Secondhand tobacco smoke and severity in wheezing children: Nasal oxidant stress and inflammation. J. Asthma.

[179] Sadeghnejad A., Karmaus W., Arshad S.H., Kurukulaaratchy R., Huebner M., Ewart S. (2008). IL13 gene polymorphisms modify the effect of exposure to tobacco smoke on persistent wheeze and asthma in childhood, a longitudinal study. Respir. Res..

[180] Bals R., Boyd J., Esposito S., Foronjy R., Hiemstra P.S., Jiménez-Ruiz C.A., Katsaounou P., Lindberg A., Metz C., Schober W. (2019). Electronic cigarettes: A task force report from the European Respiratory Society. Eur. Respir. J..

[181] McCusker C., Upton J., Warrington R. (2018). Primary immunodeficiency. Allergy Asthma Clin. Immunol..

[182] Ozbek B., Ayvaz D.Ç., Esenboga S., Halaçlι S.O., Aytekin E.S., Yaz I., Tan Ç., Tezcan I. (2019). In case of recurrent wheezing and bronchiolitis: Think again, it may be a primary immunodeficiency. Asian Pac. J. Allergy Immunol..

[183] Siriaksorn S., Suchaitanawanit S., Trakultivakorn M. (2011). Allergic rhinitis and immunoglobulin deficiency in preschool children with frequent upper respiratory illness. Asian Pac. J. Allergy Immunol..

[184] Sandin A., Björkstén B., Böttcher M.F., Englund E., Jenmalm M.C., Bråbäck L. (2011). High salivary secretory IgA antibody levels are associated with less late-onset wheezing in IgE-sensitized infants. Pediatr. Allergy Immunol..

[185] Papadopoulou A., Mermiri D., Taousani S., Triga M., Nicolaidou P., Priftis K.N. (2005). Bronchial hyper-responsiveness in selective IgA deficiency. Pediatr. Allergy Immunol..

[186] Cinicola B.L., Pulvirenti F., Capponi M., Bonetti M., Brindisi G., Gori A., De Castro G., Anania C., Duse M., Zicari A.M. (2022). Selective IgA Deficiency and Allergy: A Fresh Look to an Old Story. Medicina.

[187] Vidarsson G., Dekkers G., Rispens T. (2014). IgG subclasses and allotypes: From structure to effector functions. Front. Immunol..

[188] Kim C.K., Park J.S., Chu S.Y., Kwon E., Kim H., Callaway Z. (2020). Low immunoglobulin G4 subclass level is associated with recurrent wheezing in young children. Asia Pac. Allergy.

[189] Zaitsu M., Matsuo M. (2022). Transient low IgG4 levels cause recurrent wheezing requiring multiple hospitalizations in infancy. Pediatr. Pulmonol..

[190] Prescott S.L., Björkstén B. (2007). Probiotics for the prevention or treatment of allergic diseases. J. Allergy Clin. Immunol..

[191] Frati F., Salvatori C., Incorvaia C., Bellucci A., Di Cara G., Marcucci F., Esposito S. (2018). The Role of the Microbiome in Asthma: The Gut-Lung Axis. Int. J. Mol. Sci..

[192] Azad M.B., Kozyrskyj A.L. (2012). Perinatal programming of asthma: The role of gut microbiota. Clin. Dev. Immunol..

[193] Wang M., Karlsson C., Olsson C., Adlerberth I., Wold A.E., Strachan D.P., Martricardi P.M., Aberg N., Perkin M.R., Tripodi S. (2008). Reduced diversity in the early fecal microbiota of infants with atopic eczema. J. Allergy Clin. Immunol..

[194] Abrahamsson T.R., Jakobsson H.E., Andersson A.F., Björkstén B., Engstrand L., Jenmalm M.C. (2012). Low diversity of the gut microbiota in infants with atopic eczema. J. Allergy Clin. Immunol..

[195] Zheng T., Yu J., Oh M.H., Zhu Z. (2011). The atopic march: Progression from atopic dermatitis to allergic rhinitis and asthma. Allergy Asthma Immunol. Res..

[196] Li L., Wang F., Liu Y., Gu F. (2019). Intestinal microbiota dysbiosis in children with recurrent respiratory tract infections. Microb. Pathog..

[197] Stokholm J., Thorsen J., Blaser M.J., Rasmussen M.A., Hjelmsø M., Shah S., Christensen E.D., Chawes B.L., Bønnelykke K., Brix S. (2020). Delivery mode and gut microbial changes correlate with an increased risk of childhood asthma. Sci. Transl. Med..

[198] Arrieta M.C., Stiemsma L.T., Dimitriu P.A., Thorson L., Russell S., Yurist-Doutsch S., Kuzeljevic B., Gold M.J., Britton H.M., Lefebvre D.L. (2015). Early infancy microbial and metabolic alterations affect risk of childhood asthma. Sci. Transl. Med..

[199] Abrahamsson T.R., Jakobsson H.E., Andersson A.F., Björkstén B., Engstrand L., Jenmalm M.C. (2014). Low gut microbiota diversity in early infancy precedes asthma at school age. Clin. Exp. Allergy.

[200] Bannier M.A.G.E., van Best N., Bervoets L., Savelkoul P.H.M., Hornef M.W., van de Kant K.D.G., Jöbsis Q., Dompeling E., Penders J. (2020). Gut microbiota in wheezing preschool children and the association with childhood asthma. Allergy.

[201] Jensen M.P., Meldrum S., Taylor A.L., Dunstan J.A., Prescott S.L. (2012). Early probiotic supplementation for allergy prevention: Long-term outcomes. J. Allergy Clin. Immunol..

[202] Cabana M.D., McKean M., Caughey A.B., Fong L., Lynch S., Wong A., Leong R., Boushey H.A., Hilton J.F. (2017). Early Probiotic Supplementation for Eczema and Asthma Prevention: A Randomized Controlled Trial. Pediatrics.

[203] Canani R.B., Di Costanzo M., Bedogni G., Amoroso A., Cosenza L., Di Scala C., Granata V., Nocerino R. (2017). Extensively hydrolyzed casein formula containing Lactobacillus rhamnosus GG reduces the occurrence of other allergic manifestations in children with cow’s milk allergy: 3-year randomized controlled trial. J. Allergy Clin. Immunol..

[204] Wei X., Jiang P., Liu J., Sun R., Zhu L. (2020). Association between probiotic supplementation and asthma incidence in infants: A meta-analysis of randomized controlled trials. J. Asthma.

[205] Cuello-Garcia C.A., Brożek J.L., Fiocchi A., Pawankar R., Yepes-Nuñez J.J., Terracciano L., Gandhi S., Agarwal A., Zhang Y., Schünemann H.J. (2015). Probiotics for the prevention of allergy: A systematic review and meta-analysis of randomized controlled trials. J. Allergy Clin. Immunol..

[206] Azad M.B., Coneys J.G., Kozyrskyj A.L., Field C.J., Ramsey C.D., Becker A.B., Friesen C., Abou-Setta A.M., Zarychanski R. (2013). Probiotic supplementation during pregnancy or infancy for the prevention of asthma and wheeze: Systematic review and meta-analysis. BMJ.

[207] Kuitunen M., Kukkonen K., Juntunen-Backman K., Korpela R., Poussa T., Tuure T., Haahtela T., Savilahti E. (2009). Probiotics prevent IgE-associated allergy until age 5 years in cesarean-delivered children but not in the total cohort. J. Allergy Clin. Immunol..

[208] Lin J., Zhang Y., He C., Dai J. (2018). Probiotics supplementation in children with asthma: A systematic review and meta-analysis. J. Paediatr. Child. Health.

[209] Rose M.A., Stieglitz F., Köksal A., Schubert R., Schulze J., Zielen S. (2010). Efficacy of probiotic Lactobacillus GG on allergic sensitization and asthma in infants at risk. Clin. Exp. Allergy.

[210] Rose M.A., Schubert R., Schulze J., Zielen S. (2011). Follow-up of probiotic Lactobacillus GG effects on allergic sensitization and asthma in infants at risk. Clin. Exp. Allergy.

[211] Elazab N., Mendy A., Gasana J., Vieira E.R., Quizon A., Forno E. (2013). Probiotic administration in early life, atopy, and asthma: A meta-analysis of clinical trials. Pediatrics.

[212] Esposito S., Lelii M. (2015). Vitamin D and respiratory tract infections in childhood. BMC Infect. Dis..

[213] Pfeffer P.E., Hawrylowicz C.M. (2018). Vitamin D in Asthma: Mechanisms of Action and Considerations for Clinical Trials. Chest.

[214] Xystrakis E., Kusumakar S., Boswell S., Peek E., Urry Z., Richards D.F., Adikibi T., Pridgeon C., Dallman M., Loke T.K. (2006). Reversing the defective induction of IL-10-secreting regulatory T cells in glucocorticoid-resistant asthma patients. J. Clin. Investig..

[215] Chambers E.S., Nanzer A.M., Pfeffer P.E., Richards D.F., Timms P.M., Martineau A.R., Griffiths C.J., Corrigan C.J., Hawrylowicz C.M. (2015). Distinct endotypes of steroid-resistant asthma characterized by IL-17A(high) and IFN-γ(high) immunophenotypes: Potential benefits of calcitriol. J. Allergy Clin. Immunol..

[216] Lan N., Luo G., Yang X., Cheng Y., Zhang Y., Wang X., Wang X., Xie T., Li G., Liu Z. (2014). 25-Hydroxyvitamin D3-deficiency enhances oxidative stress and corticosteroid resistance in severe asthma exacerbation. PLoS ONE.

[217] Gupta A., Sjoukes A., Richards D., Banya W., Hawrylowicz C., Bush A., Saglani S. (2011). Relationship between serum vitamin D, disease severity, and airway remodeling in children with asthma. Am. J. Respir. Crit. Care Med..

[218] Damera G., Fogle H.W., Lim P., Goncharova E.A., Zhao H., Banerjee A., Tliba O., Krymskaya V.P., Panettieri R.A. (2009). Vitamin D inhibits growth of human airway smooth muscle cells through growth factor-induced phosphorylation of retinoblastoma protein and checkpoint kinase 1. Br. J. Pharmacol..

[219] Principi N., Bianchini S., Baggi E., Esposito S. (2013). Implications of maternal vitamin D deficiency for the fetus, the neonate and the young infant. Eur. J. Nutr..

[220] Principi N., Esposito S. (2020). Vitamin D Deficiency During Pregnancy and Autism Spectrum Disorders Development. Front. Psychiatry.

[221] Lu M., Litonjua A.A., O’Connor G.T., Zeiger R.S., Bacharier L., Schatz M., Carey V.J., Weiss S.T., Mirzakhani H. (2021). Effect of early and late prenatal vitamin D and maternal asthma status on offspring asthma or recurrent wheeze. J. Allergy Clin. Immunol..

[222] Knihtilä H.M., Stubbs B.J., Carey V.J., Laranjo N., Chu S.H., Kelly R.S., Zeiger R.S., Bacharier L.B., O’Connor G.T., Lasky-Su J. (2021). Low gestational vitamin D level and childhood asthma are related to impaired lung function in high-risk children. J. Allergy Clin. Immunol..

[223] Li W., Qin Z., Gao J., Jiang Z., Chai Y., Guan L., Ge Y., Chen Y. (2019). Vitamin D supplementation during pregnancy and the risk of wheezing in offspring: A systematic review and dose-response meta-analysis. J. Asthma.

[224] Knihtilä H.M., Kelly R.S., Brustad N., Huang M., Kachroo P., Chawes B.L., Stokholm J., Bønnelykke K., Pedersen C.T., Bisgaard H. (2021). Maternal 17q21 genotype influences prenatal vitamin D effects on offspring asthma/recurrent wheeze. Eur. Respir. J..

[225] Dogru M., Kirmizibekmez H., Yesiltepe Mutlu R.G., Aktas A., Ozturkmen S. (2014). Clinical effects of vitamin D in children with asthma. Int. Arch. Allergy Immunol..

[226] Demirel S., Guner S.N., Celiksoy M.H., Sancak R. (2014). Is vitamin D insufficiency to blame for recurrent wheezing?. Int. Forum Allergy Rhinol..

[227] Beigelman A., Zeiger R.S., Mauger D., Strunk R.C., Jackson D.J., Martinez F.D., Morgan W.J., Covar R., Szefler S.J., Taussig L.M. (2014). The association between vitamin D status and the rate of exacerbations requiring oral corticosteroids in preschool children with recurrent wheezing. J. Allergy Clin. Immunol..

[228] Stenberg Hammar K., Hedlin G., Konradsen J.R., Nordlund B., Kull I., Giske C.G., Pedroletti C., Söderhäll C., Melén E. (2014). Subnormal levels of vitamin D are associated with acute wheeze in young children. Acta Paediatr..

[229] Al-Zayadneh E., Alnawaiseh N.A., Ajarmeh S., Altarawneh A.H., Albataineh E.M., AlZayadneh E., Shatanawi A., Alzayadneh E.M. (2020). Vitamin D deficiency in children with bronchial asthma in southern Jordan: A cross-sectional study. J. Int. Med. Res..

[230] Turkeli A., Ayaz O., Uncu A., Ozhan B., Bas V.N., Tufan A.K., Yilmaz O., Yuksel H. (2016). Effects of vitamin D levels on asthma control and severity in pre-school children. Eur. Rev. Med. Pharmacol. Sci..

[231] Urrutia-Pereira M., Solé D. (2016). Is Vitamin D Deficiency a Marker of Severity of Wheezing in Children? A Cross-sectional Study. J. Investig. Allergol. Clin. Immunol..

[232] Omand J.A., To T., O’Connor D.L., Parkin P.C., Birken C.S., Thorpe K.E., Maguire J.L. (2018). 25-hydroxyvitamin D and health service utilization for asthma in early childhood. Pediatr. Pulmonol..

[233] Adam-Bonci T.I., Cherecheș-Panța P., Bonci E.A., Man S.C., Cutaș-Benedec A., Drugan T., Pop R.M., Irimie A. (2020). Suboptimal Serum 25-Hydroxy-Vitamin D Is Associated with a History of Recent Disease Exacerbation in Pediatric Patients with Bronchial Asthma or Asthma-Suggestive Recurrent Wheezing. Int. J. Environ. Res. Public Health.

[234] Ducharme F.M., Jensen M., Mailhot G., Alos N., White J., Rousseau E., Tse S.M., Khamessan A., Vinet B. (2019). Impact of two oral doses of 100,000 IU of vitamin D3 in preschoolers with viral-induced asthma: A pilot randomised controlled trial. Trials.

[235] Rueter K., Jones A.P., Siafarikas A., Lim E.M., Prescott S.L., Palmer D.J. (2020). In “High-Risk” Infants with Sufficient Vitamin D Status at Birth, Infant Vitamin D Supplementation Had No Effect on Allergy Outcomes: A Randomized Controlled Trial. Nutrients.

[236] Vlieg-Boerstra B., de Jong N., Meyer R., Agostoni C., De Cosmi V., Grimshaw K., Milani G.P., Muraro A., Oude Elberink H., Pali-Schöll I. (2022). Nutrient supplementation for prevention of viral respiratory tract infections in healthy subjects: A systematic review and meta-analysis. Allergy.

[237] Martineau A.R., Jolliffe D.A., Greenberg L., Aloia J.F., Bergman P., Dubnov-Raz G., Esposito S., Ganmaa D., Ginde A.A., Goodall E.C. (2019). Vitamin D supplementation to prevent acute respiratory infections: Individual participant data meta-analysis. Health Technol. Assess..

[238] Jolliffe D.A., Camargo C.A., Sluyter J.D., Aglipay M., Aloia J.F., Ganmaa D., Bergman P., Bischoff-Ferrari H.A., Borzutzky A., Damsgaard C.T. (2021). Vitamin D supplementation to prevent acute respiratory infections: A systematic review and meta-analysis of aggregate data from randomised controlled trials. Lancet Diabetes Endocrinol..

[239] Chen Z., Peng C., Mei J., Zhu L., Kong H. (2021). Vitamin D can safely reduce asthma exacerbations among corticosteroid-using children and adults with asthma: A systematic review and meta-analysis of randomized controlled trials. Nutr. Res..

[240] Midodzi W.K., Rowe B.H., Majaesic C.M., Saunders L.D., Senthilselvan A. (2010). Early life factors associated with incidence of physician-diagnosed asthma in preschool children: Results from the Canadian Early Childhood Development cohort study. J. Asthma.

[241] Klopp A., Vehling L., Becker A.B., Subbarao P., Mandhane P.J., Turvey S.E., Lefebvre D.L., Sears M.R., Azad M.B., CHILD Study Investigators (2017). Modes of Infant Feeding and the Risk of Childhood Asthma: A Prospective Birth Cohort Study. J. Pediatr..

[242] Leung J.Y., Kwok M.K., Leung G.M., Schooling C.M. (2016). Breastfeeding and childhood hospitalizations for asthma and other wheezing disorders. Ann. Epidemiol..

[243] Xue M., Dehaas E., Chaudhary N., O’Byrne P., Satia I., Kurmi O.P. (2021). Breastfeeding and risk of childhood asthma: A systematic review and meta-analysis. ERJ Open Res..

[244] Elliott L., Henderson J., Northstone K., Chiu G.Y., Dunson D., London S.J. (2008). Prospective study of breast-feeding in relation to wheeze, atopy, and bronchial hyperresponsiveness in the Avon Longitudinal Study of Parents and Children (ALSPAC). J. Allergy Clin. Immunol..

[245] Dogaru C.M., Nyffenegger D., Pescatore A.M., Spycher B.D., Kuehni C.E. (2014). Breastfeeding and childhood asthma: Systematic review and meta-analysis. Am. J. Epidemiol..

[246] Patria M.F., Tenconi R., Esposito S. (2012). Efficacy and safety of influenza vaccination in children with asthma. Expert Rev. Vaccines.

[247] Grohskopf L.A., Sokolow L.Z., Broder K.R., Walter E.B., Fry A.M., Jernigan D.B. (2018). Prevention and Control of Seasonal Influenza with Vaccines: Recommendations of the Advisory Committee on Immunization Practices-United States, 2018–2019 Influenza Season. MMWR Recomm. Rep..

[248] Bianchini S., Argentiero A., Camilloni B., Silvestri E., Alunno A., Esposito S. (2019). Vaccination against Paediatric Respiratory Pathogens. Vaccines.

[249] Bae E.Y., Choi U.Y., Kwon H.J., Jeong D.C., Rhim J.W., Ma S.H., Lee K.I., Kang J.H. (2013). Immunogenicity and safety of an inactivated trivalent split influenza virus vaccine in young children with recurrent wheezing. Clin. Vaccine Immunol..

[250] Bergen R., Black S., Shinefield H., Lewis E., Ray P., Hansen J., Walker R., Hessel C., Cordova J., Mendelman P.M. (2004). Safety of cold-adapted live attenuated influenza vaccine in a large cohort of children and adolescents. Pediatr. Infect. Dis. J..

[251] Miller E.K., Dumitrescu L., Cupp C., Dorris S., Taylor S., Sparks R., Fawkes D., Frontiero V., Rezendes A.M., Marchant C. (2011). Atopy history and the genomics of wheezing after influenza vaccination in children 6–59 months of age. Vaccine.

[252] Baxter R.P., Lewis N., Fireman B., Hansen J., Klein N.P., Ortiz J.R. (2018). Live Attenuated Influenza Vaccination Before 3 Years of Age and Subsequent Development of Asthma: A 14-year Follow-up Study. Pediatr. Infect. Dis. J..

[253] Gaglani M.J., Piedra P.A., Riggs M., Herschler G., Fewlass C., Glezen W.P. (2008). Safety of the intranasal, trivalent, live attenuated influenza vaccine (LAIV) in children with intermittent wheezing in an open-label field trial. Pediatr. Infect. Dis. J..

[254] Sokolow A.G., Stallings A.P., Kercsmar C., Harrington T., Jimenez-Truque N., Zhu Y., Sokolow K., Moody M.A., Schlaudecker E.P., Walter E.B. (2022). Safety of Live Attenuated Influenza Vaccine in Children With Asthma. Pediatrics.

[255] Turner P.J., Fleming L., Saglani S., Southern J., Andrews N.J., Miller E., SNIFFLE-4 Study Investigators (2020). Safety of live attenuated influenza vaccine (LAIV) in children with moderate to severe asthma. J. Allergy Clin. Immunol..

[256] Ambrose C.S., Wu X., Knuf M., Wutzler P. (2012). The efficacy of intranasal live attenuated influenza vaccine in children 2 through 17 years of age: A meta-analysis of 8 randomized controlled studies. Vaccine.

[257] Toivonen L., Karppinen S., Schuez-Havupalo L., Teros-Jaakkola T., Vuononvirta J., Mertsola J., He Q., Waris M., Peltola V. (2016). Burden of Recurrent Respiratory Tract Infections in Children: A Prospective Cohort Study. Pediatr. Infect. Dis. J..

[258] Principi N., Esposito S., Cavagna R., Bosis S., Droghetti R., Faelli N., Tosi S., Begliatti E., Snoopy Study Group (2003). Recurrent respiratory tract infections in pediatric age: A population-based survey of the therapeutic role of macrolides. J. Chemother..

[259] Esposito S., Soto-Martinez M.E., Feleszko W., Jones M.H., Shen K.L., Schaad U.B. (2018). Nonspecific immunomodulators for recurrent respiratory tract infections, wheezing and asthma in children: A systematic review of mechanistic and clinical evidence. Curr. Opin. Allergy Clin. Immunol..

[260] Esposito S., Bianchini S., Bosis S., Tagliabue C., Coro I., Argentiero A., Principi N. (2019). A randomized, placebo-controlled, double-blinded, single-centre, phase IV trial to assess the efficacy and safety of OM-85 in children suffering from recurrent respiratory tract infections. J. Transl. Med..

[261] Cao C., Wang J., Li Y., Li Y., Ma L., Abdelrahim M.E.A., Zhu Y. (2021). Efficacy and safety of OM-85 in paediatric recurrent respiratory tract infections which could have a possible protective effect on COVID-19 pandemic: A meta-analysis. Int. J. Clin. Pract..

[262] Yin J., Xu B., Zeng X., Shen K. (2018). Broncho-Vaxom in pediatric recurrent respiratory tract infections: A systematic review and meta-analysis. Int. Immunopharmacol..

[263] Schaad U.B. (2010). OM-85 BV, an immunostimulant in pediatric recurrent respiratory tract infections: A systematic review. World J. Pediatr..

[264] Esposito S., Musio A. (2013). Immunostimulants and prevention of recurrent respiratory tract infections. J. Biol. Regul. Homeost. Agents.

[265] Razi C.H., Harmancı K., Abacı A., Özdemir O., Hızlı S., Renda R., Keskin F. (2010). The immunostimulant OM-85 BV prevents wheezing attacks in preschool children. J. Allergy Clin. Immunol..

