# Supplementary material for: Risk Factors Affecting Development and Persistence of Preschool Wheezing: Consensus Document of the Emilia-Romagna Asthma (ERA) Study Group
Source: J Clin Med. 2022 Nov 4;11(21):6558. doi: 10.3390/jcm11216558 (PMC9655250; doi:10.3390/jcm11216558)
Supplement: Supplementary file 1 [file jcm-11-06558-s001.zip › jcm-1954694-supplementary.pdf]

Supplementary material S1: PICO questions and review of the literature

Risk factors affecting development and persistence of preschool wheezing: consensus document of the Emilia-Romagna Asthma (ERA) Study Group

Results of the clinical questions

Search strategies, Prisma flow chart showing the selection process of the papers and the tables with the included studies are presented for each question.

SECTION 1 – PATHOGENESIS OF PRESCHOOL WHEEZING

Question 1. What is the role of infection in the pathogenesis of preschool wheezing?

Search strategy:

((child, preschool[MeSH Terms]) OR (toddler*)) AND (((respiratory sound[MeSH Terms]) OR (wheez*)) OR (asthma[MeSH Terms:NoExp]))) AND ((((infections, upper respiratory[MeSH Terms]) OR (virus*)) OR (viral infection*)) OR (rhinovirus*)) AND ((english[Filter]) AND (2008:2021[pdat]))

What is the role of infection in the pathogenesis of preschool wheezing?

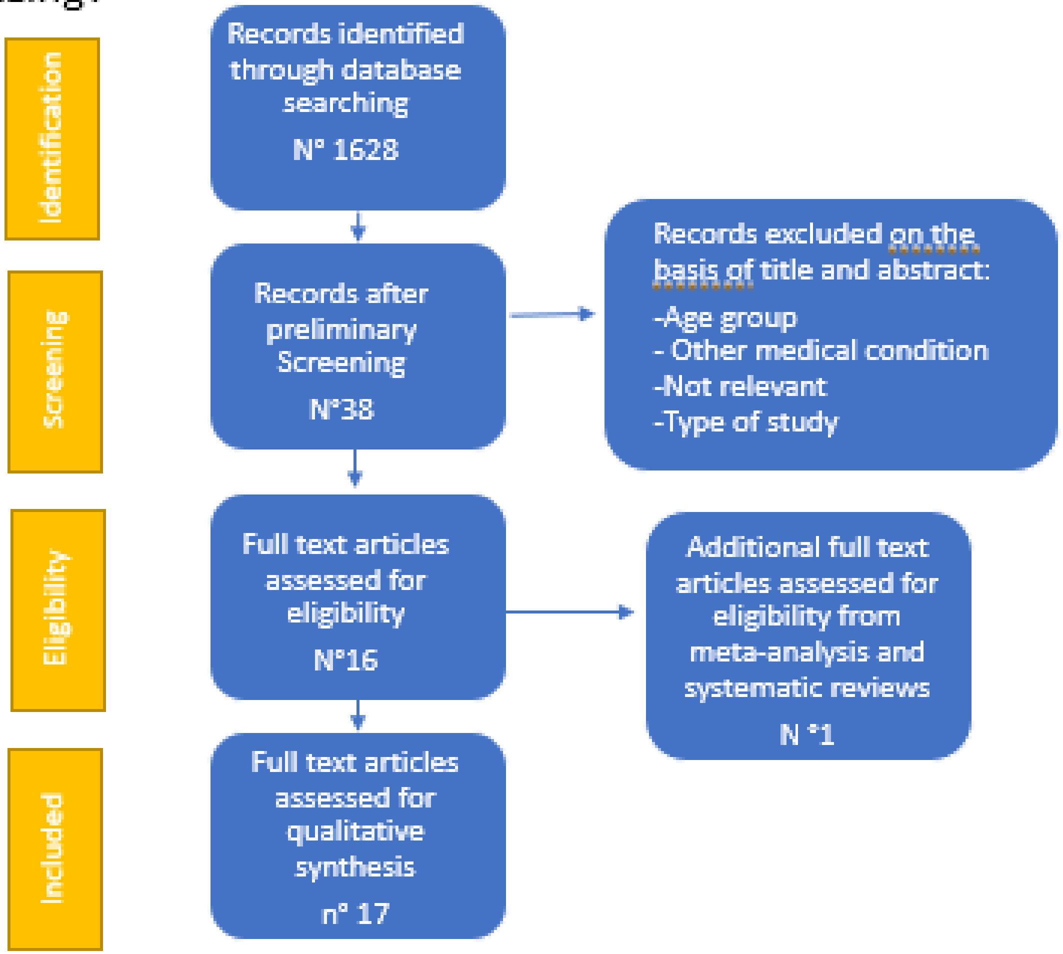

Modified from: Moher D, Liberati A, Tetzlaff J, Altman DG, The PRISMA Group (2009). Preferred Reporting Items for Systematic Reviews and Meta-Analyses: The PRISMA Statement. PLoS Med 6(7): e1000097

Title of the study, first author, year [ref]	Type of study	Study design	Population	N of patients, age	Experiments/mechanisms assessed	Outcome (primary and secondary)	Results	OR/RR (95%IC)
Rhinovirus illnesses during infancy predict subsequent childhood wheezing.<br><br>Lemanske RF, 2005<br><br>[1]	Prospective cohort study	Evaluation of wheezing development in a birth cohort at risk for allergies followed until 3 years of age	American	285<br>(0-3 years)	Nasopharyngeal mucus samples were collected during scheduled clinic visits (at 2, 4, 6, 9, and 12 months of age) and during times of acute respiratory illnesses.<br><br>Nasal specimens were analyzed for respiratory viruses including RSV, rhinovirus, influenza types A and B, parainfluenza virus types 1 to 4, adenovirus, and nonrhinovirus picornaviruses (NRVPs) by using standard techniques.8 Samples were also evaluated for rhinovirus RNA by seminested RT-PCR.8.	Risk factors for wheezing at 3 years age:		
						Passive smoke exposure	Augmented risk	2.1 (1.2-3.8)
						Older siblings	Augmented risk	2.5 (1.4-4.4)
						Allergic sensitization to foods at age 1 year	Augmented risk	2.0 (1.1-3.6)
						Any moderate to severe respiratory illness without wheezing during infancy	Augmented risk	3.6 (1.2 -11)
						At least 1 wheezing illness during infancy with respiratory syncytial virus (RSV; OR 5 3.0),	Augmented risk	3.0 (1.6-5.8)
						rhinovirus	Augmented risk	10 (4.7-23)
						non–rhinovirus/RSV pathogens.	Augmented risk	3.9 (1.9-8.1)

						When viral etiology was considered, 1st-year wheezing illnesses caused by rhinovirus infection were the strongest predictor of subsequent 3rd year wheezing.		6.6 (p <0.0001).
						Moreover, 63% of infants who wheezed during rhinovirus seasons continued to wheeze in the 3rd year of life, compared with only 20% of all other infants.		6.6; (p<0.0001).
Early-life rotavirus and norovirus infections in relation to development of atopic manifestation in infants.<br><br>Reimerink J, 2009.<br><br>[2]	Prospective cohort study	Evaluation of a birth cohort at 1 year age until 2 years old. Children were divided into 2 groups: infants who complied with a blood sample (n = 612) and those who did not (n = 508)	Dutch children	1120<br>(0 – 2 years)	At 12 months of life, a sample of capillary blood was collected to determine total IgE and specific IgE against hen's eggs, cow's milk and peanuts. At 2 years of age, venous blood samples were collected for determination of total and specific IgE against hen's egg,	Prevalence of serology positive for Rotavirus and/or Norovirus	Seroprevalence of rotavirus at 1 year of age: 39% (IgG) and 29% (IgA). Seroprevalence for norovirus GGII.4: 43% (IgG) and 18% (IgA). Seroprevalence for norovirus GGI.1: 19% (IgG) and 2% (IgA).	

					cow's milk, peanut, birch pollen, grass pollen, cat, dog and HDM.<br>Also, Rotavirus and Norovirus (genogroup II and GGI) specific antibodies (IgG and IgA) were determined. Questionnaires were used to assess eczema and/or wheezing development.	Atopic sensitization wheezing and eczema development		
						Rotavirus seropositivity and risk of recurrent wheeze in the first and second year of life	Augmented risk	OR=3.1 (1.1-9.1)
						Rotavirus seropositivity and persistent and new recurrent wheeze	Augmented risk	OR=2.7 (1.1-6.2)
A molecular epidemiological study of respiratory viruses detected in Japanese children with acute wheezing illness.<br><br>Fujitsuka A, 2011<br><br>[3]	Epidemiological study	Children with *acute wheezing*: 39 had a history of wheezing, 76 patients had no such history		115<br>(20.8 ± 25.7 months)	Nasopharyngeal swabs were collected and analysed for viral DNA/RNA extraction. Homology and phylogenetic analysis were performed.	Presence of RSV, HRV and correlation with history of wheeze/asthma.	RSV was the most common specie detected in patients with no history of wheezing and/or asthma (38 patients vs. 9 patients, $p< 0.05$), while HRV was dominant in those with a history of wheezing and/or asthma (12 patients vs. 24 patients, $p< 0.05$).	
Wheeze in Preschool Age Is Associated with Pulmonary Bacterial Infection and Resolves after Antibiotic Therapy.<br><br>Schwerk N, 2011<br><br>[4]	Retrospective cohort study	A population of children with *recurrent or persistent wheeze* who underwent flexible bronchoscopy and bronchoalveolar lavage (BAL). People who respected inclusion criteria (n=42) were compared to		98<br>(1 – 64 months)	The first aliquot of BAL was used for bacterial cultures and the second one for viruses' detection (Influenza A and B, parainfluenza types 1–3, herpes simplex, rhinovirus, metapneumovirus, respiratory syncytial virus and adenovirus). Finally, the third part of BAL was used for	Elevated bacterial count and neutrophilia in BAL Positivity of H. influenzae, Streptococcus pneumoniae, Moraxella catarrhalis, Staphylococcus aureus or other bacteria.<br>Presence of viral infection.<br>Symptoms improvement after antibiotic therapy.<br>.	Significant increase in both neutrophilia and bacterial count were observed in patients compared to the control group ($p <0.005$).<br>Bacteria species cultivated from the patients consisted mostly of H. influenzae, S. pneumoniae and M.catarrhalis, whereas none of the controls were colonized with these bacteria.	

		healthy controls (n=14).<br>Patients with acute respiratory infection symptoms were excluded.			cytological analysis.<br>Of the 42 patients, 35 were followed up for 6 months: a group (n=26) received antibiotics therapy and another group (n=9) used only anti-asthmatic medication.		Viral infection was detected in two patients (RS Virus and parainfluenza Type II).<br>92% of patients treated with appropriated antibiotics presented fewer symptoms and less exacerbations.	
Rhinovirus bronchiolitis and recurrentwheezing: 1-year follow-up.<br><br>Midulla F, 2012<br><br>[5]	Prospective cohort study	Patients (n=313) were recruited during a hospitalization for wheezing and followed for 1 year. Results were compared to healthy controls (n=39) w		313<br>(0-12 months)	Questionnaires were administered to evaluate demographical information.<br>Laboratory tests were realised.<br>Nasal washings were evaluated for virus.	Recurrent wheezing	52.7% in patients group vs 10.3% of controls group (p=0.001)	
						Risk factors of recurrent wheeze<br><br>RV infection	 <br><br>Augmented risk	 <br><br>3.3 (1.0–11.1)
						Positive family history for asthma	Augmented risk	2.5 (1.2–4.9)
In young children, persistent wheezing is associated with bronchial bacterial infection: a retrospective analysis.<br><br>De Schutter I, 2012<br><br>[6]	Retrospective cohort study	A population of *persistent wheezers* unresponsive to inhaled corticosteroid (ICS) therapy, who underwent flexible bronchoscopy with bronchoalveolar lavage (BAL).		33<br>(4 – 38 months)	BAL-samples were processed for smear, bacterial culture, viral culture, and for the detection of influenzavirus A and B, parainfluenzavirus types1, 2, 3, RSV, human metapneumovirus subtypes A and B, coronaviruses 229E and OC43, Mycoplasma pneumoniae and Chlamydophila	Evidence of bacterial and/or viral bronchial infection.<br><br>Cytological findings	Significant bacterial BAL cultures were found in 48,5 % of patients. Haemophilus influenzae was isolated in 30,3%, Streptococcus pneumoniae in 12,1 % and Moraxella catarrhalis in 12,1 %. All H.influenzae isolated were non-encapsulated strains and definitely distinguished from non-haemolytic H. haemolyticus. Respiratory viruses were detected in 21,9 % of cases with	

					pneumoniae. Cytological analysis was also performed.		mixed bacterial-viral infection in 12,1 %. Cytology revealed a marked neutrophilic inflammation	
Human rhinovirus and wheezing: Short and long-term associations in children.<br><br>Van Der Gugten A, 2013<br><br>[7]	Prospective cohort study	A birth cohort was prospectively evaluated while asymptomatic and also during acute wheeze		140<br>(2 weeks – 4 years)	Nose and throat swabs were collected monthly during the 1st year of life, regardless of any symptoms. Polymerase chain reaction was used to detect an extensive panel of respiratory pathogens. Lung function was measured before 2 months of age. Information on respiratory symptoms was collected by daily questionnaires and electronic patient files	Human rhinovirus infection<br><br>Other viruses' infection	1425 samples in 140 infants were collected. Both the presence of single or multiple pathogens (HRV equal to other pathogens) and increased respiratory system resistance were significantly associated with lower respiratory symptoms during infancy.	
						Wheezing development	HRV presence during infancy was not associated with the risk of wheezing at age 4.	
						Effect of HRV infection episode with wheezing and risk of wheezing at age 4.	Augmented risk<br><br>Association weakened after adjustment for lung function	OR=1.9 (1.1-3.5)

						Association between HRV and	This association weakened after adjustment for lung function	OR=1.4 (0.7-2.9)
Lower Respiratory Tract Infections Associated with Rhinovirus during Infancy and Increased Risk of Wheezing during Childhood. A Cohort Study.<br><br>O'Callaghan-Gordo C, 2013<br><br>[8]	Prospective cohort study	Children were enrolled during an *acute lower respiratory tract infection* and then divided in children positive at human rhinovirus (n 55) and negative at human rhinovirus (n 165)		220<br>(0-5 years)	Nasopharyngeal aspirates were collected on admission to the Hospital for viral determination. Children were classified according to presence or not of rhinovirus. The study cohort was passively followed-up at the Manhiça District Hospital for up to 4 years and 9 months	Detection of Human Rhinovirus	25% (n 55) of children had Rhinovirus detected during the LRTI episode, while 75% (n=165) tested negative for rhinovirus.	
						Development of Wheeze in infants hospitalized with LRTI associated with rhinovirus	Augmented risk	RR=1.68 (1.02-2.75); Wald test p-value = 0.039
							No evidence of increased incidence rate of visits with wheezing was observed for the remaining follow-up period	

Clinical and epidemiologic factors related to subsequent wheezing after virus-induced lower respiratory tract infections in hospitalized pediatric patients younger than 3 years.<br><br>Takeyama A, 2014<br><br>[9]	Prospective cohort study	Patients were recruited during a hospitalization for lower respiratory tract infections. They were divided into two groups: the wheezing at admission group (n=153) and the no wheezing at admission group (n=259).<br>216 of them were followed for 3 years to characterize wheezing development.	Japanese	416<br>(1 – 47 months)	Nasopharyngeal aspirates were collected at the admission and examined for the presence of respiratory syncytial virus, rhinovirus, parainfluenza-3 virus, human metapneumovirus (hMPV), and influenza virus (Flu). Clinical signs were assessed using a severity scoring system. Questionnaires were administered to evaluate wheezing development after discharge.	Viral infection during wheezing episode<br><br>Patients with wheezing at admission and RV infection who developed subsequent wheezing were 9 of 11 (p=0.005).<br><br>Patients with wheezing at admission and VRS infection who developed subsequent wheezing were 11 of 23 (p=0.765)	RSV 33%, RV 14%, RSV+RV 8%, Influenza virus 8%, PIV-3 5%, MPV 3% of patients.	
Recurrent wheezing 36 months after bronchiolitis is associated with rhinovirus infections and blood eosinophilia.<br><br>Midulla F, 2014<br><br>[10]	Prospective cohort study	Children were followed for 3 years after hospitalization for bronchiolitis.		313<br>(7-11 months)	Nasal wash specimens were analysed by real-time polymerase chain reaction to find a total of 14 respiratory viruses: RSV, influenza virus A and B, human coronavirus OC43, 229E, NL-63, HUK1, adenovirus, rhinovirus, parainfluenza virus 1–3, human bocavirus and human metapneumovirus. Parents were interviewed 12, 24 and 36 months after their child was discharged, to gather information on possible wheezing episodes	Wheezing episodes	137children (51.7%) at 12 months, 117 (48.3%) at 24 months and 93 (40.4%) at36 months.	
						Rrisk of recurrent wheezing after three years:<br><br>- if they had episodes of wheezing during the first year after bronchiolitis,	Augmented risk	7.2 (3.9 - 13.3)
						-if they had episodes of wheezing during the second year	Augmented risk	16.8 (8.7–32.7)
						- if they wheezed during both years	Augmented risk	55.0 (22.7–133.2)

						Blood eosinophils>400 cells/uL	Augmented risk	7.7 (1.4–41.8)
						Rhinovirus infections		3.1 (1.0–9.4)
Early-life risk factors for childhood wheeze phenotypes in a high-risk birth cohort.<br><br>Lodge C, 2014<br><br>[11]	Randomized controlled trial	Children from a birth cohort *at risk for allergy* were followed up and divided by latent class analysis in five wheeze phenotypes: never/infrequent wheeze; early transient wheeze; early persistent wheeze; intermediate-onset wheeze and late-onset wheeze.	Australian<br><br>Children at risk for allergy	620<br>(0 – 12 years)	Telephone surveys about wheezing symptoms were conducted every 4 weeks from birth to age 15 months, once at age 18 months, yearly at age 2-7 years, and once at 12 years.<br>Potential early-life risk factors were also indagated: parental asthma, education, and smoking; infant sex and 4- week weight; breastfeeding for <3 months; adiposity (2-year body mass index [BMI]); child atopy, defined as sensitization (food at 1 year, aeroallergen at 2 years, capturing the earliest/most prevalent) and early eczema (6 months as a strong indicator of allergic predisposition); exposure to cats and/or dogs; lower	Risk factors for development of **early transient wheeze**:<br><br>Lower respiratory tract infection (LRTI) by 1 year	Increased risk	RR= 3.00 (1.58-5.70),
						Childcare by 1 year	Increased risk	RR=1.51(1.02-2.22)
						Higher body mass index	Increased risk	RR=2.51 (1.09-5.81)
						Breastfeeding	Reduced risk	RR=0.54 (0.32-0.90).
						Risk factors for development of **early persistent wheeze**<br><br>LRTI	Increased risk	RR=6.54 (2.55-16.76)
						aeroallergen sensitization	Increased risk	RR=4.95 (1.74-14.02)

					respiratory tract infection (LRTI) in the first year; first-born status; and childcare attendance by age 12 months.	Risk factors for development of **intermediate-onset wheeze.** LRTI	Increased risk	RR=5.31 (2.71-10.41)
						Eczema	Increased risk	RR=2.77 (1.78-4.31),
						aeroallergen sensitization	Increased risk	RR=5.60 (2.86-10.9)
						food sensitization	Increased risk	RR=2.77 (1.56-4.94)
						Dog exposure at baseline	Reduced risk	RR=0.52 (0.32-0.84)
						first-born status	Reduced risk	RR=0.49 (0.32-0.76)
						Risk factors for development of **late-onset wheeze** Heavy parental smoking at birth	Increased risk	RR=3.18 (1.02-9.88)
						Breastfeeding	Reduced risk	RR=0.34 (0.12-0.96).
Rhinovirus wheezing illness in infancy is associated with medically attended third year wheezing in low-risk infants: results of a healthy birth cohort study.	Prospective cohort study	A birth cohort at low risk for asthma was follower until		181 (0-3 years)	Children were followed during first year of life by daily logs to evaluate	Wheezing at 3 years of life in children RV-WI vs children without RV-WI	Augmented risk in children with RV-WI	39% vs 6%. OR= 9.7 (3.1–33.5) p<0.0001

de Winter J, 2015<br><br>[12]		three years of life. Children were divided into groups: patients with rhinovirus wheezing illness (RV-WI) (n=18) and patients without rhinovirus wheezing episodes (n=163).			rhinovirus wheezing development and simultaneous molecular rhinovirus detection. Respiratory function and blood eosinophil count were both measured in the first month of life.			
Associations between patient clinical characteristics and the presence of cytomegalovirus DNA in the bronchoalveolar lavage fluid of children with recurrent wheezing.<br><br>Sun H, 2018<br><br>[13]	Cross-sectional study	Children with *recurrent wheezing* were enrolled and divided in patients with CMV in BALF (n 57) and patients without CMV in BALF (n 54).	Chinese Recurrent wheezers	111<br>(4 to 36 months)	At the admission to the hospital, clinical information was collected, and blood tests were performed, including eosinophils count and counts of subpopulations of lymphocytes.<br>Also, a flexible fibreoptic bronchoscopy and bronchoalveolar lavage fluid testing were effectuated.<br>On BALF, Cytomegalovirus DNA were researched by real-time PCR.	Presence of CMV DNA<br><br>Correlation with age, modified asthma predictive index (mAPI), duration of the hospitalization and eosinophils in BALF.	Cytomegalovirus DNA was detected in 51.4% of patients (n = 111) and it was more prevalent among patients aged 12 to 36 months with a positive modified asthma predictive index (mAPI) (n = 38, median 23.5 (IQR 19.7-31.2) months) than in those of the same age group with a negative mAPI (n = 25, median 15.0 (IQR 13.0-19.0) months) (57.9% vs. 20.0%, p = 0.003).<br>Bronchoalveolar lavage fluid CMV DNA copy number [median 7560 (IQR 1200-71,150) copies/mL] was positively correlated with the duration of hospitalization (r = 0.33, p = 0.013), and negatively correlated with patient age (r = - 0.41, p = 0.002) and the percentage of BALF eosinophils (r = - 0.38, p = 0.004).	

Recurrent wheezing in children following human metapneumovirus infection.<br><br>Coverstone A, 2018.<br><br>[14]	Prospective cohort study	Children with Human metapneumovirus (HMPV) lower respiratory tract infection (n 38) compared to healthy controls (n 30) and followed for wheezing and/or asthma development	America, asymptomatic for wheezing or asthma	68<br>(0 – 5 years)	Nasal swabs were collected from children symptomatic for lower respiratory tract infection and analysed for HMPV. Patients positive for HMPV infection were included and followed up every 3-6 months by phone calls or clinical visit to indagate wheezing and/or asthma development.		79% of HMPV children with follow-up data experienced any wheezing in follow-up compared to controls (44%) (p=0.007). The HMPV group experienced a higher rate of wheezing episodes compared to controls (1.2±1.4 vs 0.6±1.1 events/year, p=0.02). Asthma was diagnosed in a numerically higher proportion of children in the HMPV group (9 of 29 [31%]) in follow-up compared to controls (4 of 27 [15%]); however, this was not statistically significant (p=0.15).	
						HMPV LRTI and risk of wheezing after enrolment	Augmented risk	HR 2.8 (1.4-5.8) p=0.005
						HMPV LRTI and risk of wheezing with a cold	Augmented risk	HR 2.5 (1.1-5.2) p=0.02)
						HMPV LRTI and risk of wheezing apart from a cold	Augmented risk	HR 4.6 (1.7-12.4) p=0.003

							Results did not change when adjusted for age at enrolment, baseline eosinophil percentage or maternal smoking status.	
Pulmonary function and bronchial reactivity 4 years after the first virus-induced wheezing.<br><br>Leino A, 2019<br><br>[15]	Prospective cohort study	Children with their first virus-induced wheezing episode at the ages of 3 to 23 months		76<br>(0 – 6 years)	At study entry, viral etiology, Rhinovirus genome load, atopic and clinical characteristics, and standardized questionnaire were analysed.<br>At 4 -year follow -up visit, pulmonary function was evaluated by impulse oscillometry with exercise challenge.	Viral infection at first wheeze episode.<br>Atopy at study entry<br><br>Bronchial reactivity	At study entry, 57 (75%) were rhinovirus positive and 22 (30%) were sensitized.<br>Four years later, 37 (49%) were using asthma medication regularly<br><br>Bronchial reactivity (≥35% change in mean crude values of resistance) after exercise challenge or bronchodilation was present in 9 (12%) children.	
						Correlation between atopy and bronchial reactivity development	Augmented risk	OR 8.8<br>(p=0.03)
Lower airway microbiota associates with inflammatory phenotype in severe preschool wheeze.<br><br>Robinson P, 2019<br><br>[16]	Prospective cohort study	Patients with *severe wheezing* who underwent a clinically indicated bronchoscopy.<br><br>Children were divided in Episodic viral		35<br>(12-72 months)	Patients were clinically assessed using questionnaires and divided in EVW and MTW.<br>BAL was analysed to find bacterial and/or viral infection and to determinate microbiota	Viral and/or bacterial infection.<br><br>Microbiota characteristics<br><br>Cytological differences	MTWs had more BAL fluid macrophages than EVWs, while EVWs had an approximately 6 times greater median BAL neutrophil count than MTW.<br>Numbers of EVWs and MTWs with positive BAL fluid bacterial culture or	

		wheezers (EVWs) (n=14) and multiple-trigger wheezers (MTWs) (n=21)			characteristics.<br><br>Cytological analysis of BAL was also performed.		viral detection were similar.<br>Greater BAL fluid neutrophil counts were associated with positive BAL fluid bacterial cultures in MTWs but not EVWs.<br>Microbiota findings revealed 2 distinct profiles referred as ‘‘mixed’’ or ‘‘Moraxella’’.<br>The Moraxella profile had lower proportions of BAL fluid macrophages and lymphocytes but significantly higher neutrophil counts.<br><br>Microbiota profiles did not significantly associate with MTW or EVW phenotypes. However, the majority of EVWs had a Moraxella profile and also a trend toward greater BAL neutrophil counts.	
Association between rhinovirus wheezing illness and the development of childhood asthma: A meta-analysis.<br><br>Liu L, 2017<br><br>[17]	Metanalysis	All studies selected included children with a confirmed diagnosis of Rhinovirus infection. Maximum age at the time of wheeze presentation was 3 years old.	American, Australian, Finnish, Italian, Dutch, Thai, Japanese	3183<br>(0 – 18 years)	Studies were selected if eligible: (1) original article; (2) the maximum age at the time of wheezing was 3 years; (3) diagnosis of RV infection was virologically confirmed in all cases; (4)a follow-up period was included; (5) outcome of interest wheezing/asthma.	Asthma or wheezing an RV wheezing illness in the first 3 years of life.	15 original articles met the criteria, while 10 articles reported the results of 4 longitudinal cohort studies with different follow-up periods.	
							Increased risk	RR=2.00<br>(1.62-2.49)<br>p<0.001
						Association in patients <10 years	Increased risk	RR=2.02<br>(1.70 -2.39)

								$p<0.001$
						Association in patient ≥10 years	Increased risk	RR=1.92 (1.36-2.72) $p<0.001$

References

1. Lemanske RF Jr, Jackson DJ, Gangnon RE, Evans MD, Li Z, Shult PA, Kirk CJ, Reisdorf E, Roberg KA, Anderson EL, Carlson-Dakes KT, Adler KJ, Gilbertson-White S, Pappas TE, Dasilva DF, Tisler CJ, Gern JE. Rhinovirus illnesses during infancy predict subsequent childhood wheezing. J Allergy Clin Immunol. 2005 Sep;116(3):571-7. doi: 10.1016/j.jaci.2005.06.024. PMID: 16159626.
2. Reimerink J, Stelma F, Rockx B, Brouwer D, Stobberingh E, van Ree R, Dompeling E, Mommers M, Thijs C, Koopmans M. Early-life rotavirus and norovirus infections in relation to development of atopic manifestation in infants. Clin Exp Allergy. 2009 Feb;39(2):254-60. doi: 10.1111/j.1365-2222.2008.03128.x. Epub 2008 Nov 17. PMID: 19032360.
3. Fujitsuka A, Tsukagoshi H, Arakawa M, Goto-Sugai K, Ryo A, Okayama Y, Mizuta K, Nishina A, Yoshizumi M, Kaburagi Y, Noda M, Tashiro M, Okabe N, Mori M, Yokota S, Kimura H. A molecular epidemiological study of respiratory viruses detected in Japanese children with acute wheezing illness. BMC Infect Dis. 2011 Jun 10;11:168. doi: 10.1186/1471-2334-11-168. PMID: 21663657; PMCID: PMC3123215.
4. Schwerk N, Brinkmann F, Soudah B, Kabesch M, Hansen G. Wheeze in preschool age is associated with pulmonary bacterial infection and resolves after antibiotic therapy. PLoS One. 2011;6(11):e27913. doi: 10.1371/journal.pone.0027913. Epub 2011 Nov 29. PMID: 22140482; PMCID: PMC3226624.
5. Midulla F, Pierangeli A, Cangiano G, Bonci E, Salvadei S, Scagnolari C, Moretti C, Antonelli G, Ferro V, Papoff P. Rhinovirus bronchiolitis and recurrent wheezing: 1-year follow-up. Eur Respir J. 2012 Feb;39(2):396-402. doi: 10.1183/09031936.00188210. Epub 2011 Aug 18. PMID: 21852336.
6. De Schutter I, Dreesman A, Soetens O, De Waele M, Crokaert F, Verhaegen J, Piérard D, Malfroot A. In young children, persistent wheezing is associated with bronchial bacterial infection: a retrospective analysis. BMC Pediatr. 2012 Jun 22;12:83. doi: 10.1186/1471-2431-12-83. PMID: 22726254; PMCID: PMC3420249.
7. van der Gugten AC, van der Zalm MM, Uiterwaal CS, Wilbrink B, Rossen JW, van der Ent CK. Human rhinovirus and wheezing: short and long-term associations in children. Pediatr Infect Dis J. 2013 Aug;32(8):827-33. doi: 10.1097/INF.0b013e318290620e. PMID: 23584579.
8. O'Callaghan-Gordo C, Bassat Q, Díez-Padrisa N, Morais L, Machevo S, Nhampossa T, Quintó L, Alonso PL, Roca A. Lower respiratory tract infections associated with rhinovirus during infancy and increased risk of wheezing during childhood. A cohort study. PLoS One. 2013 Jul 31;8(7):e69370. doi: 10.1371/journal.pone.0069370. PMID: 23935997; PMCID: PMC3729956.
9. Takeyama A, Hashimoto K, Sato M, Sato T, Tomita Y, Maeda R, Ito M, Katayose M, Kawasaki Y, Hosoya M. Clinical and epidemiologic factors related to subsequent wheezing after virus-induced lower respiratory tract infections in hospitalized pediatric patients younger than 3 years. Eur J Pediatr. 2014 Jul;173(7):959-66. doi: 10.1007/s00431-014-2277-7. Epub 2014 Feb 18. PMID: 24535712.
10. Midulla F, Nicolai A, Ferrara M, Gentile F, Pierangeli A, Bonci E, Scagnolari C, Moretti C, Antonelli G, Papoff P. Recurrent wheezing 36 months after bronchiolitis is associated with rhinovirus infections and blood eosinophilia. Acta Paediatr. 2014 Oct;103(10):1094-9. doi: 10.1111/apa.12720. Epub 2014 Jul 7. PMID: 24948158; PMCID: PMC7159785.
11. Lodge CJ, Zaloumis S, Lowe AJ, Gurrin LC, Matheson MC, Axelrad C, Bennett CM, Hill DJ, Hosking CS, Svanes C, Abramson MJ, Allen KJ, Dharmage SC. Early-life risk factors for childhood wheeze phenotypes in a high-risk birth cohort. J Pediatr. 2014 Feb;164(2):289-94.e1-2. doi: 10.1016/j.jpeds.2013.09.056. Epub 2013 Nov 14. PMID: 24238860.

12. de Winter JJ, Bont L, Wilbrink B, van der Ent CK, Smit HA, Houben ML. Rhinovirus wheezing illness in infancy is associated with medically attended third year wheezing in low risk infants: results of a healthy birth cohort study. Immun Inflamm Dis. 2015 Aug 27;3(4):398-405. doi: 10.1002/iid3.77. PMID: 26734461; PMCID: PMC4693725.
13. Sun H, Li S, Yan Y, Chen Z, Wang Y, Hao C, Ji W. Associations between patient clinical characteristics and the presence of cytomegalovirus DNA in the bronchoalveolar lavage fluid of children with recurrent wheezing. BMC Infect Dis. 2018 Sep 10;18(1):458. doi: 10.1186/s12879-018-3345-9. PMID: 30200894; PMCID: PMC6131915.
14. Coverstone AM, Wilson B, Burgdorf D, Schechtman KB, Storch GA, Holtzman MJ, Castro M, Bacharier LB, Sumino K. Recurrent wheezing in children following human metapneumovirus infection. J Allergy Clin Immunol. 2018 Jul;142(1):297-301.e2. doi: 10.1016/j.jaci.2018.02.008. Epub 2018 Mar 2. PMID: 29481834; PMCID: PMC7773053.
15. Leino A, Lukkarinen M, Turunen R, Vuorinen T, Söderlund-Venermo M, Vahlberg T, Camargo CA Jr, Bochkov YA, Gern JE, Jartti T. Pulmonary function and bronchial reactivity 4 years after the first virus-induced wheezing. Allergy. 2019 Mar;74(3):518-526. doi: 10.1111/all.13593. Epub 2018 Oct 8. PMID: 30144084; PMCID: PMC6387855.
16. Robinson PFM, Pattaroni C, Cook J, Gregory L, Alonso AM, Fleming LJ, Lloyd CM, Bush A, Marsland BJ, Saglani S. Lower airway microbiota associates with inflammatory phenotype in severe preschool wheeze. J Allergy Clin Immunol. 2019 Apr;143(4):1607-1610.e3. doi: 10.1016/j.jaci.2018.12.985. Epub 2018 Dec 21. PMID: 30579850.
17. Liu L, Pan Y, Zhu Y, Song Y, Su X, Yang L, Li M. Association between rhinovirus wheezing illness and the development of childhood asthma: a meta-analysis. BMJ Open. 2017 Apr 3;7(4):e013034. doi: 10.1136/bmjopen-2016-013034. PMID: 28373249; PMCID: PMC5387933.

Question 2. What is the role of atopy in the pathogenesis of preschool wheezing?

Search strategy:

((((child, preschool[MeSH Terms]) OR (toddler*)) AND (((respiratory sound[MeSH Terms]) OR (wheez*)) OR (asthma[MeSH Terms:NoExp]))) AND ((((((((inflammation[MeSH Terms]) OR (cytokine*)) OR (interleukine*)) OR (neutrophil*)) OR (mast cell*)) OR (eosinophil*)) OR (t cell*)) OR (innate lymphoid cell*))) AND ((((allergy[MeSH Terms]) OR (atopy)) OR (eosinophil*)) OR (allergen*)) AND ((english[Filter]) AND (2008:2021[pdat]))

What is the role of atopy in the pathogenesis of preschool wheezing?

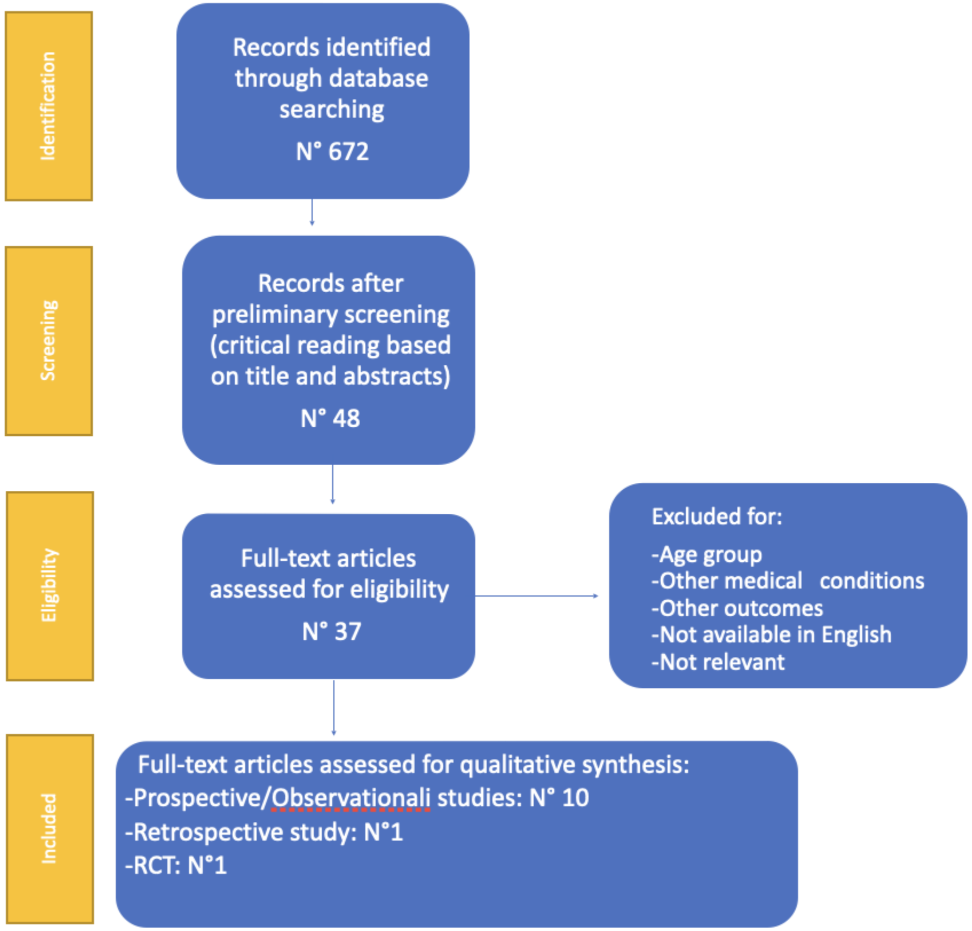

Modified from: Moher D, Liberati A, Tetzlaff J, Altman DG, The PRISMA Group (2009). Preferred Reporting Items for Systematic Reviews and Meta-Analyses: The PRISMA Statement. PLoS Med 6(7): e1000097

Title of the study, first author, year [ref]	Type of study	Study design	Population	N of patients, Age	Experiments/mechanisms assessed	Outcome (primary and secondary)	Results	Relative Risk (RR) or odds ratio (OR) + Confidence Interval (CI)
Lack of eosinophilia can predict remission in wheezy infants?<br><br>Just J, 2008<br><br>[1]	Prospective cohort study	To determine the critical thresholds of common biological markers of atopy in wheezy infants associated with persistence of wheezing into childhood and secondly to rank these biological markers together with clinical parameters	Infants were referred to the Paediatric Pulmonology and Allergy Center in Paris by their primary care physician because of recurrent wheezing (at least three episodes) before 30 months of age	219<br><br><30 months followed up prospectively until the age of 6 years	ISAAC questionnaire, blood test, Severity score for wheezy infants	Determine predictive factors for persistent wheezing at 6 years of age (60 of 219 children)	Children with persistent wheezing at age 6: vs Remission of wheezing at age 6	
						Gender (male)	72% (43) vs 69% (109) p=0.085	
						Paternal asthma	25% (15) vs 13% (21) p=0.032	
						Maternal asthma	18% (11) vs 16% (26) p=0.434	
						Paternal atopic dermatitis	3% (2) vs 8% (13) p=0.168	
						Maternal atopic dermatitis	15% (9) vs 10% (16) p=0.213	
						Paternal allergic rhinitis	12% (7) vs 7% (12) p=0.238	
						Maternal allergic rhinitis	13% (8) vs 11% (18) p=0.420	
						Severe infant wheezing	38% (23) vs 28% (44) p=0.088	
						Infant atopic dermatitis	37% (22) vs 28% (44) p=0.130	

						Eosinophilia	32% (19) vs 9% (15) p<0.001	
						Elevated IgE	42% (25) vs 26% (42) p=0.023	
						Allergic sensitization	42% (25) vs 18% (28) p<0.001	
Presence of Eosinophils in Nasal Secretion during Acute Respiratory Tract Infection in Young Children Predicts Subsequent Wheezing within Two Months<br><br>Shinohara M, 2008<br><br>[2]	Prospective cohort study	To examine the association between the presence of nasal eosinophils during RTI and subsequent wheezing in young children	A total of 35 young who visited outpatient clinic in Kochi, Japan, between April and July 2004, and whose chief complaint was rhinorrhea were enrolled in this prospective cohort study.	35 (6-33 months)	Questionnaire, nasal secretion samples evaluated for the presence or absence of eosinophils by examining 5 high power fields (HPFs)	Risk of wheezing during the subsequent 2 month		
						Allergic diseases in the subjects	7 of 14 (50%) adjusted OR 3.919, p=0.302	(0.293-52.414)
						Family history of allergic diseases	5 of 16 (31%) adjusted OR 0.267, p=0.222	(0.032-2.218)
						Wheezing at study entry	8 of 14 (57%) adjusted OR 20.342, p=0.024	(1.480-279.619)
						Eosinophils in nasal secretions	8 of 16 (50%) adjusted OR 27.618, p=0.01	(1.859-410.201)
						Risk of wheezing during the subsequent 12 months		

						Allergic diseases in the subjects	8 of 14 (57%) adjusted OR 5.128 p=0.095	(0.751-35.020)
						Family history of allergic diseases	5 of 16 (31%) adjusted OR 0.296 p=0.161	(0.060-1.458)
						Wheezing at study entry	8 of 14 (57%) adjusted OR 2.488 p=0.304	(0.438-14.127)
						Eosinophils in nasal secretions	8 of 16 (50%) adjusted OR 4.099 p=0.161	(0.577-29.500)
Allergic sensitization is associated with rhinovirus-, but not other virus-, induced wheezing in children<br><br>Jartti T, 2010<br><br>[3]	Observational study	To evaluate the association between IgE sensitization and viral infection in children with acute wheezing necessitating hospitalization	Children enrolled at Department of Pediatrics, Turku University Hospital from September 2000 to May, 2002	247 (median age 1.6 years, range 0.25-15)	On admission , a nasopharyngeal aspirate sample Blood samples on admission and 2–3 weeks later. Virus antigen detection and virus culture	Atopic characteristics and sole rhinovirus infection	Allergen specific IgE sentization: OR 4.59 (p<0.05)	(1.78, 11.8)
							Aeroallergen sentization: OR 4.18 (p<0.05)	
							Total IgE level: OR: 2.06 (p<0.05)	
							Food allergen sensitization: OR 2.02 (p<0.05)	

							Nasal eosinophil count OR: 1.52 ($p<0.05$)	
						Atopic characteristics and sole respiratory syncytial virus infection	nitric oxide level OR:0.028 ($p<0.05$)	
							blood eosinophil count OR: 0.046 ($p<0.05$)	
							number of wheezing episodes OR: 0.095 ($p<0.05$)	
							nasal eosinophil count OR: 0.13 ($p<0.05$)	
							Total IgE level OR: 0.37 ($p<0.05$)	
						Atopic characteristics and sole enterovirus infection	Blood eosinophil OR 3.52 ($p<0.05$)	
							Nasal eosinophil count OR 1.96 ($p<0.05$)	

							Use of inhaled corticosteroid at study entry OR 2.73 (p<0.05)	
						Atopic characteristics and other viral etiology	No significant associations were found between atopic characteristics and these groups	
Does airway allergic inflammation pre-exist before late onset wheeze in children?<br><br>Thavagnanam S, 2010<br><br>[4]	Observational study	Comparison between children with late onset wheeze (9 LOCW) and no wheeze (45 NW) to determine whether lower airway allergic inflammation pre-exist in late onset childhood wheeze	Children below 5 years who had bronchoalveolar lavage (BAL), evaluated at least 7 years following the initial BAL	54 (median age 3.2, range 1-4.8 years)	BAL, modified ISAAC questionnaire	Blood white cell count	Lower in LOCW than NW (median 6.04x10^5, IQR 5.4-7.5 against 8.40x10^5, IQR 6.9-10.5, p=0.03)	
						Blood eosinophils %	No difference (p=0.27)	
						BAL eosinophils count	Increased in LOCW (median 1.55, IQR 0.33-3.92) compared to NW children (median 0.10 IQR 0.0-0.3 p=0.01)	

						BAL other cell count	No difference (Neutrophils p=0.71, macrophages p=0.79, lymphocytes p=0.40, mast cell p= 0.34, epithelial cell p=0.77)	
						BAL cytokine	No difference	
Asthma-predictive-index, bronchial-challenge, sputum eosinophils in acutely wheezing pre-schoolers.<br><br>Ater D, 2014<br><br>[5]	Prospective controlled trial	To test whether wheezing pre-schoolers presenting to the ED are different from the above in three different domains defining asthma: the atopic characteristics based on stringent asthma predictive index (S-API), the characteristics of bronchial hyper-responsiveness	Preschool children presenting to the ED with acute wheezing recruited during January 2009 to January 2011 compared to healthy pre-schoolers (n = 109)	41 pre-schoolers (age 31.9 ± 17.4 months, range; 1-6 years)	S-API<br>Bronchial challenge tests (BCT)-(methacholine and adenosine)<br>Induced sputum analysis	Primary: S-API	Significantly positive more in wheezing pre-schoolers than in community control group: 20/41 (48.7%) versus 15/109 (13.7%, P < 0.001)	
						Secondary		

		(BHR), and airway inflammation.				BCTs characteristics	- All methacholine-BCTs-30/30 (100%) were positive compared with 13/14 (92.8%) in the ambulatory control group (P = 0.32).<br>- 23/27 (85.2%) were adenosine-BCT positive versus 3/17 (17.5%) in the ambulatory control group (P < 0.001).	
						eosinophils in IS	9/18 (50.0%) showed eosinophilia in the IS	
Hyper-responsive T-cell cytokine profile in	Observational case-control study	To study and compare the influence of	195 eligible subjects of a birth cohort of 253	195	Cord blood samples,	T-cell cytokine responses		

association with development of early childhood wheeze but not eczema at 2 years<br><br>Quah PL, 2014<br><br>[6]		intrinsic T-cell cytokine responses on the development of wheezing and eczema in the first 2 years of life in a birth cohort of at risk (first degree family with atopic disease) infants.	subjects enrolled at the National University Hospital of Singapore that were required to have at least one parent with allergic rhinitis, eczema or asthma.The subjects studied were those who developed either wheezing (n = 34) or eczema (n = 29) in the first 2 years of life, and 65 healthy infants served as control.		stimulation of cord blood mononuclear cells and Cytokine detection. Blood samples Prick test	IL2	Higher In wheeze group than control: 3.38 ng/mL (1.78 - 4.91) vs 1.23 ng/mL (0.29 - 2.40) p=0.021, OR 2.44	(1.14-5.22)
							No significant difference between eczema group and control) p= 0.831 OR 1.07	(0.58 - 1.98)
						INF-γ	Higher In wheeze group than control: 2.71 (0.96 - 4.28) vs 0.75 ng/mL (0.33 - 1.86) p=0.050 OR 2.50	(0.97-6.20)

							No significant difference between eczema group and control, p =0.495 OR 1.31	(0.60- 2.88)
						IL5	Higher In wheeze group than control: 0.03 (0.02 - 0.05) vs 0.01 ng/mL (0.01 - 0.03), p= 0.002 OR 16.8	(2.73-103.9)
							No significant difference between eczema group and control, p= 0.826 OR 1.22	(0.20 -7.40)
						IL13	Higher In wheeze group than control: 4,461 (3,448-7,631) vs 3,919 pg/ml (2,698-4,837), p= 0.047 OR 1.02	(1.00-1.04)

							No significant difference between eczema group and control p= 0.078 OR 0.25	(0.05-1.17)
Risk factors for recurrent wheezing in infants: a case-control study<br><br>De Sousa RB, 2016<br><br>[7]	Observational case-control study	To evaluate the association between recurrent wheezing and atopy, the Asthma Predictive Index, exposure to risk factors, and total serum IgE levels as potential factors to predict recurrent wheezing	Infants treated at the outpatient clinic of the Hospital das Clínicas of the Universidade Federal de Pernambuco (HC/UFPE) from November 2011 to March 2013. Recurrent wheezing infants composed the case group. Infants of the same age group from the Pediatrics and Childcare Clinic, with no history of wheezing, constituted the control group.	113 aged 6-24 months: 65 infants with recurrent wheezing (mean age of 14.8 months) and 48 healthy infants (mean age of 15.2 months)	EISL questionnaire<br>Asthma Predictive Index<br>Skin prick tests<br>Blood samples	Sensitization to allergens	Higher in wheezy group than control OR = 12.45; p=0.029	(1.28–19.11)
						Positive API	Positive for 81.5% of recurrent wheezing infants and 44.8% of non-wheezers OR = 5.57, p<0.001	(2.23–7.96)
						Exposure to smoke	Higher in wheezy group than control OR = 2.63, p=0.030	(1.09–6.30)
						Total serum IgE ≥100 UI/mL	48% of recurrent wheezing infants and 30.0% of control (p = 0.059)	

						Peripheral blood eosinophilia ≥4.0%	46.2% of recurrent wheezers and in 18.8% of control (p = 0.002)	

Individualized therapy for persistent asthma in young children<br><br>Fitzpatrick AM, 2016<br><br>[8]	Multicenter, randomized, double-blind, double-dummy clinical trial<br><br>(Individualized Therapy for Asthma in Toddlers study)	To evalutate different phenotypic presentations in young children with asthma that might contribute to differential responses to asthma controller medications.	Children necessitating treatment with daily controller (Step 2) therapy.	N = 300<br><br>From 12 to 59 months of age	Participants completed a 2- to 8-week run-in period followed by 3 crossover periods with daily inhaled corticosteroids (ICSs), daily leukotriene receptor antagonists, and as-needed ICS treatment coadministered with albuterol	The primary outcome was differential response to asthma medication based on a composite measure of asthma control. The primary analysis involved 2 stages: determination of differential response and assessment of whether 3 prespecified features (aeroallergen sensitization, previous exacerbations, and sex) predicted a differential response.	Seventy-four percent (170/230) of children with analyzable data had a differential response to the 3 treatment strategies. Within differential responders, the probability of best response was highest for a daily ICS and was predicted by aeroallergen sensitization but not exacerbation history or sex.<br>The probability of best response to daily ICS was further increased in children with both aeroallergen sensitization and blood eosinophil counts of 300/μL or greater. In these children daily ICS use was associated with more asthma control days and fewer exacerbations compared with the other treatments.	In young children with asthma necessitating Step 2 treatment, phenotyping with aeroallergen sensitization and blood eosinophil counts is useful for guiding treatment selection and identifies children with a high exacerbation probability for whom treatment with a daily ICS is beneficial despite possible risks of growth suppression.

Neutrophilic Steroid-Refractory Recurrent Wheeze and Eosinophilic Steroid-Refractory Asthma in Children<br><br>Guiddir T, 2017<br><br>[9]	Prospective cross-sectional study	To characterize phenotypes of severe recurrent wheeze and severe asthma during childhood in terms of triggers (allergic or not), involved cells (eosinophil or neutrophil), and corticoid responsiveness.	Children with moderate-to-severe asthma and preschool children with moderate-to-severe recurrent wheeze were enrolled prospectively from 2011 to 2015 from SAMP (Severe Asthma Molecular Phenotype), a part of the TAP (Trousseau Asthma Program) cohort, at Trousseau Hospital, Paris.	350 children aged from 1 to 15 years at the time of exploration. Preschool recurrent wheeze children (n= 217 mean age 33.9 months) and Asthma children (n=133 mean age 138 months)	Cluster analysis with 34 variables Skin prick test Blood samples Flexible bronchos copy and microbiol ogical cultures of BAL	Cluster analysis reveal 3 clusters of children with shared phenotypic characteristics. Cluster 1: Neutrophilic steroid-refractory recurrent wheeze phenotype (n =138)<br>Cluster 2: Severe recurrent wheeze with sensitization to a single aeroallergen (n= 104)<br>Cluster 3: Eosinophilic steroid-refractory asthma phenotype (n=108)		

Remodelling and inflammation in preschoolers with severe recurrent wheeze and asthma outcome at school age<br><br>Lezmi G, 2018<br><br>[10]	Observational study	To assess the influence of airway remodelling and inflammation in preschoolers with severe recurrent wheeze on asthma	Preschool children with severe recurrent wheeze from the Severe Preschool Asthma Survey and Monitoring (SPASM) cohort initially investigated with bronchial biopsies were re-assessed for asthma symptoms and lung function at school age	49 (median age: 38.4 months)<br>36 of 49 (73.5%) were assessed at 10.9 years (26 – 72.2% had persistent asthma)	Bronchial biopsies	Submucosal eosinophil counts	Higher in children with severe exacerbations at school age than in those without (16/0.1 mm2 [11.2-30.4] vs 8/0.1 mm2 [2.4-17.6], p = 0.02) and correlated with the number of severe exacerbations (p = 0.04, r = 0.35)	

						Submucosal neutrophil counts	No difference between children with severe exacerbations at school age than in those without but correlate with post-bronchodilator FEV1/FVC ($p < 0.01$, $r = 0.47$), and post bronchodilator FEF25-75% predicted ($p = 0.02$, $r = 0.43$), but not with post-bronchodilator FEV1% predicted ($p = 0.78$, $r = 0.05$)	

						Markers of remodelling	At baseline similar between the children with persistent asthma and those with no asthma at school age. The ASM (airway smooth muscle) area at baseline correlated with post-bronchodilator FEV1/FVC at school age ($p < .01$, $r = 0.51$), but not with post-bronchodilator FEV1 or FEF25-75% predicted. The number of vessels at baseline negatively correlated with post-bronchodilator FEV1% predicted, and FEV1/FVC at school age ($p = 0.03$, $r = 0.43$; $P = 0.05$, $r = 0.41$; respectively), as well as post-bronchodilator FEF25-75% predicted ($p = 0.01$, $r = 0.46$)	

Phenotypes of the inflammatory cells in the induced sputum from young children or infants with recurrent wheezing<br><br>Guo Y, 2019<br><br>[11]	Retrospective study	To identify inflammatory cell types by phenotypic analysis of the inflammatory cells in the induced sputum	Young children or infants with recurrent wheezing were enrolled into this study at the Tianjin Children's Hospital between January 2009 and December 2013. All children were followed up for 2 years and were assigned into mild/moderate groups (326) and severe group (602) by clinical symptom scores	1232 (age from 6 months to 3 years old)	Sputum induction Quantification of serum total lgE, inhaled allergen lgE, food allergen lgG, blood eosinophil, and neutrophil	Sputum eosinophil	gradually increased in the order of mild (2.01 ± 2.04), moderate (3.65 ± 2.43), and severe (4.76 ± 2.49) (*p* < 0.001).	
						Sputum macrophage	No statistical difference between three groups (p=0.347)	
						Sputum epithelial cell count	Increased in moderate (13.04 ± 7.12) and severe (14.78 ± 6.99) groups compared to that in mild group (12.79 ± 7.16) (p = 0.01)	
						Sputum lymphocyte	No statistical difference between three groups (p=0.702)	

						Sputum other cells	No statistical difference between three groups (p=0.730)	
						Blood eosinophil	No statistical difference between three groups (p=0.146)	
						Blood neutrophil	significantly different between the three groups (mild: 64.13 ± 16.94; moderate: 59.26 ± 18.5; severe: 57.25 ± 16.6) (p< 0.01)	

						Eosinophil-derived neurotoxin (EDN)	Highest in the eosinophil type group (112.6 ± 41.2 μg /1) compared to that in the mixed granulocytic type group (104.8 ± 39.4 μg/1), neutrophil type group (88.2 ± 36.6μg/1), or paucigranulocytic type group (60.9 ± 34.6 μg/1) but there was no significant difference between any two groups (p>0.05)	
Recurrent Severe Preschool Wheeze: From Prespecified Diagnostic Labels to Underlying	Observational study	To investigate lower airway inflammation and infection in preschool children with different clinical	Children aged 12–72 months undergoing clinically indicated, elective bronchoscopy for	136 (1-5 years)	Bronchoscopy and BAL and differential cell count Periphera	Peripheral blood count		
						Eosinophil %	RSW: 4.1 (0–16) vs NWRD: 2.0 (1–10) p=0.004	

Endotypes<br><br>Robinson PFM, 2021<br><br>[12]		diagnoses undergoing elective bronchoscopy and BAL	severe respiratory symptoms at the Royal Brompton Hospital (London, United Kingdom): 105 with recurrent severe wheeze [RSW]; 31 with nonwheezing respiratory disease [NWRD]). Children with RSW were assigned as having episodic viral wheeze (EVW) or multiple-trigger wheeze (MTW)		l blood sample	Neutrophil %	RSW: 41.7 (14–81) vs NWRD: 42.6 (23–76) p=0.71	
						Lymphocyte %	RSW: 46.3 (11–74) vs NWRD: 47.4 (19–84) p=0.7	
						Monocyte %	RSW: 7.0 (2–17) vs NWRD: 7.2 (5–17) p=0.61	
						IgE IU/ml	RSW: 21 (0–1575) vs NWRD: 10 (1–1,000) p=0.09	
						BAL samples		
						Eosinophil %	RSW: 1.3 (0–16) vs NWRD: 0.5 (0–8) p=0.09	
						Neutrophil %	RSW: 7.5 (0–82) vs NWRD: 16.9 (1–82) p=0.09	
						Lymphocyte %	RSW: 11.2 (2–59 [82]) vs NWRD: 9.1 (0.7–38) p=0.44	

						Macrophage %	RSW: 71.7 (6–94 [82]) vs NWRD: 65.4 (10–93) p=0.35	
						Positive bacteriology result n, (%)	RSW: 45/102 (44) vs NWRD: 14/31 (45) p=0.99	
						Positive viral PCR result, n, (%)	RSW: 44/99 (44) vs NWRD: 16/29 (55) p=0.4	

References

1. Just J, Nicoloyanis N, Chauvin M, Pribil C, Grimfeld A, Duru G. Lack of eosinophilia can predict remission in wheezy infants? Clin Exp Allergy. 2008 May;38(5):767-73. doi: 10.1111/j.1365-2222.2008.02966.x. Epub 2008 Mar 4. PMID: 18325032.
2. Shinohara M, Wakiguchi H, Saito H, Matsumoto K. Presence of eosinophils in nasal secretion during acute respiratory tract infection in young children predicts subsequent wheezing within two months. Allergol Int. 2008 Dec;57(4):359-65. doi: 10.2332/allergolint.O-08-544. Epub 2008 Dec 1. PMID: 18797177.
3. Jartti T, Kuusipalo H, Vuorinen T, Söderlund-Venermo M, Allander T, Waris M, Hartiala J, Ruuskanen O. Allergic sensitization is associated with rhinovirus-, but not other virus-, induced wheezing in children. Pediatr Allergy Immunol. 2010 Nov;21(7):1008-14. doi: 10.1111/j.1399-3038.2010.01059.x. PMID: 20977499; PMCID: PMC7168081.
4. Thavagnanam S, Williamson G, Ennis M, Heaney LG, Shields MD. Does airway allergic inflammation pre-exist before late onset wheeze in children? Pediatr Allergy Immunol. 2010 Nov;21(7):1002-7. doi: 10.1111/j.1399-3038.2010.01052.x. PMID: 20573036.
5. Ater D, Bar BE, Fireman N, Fireman E, Shai H, Tasher D, Dalal I, Mandelberg A. Asthma-predictive-index, bronchial-challenge, sputum eosinophils in acutely wheezing preschoolers. Pediatr Pulmonol. 2014 Oct;49(10):952-9. doi: 10.1002/ppul.22926. Epub 2013 Oct 25. PMID: 24166822.
6. Quah PL, Huang CH, Shek LP, Chua KY, Lee BW, Kuo IC. Hyper-responsive T-cell cytokine profile in association with development of early childhood wheeze but not eczema at 2 years. Asian Pac J Allergy Immunol. 2014 Mar;32(1):84-92. doi: 10.12932/AP0355.32.1.2014. PMID: 24641295.
7. De Sousa RB, Medeiros D, Sarinho E, Rizzo JÂ, Silva AR, Bianca AC. Risk factors for recurrent wheezing in infants: a case-control study. Rev Saude Publica. 2016;50:15. doi: 10.1590/S1518-8787.2016050005100. Epub 2016 May 3. PMID: 27143615; PMCID: PMC4904490.
8. Fitzpatrick AM, Jackson DJ, Mauger DT, Boehmer SJ, Phipatanakul W, Sheehan WJ, Moy JN, Paul IM, Bacharier LB, Cabana MD, Covar R, Holguin F, Lemanske RF Jr, Martinez FD, Pongracic JA, Beigelman A, Baxi SN, Benson M, Blake K, Chmiel JF, Daines CL, Daines MO, Gaffin JM, Gentile DA, Gower WA, Israel E, Kumar HV, Lang JE, Lazarus SC, Lima JJ, Ly N, Marbin J, Morgan W, Myers RE, Olin JT, Peters SP, Raissy HH, Robison RG, Ross K, Sorkness CA, Thyne SM, Szefler SJ; NIH/NHLBI AsthmaNet. Individualized therapy for persistent asthma in young children. J Allergy Clin Immunol. 2016 Dec;138(6):1608-1618.e12. doi: 10.1016/j.jaci.2016.09.028. Epub 2016 Oct 21. PMID: 27777180; PMCID: PMC5148729.
9. Guiddir T, Saint-Pierre P, Purenne-Denis E, Lambert N, Laoudi Y, Couderc R, Gouvis-Echraghi R, Amat F, Just J. Neutrophilic Steroid-Refractory Recurrent Wheeze and Eosinophilic Steroid-Refractory Asthma in Children. J Allergy Clin Immunol Pract. 2017 Sep-Oct;5(5):1351-1361.e2. doi: 10.1016/j.jaip.2017.02.003. Epub 2017 Mar 28. PMID: 28363401.
10. Lezmi G, Deschildre A, Abou Taam R, Fayon M, Blanchon S, Troussier F, Mallinger P, Mahut B, Gosset P, de Blic J. Remodelling and inflammation in preschoolers with severe recurrent wheeze and asthma outcome at school age. Clin Exp Allergy. 2018 Jul;48(7):806-813. doi: 10.1111/cea.13143. Epub 2018 May 7. PMID: 29603800.
11. Guo Y, Zou Y, Zhai J, Li J, Liu J, Ma C, Jin X, Zhao L. Phenotypes of the inflammatory cells in the induced sputum from young children or infants with recurrent wheezing. Pediatr Res. 2019 Mar;85(4):489-493. doi: 10.1038/s41390-018-0268-5. Epub 2019 Jan 15. PMID: 30644443.
12. Robinson PFM, Fontanella S, Ananth S, Martin Alonso A, Cook J, Kaya-de Vries D, Polo Silveira L, Gregory L, Lloyd C, Fleming L, Bush A, Custovic A, Saglani S. Recurrent Severe Preschool Wheeze: From Prespecified Diagnostic Labels to Underlying Endotypes. Am J Respir Crit Care Med. 2021 Sep 1;204(5):523-535. doi: 10.1164/rccm.202009-3696OC. PMID: 33961755; PMCID: PMC8491264.

SECTION 2 - RISK FACTORS FOR WHEEZE DEVELOPMENT

When considering risk factors for preschool development, different search strategies have been created according to the PICO question:

P (*patient*) = Wheezy preschool children

I (*intervention*) = Exposition to (or presence of) risk factors

- **I1** = Allergy
- **I2** = Previous bronchiolitis
- **I3** = Prematurity
- **I4** = Family history
- **I5** = Other risk factors (pollution, other infections, genetics)

C (*comparison*) = Preschool children not exposed to the above-mentioned risk factors

O1 (*outcome*) = Onset of preschool wheezing

O2 (*outcome*) = Evolution of preschool wheezing

Six different search queries were performed, 4 specifically addressing allergy, bronchiolitis, prematurity and family history and 2 addressing overall risk factors AND onset OR evolution of preschool wheezing.

Does the presence of risk factors such as family history, allergy, prematurity or previous bronchiolitis influence the onset and the evolution of preschool wheezing?

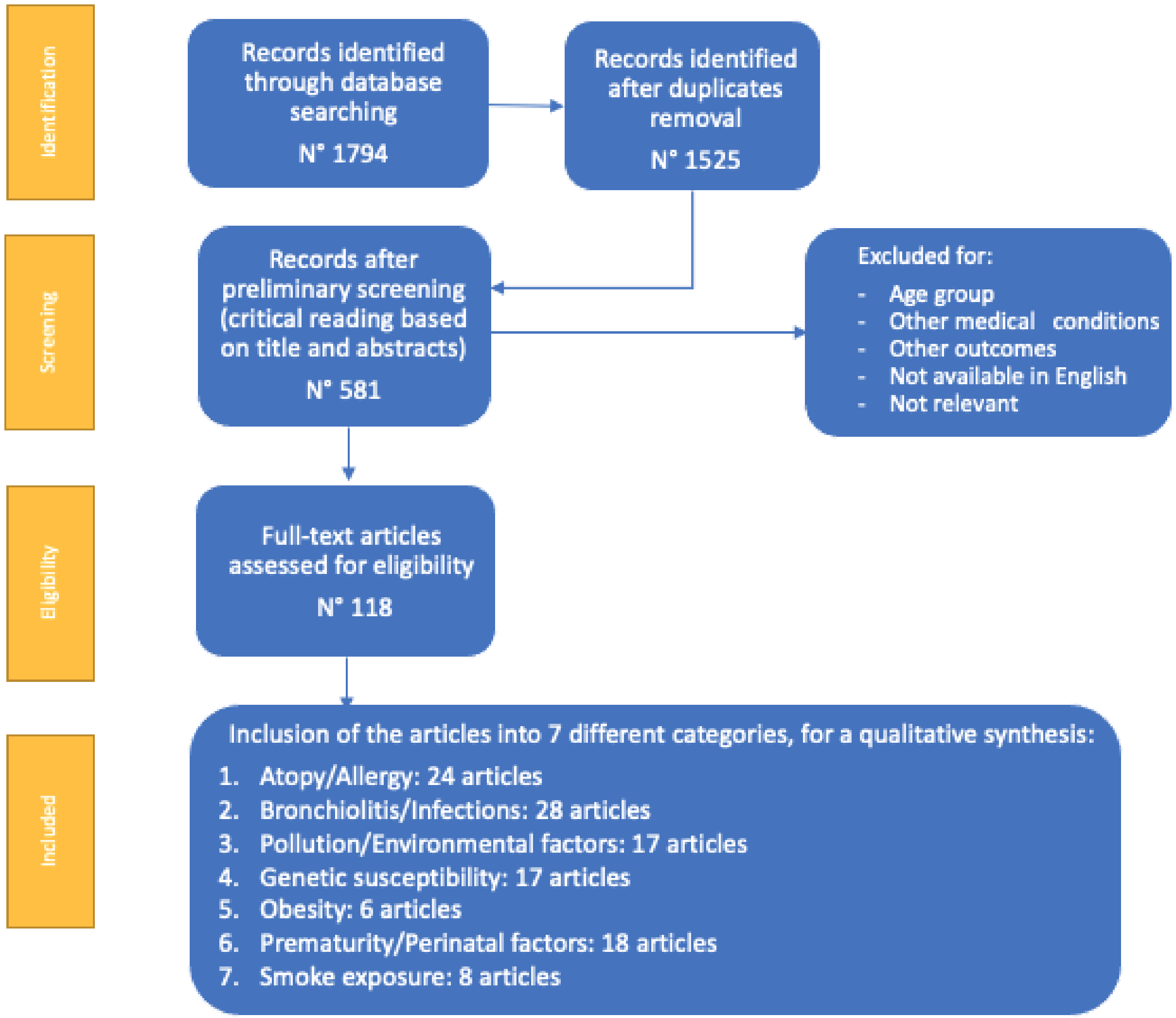

Modified from: Moher D, Liberati A, Tetzlaff J, Altman DG, The PRISMA Group (2009).
Preferred Reporting Items for Systematic Reviews and Meta-Analyses: The PRISMA Statement. PLoS Med 6(7): e1000097

Question 1. Does the presence of risk factors such as allergy/atopy influence the onset and the evolution of preschool wheezing?

Search strategies:

(“child, preschool”[MeSH Terms] OR “toddler*”[All Fields]) AND (“respiratory sounds”[MeSH Terms] OR “wheez*”[All Fields] OR “Asthma”[MeSH Terms:noexp]) AND (“allergy and immunology”[MeSH Terms] OR “atopy”[All Fields] OR “inhaled allergen*”[All Fields] OR “food allergy”[All Fields]) AND “45nglish”[Language] AND 2008/01/01:2021/12/31[Date – Publication]

("child, preschool"[MeSH Terms] OR "toddler*"[All Fields]) AND ("respiratory sounds"[MeSH Terms] OR "wheez*"[All Fields] OR "Asthma"[MeSH Terms:noexp]) AND "Risk Factors"[MeSH Terms] AND ("Age of Onset"[MeSH Terms] OR "Onset"[All Fields]) AND "english"[Language] AND 2008/01/01:2021/12/31[Date - Publication]

("child, preschool"[MeSH Terms] OR "toddler*"[All Fields]) AND ("respiratory sounds"[MeSH Terms] OR "wheez*"[All Fields] OR "Asthma"[MeSH Terms:noexp]) AND "Risk Factors"[MeSH Terms] AND ("disease progression"[MeSH Terms] OR "disease evolution"[All Fields] OR "evolution"[All Fields]) AND "english"[Language] AND 2008/01/01:2021/12/31[Date - Publication]

Title of the study, first author, year	Type of study	Objective	Population	N of patients, Age	Experiments/mechanisms assessed	Relative risk (RR) or odds ratio (OR) + confidence interval (CI 95%)	Results
Associations of wheezing phenotypes in the first 6 years of life with atopy, lung function and airway responsiveness in mid-childhood.<br><br>Henderson J, 2008<br><br>[1]	Population-based birth cohort study	To find an improved characterization of wheezing phenotypes that could lead to the identification of environmental influences on the development of asthma and airway diseases in predisposed individuals.	Children enrolled through ALSPAC, a study that recruited 14 541 pregnant women resident in Avon, UK with expected dates of delivery 1 April 1991 to 31 December 1992.	6265 children, followed from birth to 7 years	At 6, 18, 30, 42, 54, 69 and 81 months after birth, study mothers were sent a self-completion questionnaire about the health of their child. They were asked to report the occurrence of 15 common symptoms, including wheezing.<br><br>The atopic status of the children was determined at 7–8 years of age by skin prick test responses to a panel of up to 12 common allergens.<br><br>At 8–9 years of age, lung function was measured by spirometry.	- The strongest associations with atopy and airway responsiveness were found for intermediate onset (18 months) wheezing (OR for atopy 8.36, 95% CI 5.2 to 13.4; mean difference in dose response to methacholine 1.76, 95% CI 1.41 to 2.12 %FEV1 per mmol, compared with infrequent/never wheeze phenotype).<br><br>- Late onset wheezing (after 42 months) was also associated with atopy (OR 6.6, 95% CI 4.7 to 9.4) and airway responsiveness (mean difference 1.61, 95% CI 1.37 to 1.85 %FEV1 per mmol).	The wheezing phenotypes most strongly associated with atopy and airway responsiveness were characterized by onset after age 18 months. This has potential implications for the timing of environmental influences on the initiation of atopic wheezing in early childhood.

Effects of dog ownership in early childhood on immune development and atopic diseases.<br><br>Bufford JD, 2008<br><br>[2]	Prospective birth cohort study	To determine whether the effects of pet exposure on immune development and atopy in early childhood can be explained by alterations in exposure to innate immune stimuli in settled dust.	Newborns enrolled from November 1998 through May 2000 in the Childhood Origins of ASThma (COAST) study.	275 three years-old children	Two hundred and seventy-five children at increased risk of developing allergic diseases were evaluated to age 3 years for pet ownership, blood cell cytokine responses, and atopy.<br><br>Can f 1, Fel d 1, endotoxin, ergosterol, and muramic acid were measured in settled dust from 101 homes.	Dog exposure at birth was associated with decreased atopic dermatitis (AD) (12% vs. 27%; P=0.004) and wheezing (19% vs. 36%; P=0.005) in year 3.<br><br>Can f 1 levels in bedroom dust were positively associated with IL-10 (r=0.26; P=0.01), IL-5 (r=0.34, P<0.001), and IL-13 (r=0.28; P=0.004) responses at age 1, and IL-5 (r=0.24; P=0.022) and IL-13 (r=0.25; P=0.015) responses at age 3.<br><br>In contrast, endotoxin was associated with IFN-gamma (r=0.31; P=0.002) and IL-13 (r=0.27; P=0.01) responses at age 3 but not at age 1, and similar relationships were present for muramic acid.	Exposure to dogs in infancy, and especially around the time of birth, is associated with changes in immune development and reductions in wheezing and atopy.

Anti-cockroach and anti-mouse IgE are associated with early wheeze and atopy in an inner-city birth cohort.<br><br>Donohue KM, 2008<br><br>[3]	Longitudinal birth-cohort study	To verify if the presence of anti-cockroach and anti-mouse IgE by age 3 years in inner-city children might be associated with respiratory and atopic symptoms.	A birth cohort selected independently of family history of asthma and atopy. African American and Dominican women aged 18 to 35 years who had lived in Northern Manhattan or the South Bronx for at least 1 year were recruited during pregnancy.	404 children, followed from birth until 3 years old.	Detailed questionnaires were administered to the mother prenatally and every 3 months until the child reached age 2 years and subsequently every 6 months through age 3 years.<br><br>Dust samples were collected prenatally and postnatally from the kitchens and beds.<br><br>Serum samples were collected at age 2 years (n = 344) and 3 years (n = 322).	The odds of early wheeze were significantly higher among children who had IgE to cockroach (odds ratio [OR], 3.3; 95% CI, 1.8-6.2), mouse (OR, 4.6; 95% CI, 2.3-9.0), or both (OR, 9.7; 95% CI, 3.4-27.3).<br><br>The odds of rhinitis or atopic dermatitis were also higher among children with IgE to cockroach, mouse, or both.<br><br>Higher IgE class to cockroach and mouse was associated with wheeze and atopic dermatitis (tests for trend, $P < .002$).	Children age 2 to 3 years who have anti-cockroach and anti-mouse IgE are at increased risk of wheeze and atopy.

Risk factors associated with transient wheezing in young children.<br><br>Simon MR, 2008<br><br>[4]	Birth cohort study	A prospective analysis of a relatively large population-based<br><br>birth cohort to define the relationship of atopic risk factors and sensitization in association with transient wheezing in young children.	Childhood Allergy Study population	372 children<br>- 189 females<br>- 183 males	Data were collected by questionnaires at ages 1, 2, 3 and 4.<br>Information collected on:<br>- presence of transient wheezing,<br>- atopy risk factor,<br>- exposition to pets,<br>- early antibiotic administration,<br>- persistent breast-feeding,<br>- Daycare attendance.<br><br>Between ages 6 and 7 years the children were clinically evaluated for circulating IgE to Dermatophagoides farinae, dog, cat, short ragweed, and timothy grass antigens by skin prick tests.	A. 175 (47.0%) of the 372 subjects had never wheezed and 128 (34.4%) subjects had wheezed in the previous year at ages 1, 2, and/or 4 years, but not at age 6 years (transient wheezing).<br>B. Boys were more likely to transiently wheeze (RR 1.7; CI, 1.1–2.8; p 0.018; Table 2).<br>C. Early antibiotic use was associated also with an increased risk (RR 1.6; CI, 1.0 –2.6; p 0.048).<br>D. Breastfeeding for 4 or more months was not associated with transient wheezing compared with never having wheezed (RR 0.9; CI, 0.7–1.1; p 0.34). Breastfeeding was further evaluated by assessing transient wheezing in children who were exclusively breast-fed (RR 0.9; CI, 0.7–1.2; p 0.62) and those who received supplemental feedings as well as breast milk (RR 0.7; CI, 0.5–1.1; p 0.082), suggesting some protection from supplementation and breast-feeding.<br>E. There was no association of birth order, parental allergy, pet keeping, fever in the 1st year of life, or day care with transient wheezing.<br><br>Children with transient wheezing were not more	Transient wheezing in young<br>children is not associated with allergy and appears to be more common in<br>boys and among children who received treatment with<br><br>antibiotics in the first 6 months of life.

						likely to be classified as atopic by ELISA (OR 1.2; CI, 0.5–2.7; p 0.66; Table 3), and they were not more likely to have positive skin-prick test results (OR 0.8; CI, 0.4 –1.5; p 0.47; Table 3).	

Impact of innate and environmental factors on wheezing persistence during childhood.<br><br>Just J, 2010<br><br>[5]	Prospective cohort study	The aim of this study was to evaluate the importance of innate and environmental factors associated with occurrence of asthma during childhood in a population of recurrent wheezing infants followed prospectively.	Newborns aged 30 months or less, referred to the Paediatric Pulmonology and Allergy Center in Paris<br><br>because of recurrent wheezing.	219 subjects aged 15 +/- 5 months.	Infants were followed up prospectively until the age of 6 years in order to evaluate wheezing outcome.<br><br>Data were collected using a questionnaire and blood tests.<br><br>The outcome (remission of wheezing) was determined at 6 y.o. using the ISAAC questionnaire, completed by the family.	Identified three risk factors associated with a greater probability of persistence of wheezing:<br>- eosinophilia≥470/mm3 (OR=3.48 [95% CI: 1.38–8.79]),<br>- allergic sensitization (OR=2.23 [95% CI: 1.20–4.12]),<br>- father with asthma (OR = 2.34 [95% CI: 1.06–5.17]).<br><br>Three protective factors associated with a lower risk of persistence of wheezing were:<br>- ≥3 siblings during the first year of life (OR = 0.36 [95% CI: 0.17–0.76]),<br>- breastfeeding for longer than 3 months (OR = 0.43 [95% CI: 0.23–0.82]),<br><br>- pets at home during the first year of life (OR = 0.32 [95% CI: 0.16–0.63]).	This study confirms the role of atopic host factors on wheezing persistence during<br><br>childhood and detected protective environmental factors.

Particular characteristics of allergic symptoms in tropical environments: follow up to 24 months in the FRAAT birth cohort study<br><br>Acevedo N, 2012<br><br>[6]	Cohort study	Investigation of genetic and environmental risk factors for asthma and atopy, considering the effects of prenatal sociodemographic characteristics and environmental exposures at 6, 12 and 24 months and/or recurrent wheezing during the first 24 months	326 mother-infant pairs	- Mothers: 326<br>- Newborns: 326<br>- Child (3-6 months): 286<br>- Child (12 months): 280<br>- Child (24 months): 274<br><br>From 0 to 24 months of age.	Mother-infant pairs included at baseline; collection of biological samples from birth to 24 months for immunological testing, molecular genetics and gene expression analysis. Pre and post-natal information collected using questionnaires.	A. Maternal allergic rhinitis associated with wheezing ever at 6 months (after adjustment for gender) □ [aOR 3.03 (95%CI 1.60-5.74), p = 0.001]<br><br>B. Male gender associated with increased susceptibility of wheezing during the interval 0 to 6 months (independently of maternal rhinitis) □ [aOR 2.09 (95%CI 1.09 - 4.01), p = 0.026].<br><br>C. Maternal asthma associated with **increased risk of wheezing between 7 and 12 months** (after adjustment by maternal age and child gender □[aOR 3.87 (95%CI 1.24-12.1), p = 0.02]<br>Maternal asthma also associated **with wheezing at 24 months** □ [aOR 3.65 (95%CI 1.23-10.8) p = 0.01], after adjustment by maternal age, number of siblings and child birth weight and gender<br>Also associated **with recurrent wheezing** (significant effects after adjustment for maternal age, number of siblings and birth weight) □ [aOR 4.42 (95%CI 1.46-13.4), p = 0.008].<br><br>D. Bronchiolitis associated with wheezing at 6 months □ (aOR 18.3 (95%CI 7.6-44.0), p < 0.0001) and 24 months (aOR 20.6 (95%CI 6.08-70.1), p <0.0001).	At 24 months, the history of allergic symptoms is different to the "atopic march" described in some populations of industrialized countries.<br><br>Although the rates of wheezing were higher than those reported in these countries, cases of eczema were not found.<br><br>Wheezing is the most frequent phenotype during the first 24 months of life and is strongly associated with maternal asthma.

						Also for recurrent wheezing at 24 months (aOR 6.8 (95%CI 3.0-15.5), p < 0.0001), after adjustment for maternal asthma, maternal age, number of siblings and children gender.<br><br>E. Maternal atopy, number of sibling, natural or propane gas exposition, maternal active or passive smoking, poverty, co-existence with pets or poultries no association.	

Evidence for a causal relationship between allergic sensitization and rhinovirus wheezing in early life.<br><br>Jackson DJ, 2012<br><br>[7]	Prospective birth cohort study	To define the developmental relationship between aeroallergen sensitization and virus-induced wheezing.	Children at high risk for asthma and allergic disease based on parental histories of asthma and allergic sensitization were enrolled at birth and followed prospectively in the COAST study	285 children (followed from birth until 6 y.o.) classified in one of four states at each yearly visit:<br>- **State 1**, neither sensitized to aeroallergen(s) or viral wheeze;<br>- **State 2**, viral wheeze only;<br>- **State 3**, sensitized to aeroallergen(s) only;<br><br>- **State 4**, sensitized to aeroallergen(s) and viral wheeze.	The etiology and timing of specific wheezing viral respiratory illnesses during the first 6 years of life were assessed using nasal lavage, culture, and PCR-based viral diagnostic.<br><br>Peripheral blood was drawn on an annual basis, and aeroallergen sensitization was assessed in serum.	**Primary outcome**:<br>Beginning at 1 year of age and continuing throughout the first 6 years of life, allergic sensitization led to an increased risk of wheezing illnesses caused by HRV(HR, 2.3; 95% CI, 1.3–4.0) (Table 1). In contrast, allergic sensitization did not lead to a statistically significant increase in risk of RSV wheezing illnesses (HR, 1.6; 95% CI, 0.87–2.9).<br><br>**Secondary outcome**:<br>Sensitized children were at increased risk of transitioning to HRV wheezing (HR, 2.8; 95% CI, 1.5–5.1) but not RSV wheezing (HR, 0.71; 95% CI, 0.25–2.0). The difference between these ratios was statistically significant (P ¼ 0.02).	Allergic sensitization precedes HRV wheezing and the converse is not true.

The effect of parental allergy on childhood allergic diseases depends on the sex of the child.<br><br>Arshad SH, 2012<br><br>[8]	Birth-cohort study	To investigate parent of origin effect in childhood allergic diseases.	An unselected whole population birth cohort recruited in 1989 to prospectively study the natural history of asthma and allergic conditions.	1,456 children<br>- 1,374 (94%) seen at the age of 1 year,<br>- 1,231 (85%) at 2 years,<br>- 1,214 (83%) at 4 years,	Information on prevalence of asthma, eczema, rhinitis and environmental factors was obtained using validated questionnaires.<br><br>Skin prick tests were carried out at ages 4, 10 and 18 year, and total IgE at 10 and 18 years.<br><br>Parental history of allergic disease was assessed soon after the birth of the child when maternal IgE was also measured.	When stratified for sex of the child, maternal asthma was associated with asthma in girls [PR:1.91 (CI:1.34–2.72), p=0.0003], but not in boys [PR:1.29 (CI:0.85–1.96), p=0.23), while paternal asthma was associated with asthma in boys [PR:1.99 (CI:1.42–2.79), p<0.0001], but not in girls [PR: 1.03 (0.59–1.80) p=0.92).<br><br>Maternal eczema increased the risk of eczema in girls [PR: 1.92 (CI: 1.37–2.68); p=0.0001] only, while paternal eczema did the same for boys (PR: 2.07 (CI:1.32–3.25); P=0.002).	The current study indicates a sex dependent association of parental allergic conditions with childhood allergies; maternal allergy increasing the risk in girls and paternal allergy in boys.

Eczema in early childhood is strongly associated with the development of asthma and rhinitis in a prospective cohort.<br><br>Von Kobyletzki LB, 2012<br><br>[9]	Prospective population-based cohort study	To estimate the association between eczema in early childhood and the onset of asthma and rhinitis later in life in children.	Children were included in the Dampness in Building and Health (DBH) study in the year 2000.	3,124 children aged 1-2 years, followed up 5 years later.	A questionnaire based on an International Study of Asthma and Allergies in Childhood (ISAAC) protocol was given to parents of all children aged 1 to 5 years (DBH-I).<br><br>Follow-up was performed in 2005 in children who were aged 1 to 3 years in 2000 (DBH-III).	Children with eczema had a 3-fold increased odds of developing asthma ([aOR], 3.07; 95% confidence interval (CI) 1.79-5.27), and a nearly 3-fold increased odds of developing rhinitis (aOR, 2.63; 1.85-3.73) at follow-up compared with children without eczema.<br><br>When eczema was divided into subgroups, the odd of developing asthma and rhinitis increased:<br>- moderate to severe eczema (aOR, 3.56; 1.62-7.83 and aOR, 3.87; 2.37-6.33, respectively),<br>- early onset of eczema (aOR, 3.44; 1.94-6.09 and aOR, 4.05; 2.82-5.81; respectively),<br>- persistence of eczema (aOR, 5.16; 2.62-10.18 and aOR, 4.00; 2.53-6.22, respectively)<br><br>Further independent risk factors increasing the odds of developing asthma were a parental history of allergic disease (aOR, 1.83; 1.29-2.60) and a period of breast feeding shorter than 6 months (aOR, 1.57; 1.03-2.39).<br><br>The incidence of rhinitis was increased for parental history of allergic disease (aOR, 2.00; 1.59-2.51) and polyvinylchloride flooring (aOR, 1.60; 1.02-2.51).	Eczema in infancy is associated with development of asthma and rhinitis during the following 5-year period, and eczema is one of the strongest risk factors.

Early eczema and the risk of childhood asthma: a prospective, population-based study.<br><br>Saunes M, 2012<br><br>[10]	Prospective, population-based study	The aim of the present study was to prospectively investigate the association between a history of eczema at 2 years of age and current asthma at 6 years of age in a general population.<br><br>We also aimed to determine the prevalence of allergy-related diseases at 6 years of age according to eczema status at 2 years of age.	The Prevention of Allergy among Children in Trondheim (PACT) study enrolled from Sept. 2000 to March 2005 the 2 y.o. and included the 6 y.o. from Sept. 2000 to Dec. 2008.	4780 children<br>- 2192 with follow-up data at 6 years<br><br>- 2588 with no follow-up data	Questionnaires assessing various environmental exposures and health variables were administered at 2 years of age.<br><br>An identical health questionnaire was completed at 6 years of age.	The estimate for the association between eczema at 2 years and current asthma at 6 years was OR=1.80 (95% CI 1.10-2.96).<br><br>Four of ten children with eczema at 6 years had the onset of eczema after the age of 2 years, but the co-existence of different allergy-related diseases at 6 years was higher among those with the onset of eczema before 2 years of age.	Early eczema was associated with an increased risk of developing childhood asthma.

Multifaceted allergen avoidance during infancy reduces asthma during childhood with the effect persisting until age 18 years.<br><br>Scott M, 2012<br><br>[11]	Single-blinded RCT	The aim of this study was to evaluate the efficacy of environmental modification in the first 12 months of life on the prevalence of asthma in high-risk individuals.	In 1990, 120 infants who were considered at high risk of developing allergic disease on the basis of dual heredity or single heredity were recruited at birth.	120 Children considered at high risk of allergic disorders, randomised into:<br>- prevention group (n=58)<br><br>- control group (n=62).	Infants in the intervention arm were breast fed with the mother on a low allergen diet or given an extensively hydrolysed formula. Exposure to house dust mite allergen was reduced.<br><br>The control group followed standard advice.<br><br>Children were assessed at ages 1, 2, 4, 8 and 18 years for the presence of asthma and atopy.	At 18 years of age, there was a significantly lower prevalence of asthma in the prevention group compared with the control group (OR: 0.23, 95% CI 0.08 to 0.70, p=0.01), primarily due to asthma that developed during childhood but persisted until age 18 years.<br><br>Repeated-measure analysis showed that there was an overall reduction in asthma prevalence from 1 to 18 years (OR: 0.51, CI 0.32 to 0.81, p=0.04).<br><br>Prevalence of atopy was not significantly different between the two groups at age 18.	Comprehensive allergen avoidance in the first year of life is effective in preventing asthma onset in individuals considered at high risk due to heredity. The effect occurs in the early years, but persists through to adulthood.

Severe eczema in infancy can predict asthma development. A prospective study to the age of 10 years.<br><br>Ekbäck M, 2014<br><br>[12]	Prospective multicenter study	To follow infants with eczema and suspected food allergy over time, focusing on sensitization to allergens, severity of eczema and the development of allergic airway symptoms at 4.5 and 10 years of age.<br><br>To identify any early signs that could be associated with a<br><br>higher risk of developing allergic airway symptoms.	The study population was 123 children (71 boys and 52 girls) with eczema and suspected food allergy recruited to the study from June 1999 to September 2001.	123 children (mean age 6 months, range 1–23).<br><br>115 children at the follow-up at 4.5 years of age.	Hanifin-Rajka criteria and SCORAD index were used to describe the eczema.<br><br>Episodes of wheezing were registered, skin prick tests and IgE tests were conducted and questionnaires were filled out.<br><br>At 4.5 years of age, exposure to tobacco smoke, having furred pets at home, sensitization to milk, egg and aeroallergens, SCORAD points, eczema fulfilling Hanifin- Rajka criteria, ARC and asthma were studied.	For children with remaining sensitization to egg at 4.5 years of age, the OR for ARC at 10 years of age was 10.30 (95%CI 3.28–32.38; P= 0.00).<br><br>Sensitization to aeroallergens at 4.5 years of age meant an OR of 4.70 (95%CI 1.50–14.75; P= 0.01) for ARC at 10 years of age and an OR of 6.21 (95%CI 1.83–21.06; P= 0.00) for asthma at 10 years of age.<br><br>ARC at 4.5 years of age gave an OR of 11.28 (95%CI 3.61–35.28; P= 0.00) for asthma development.<br><br>For children diagnosed with asthma at 4.5 years of age, the OR for asthma at 10 years of age was 14.44 (95%CI 4.77–43.72; P= 0.00), whereas the OR for only ARC at 10 years of age was 1.08 (95% CI 0.34–3.47; P =0.89).	Children with eczema and wheezing episodes during infancy are more likely to develop asthma than are infants with eczema alone. Eczema in infancy combined with early onset of ARC seems to indicate a more severe allergic<br><br>disease, which often leads to asthma development.

Effects of early-life exposure to allergens and bacteria on recurrent wheeze and atopy in urban children<br><br>Lynch SV, 2014<br><br>[13]	Prospective birth-cohort study with a nested case-control study	To examine environmental factors associated with recurrent wheezing in inner-city environments.	The Urban Environment and Childhood Asthma (URECA) study examined a birth cohort at high risk for asthma in Baltimore, Boston, New York, and St Louis.	N = 560<br><br>From 0 to 3 years of age	Environmental assessments included allergen exposure and, in a nested case-control study of 104 children, the bacterial content of house dust collected in the first year of life. Associations were determined among environmental factors, aeroallergen sensitization, and recurrent wheezing at age 3 years.	A Cumulative allergen exposure over the first 3 years was associated with allergic sensitization, and sensitization at age 3 years was related to recurrent wheeze:<br>- Any food: aOR 1.94 (CI 95% 1.25-3.03, p=0.003)<br>- Any aeroallergen: aOR 1.63 (CI 95% 1.03-2.56, p=0.04)<br>- Cat: aOR 1.93 (CI 95% 1.09-3.44, p=0.03)<br>- Dog: aOR 2.22 (CI 95% 1.14-4.34, p=0.02)<br>- Cockroach: aOR 1.74 (CI 95%0.94-3.21, p=0.08)<br>- Mouse: aOR 1.74 (CI 95% 1.01-2.97, p=0.04)<br>- Dust mite (D. farinae): aOR 2.40 (CI 95% 1.22-4.72, p=0.01)<br>- Dust mite (D. pteronyssinus): aOR 1.57 (CI 95% 0.82-3.03, p=0.17)<br><br>B. In contrast, first-year exposure to cockroach, mouse, and cat allergens was negatively associated with recurrent wheeze (OR 0.60, 0.65, and 0.75, respectively; P ≤ .01).<br><br>C. Differences in house dust bacterial content in the first year, especially reduced exposure to specific Firmicutes and Bacteriodetes, was associated with atopy and atopic wheeze.<br><br>D. Exposure to high levels of both allergens and this subset	In inner-city environments children with the highest exposure to specific allergens and bacteria during their first year were least likely to have recurrent wheeze and allergic sensitization. These findings suggest that concomitant exposure to high levels of certain allergens and bacteria in early life might be beneficial and suggest new preventive strategies for wheezing and allergic diseases

						of bacteria in the first year of life was most common among children without atopy or wheeze.	

Early-life risk factors for childhood wheeze phenotypes in a high-risk birth cohort.<br><br>Lodge CJ, 2014<br><br>[14]	Birth-cohort study	Definition of a longitudinal childhood wheeze phenotypes in children up 7 years of age.<br><br>Investigation over the relationships of these phenotypes with earlylife exposures and wheeze at age 12 years (not consistent with pico question).	The Melbourne Atopy Cohort Study (MACS), a high-allergy-risk birth cohort that enrolled pregnant women from Melbourne, Australia between 1990 and 1994.	620 infants	Telephone surveys conducted with the infants' mothers every 4 weeks from birth to age 15 months, once at age 18 months, yearly at age 2-7 years, and once at 12 years.<br>Mothers reported each time the days of cough, rattle, and wheeze experienced during the previous 4 weeks.<br><br>Potential early-life risk factors for wheeze reported:<br>- parental asthma, education, and smoking;<br>- infant sex and 4-week weight;<br>- breastfeeding;<br>- child atopy and early eczema;<br>- exposure to cats and/or dogs;<br>- lower respiratory tract infection (LRTI) in the first year;<br>- first-born status;<br>- childcare attendance by age 12 months<br><br>SPT used to test for cow's milk, egg white, peanut, house dust mite, rye grass, and cat dander allergies at age 1 and 2 years.	- Lower respiratory tract infection (LRTI) by 1 year (relative risk [RR], 3.00; 95% CI, 1.58-5.70), childcare by 1 year (RR, 1.51; 95% CI, 1.02-2.22), and higher body mass index (RR, 2.51; 95% CI, 1.09-5.81) were associated with increased risk of early transient wheeze.<br><br>- Breastfeeding was protective (RR, 0.54; 95% CI, 0.32-0.90).<br><br>- LRTI (RR, 6.54; 95% CI, 2.55-16.76) and aeroallergen sensitization (RR, 4.95; 95% CI, 1.74-14.02) increased the risk of early persistent wheeze.<br><br>- LRTI (RR, 5.31; 95% CI, 2.71-10.41), eczema (RR, 2.77; 95% CI, 1.78-4.31), aeroallergen sensitization (RR, 5.60; 95% CI, 2.86-10.9), and food sensitization (RR, 2.77; 95% CI, 1.56-4.94) increased the risk of intermediate-onset wheeze.<br><br>- Dog exposure at baseline (RR, 0.52; 95% CI, 0.32-0.84) and first-born status (RR, 0.49; 95% CI, 0.32-0.76) were protective.<br><br>- Heavy parental smoking at birth (RR, 3.18; 95% CI, 1.02-9.88) increased the risk of late-onset wheeze, whereas breastfeeding reduced it (RR, 0.34; 95%	Evidence of novel insights into potential causative or aggravating environmental exposures for different childhood wheeze phenotypes.

						CI, 0.12-0.96).	

Associations of Pet Ownership with Wheezing and Lung Function in Childhood: Findings from a UK Birth Cohort.<br><br>Collin SM, 2015<br><br>[15]	Birth-cohort study	Investigation of whether pet ownership during pregnancy and early childhood was associated with wheezing from birth to age 7 years and with lung function at age 8 years in a UK population-based birth cohort.	Pregnant women residing in the former Avon Health Authority in south-west England who had an estimated date of delivery between 1 April 1991 and 31 December 1992 were enrolled in the Avon Longitudinal Study of Parents and Children (ALSPAC)	4,706 children for primary outcome<br><br>4,177 children for secondary outcome	The primary source of data collection was via self-completion questionnaires sent to mothers at four time-points during pregnancy then at approximately annual intervals following birth.<br><br>In addition to the exposure to pets, potential confounder factors were also evaluated:<br>- sex of the child<br>- Maternal history of asthma or allergy,<br>- Maternal smoking during pregnancy,<br>- Family Adversity Index (a composite index of 18 elements evaluating the familial environment)<br><br>**Primary outcome — Episodes of wheeze and wheezing phenotypes.**<br>Parental reports of child wheezing were obtained from questionnaires sent to the mothers at annual intervals from 6 months to 7 years of age (at approximate ages 6, 18, 32, 42, 54, 69, and 81 months)<br><br>**Secondary outcome — Lung function.**	A. Ownership of any pet was associated with 13% higher odds of wheezing at age 6–8 months (odds ratio (OR) = 1.13; 95% CI 1.02–1.25) but there was no association at any of the later time-points and no overall association.<br>- Cat ownership was associated with lower odds of wheezing at ages 18 and 42 months with an overall 6% lower odds of wheezing (OR = 0.94; 95% CI 0.89–0.99) (Table 2, S2 Table).<br>- Rabbit or rodent increased the overall odds of wheezing by 21% (OR = 1.21; 95% CI 1.12–1.31) and 11% (OR = 1.11; 95% CI 1.02–1.21), respectively, with the strongest associations evident during infancy.<br>- Ownership of fish, turtles and tortoises was not associated with occurrence of wheezing.<br><br>B. Pet ownership was not associated with lung function at age 8 years, with the exception of positive associations of rodent and bird ownership with better lung function.<br>- For rodent ownership (comparing ever owned versus never owned), the mean	Cat ownership was associated with reduced risk, and rabbit and rodent ownership with increased risk, of wheezing during childhood.

					Lung function was measured by spirometry according to American Thoracic Society criteria.	difference in FEV1 z-score was 0.072 (95% CI 0.008 to 0.136, P = 0.03), and the mean difference in FVC z-score was 0.065 (95% CI 0.003 to 0.127, P = 0.04);<br>- for bird ownership, the mean difference in FEV1 z-score was 0.105 (95% CI 0.014 to 0.195, P = 0.02).	

Risk factors for recurrent wheezing in infants: a case-control study.<br><br>De Sousa RB, 2016<br><br>[16]	Case-Control study	To evaluate the association between recurrent wheezing and atopy, the Asthma Predictive Index, exposure to risk factors, and total serum IgE levels as potential factors to predict recurrent wheezing.	Infants treated at a specialized outpatient clinic from November 2011 to March 2013.	N = 113<br><br>From 6 to 24 months of age.<br><br>- 65 infants with recurrent wheezing (63.0% male) with a mean age of 14.8 (SD = 5.2) months;<br><br>- 48 healthy infants (44.0% male) with a mean age of 15.2 (SD = 5.1) months	Multiple analysis model included sensitivity to inhalant and food antigens, positive Asthma Predictive Index (API), and other risk factors for recurrent wheezing (smoking during pregnancy, presence of indoor smoke, viral infections, and total serum IgE levels).<br>A logistic regression model was applied to the independent variables presenting p < 0.10 in the univariate analysis. The strength of the association between wheezing condition and the various outcomes was evaluated by odds ratio. Pearson's Chi-square test was used for the categorical variables. Fisher's exact test was used when the expected values were lower than five. Student's t-test was used to compare the mean age between both groups. A significance level of 5% was considered.	A. Risk factors for recurrent wheezing:<br>- antigen sensitivity (OR = 12.45; 95%CI 1.28–19.11);<br>- positive Asthma Predictive Index (OR = 5.57; 95%CI 2.23–7.96);<br>- exposure to environmental smoke (OR = 2.63; 95%CI 1.09–6.30).<br><br>1533. Eosinophilia ≥ 4.0% e total IgE ≥ 100 UI/mL were more prevalent in the wheezing group but failed to remain in the model.<br><br>C. Smoking during pregnancy was identified in a small number of mothers, and secondhand smoke at home was higher in the control group.	Presence of atopy, positive Asthma Predictive Index and exposure to environmental smoke are associated to recurrent wheezing.<br>Identifying these factors enables the adoption of preventive measures, especially for children susceptible to persistent wheezing and future asthma onset

Prevalence and risk factors for atopic disease in a population of preschool children in Rome: Challenges to early intervention.<br><br>Indinnimeo L, 2016<br><br>[17]	Observational study	Evaluate the prevalence of the major allergic and respiratory diseases in a group of preschool children attending nurseries in Rome and assessing the related factors, especially in relation to environment	Children attending nursery schools	494 children<br><br>From 3 to 6 years of age	Standardized questionnaire (SIDRIA-2 protocol) for the assessment of potential risk factors and outcomes. Questionnaires were distributed at school, completed at home by parents and finally delivered in anonymous form to the teachers.<br><br>The questionnaire investigated: individual characteristics such as age, gender, medical history, family history, allergies, eating habits; environmental factors such as traffic level (defined by the parents), house crowding, presence of pets at home, nursery and daycare attendance; allergic and respiratory diseases such as rhinitis, wheezing in the past 12 months, asthma diagnosed by physician,	A1. UNIVARIATE ANALYSIS: Wheezing during the last 12 months positively associated with:<br>- siblings' history of atopy □ (odds ratio [OR], 2.42; 95% confidence interval [CI], 1.39–4.22);<br>- recurrent siblings' bronchitis □ (OR, 2.32; 95% CI, 1.26–4.27);<br>- dermatitis □ (OR, 1.69; 95% CI, 0.99–2.89).<br><br>A2. UNIVARIARE ANALYSIS: diagnosis of asthma positively associated with:<br>- nationality not being Italian □ (OR, 2.54; 95% CI, 1.09–5.92);<br>- duration of breastfeeding longer than 1 month □ (OR, 2.78; 95% CI, 0.97–7.97):<br>- daycare attendance □ (OR, 2.26; 95% CI, 1.14–4.52);<br>- mother's history of atopy □ (OR, 2.04; 95% CI, 1.13–3.66);<br>-siblings' history of atopy □(OR, 2.09; 95% CI, 1.12–3.89];<br>- recurrent siblings' bronchitis □ (OR, 1.99; 95% CI, 0.99–4.00);<br>- dermatitis □ (OR, 2.02; 95% CI, 1.13–3.61);<br>- Girls had a lower risk of	Wheezing prevalence (15.0%) was higher compared to the SIDRIA-2 study in Rome (9%) in children aged 6–7 years.<br><br>Allergic rhinitis prevalence was lower (5.5%) compared to previous Italian studies. The increase of allergic rhinitis and decrease of atopic dermatitis with age is observed.<br><br>The diagnosis of asthma was positively associated with the breastfeeding longer than 1 month.<br><br>Family size and siblings' recurrent bronchitis could be associated with a reduced risk of wheezing and allergic sensitization, respectively. The siblings' recurrent bronchitis is a

					respiratory symptoms, mouth breathing and snoring during sleep, food allergy or food anaphylaxis, eczema, urticaria, otitis, diarrhea, infectious diseases; medical history including therapies (e.g. bronchodilators, antihistamines, oral corticosteroids, nasal steroids, herbal medicine) received during the past year.	developing asthma with respect to boys □ (OR, 0.50; 95% CI, 0.27–0.90).<br><br>B1. MULTIVARIATE ANALYSIS: association of wheezing in the last 12 months with<br>- siblings' history of atopy and correlation is even stronger □ (ORadj, 4.43; 95% CI, 1.95–10.0);<br>□ protective effect of having more than one sibling becomes statistically significant □ (ORadj, 0.17; 95% CI, 0.04–0.76).<br><br>B2. MULTIVARIATE ANALYSIS: The analysis of asthma onset confirms an effect of gender (girls were protected compared to boys), daycare attendance, and maternal history of atopy. No statistically significance for breastfeeding longer than 1 month.<br><br>C. Urban traffic, pets, passive smoking, and house crowding do not represent significant risk factors for respiratory symptoms (small size sample?)	protective factor for atopy.<br><br>Repeated viral infections reduce the risk for the development of asthma up to school age, whereas no effects were observed for other types of infection<br><br>Urban traffic, pets, passiv e smoking, and house crowding do not represent significant risk factors for atopy and respiratory symptoms.

Sensitization predicts asthma development among wheezing toddlers in secondary healthcare.<br><br>Boersma NA, 2017<br><br>[18]	Longitudinal study	To examine the predictive value of sensitization to inhalant allergens in wheezing toddlers in a secondary care setting for the<br><br>development of asthma at school age.	All children who visited the St. Antonius Hospital between January 1, 2007 and December 31, 2011 with<br><br>wheezing at the age of 1, 2, or 3 years.	166 children	The Dutch version of the "International Study of Asthma and Allergies in Childhood" (ISAAC) core questionnaire was used to collect data about wheezing symptoms at school age.<br><br>Information about specific IgE measurement at the age of 1–3 years and<br><br>hospitalization for respiratory problems was obtained.	Eczema in the first year of life combined with hospital admission in the first 3 years of life resulted in the best prediction of asthma at school age based on non-invasive determinants. The odds ratios of these determinants in a combined model were 4.6 (CI 1.7–12.3) and 3.7 (1.5–9.0), respectively.<br><br>Combined with eczema and hospital admission, sensitization remained a strong predictor of asthma, with respective odds ratios of 2.7 (CI 0.9–7.8), 4.2 (CI 1.5–11.4), and 6.8 (2.4–18.9) in multivariate analysis.	Sensitization to inhalant allergens at the age of 1–3 years is probably a strong predictor of asthma.

Prevalence and risk factors for wheezing and allergic diseases in preschool children: A perspective from the Mediterranean coast of Turkey<br><br>Bolat E, 2017<br><br>[19]	Phase 1: prevalence study<br><br>Phase 2: case-control study	Determine the prevalence and risk Factors of respiratory And allergic diseases Using a modified ISAAC questionnaire In preschool children Attending daycare Centres in the city<br><br>Of Mersin.	All children Attending day-care Centres in the City centre Who were Randomly selected from The list of all Daycare centres in Mersin from January to December<br><br>2011.	396 preschool children<br>- 206 males<br>- 190 females<br><br>Mean age: 4.4± 0.9years.	**Phase 1.**<br>A modified ISAAC Questionnaire was used to assess the Symptoms of allergic And respiratory diseases, and the potential risk factors For the outcomes. The questionnaire Included questions About the symptoms And diagnosis of Respiratory diseases, eczema, food allergy And risk factors such As demographic characteristics, gestational factors, Family history, feeding practices, household Characteristics such As house crowding, Presence of pets, dampness, and tobacco Smoke exposure.<br><br>**Phase 2.**<br>Serum food and inhalant specific IgE, And skin tests were Performed in 45	Significant risk factors for physician-diagnosed asthma:<br>- family history of atopy (OR=2.5, 95% CI: 1.3-4.7, p=0.004),<br>- dampness at home (OR=2.4, 95% CI: 1.2-4.8, p=0.008),<br>- a history of intestinal parasites (OR=4.3, 95% CI: 1.7-10.9, p=0.002),<br>- previous history of pneumonia (OR=6.9, 95% CI: 1.9-25.9, p=0.004),<br>- initiation of complementary foods before the age of three months (OR=6.1, 95%CI: 1.4-26.9, p=0.02)<br>- presence of food allergy (OR=3.1, 95% CI: 1.1-9.2, p=0.03)<br><br>The risk factors for frequent wheezing were:<br>- maternal smoking during pregnancy (OR=5.2, 95% CI: 0.9-28.7, p=0.05)<br><br>- high serum IgE levels (OR=2.9, 95% CI: 0.9-9.0, p=0.05) at borderline significance.	A high prevalence of asthma and allergic diseases, probably related to humid climatic properties in addition to other environmental and genetic factors was demonstrated.

					children with frequent Wheezing and 28 Children with no wheezing.		

Food Allergy Is an Important Risk Factor for Childhood Asthma, Irrespective of Whether It Resolves.<br><br>Vermeulen EM, 2018<br><br>[20]	Longitudinal population based cohort study	To understand whether challenge-proven food<br><br>allergy in infancy increases the risk of asthma at age 4 years.	The HealthNuts study, a longitudinal population-based cohort<br><br>study of allergic disease in Melbourne, Australia.	2789 12-month-old infants	Infants underwent skin prick test to egg, peanut, and sesame and those with a detectable skin prick test result had oral food challenges.<br>At age 4 years, food challenges were repeated to determine persistence or resolution of food allergy.<br><br>The association between food allergy and doctor-diagnosed asthma was examined using binomial regression.	Children with food allergy at age 1 year had an increased risk of asthma (1 food allergy: relative risk [RR], 1.69; 95% CI, 1.29-2.21; 2 or more food allergies: RR, 2.76; 95% CI, 1.94-3.92).<br><br>The risk of asthma was highest in children with food allergy and coexistent eczema in infancy (RR, 2.87; 95% CI, 2.22-3.70).<br><br>Transient food allergy and persistent food allergy<br><br>were both associated with an increased risk of asthma (transient egg allergy: RR, 1.92; 95% CI, 1.46-2.51; persistent egg allergy: RR, 2.60; 95% CI, 1.76-3.85).	Asthma at age 4 years is twice as common in those with challenge-proven food allergy at age 1 year, irrespective of whether the food allergy subsequently resolves.<br><br>Children with 2 or more food allergies and those with coexistent eczema were almost 3 times as likely to develop asthma compared with those with no food allergies.

Early-onset eczema is associated with increased milk sensitization and risk of rhinitis and asthma in early childhood.<br><br>Chiu CY, 2019<br><br>[21]	Birth cohort study	To investigate the differences in early onset<br>(<2 years old) and late-onset (>/=2 years old) eczema<br>in children.	Birth cohort in the Prediction of Allergies in Taiwanese Children (PATCH) study for a 4-year follow-up period.	186 children, classified into three groups: - early-onset eczema(< 2 years old, n=55),<br>- late-onset eczema (>/= 2 years old, n=40),<br>- never eczema groups (n=91).	The parents or legal guardians of the subjects answered a questionnaire derived from well-validated International Study of Asthma and Allergies in Childhood (ISAAC) at birth, 6 months, and 1, 2, 3, and 4 years of age.<br><br>A complete peripheral blood cell count was obtained usingautomated analysis, and the absolute eosinophil count (AEC) and levels of total serum IgE was calculated.	A significantly higher prevalence of sensitization to food, especially milk,<br>was found in children with early-onset eczema compared with children without eczema at ages 1, 1.5, 2, 3, and 4 years old.<br><br>A significantly higher number of eosinophils was detected in children with early or late-onset eczema compared with children without eczema at the age of 1.5 years.<br><br>The early-onset eczema showed a significantly increased risk of<br>allergic rhinitis [odds ratio (OR), 3.71; 95% confidence interval<br><br>(CI), 1.37e10.02; P Z 0.010] and asthma (OR, 3.80; 95% CI, 1.12e12.85; P Z 0.032) at 4 years old.	In the early childhood, eczema appears to<br>have increased the AEC, with higher prevalence of sensitization<br>to milk, especially in children with early-onset<br>eczema. Although both the early- and late-onset eczema<br>are associated with higher prevalence of atopic diseases,<br>early-onset eczema is significantly related to the increased<br><br>risk of developing allergic airway diseases later in life.

Development of atopic sensitization in Finnish and Estonian children: A latent class analysis in a multicenter cohort.<br><br>Schmidt F, 2019<br><br>[22]	Birth cohort study	To define phenotypes of atopic sensitization in early childhood and examine their association with allergic<br><br>diseases and hereditary background in Finland and Estonia.	Finnish and Estonianchildren from the DIABIMMUNE multicenter young children<br><br>Cohort, born between January 2006 and July 2008.	1603 Finnish and 1657 Estonian Children.	Information about atopic eczema, allergic rhinitis, and wheezing was obtained by using validated questions from the International Study of Asthma and Allergies in Childhood.<br><br>Concentrations of sIgE antibodies to common food allergens (hen's egg, cow's milk, and peanut) and aeroallergens (cat, dog, house dust mite, Timothy<br><br>grass, and birch pollen) were measured.	The strongest associations of high total and specific IgE levels with disease were found for the severe atopy phenotype, which was strongly associated with<br>- wheeze (OR, 5.64 [95% CI, 3.07-10.52] and 4.56 [95% CI, 2.35-8.52]),<br>- allergic rhinitis (OR, 22.4 [95% CI, 11.67- 44.54] and 13.97 [95% CI, 7.33-26.4]), - atopic eczema (OR, 9.39 [95% CI, 4.9-19.3] and 9.5 [95% CI, 5.2-17.5] for Finland and Estonia, respectively).	Despite profound differences in environmental exposures, there might exist genuine patterns of atopic sensitization. The distribution of these patterns might determine<br><br>the contribution of atopic sensitization to disease onset.

Treatment of allergic rhinitis reduces acute asthma exacerbation risk among asthmatic children aged 2-18 years.<br><br>Yu CL, 2019<br><br>[23]	Population-based cohort study	To determine the relationship between Allergic rhinitis (AR) and asthma with Acute exacerbations (AE) among asthma patients.	A group of preschool children (2-6 y.o.)<br><br>A group of school-age children (7-18 y.o.)	6506 aged 2-6 y.o.<br><br>Preschool children (2-6 y.o.)<br><br>School-age children (7-18 y.o.)	To determine the prevalence rates of AR among asthmatic children and to assess whether different age groups of asthmatic children with or without AR have an increased incidence of acute exacerbation (AE) of asthma.<br><br>To determine whether treatment of AR with INCS (intra-nasal corticosteroids) reduce the risk of AE in asthmatic children of different ages.	1533. Prevalence of AE higher in the preschool (2–6 years old) group than the older (7–18 years old) group (adj. HR: 1.68, 95% CI: 1.44–1.95).<br><br>A1. The prevalence of AE did not differ significantly between male and female patients.<br><br>1533. The AR without treatment group did not have a significant difference with the non-AR group (HR: 0.78, 95% CI: 0.59–1.02), but the AR with INCS (intra-nasal corticosteroids) and/or SGH (second generation antihistamine) group was found to have a lower risk of AE within one year of follow-up than the non-AR group (HR: 033, 95% CI: 0.27–0.42; 0.39, 95% CI: 0.30–0.51; 0.28, 95% CI: 0.21–0.38). The risk of AE in the AR with treatment groups was also found to be lower	Primary outcome: occurrence of acute exacerbations of asthma lower risk of AE in the AR patients with INCS and/or SGH treatment, but no reduced risk in the AR patients without treatment compared to the non-AR patients.<br><br>Other outcomes: evaluation of the effects of drugs used for AR on AE (not consistent with the PICO question)

						than that in the non-AR group after adjustments were made for age, gender, asthma controller, acute sinusits, and GERD (adj. HR: 032, 95% CI: 0.26–0.41; 0.44, 95% CI: 0.34–0.58; 0.30, 95% CI: 0.22–0.40).<br><br>B1. Same results found in the different age groups.	

Rhinovirus bronchiolitis, maternal asthma, and the development of asthma and lung function impairments<br><br>Da Silva Sena CR, 2021<br><br>[24]	Prospective birth cohort study	To investigate the combined effects of hospitalization for RV positive bronchiolitis in infancy and a history of maternal asthma on the development of asthma at preschool age.	Preschool-aged children, with a history of hospital admission for bronchiolitis in infancy	N = 139<br><br>(64.8% males)<br><br>Age at admission in months: 7 (2-11)<br><br>Age at a visit in months: 47 (42-51)	A follow-up to ascertain asthma and asthma-like symptoms was conducted, along with skin prick allergy test positivity, and lung function measured pre- and post-bronchodilator using impulse oscillometry.	A. Children with a past hospitalization for RV positive bronchiolitis (42.4% of all) and a history of maternal asthma (36.7% of all) had the greatest prevalence and risk ratio (RR) for<br>- doctor-diagnosed asthma [prevalence 81.8%; RR 2.10 (95%CI 1.37–3.19, p = .001)];<br>- use of inhaled corticosteroids [68.2%; RR 2.17, 95% CI 1.19–3.99, p = .001)];<br>- short-acting β-agonists in the last 12 months [(95.2% and RR 1.49, 95% CI 1.17–1.89, p = .001)]<br>as compared to those with RV negative bronchiolitis and no maternal asthma history.<br><br>B. More children in this group had an abnormal airway resistance (33.3% and adjusted risk ratio [aRR] 3.11, 95% CI 1.03–9.47, p = .045) and reactance (27.8% and aRR 2.11, 95% CI 1.06–4.26, p = .035) at 5 Hz, measured with impuls oscillometry, as compared to those with RV negative bronchiolitis and no maternal asthma history.	Hospitalization for RV positive bronchiolitis in early life combined with a history of maternal asthma identifies a subgroup of children with a high asthma burden while participants with only one of the two risk factors had intermediate risk for asthma.

References

1. Henderson J, Granell R, Heron J, Sherriff A, Simpson A, Woodcock A, Strachan DP, Shaheen SO, Sterne JA. Associations of wheezing phenotypes in the first 6 years of life with atopy, lung function and airway responsiveness in mid-childhood. Thorax. 2008 Nov;63(11):974-80. doi: 10.1136/thx.2007.093187. Epub 2008 Aug 4. PMID: 18678704; PMCID: PMC2582336.
2. Bufford JD, Reardon CL, Li Z, Roberg KA, DaSilva D, Eggleston PA, Liu AH, Milton D, Alwis U, Gangnon R, Lemanske RF Jr, Gern JE. Effects of dog ownership in early childhood on immune development and atopic diseases. Clin Exp Allergy. 2008 Oct;38(10):1635-43. doi: 10.1111/j.1365-2222.2008.03018.x. Epub 2008 Aug 12. PMID: 18702654.
3. Donohue KM, Al-alem U, Perzanowski MS, Chew GL, Johnson A, Divjan A, Kelvin EA, Hoepner LA, Perera FP, Miller RL. Anti-cockroach and anti-mouse IgE are associated with early wheeze and atopy in an inner-city birth cohort. J Allergy Clin Immunol. 2008 Nov;122(5):914-20. doi: 10.1016/j.jaci.2008.08.034. PMID: 19000580; PMCID: PMC2590748.
4. Simon MR, Havstad SL, Wegienka GR, Ownby DR, Johnson CC. Risk factors associated with transient wheezing in young children. Allergy Asthma Proc. 2008 Mar-Apr;29(2):161-5. doi: 10.2500/aap.2008.29.3089. PMID: 18430313.
5. Simon MR, Havstad SL, Wegienka GR, Ownby DR, Johnson CC. Risk factors associated with transient wheezing in young children. Allergy Asthma Proc. 2008 Mar-Apr;29(2):161-5. doi: 10.2500/aap.2008.29.3089. PMID: 18430313.
6. Acevedo N, Sánchez J, Zakzuk J, Bornacelly A, Quiróz C, Alvarez Á, Puello M, Mendoza K, Martínez D, Mercado D, Jiménez S, Caraballo L. Particular characteristics of allergic symptoms in tropical environments: follow up to 24 months in the FRAAT birth cohort study. BMC Pulm Med. 2012 Mar 22;12:13. doi: 10.1186/1471-2466-12-13. PMID: 22439773; PMCID: PMC3331807.
7. Jackson DJ, Evans MD, Gangnon RE, Tisler CJ, Pappas TE, Lee WM, Gern JE, Lemanske RF Jr. Evidence for a causal relationship between allergic sensitization and rhinovirus wheezing in early life. Am J Respir Crit Care Med. 2012 Feb 1;185(3):281-5. doi: 10.1164/rccm.201104-0660OC. Epub 2011 Sep 29. PMID: 21960534; PMCID: PMC3297109.
8. Arshad SH, Karmaus W, Raza A, Kurukulaaratchy RJ, Matthews SM, Holloway JW, Sadeghnejad A, Zhang H, Roberts G, Ewart SL. The effect of parental allergy on childhood allergic diseases depends on the sex of the child. J Allergy Clin Immunol. 2012 Aug;130(2):427-34.e6. doi: 10.1016/j.jaci.2012.03.042. Epub 2012 May 18. PMID: 22607991; PMCID: PMC3409323.
9. von Kobyletzki LB, Bornehag CG, Hasselgren M, Larsson M, Lindström CB, Svensson Å. Eczema in early childhood is strongly associated with the development of asthma and rhinitis in a prospective cohort. BMC Dermatol. 2012 Jul 27;12:11. doi: 10.1186/1471-5945-12-11. PMID: 22839963; PMCID: PMC3469362.
10. Saunes M, Øien T, Dotterud CK, Romundstad PR, Storrø O, Holmen TL, Johnsen R. Early eczema and the risk of childhood asthma: a prospective, population-based study. BMC Pediatr. 2012 Oct 24;12:168. doi: 10.1186/1471-2431-12-168. PMID: 23095804; PMCID: PMC3532218.
11. Scott M, Roberts G, Kurukulaaratchy RJ, Matthews S, Nove A, Arshad SH. Multifaceted allergen avoidance during infancy reduces asthma during childhood with the effect persisting until age 18 years. Thorax. 2012 Dec;67(12):1046-51. doi: 10.1136/thoraxjnl-2012-202150. Epub 2012 Aug 2. PMID: 22858926.
12. Ekbäck M, Tedner M, Devenney I, Oldaeus G, Norrman G, Strömberg L, Fälth-Magnusson K. Severe eczema in infancy can predict asthma development. A prospective study to the age of 10 years. PLoS One. 2014 Jun 10;9(6):e99609. doi: 10.1371/journal.pone.0099609. PMID: 24914552; PMCID: PMC4051764.
13. Lynch SV, Wood RA, Boushey H, Bacharier LB, Bloomberg GR, Kattan M, O'Connor GT, Sandel MT, Calatroni A, Matsui E, Johnson CC, Lynn H, Visness CM, Jaffee KF, Gergen PJ, Gold DR, Wright RJ, Fujimura K, Rauch M, Busse WW, Gern JE. Effects of early-life exposure to allergens and bacteria on recurrent wheeze and atopy in urban children. J Allergy Clin Immunol. 2014 Sep;134(3):593-601.e12. doi: 10.1016/j.jaci.2014.04.018. Epub 2014 Jun 4. PMID:

24908147; PMCID: PMC4151305.
14. Lodge CJ, Zaloumis S, Lowe AJ, Gurrin LC, Matheson MC, Axelrad C, Bennett CM, Hill DJ, Hosking CS, Svanes C, Abramson MJ, Allen KJ, Dharmage SC. Early-life risk factors for childhood wheeze phenotypes in a high-risk birth cohort. J Pediatr. 2014 Feb;164(2):289-94.e1-2. doi: 10.1016/j.jpeds.2013.09.056. Epub 2013 Nov 14. PMID: 24238860.
15. Collin SM, Granell R, Westgarth C, Murray J, Paul ES, Sterne JA, Henderson AJ. Associations of Pet Ownership with Wheezing and Lung Function in Childhood: Findings from a UK Birth Cohort. PLoS One. 2015 Jun 10;10(6):e0127756. doi: 10.1371/journal.pone.0127756. PMID: 26061067; PMCID: PMC4465326.
16. de Sousa RB, Medeiros D, Sarinho E, Rizzo JÂ, Silva AR, Bianca AC. Risk factors for recurrent wheezing in infants: a case-control study. Rev Saude Publica. 2016;50:15. doi: 10.1590/S1518-8787.2016050005100. Epub 2016 May 3. PMID: 27143615; PMCID: PMC4904490.
17. Indinnimeo L, Porta D, Forastiere F, De Vittori V, De Castro G, Zicari AM, Tancredi G, Melengu T, Duse M. Prevalence and risk factors for atopic disease in a population of preschool children in Rome: Challenges to early intervention. Int J Immunopathol Pharmacol. 2016 Jun;29(2):308-19. doi: 10.1177/0394632016635656. Epub 2016 Mar 2. PMID: 26936651; PMCID: PMC5806707.
18. Boersma NA, Meijneke RWH, Kelder JC, van der Ent CK, Balemans WAF. Sensitization predicts asthma development among wheezing toddlers in secondary healthcare. Pediatr Pulmonol. 2017 Jun;52(6):729-736. doi: 10.1002/ppul.23668. Epub 2017 Jan 11. PMID: 28076664.
19. Bolat E, Arikoglu T, Sungur MA, Batmaz SB, Kuyucu S. Prevalence and risk factors for wheezing and allergic diseases in preschool children: A perspective from the Mediterranean coast of Turkey. Allergol Immunopathol (Madr). 2017 Jul-Aug;45(4):362-368. doi: 10.1016/j.aller.2017.01.002. Epub 2017 Apr 11. PMID: 28410872.
20. Vermeulen EM, Koplin JJ, Dharmage SC, Gurrin LC, Peters RL, McWilliam V, Ponsonby AL, Dwyer T, Lowe AJ, Tang MLK, Allen KJ; HealthNuts investigators. Food Allergy Is an Important Risk Factor for Childhood Asthma, Irrespective of Whether It Resolves. J Allergy Clin Immunol Pract. 2018 Jul-Aug;6(4):1336-1341.e3. doi: 10.1016/j.jaip.2017.10.019. Epub 2017 Nov 15. PMID: 29153880.
21. Chiu CY, Yang CH, Su KW, Tsai MH, Hua MC, Liao SL, Lai SH, Chen LC, Yeh KW, Huang JL. Early-onset eczema is associated with increased milk sensitization and risk of rhinitis and asthma in early childhood. J Microbiol Immunol Infect. 2020 Dec;53(6):1008-1013. doi: 10.1016/j.jmii.2019.04.007. Epub 2019 May 8. PMID: 31129013.
22. Schmidt F, Hose AJ, Mueller-Rompa S, Brick T, Hämäläinen AM, Peet A, Tillmann V, Niemelä O, Siljander H, Knip M, Weber J, von Mutius E, Ege MJ; DIABIMMUNE Study Group. Development of atopic sensitization in Finnish and Estonian children: A latent class analysis in a multicenter cohort. J Allergy Clin Immunol. 2019 May;143(5):1904-1913.e9. doi: 10.1016/j.jaci.2018.12.1014. Epub 2019 Jan 23. PMID: 30682459.
23. Yu CL, Huang WT, Wang CM. Treatment of allergic rhinitis reduces acute asthma exacerbation risk among asthmatic children aged 2-18 years. J Microbiol Immunol Infect. 2019 Dec;52(6):991-999. doi: 10.1016/j.jmii.2018.10.003. Epub 2018 Oct 25. PMID: 30409760.
24. Da Silva Sena CR, Morten M, Meredith J, Kepreotes E, E Murphy V, G Gibson P, D Robinson P, D Sly P, Whitehead B, Karmaus W, Collison A, Mattes J. Rhinovirus bronchiolitis, maternal asthma, and the development of asthma and lung function impairments. Pediatr Pulmonol. 2021 Feb;56(2):362-370. doi: 10.1002/ppul.25165. Epub 2020 Nov 24. PMID: 33179407.

Question 2. Does the presence of risk factors such as respiratory tract infection/bronchiolitis influence the onset and the evolution of preschool wheezing?

Search strategies:
(“child, preschool”[MeSH Terms] OR “toddler*”[All Fields]) AND (“respiratory sounds”[MeSH Terms] OR “wheez*”[All Fields] OR “Asthma”[MeSH Terms:noexp]) AND (“bronchiolitis”[MeSH Terms] OR “previous bronchiolitis”[All Fields] OR “respiratory syncytial virus”[All Fields] OR “RSV infection”[All Fields] OR “previous RSV infection”[All Fields]) AND “english”[Language] AND 2008/01/01:2021/12/31[Date — Publication].

("child, preschool"[MeSH Terms] OR "toddler*"[All Fields]) AND ("respiratory sounds"[MeSH Terms] OR "wheez*"[All Fields] OR "Asthma"[MeSH Terms:noexp]) AND "Risk Factors"[MeSH Terms] AND ("Age of Onset"[MeSH Terms] OR "Onset"[All Fields]) AND "english"[Language] AND 2008/01/01:2021/12/31[Date - Publication]

("child, preschool"[MeSH Terms] OR "toddler*"[All Fields]) AND ("respiratory sounds"[MeSH Terms] OR "wheez*"[All Fields] OR "Asthma"[MeSH Terms:noexp]) AND "Risk Factors"[MeSH Terms] AND ("disease progression"[MeSH Terms] OR "disease evolution"[All Fields] OR "evolution"[All Fields]) AND "english"[Language] AND 2008/01/01:2021/12/31[Date - Publication]

Title of the study, first author, year	Type of study	Objective	Population	N of patients, Age	Experiments/mechanisms assessed	Relative risk (RR) or odds ratio (OR) + confidence interval (CI 95%)	Results
Allergic sensitization is associated with rhinovirus-, but not other virus-, induced wheezing in children.<br><br>Jartti T, 2010<br><br>[1]	Observational study	To evaluate the association between allergic sensitization and each virus causing wheezing in hospitalized children	Hospitalized wheezing children	N = 247; (median age 1.6; interquartile range 1.1, 2.9)	Allergic sensitization indexes (such as specific IgE, total IgE, blood and nasal 81osinophil count, exhaled nitric oxide, eczema, family history positive for allergy and asthma, number of wheezing episodes and use of inhaled corticosteroid) have been correlated with each specific viral etiology found in children hospitalized for wheezing	Atopy was associated with rhinovirus (n = 58). The number of sensitizations was associated with rhinovirus (OR 4.59; 95% confidence interval 1.78, 11.8), aeroallergen sensitization (respectively; 4.18; 2.00, 8.72), total IgE level (2.06; 1.32, 3.21), food allergen sensitization (2.02; 1.08, 3.78), and nasal eosinophil count (1.52; 1.08, 2.13).<br><br>No correlation was found between atopy and respiratory syncytial virus, enterovirus, bocavirus, other virus, mixed viral infection, or virus negative etiology.	Allergic<br><br>sensitization is associated with rhinovirus induced wheezing, but not with other viral etiologies.

Serious early childhood wheezing after respiratory syncytial virus lower respiratory tract illness (RSV-LRI) in preterm infants.<br><br>Romero JR, 2010<br><br>[2]	Retrospective cohort study	To determine whether RSV-LRI during early infancy of preterm infants was associated with an increased risk for serious early childhood wheezing (SECW) by age 3 years.	The study population included infants ≤6 months of age born at ≤36 weeks' gestational age or weighing <2500 g, or both. Preterm infants with any medically attended RSV-LRI from May 2001 through April 2004 with 3 years of continuous eligibility were selected and propensity matched with ≤3 control infants.	Infants with RSV infection: 378<br><br>Controls: 606<br><br>Up to 6 years of age	The presence of SECW between ages 2 and 3 years was compared between infants with and without RSV-LRI using univariate and multivariate methods. Health care costs for patients with SECW were explored.	Preterm infants with RSV in early life were 2.52-fold (95% CI,1.65–3.85) more likely to present with SECW between ages 2 and 3 years ($P < 0.001$).	The development of RSV-LRI in infancy in preterm infants was associated with an increased prevalence of SECW between ages 2 and 3 years.<br><br>(Patients with SECW had higher total health care costs than those who did not have SECW.)

Severe early lower respiratory tract infection is associated with subsequent respiratory morbidity in preschool Inuit children in Nunavut, Canada.<br><br>Kovesi TA, 2011<br><br>[3]	Cross-sectional survey	To compare respiratory symptoms in preschool children with lower respiratory tract infection (LRTI) in the first two years of life and controls	Inuit preschool children	N = 388<br><br>3- to 5-years-old Inuit children	Evaluation of respiratory symptoms in Inuit preschool children through a questionnaire, comparing those with LRTI infection in the first two years of life with controls.	Prevalence of LRTI in the first 2 years of life: N 112 (32.5%)<br><br>Wheezing: LRTI group: 36.9% (41/111) vs controls: 17.3%(40/231)<br>RR: 2.1 (1.5–3.1)<br><br>Hospitalized in the past 12 months: LRTI group11.6% (13/112) vs controls: 1.3%(3/230)<br>RR: 8.9 (2.6–30.6)<br><br>Bronchitis diagnosis in past 12 months: LRTI group 9.9% (11/111) vs controls 1.7%(4/231)<br>RR: 5.7 (1.9–17.6)<br><br>Pneumonia diagnosis in past 12 months: LRTI group: 16.1% (18/112) vs controls 1.3%(3/231)<br>RR: 12.4 (3.7–41.1)	Severe LRTI in the first 2 years of life is associated with ongoing respiratory morbidity in preschool Inuit children

Determinants of asthma after severe respiratory syncytial virus bronchiolitis.<br><br>Bacharier LB, 2012<br><br>[4]	Prospective cohort study	Follow up until 6 years of age of children with severe RSV bronchiolitis at ≤ 12 months<br>Analysis of analyzed CCL5 (RANTES) mRNA expression in upper airway epithelial cells in a subgroup (81 children)	Children with initial severe RSV bronchiolitis	N = 206<br><br>Age ≤ 12 months until 6 years of age	To evaluate the determinants of physician-diagnosed asthma after severe RSV bronchiolitis	48% of children had physician-diagnosed asthma<br>Increased risk for asthma is found in:<br>- maternal asthma (OR, 5.2; 95% CI, 1.7-15.9; P = .004)<br>- exposure to high levels of dog allergen (OR, 3.2; 95% CI, 1.3-7.7; P = .012)<br>- aeroallergen sensitivity at age 3 years (OR, 10.7; 95% CI, 2.1-55.0; P = .005)<br>- recurrent wheezing during the first 3 years of life (OR, 7.3; 95% CI, 1.2-43.3; P = .028<br>- CCL5 expression in nasal epithelia during acute RSV infection (OR, 3.8; 95% CI, 1.2-2.4; P < .001).<br>Reduced risk for asthma is found in:<br><br>- white children (OR, 0.19; 95% CI, 0.04-0.93; P = .041) — children attending day care (OR, 0.18; 95% CI, 0.04-0.84; P = .029)	48% of children have an asthma diagnosis after severe RSV bronchiolitis.<br><br>There is an increased risk of asthma in those with increased CCL5 in nasal epithelia at the time of bronchiolitis or in those with the development of allergic sensitization by age 3 years.

Environmental factors association between asthma and acute bronchiolitis in young children—a perspective cohort study.<br><br>Lin HV, 2012<br><br>[5]	Prospective cohort study	Evaluation of the association between asthma or acute bronchiolitis and various risk factors in young children	Children with diagnosis of acute bronchiolitis vs control group, from Taiwan Longitudinal Health Insurance Database	4586 children younger than 2 years with a recorded diagnosis of acute bronchiolitis vs control group (N: 4,263).	Statistical analysis concerning a 3-years follow-up data of children with acute bronchiolitis compared to controls	In total 355 experienced asthma: children with acute bronchiolitis were more likely to have asthma than control population (HR: 13.55, 95% CI 8.87-20.71).	Young children with acute bronchiolitis should be monitored for 2 years to prevent them from developing asthma.

Human rhinovirus species C (HRV-C) infection in young children with acute wheeze is associated with increased acute respiratory hospital admissions.<br><br>Cox DW, 2013<br><br>[6]	Prospective study	Prospective collection of clinical information and nasal samples in children presenting to hospital with wheezing episode	Children with acute wheezing episodes	197 children younger than 5 years of age<br><br>Presenting to hospital with acute wheezing	To determine whether acute wheezing exacerbations due to HRV-C are associated with increased hospital attendances due to acute respiratory illnesses (ARIs).	HRV-C was identified in 81 (67.5%) samples.<br><br>ARI was increased in:<br><br>-children with an HRV-related wheezing illness (RR 3.44; 95% CI, 1.17-10.17; P = 0.03) compared with any other virus.<br><br>- in atopic subjects (RR 6.82; 95% CI, 2.16-21.55; P = 0.001).<br><br>Hospital admission was increased:<br><br>-in HRV-C, compared with any other virus, both before (49.4% vs. 27.3%, respectively; P = 0.004) and within 12 months (34.6% vs. 17.0%; P = 0.01) of recruitment.	HRV-C-related wheezing illnesses is associated with an increased risk of prior and subsequent hospital respiratory admissions.

Persistent recurring wheezing in the fifth year of life after laboratory-confirmed, medically attended respiratory syncytial virus infection in infancy.<br><br>Escobar GJ, 2013<br><br>[7]	Retrospective cohort study	Study the outcomes of a cohort of children born at ≥32 weeks gestational age (GA) who were followed through the fifth year of life, in an expanded cohort with two additional years of follow-up.<br>The goal was to:<br>1533) uantify the relationship between laboratory-confirmed, medically attended RSV infection in the first year of life with the presence of recurrent wheezing (RW) during a time when the	Infants > 32 weeks GA, from the Kaiser Permanente Northern California Database.<br><br>The study population is ethnically diverse, and the majority of mothers (73%) were 18–34 years of age. Using maternal records, we determined that 3.6% of infants had a maternal history of asthma.	72.602 children<br><br>From birth to 5 years of age	The occurrence of wheezing at ages 3 and 5 years was analyzed using logistic regression.	1533. RSV infection that only involved an outpatient encounter had an adjusted odds ratio (aOR) of 1.38 (95% CI, 1.03–1.85)<br><br>A1. RSV infection that involved prolonged hospitalization aOR 2.59 (95% CI, 1.49–4.50).<br><br>B. Exposure to oxygen in the neonatal period showed a similar gradient:<br>- <200 hours oxygen, no BPD aOR 1.32 (95% CI, 1.12 -1.57; p = 0.0012)<br>- >200 hours oxygen, no BPD aOR 2.00 (95% CI, 1.37 -2.90; p = 0.0003)<br>- oxygen exposure in BPD aOR 2.80 (95% CI, 1.31 - 5.98; p = 0.0078)<br><br>1533. Infections with other pathogens than RSV were not associated with increased rates of RW at age 5 years.<br><br>C1. Prolonged hospitalizations with unspecified organisms did show a strong association (AOR, 3.46, 95% CI, 1.94–6.16).<br><br>D. Gestational age <37	Primary outocome: occurrence of RW in the fifth year of life<br><br>Laboratory-confirmed, medically attended RSV infection, prematurity, and neonatal exposure to supplemental oxygen have independent associations with development of recurrent wheezing in the fifth year of life.

		prevalence of these conditions is expected to decrease.<br><br>2) explore the degree to which the relationships were dependent on the RW definition				weeks was associated with increased risk of RW5:<br>- 32-33 weeks of GA □ aOR 1.49 (95% CI, 1.15 - 1.92; p = 0.0022)<br>- 34-36 weeks of GA □ aOR 1.30 (95% CI, 1.14 - 1.48; p < 0.0001)<br>- 37 weeks of GA □ not significative (p =0.1049)<br><br>The relative contribution of RSV infection to the overall predictive ability of the model was 6.6%, whereas the relative contribution of oxygen exposure was 4.9%. In contrast, the relative contribution of non-modifiable risk factors was 22.8% for sex, 4.8% for GA, 21.2% for race, and 28.7% for family history of asthma.	

Severe bronchiolitis in infancy: can asthma in adolescence be predicted?<br><br>Mikalsen IB, 2013<br><br>[8]	Prospective study	Follow-up at 2 and 11 years of age of infants hospitalized for bronchiolitis in the first year of life	Infants hospitalized with bronchiolitis	105 children younger than 12 months.<br>Of these:<br>-101 participated in the first follow-up at 2 years of age<br><br>-93 participated in the second follow-up at age 11.	To assess if clinical variables at 2 years of age could predict asthma at 11 years of age	Prevalence of asthma at 11 years of age was 22.6% and it was associated with recurrent wheeze at 2 years (OR: 7.2; 95% CI: 1.3, 41.6; P = 0.015), especially if combined with parental atopy, asthma or with atopic dermatitis	Recurrent wheeze at 2 years of age, together with family history positive for atopy or allergy, can predict the development of asthma at 11 years of age.
Lower respiratory tract infections associated with rhinovirus during infancy and increased risk of wheezing during childhood.<br><br>O'Callaghan-Gordo C, 2013<br><br>[9]	Prospective cohort study	Follow-up through data collection for up to 4 years and 9 months of children admitted for LRTI during a 12 months period.<br><br>Population was divided in two group according to presence of Rhinovirus or not in nasopharyngeal aspirates collected on admission	Infants admitted for lower respiratory tract infection (LRTI) and survived	220 infants younger than 1 year<br><br>25% of them with Rhinovirus infection	To evaluate whether LRTI hospitalization associated with rhinovirus during infancy is associated with an increased risk of wheezing.	Infants hospitalized with LRTI associated with Rhinovirus had higher incidence of visits due to wheezing within the year following hospitalization RR=1.68, (95% CI=1.02-2.75).<br><br>No evidence of increased incidence rate of visits with wheezing was observed for the remaining follow-up period.	There is a short term (1 year) increased risk of wheezing after an initial episode of lower respiratory tract infection due to Rhinovirus

Association between respiratory syncytial virus hospitalizations in infants and respiratory sequelae: systematic review and meta-analysis.<br><br>Régnier SA, 2013<br><br>[10]	Systematic review and Meta-analysis	Systematic research of literature published in MEDLINE and EMBASE databases of studies that assess the association between RSV hospitalization and asthma/wheezing later in life.	Children hospitalized for RSV-related disease	1533 children<br><br>up to 3 years of age	To assess the correlation between RSV hospitalization in early life and subsequent diagnosis of wheeze/asthma.	Incidence of asthma/wheezing was higher in children who had RSV disease in early life (OR: 3.84; 95% CI: 3.23-4.58).<br><br>The effect decreases with age.	Despite poor quality evidence, there is an association between RSV hospitalization in infancy and asthma/wheezing development, which decreases with age.

Early-life risk factors for childhood wheeze phenotypes in a high-risk birth cohort.<br><br>Lodge CJ, 2014<br><br>[11]	Birth-cohort study	Definition of a longitudinal childhood wheeze phenotypes in children up 7 years of age.<br><br>Investigation over the relationships of these phenotypes with earlylife exposures and wheeze at age 12 years (not consistent with pico question).	The Melbourne Atopy Cohort Study (MACS), a high-allergy-risk birth cohort that enrolled pregnant women from Melbourne, Australia between 1990 and 1994.	620 infants	Telephone surveys conducted with the infants' mothers every 4 weeks from birth to age 15 months, once at age 18 months, yearly at age 2-7 years, and once at 12 years. Mothers reported each time the days of cough, rattle, and wheeze experienced during the previous 4 weeks.<br><br>Potential early-life risk factors for wheeze reported:<br>- parental asthma, education, and smoking;<br>- infant sex and 4-week weight;<br>- breastfeeding;<br>- child atopy and early eczema;<br>- exposure to cats and/or dogs;<br>- lower respiratory tract infection (LRTI) in the first year;<br>- first-born status;<br>- childcare attendance by age 12 months	- Lower respiratory tract infection (LRTI) by 1 year (relative risk [RR], 3.00; 95% CI, 1.58-5.70), childcare by 1 year (RR, 1.51; 95% CI, 1.02-2.22), and higher body mass index (RR, 2.51; 95% CI, 1.09-5.81) were associated with increased risk of early transient wheeze.<br><br>- Breastfeeding was protective (RR, 0.54; 95% CI, 0.32-0.90).<br><br>- LRTI (RR, 6.54; 95% CI, 2.55-16.76) and aeroallergen sensitization (RR, 4.95; 95% CI, 1.74-14.02) increased the risk of early persistent wheeze.<br><br>- LRTI (RR, 5.31; 95% CI, 2.71-10.41), eczema (RR, 2.77; 95% CI, 1.78-4.31), aeroallergen sensitization (RR, 5.60; 95% CI, 2.86-10.9), and food sensitization (RR, 2.77; 95% CI, 1.56-4.94) increased the risk of intermediate-onset wheeze.<br><br>- Dog exposure at baseline (RR, 0.52; 95% CI, 0.32-0.84) and first-born status (RR, 0.49;	Evidence of novel insights into potential causative or aggravating environmental exposures for different childhood wheeze phenotypes.

					SPT used to test for cow's milk, egg white, peanut, house dust mite, rye grass, and cat dander allergies at age 1 and 2 years.	95% CI, 0.32-0.76) were protective.<br><br>- Heavy parental smoking at birth (RR, 3.18; 95% CI, 1.02-9.88) increased the risk of late-onset wheeze, whereas breastfeeding reduced it (RR, 0.34; 95% CI, 0.12-0.96).	

A longitudinal study on early hospitalized airway infections and subsequent childhood asthma.<br><br>Jeng MJ, 2015<br><br>[12]	Longitudinal study	To investigate the relationship between hospitalized airway infections (HAI) in young children (< 3 years old) and later childhood asthma	Children hospitalized for airway infections (HAI) and bronchiolitis compared to controls	3,264 children (1,981 with bronchiolitis; 1,283 with other HAIs) vs 18,527 controls<br><br>Younger than 3 years of age	To assess the relationship between hospitalized airway infections (HAI) and bronchiolitis in young children and asthma	Incidence of preschool asthma was higher:<br>-in bronchiolitis group compared to controls □ HR 1.583 (95% CI: 1.414-1.772)<br>- in other HAIs group compared to controls -_> HR 1.226 (95% CI: 1.053-1.428<br>-in bronchiolitis group compared to HAIs group □ HR 1.228 (95% CI: 1.075-1.542)<br>- in bronchiolitis group when affected also by congenital heart disease compared to controls □ OR (1.973, 95% CI: 1.193-3.263)	Children hospitalized before 3 years of age due to acute HAIs and especially bronchiolitis are at a higher risk of developing preschool asthma, especially if affected by congenital heart disease.

Incidence and characteristics of early childhood wheezing, Dhaka, Bangladesh, 2004-2010.<br><br>Dawood FS, 2016<br><br>[13]	Longitudinal observational study	To estimate wheezing incidence, describe wheezing phenotypes, and explore the contribution of respiratory viral illnesses	Children living in urban Bangladesh	N = 23.609<br><br>From birth to 5 years of age	During 2004–2010, respiratory illness surveillance was conducted through weekly home visits. Children with fever or respiratory illness were referred for examination by study physicians including lung auscultation. During 2005–2007, every fifth referred child had nasal washes tested for human metapneumovirus, respiratory syncytial viruses, and influenza and parainfluenza viruses.	A. Among children aged <5 years, incidences of wheezing and wheezing hospitalizations were 2,335/10,000 and 192/10,000 child-years.<br>28% had recurrent wheezing.<br><br>B1. Recurrent versus non-recurrent wheezing episodes were more likely to be associated with:<br>- oxygen saturation <93% (OR 6.9, 95%CI 2.8–17.3);<br>- increased work of breathing (OR 1.6, 95%CI 1.4–1.8);<br>- hospitalization (OR 2.0, 95%CI 1.6–2.4).<br><br>B2. Children with recurrent wheezing had episodes with a longer median duration of illness (6 vs. 8 days, P < 0.01).<br><br>B3. Children with recurrent wheezing at <12 months of age were at increased risk for recurrent wheezing at 24–35 months (RR 4.9, 95% CI 2.6–9.5), but overall, only 10% of children with recurrent wheezing at <12 months of age still had recurrent wheezing at 24–35 months of age.<br><br>C. Respiratory viruses were detected in 66% (578/873) of episodes with testing.	In urban Bangladesh, early childhood wheezing is common and largely associated with respiratory virus infections. Recurrent wheezing is associated with more severe illness and may predict children who would benefit most from closer follow-up and targeted interventions

Prevalence of rhinovirus in wheezing children: a comparison with respiratory syncytial virus wheezing.<br><br>Sun H, 2016<br><br>[14]	Prospective cohort study	Collection of clinical data (cough, fever, dyspnea, crackles) on the first day of admission and collection of results of nasopharyngeal aspirates (PCR to test rhinovirus, bocavirus, metapneumovirus and direct immunofluorescence assay to test respiratory syncytial virus, adenovirus, parainfluenza virus types 1-3, and influenza virus types A and B)	Consecutive hospitalized children presenting with wheezing	709<br><br>children younger than 60 months of age	To compare the clinical differences between rhinovirus- and respiratory syncytial virus-induced wheezing.	Prevalence of isolation:<br>-respiratory syncytial virus 21.0% (peak in winter)<br>-Rhinovirus 14.7% (peak in summer)<br>Rhinovirus-infected children experienced earlier wheezing more often than respiratory syncytial virus children (OR 3.441; 95% CI, 1.187-9.979; p=0.023).	Rhinovirus-infected children experienced earlier wheezing more often than respiratory syncytial virus children

Prevalence and risk factors for wheezing and allergic diseases in preschool children: A perspective from the Mediterranean coast of Turkey<br><br>Bolat E, 2017<br><br>[15]	Phase 1: prevalence study<br><br>Phase 2: case-control study	Determine the prevalence and risk Factors of respiratory And allergic diseases Using a modified ISAAC questionnaire In preschool children Attending daycare Centres in the city<br><br>Of Mersin.	All children Attending day-care Centres in the City centre Who were Randomly selected from The list of all Daycare centres in Mersin from January to December<br><br>2011.	396 preschool children<br>- 206 males<br>- 190 females<br><br>Mean age: 4.4± 0.9years.	**Phase 1.**<br>A modified ISAAC Questionnaire was used to assess the Symptoms of allergic And respiratory diseases, and the potential risk factors For the outcomes. The questionnaire Included questions About the symptoms And diagnosis of Respiratory diseases, eczema, food allergy And risk factors such As demographic characteristics, gestational factors, Family history, feeding practices, household Characteristics such As house crowding, Presence of pets, dampness, and tobacco Smoke exposure.<br><br>**Phase 2.**<br>Serum food and inhalant specific IgE, And skin tests were Performed in 45 children with frequent Wheezing and 28 Children with no<br><br>wheezing.	Significant risk factors for physician-diagnosed asthma:<br>- family history of atopy (OR=2.5, 95% CI: 1.3-4.7, p=0.004),<br>- dampness at home (OR=2.4, 95% CI: 1.2-4.8, p=0.008),<br>- a history of intestinal parasites (OR=4.3, 95% CI: 1.7-10.9, p=0.002),<br>- previous history of pneumonia (OR=6.9, 95% CI: 1.9-25.9, p=0.004),<br>- initiation of complementary foods before the age of three months (OR=6.1, 95%CI: 1.4-26.9, p=0.02)<br>- presence of food allergy (OR=3.1, 95% CI: 1.1-9.2, p=0.03)<br><br>The risk factors for frequent wheezing were:<br>- maternal smoking during pregnancy (OR=5.2, 95% CI: 0.9-28.7, p=0.05)<br>- high serum IgE levels (OR=2.9, 95% CI: 0.9-9.0, p=0.05) at borderline significance.	A high prevalence of asthma and allergic diseases, probably related to humid climatic properties in addition to other environmental and genetic factors was demonstrated.

Risk Factors for Virus-induced Acute Respiratory Tract Infections (ARTIs) in Children Younger Than 3 Years and Recurrent Wheezing at 36 Months Follow-Up After Discharge.<br><br>Nicolai A, 2017<br><br>[16]	Retrospective study	Retrospective data collection from clinical records of children hospitalized for ARTIs with nasopharyngeal specimen positive for a respiratory virus<br><br>Versus children with no history of respiratory diseases<br><br>Plus telephone interview of their parents collected at 12, 24 and 36 months after children's discharge, with a structured questionnaire on wheezing episodes	Children hospitalized for ARTIs<br><br>Versus children with no history of respiratory diseases	273 full-term children (median age, 2.9 months; range, 0.26-39; boys, 61.2%)<br><br>Vs 101 controls (median age, 8 months; range, 0.5-36.5; boys, 58.4%).	To detect risk factors for specific virus-induced acute respiratory tract infections (ARTIs) in children younger than 3 years old and for wheezing at 36-month follow-up.	The main risk factor for recurrent wheezing was exposure to tobacco smoke [OR: 2.5 (95% CI: 1.1-15.6)].<br><br>Risk factors for virus-induced ARTIs were:<br><br>-siblings [OR: 3.0 (95% CI: 1.8-5.2)]<br><br>- smoking cohabitants (OR: 2.3 (95% CI: 2-4.2)] especially for RSV infection[OR: 1.8 (95% CI: 1.1-3.2)].<br><br>-breastfeeding lasting less than 3 months [OR: 0.5 (95% CI: 0.3-0.9)].<br><br>Risk factors for human rhinovirus-induced ARTIs were:<br><br>-attending day-care [OR: 5.0 (95% CI: 2.3-10.6)]<br><br>-high eosinophil blood counts [OR: 2.6 (95% CI: 1.2-5.7)].	The exposure to tobacco smoke is one of the main risk factors for recurrent wheezing and respiratory tract infection in children

Bronchiolitis in young infants: is it a risk factor for recurrent wheezing in childhood?<br><br>Rinawi F, 2017<br><br>[17]	Retrospective study	To evaluate prevalence, clinical manifestations and risk factors for recurrent wheezing in children younger than 3 years and for persistent wheezing beyond this age in children hospitalized for bronchiolitis in infancy	Children hospitalized for bronchiolitis before 6 months of age Vs population-based controls with no bronchiolitis in the first 6 months of life	150 children 6 years old hospitalized for bronchiolitis before 6 moths of life<br>Vs 66 controls	Retrospective data (epidemiology, prevalence, age at onset, number of and treatments for wheezing episodes, pathogens detected, and severity of bronchiolitis) were collected by pediatricians up to 6 years of age about children hospitalized for bronchiolitis in the first 6 months of life	Hospitalization due to acute bronchiolitis in the first 6 months of life is a risk factor for recurrent wheezing OR 4.910 (2.2-11.7), p=0.001	Hospitalization for bronchiolitis within the first 6 months of life is a risk factor for recurrent wheezing episodes in children younger than 3 years

Prevalence estimates and risk factors for early childhood wheeze across Europe: the EuroPrevall birth cohort<br><br>Selby A, 2018<br><br>[18]	Birth cohort study	To assess the prevalence of early childhood wheeze across Europe and evaluate risk factors focusing on food allergy, breast feeding and smoke exposure.	Infants from nine countries were recruited into the EuroPrevall birth cohort: Reykjavik (Iceland), Southampton (UK), Amsterdam (The Netherlands), Berlin (Germany), Lodz (Poland), Vilnius (Lithuania), Madrid (Spain), Milan (Italy) and Athens (Greece).	8805 infants with 24-months follow up data	Evaluation began at birth with follow-up of participants at 12 and 24 months using standardised questionnaires based on those used in previous epidemiological studies such as ISAAC.<br><br>At recruitment, data were collected on birth details, maternal diet, family history, maternal education (as a marker of socioeconomic status) and environmental exposures, including cigarette smoke and pet ownership. The 12-month and 24-month questionnaires included an extensive list of foods found in children's diets.<br><br>Additional assessments, including skin prick testing, measurement of specific IgE with or without a DBPCFC were performed according to a standardised protocol whenever	The prevalence of wheeze in the second year of life ranged:<br>- from <2% in Lodz (Poland) and Vilnius (Lithuania)<br>- to 13.1% (95% CI 10.7% to 15.5%) in Southampton (UK) and 17.2% (95% CI 15.0% 19.5%) in Reykjavik (Iceland).<br><br>In multivariable analysis, Factors associated with wheeze:<br>- frequent lower respiratory tract infections in the first and second years of life (incidence rate ratio (IRR) 1.9 (95% CI 1.3 to 2.6) and 2.5 (95% CI 1.9 to3.4), respectively),<br>- postnatal maternal smoking (IRR 1.6, 95% CI 1.1 to 2.4),<br>- day care attendance (IRR 1.6, 95% CI 1.1 to 2.5)<br>- male gender (IRR 1.3, 95% CI 1.0 to 1.7).<br><br>Food allergy and breast feeding were not independently associated with wheeze.	The prevalence of early childhood wheeze varied considerably across Europe. Lower respiratory tract infections, day care attendance, postnatal smoke exposure and male gender are important risk factors.

					parents reported symptoms suggestive of food allergy in their children.1		

Severe bronchiolitis profiles and risk of recurrent wheeze by age 3 years.<br><br>Dumas O, 2019<br><br>[19]	Retrospective study	To compare the risk of developing wheezing among different profiles of hospitalized children for bronchiolits	Infants hospitalized for bronchiolitis in 17 hospitals (USA) during winter (2011-2014)<br><br>with post-hospitalization follow-up.	921 infants younger than 1 years	Retrospective collection of data of a cohort of infants hospitalized for bronchiolitis and followed-up post-hospitalization.<br><br>Patients were divided in 3 profiles:<br><br>Profile A: history of breathing problems/eczema and non-RSV infection<br><br>Profile B: history of RSV infection<br><br>Profile C: severe ill infants	Profile A has higher risk of recurrent wheeze compared to profile B (HR, 2.64; 95% CI, 1.90-3.68) and to profile C (HR 1.51; 95% CI, 1.14-2.01).	Among infants hospitalized for bronchiolitis, those with history of breathing problems/eczema and non-RSV infection seems to be at higher risk of asthma.

Association of Rhinovirus C Bronchiolitis and Immunoglobulin E Sensitization During Infancy With Development of Recurrent Wheeze.<br><br>Hasegawa K, 2019<br><br>[20]	Multicentre prospective cohort study	Prospective follow-up of infants hospitalized for bronchiolitis due to RSV and rhinovirus species (A, B, and C).	Infants younger than 1 year hospitalized for bronchiolitis in 17 hospitals (USA) during 3 consecutive fall to winter seasons (2011-2014).	716 infants hospitalized for RSV-only or rhinovirus bronchiolitis. Median age: 2.9 months (interquartile range, 1.6-3.8 months),	To assess the risk to develop recurrent wheeze by age 3 years, after bronchiolitis caused by respiratory syncytial virus and rhinovirus species	Infants with Rhinovirus C bronchiolitis showed higher risk to develop recurrent wheeze compared to RSV group (HR, 1.58; 95% CI, 1.08-2.32), while no significant difference were found among RSV and Rhinovirus A (HR, 1.27; 95% CI, 0.86-1.88) and B (HR, 1.39; 95% CI, 0.51-3.77) .<br><br>Infants with both rhinovirus C and IgE sensitization (to food or aeroallergens) during infancy had higher risks of recurrent wheeze (HR, 3.03; 95% CI, 1.20-7.61). and of development of asthma at age 4 years (HR, 4.06; 95% CI, 1.17-14.1).	Infants with Rhinovirus C bronchiolitis, especially if with IgE sensitization, show the highest risk to develop recurrent wheeze and asthma.

Preschool respiratory hospital admissions following infant bronchiolitis: a birth cohort study.<br><br>Skirrow H, 2019<br><br>[21]	Retrospective population-based birth cohort study	To assess the risk of respiratory hospital admission in children under 5 years following bronchiolitis admission in infancy	Respiratory hospital admission in children previously admitted for bronchiolitis Versus controls	16288 children younger than 5 previously admitted for bronchiolitis vs controls without previous admission for bronchiolitis	Retrospective data collection about birth cohort of 613 377 infants born between 1 April 2007 and 31 March 2008 in NHS hospital in England	By age 5 years, respiratory hospital admission rate is higher in those previously admitted for bronchiolitis than in controls (HR 2.82, 95% CI 2.72 to 2.92), especially for asthma (HR 4.35, 95% CI 4.00 to 4.73) and wheezing admissions (HR 5.02, 95% CI 4.64 to 5.44)	Hospital admission for bronchiolitis in infancy is associated with higher risk of subsequent respiratory hospital admissions in children younger than 5

Association between respiratory syncytial virus hospitalization in infancy and childhood asthma.<br><br>Coutts J, 2020<br><br>[22]	Retrospective study	To compare asthma hospitalization, admission rate and medication usage in children hospitalized for RSV infection before 2 years of age compare to controls	All children born in National Health Service Scotland between 1996 and 2011 were included.	740.418 children (median follow-up: 10.6 years), of which 15.795 (2.1%) with respiratory syncytial virus hospitalization at ≤2 years (median age: 143 days)	Data collection about asthma hospital admissions and medication use through 18 years with the aim to compare children with (cases) and without (controls) RSV hospitalization in the first 2 years of life.	In children hospitalized for RSV infection compared with controls:<br>-asthma hospitalizations were higher (8.4% vs 2.4%; RR: 3.3, 95% CI: 3.1-3.5; p < .0001)<br>- admission rates were higher (193.2 vs 46.0/1000)<br>- medication usage was higher (25.5% vs 14.7%; relative risk: 1.7, 95% CI: 1.7-1.8; p < .0001)<br>- having both an asthma admission and medication usage was even higher (4.8% vs 1.5%; relative risk 3.1, 95% CI: 2.9-3.3; P < .0001).<br>- admission rates and medication use remained significantly (p < .001) higher throughout childhood<br><br>- RSV hospitalization was the most significant risk factor for asthma hospitalizations±medication use (OR: 1.9-2.8; p < .001).	RSV hospitalization is associated with increased rate of subsequent admissions, medication usage and asthma.

Cumulative incidence of post-infection asthma or wheezing among young children clinically diagnosed with respiratory syncytial virus infection in the United States: A retrospective database analysis.<br><br>Nguyen-Van-Tam J, 2020<br><br>[23]	Retrospective study	To estimate the cumulative incidence of asthma/wheezing following RSV infection at 1, 3, and 5 years of follow-up	Children with RSV infection before 2 years of life, with follow-up at 1,3 and 5 years, from January 2007 until March 2016.<br><br>The three cohorts were stratified by risk factor such as pre-term birth and pre-existing comorbidities.	Children younger than 2 years with RSV infection, with and without high risk factors (pre-term birth and pre-defined, pre-existing comorbidities), follow-up at 1, 3 and 5 years.<br><br>Cohorts without high risk factors:<br>-1 y(N:9811)<br>-3 y(N:4524)<br>-5 y(N:1788)<br>Cohorts with high risk factors:<br>-1y (N:3030)<br>-3y (N:1378)<br>-5y (N:552)	Restrospective data collection from Optum® integrated electronic health records and database	Risk of wheezing/asthma in RSV hospitalization vs RSV non-hospitalized in infants without high risk factors at<br>-1y OR 2.1 (1.8-2.6)<br>-3y OR 2.3 (1.8-2.9)<br>-5y OR 2.7 (1.8-4.0)<br><br>Risk of wheezing/asthma in RSV hospitalization vs RSV non-hospitalized in infants with high risk factors at:<br>-1y OR 2.4 (1.8-3.0)<br>-3y OR 2.0 (1.4-2.8)<br>-5y OR 1.7 (1.0-3.0)	Compared with non-hospitalized infants, hospitalization due to RSV-infection is associated with an increased risk to develop asthma or wheezing for at least 5 years.

Bronchiolitis severity is related to recurrent wheezing by age 3 years in a prospective, multicenter cohort.<br><br>Mansbach JM, 2020<br><br>[24]	Prospective multicentric study	Incidence of recurrent wheezing by age 3 years and asthma by age 4 years.	Infants hospitalized for bronchiolitis in 17 hospitals (US) during winter (2011-2014)<br>with post-hospitalization follow-up.<br>(MARC-35 cohort)	921 infants median age at enrollment: 3.2 months (IQR 1.6–5.9 months)<br><br>89% of them had follow-up at 3 years.	Prospective multicentric study of infants hospitalized for bronchiolitis needed intensive care<br>Vs<br>Not,<br>with post-hospitalization follow-up.	Who required intensive care present higher risk to develop recurrent wheezing by age 3 years is significantly higher in (HR 1.45; 95%CI 1.24–1.69) and asthma at age 4 years (OR 1.62; 95%CI 1.02–2.59).	Intensive care treatment is associated with increased risk of recurrent wheezing by age 3 years and asthma by age 4 years.
Association Between Respiratory Syncytial Virus-Associated Acute Lower Respiratory Infection in Early Life and Recurrent Wheeze and Asthma in Later Childhood.<br><br>Shi T, 2020<br><br>[25]	Systematic review	To assess the association between RSV infection in infancy and respiratory sequelae up to age 12 years	Data are collected from 41 studies published between January 1995 and May 2018.	234.000 children	3 control groups and 3 follow-up age groups with previous RSV infection	Early life RSV infection is associated with childhood recurrent wheeze at:<br>- 0 to <36 months: OR 3.05 (95% CI, 2.50-3.71)<br>- 36 to 72 months: OR 2.60 (95% CI, 1.67-4.04)<br>- 73 to 144 months: OR 2.14 (95% CI, 1.33-3.45)<br>The risk to develop asthma is statistically significative for those aged 73-144 months at follow-up: OR 2.95 (95% CI, 1.96-4.46).	RSV infection is associated with recurrent wheeze development

Early childhood respiratory morbidity and antibiotic use in ex-preterm infants: a primary care population-based cohort study.<br><br>Tan S, 2020<br><br>[26]	Retrospective cohort study	To examine early childhood rates of primary care consultations for respiratory tract infections (RTI), lower respiratory tract infections (LRTI), wheeze and antibiotic prescriptions in ex-preterm and term children. A secondary aim was to examine differences between preterm infants discharged home with or without oxygen.	Children born between 1997 and 2014.	253 277 eligible children, with 1666 born preterm at <32 weeks' gestation, followed-up from primary care registration to age 5 years.	For primary analysis, we identified two cohorts of newborn infants: 1) term infants born ⩾37 completed weeks of gestation, with birthweights ⩾2500 g and without prescriptions for home oxygen preterm infants born before 32 completed weeks of gestation with birthweights <1500 g ("preterm").<br><br>The preterm group was further divided into two cohorts for secondary subgroup analysis: those with (PT-O2) and without (PT-Air) home oxygen prescriptions in their medical records within 3 months of registration at their general practice.<br>Outcome<br><br>The primary outcomes were:<br>rates of primary care consultations for three respiratory conditions:<br>respiratory tract infection (RTI, including both upper and lower RTIs), lower	Adjusted incidence rate ratios (aIRRs) for respiratory tract infections RTI (1.37, 95% CI 1.33–1.42), lower respiratory tract infections LRTI (2.79, 95% CI 2.59–3.01), wheeze (3.05, 95% CI 2.64–3.52) and antibiotic prescriptions (1.49, 95% CI 1.44–1.55) were higher for ex-preterm infants.<br><br>Ex-preterm infants discharged home on oxygen had significantly greater morbidity across all respiratory diagnoses and antibiotic prescriptions compared to those without home oxygen.	Ex-preterm infants, particularly those with BPD requiring home oxygen, have significant respiratory morbidity and antibiotic prescriptions in early childhood.

					respiratory tract infection specifically (LRTI) and wheeze; rates of antibiotic prescriptions.		

Rhinovirus bronchiolitis, maternal asthma, and the development of asthma and lung function impairments<br><br>Da Silva Sena CR, 2021<br><br>[27]	Prospective birth cohort study	To investigate the combined effects of hospitalization for RV positive bronchiolitis in infancy and a history of maternal asthma on the development of asthma at preschool age.	Preschool-aged children, with a history of hospital admission for bronchiolitis in infancy	N = 139<br><br>(64.8% males)<br><br>Age at admission in months: 7 (2-11)<br><br>Age at a visit in months: 47 (42-51)	A follow-up to ascertain asthma and asthma-like symptoms was conducted, along with skin prick allergy test positivity, and lung function measured pre- and post-bronchodilator using impulse oscillometry.	A. Children with a past hospitalization for RV positive bronchiolitis (42.4% of all) and a history of maternal asthma (36.7% of all) had the greatest prevalence and risk ratio (RR) for<br>- doctor-diagnosed asthma [prevalence 81.8%; RR 2.10 (95%CI 1.37–3.19, p = .001)];<br>- use of inhaled corticosteroids [68.2%; RR 2.17, 95% CI 1.19–3.99, p = .001)];<br>- short-acting β-agonists in the last 12 months [(95.2% and RR 1.49, 95% CI 1.17–1.89, p = .001)]<br>as compared to those with RV negative bronchiolitis and no maternal asthma history.<br><br>B. More children in this group had an abnormal airway resistance (33.3% and adjusted risk ratio [aRR] 3.11, 95% CI 1.03–9.47, p = .045) and reactance (27.8% and aRR 2.11, 95% CI 1.06–4.26, p = .035) at 5 Hz, measured with impuls oscillometry, as compared to those with RV negative bronchiolitis and no maternal asthma history.	Hospitalization for RV positive bronchiolitis in early life combined with a history of maternal asthma identifies a subgroup of children with a high asthma burden while participants with only one of the two risk factors had intermediate risk for asthma.

Association between early viral LRTI and subsequent wheezing development, a meta-analysis and sensitivity analyses for studies comparable for confounding factors.<br><br>Kenmoe S, 2021<br><br>[28]	Meta-analysis	Meta-analysis and sensitivity analysis of 22 cohort studies comparing the frequency of subsequent wheezing in children with and without LRTI in childhood.			To establish the association between viral LRTI at ≤ 5 years of age and the development of wheezing in adolescence or adulthood	Viral LRTI in children ≤ 3 years is associated with increased risk of wheezing (OR = 3.1, 95% CI = 2.4-3.9).<br><br>This risk is conserved when considering studies with comparable groups for socio-demographic and clinical confounders.	Viral LRTI before 3 years of age is associated to an increased risk to develop wheezing.

References

1. Jartti T, Kuusipalo H, Vuorinen T, Söderlund-Venermo M, Allander T, Waris M, Hartiala J, Ruuskanen O. Allergic sensitization is associated with rhinovirus-, but not other virus-, induced wheezing in children. Pediatr Allergy Immunol. 2010 Nov;21(7):1008-14. doi: 10.1111/j.1399-3038.2010.01059.x. PMID: 20977499; PMCID: PMC7168081.
2. Romero JR, Stewart DL, Buysman EK, Fernandes AW, Jafri HS, Mahadevia PJ. Serious early childhood wheezing after respiratory syncytial virus lower respiratory tract illness in preterm infants. Clin Ther. 2010 Dec;32(14):2422-32. doi: 10.1016/j.clinthera.2011.01.007. PMID: 21353110.
3. Kovesi TA, Cao Z, Osborne G, Egeland GM. Severe early lower respiratory tract infection is associated with subsequent respiratory morbidity in preschool Inuit children in Nunavut, Canada. J Asthma. 2011 Apr;48(3):241-7. doi: 10.3109/02770903.2011.560320. PMID: 21391880.
4. Bacharier LB, Cohen R, Schweiger T, Yin-Declue H, Christie C, Zheng J, Schechtman KB, Strunk RC, Castro M. Determinants of asthma after severe respiratory syncytial virus bronchiolitis. J Allergy Clin Immunol. 2012 Jul;130(1):91-100.e3. doi: 10.1016/j.jaci.2012.02.010. Epub 2012 Mar 22. PMID: 22444510; PMCID: PMC3612548.
5. Lin HW, Lin SC. Environmental factors association between asthma and acute bronchiolitis in young children--a perspective cohort study. Eur J Pediatr. 2012 Nov;171(11):1645-50. doi: 10.1007/s00431-012-1788-3. Epub 2012 Jul 10. PMID: 22777642.
6. Cox DW, Bizzintino J, Ferrari G, Khoo SK, Zhang G, Whelan S, Lee WM, Bochkov YA, Geelhoed GC, Goldblatt J, Gern JE, Laing IA, Le Souëf PN. Human rhinovirus species C infection in young children with acute wheeze is associated with increased acute respiratory hospital admissions. Am J Respir Crit Care Med. 2013 Dec 1;188(11):1358-64. doi: 10.1164/rccm.201303-0498OC. PMID: 23992536; PMCID: PMC5447292.
7. Escobar GJ, Masaquel AS, Li SX, Walsh EM, Kipnis P. Persistent recurring wheezing in the fifth year of life after laboratory-confirmed, medically attended respiratory syncytial virus infection in infancy. BMC Pediatr. 2013 Jun 19;13:97. doi: 10.1186/1471-2431-13-97. PMID: 23782528; PMCID: PMC3703269.
8. Mikalsen IB, Halvorsen T, Eide GE, Øymar K. Severe bronchiolitis in infancy: can asthma in adolescence be predicted? Pediatr Pulmonol. 2013

Jun;48(6):538-44. doi: 10.1002/ppul.22675. Epub 2012 Sep 13. PMID: 22976850.
9. O'Callaghan-Gordo C, Bassat Q, Díez-Padrisa N, Morais L, Machevo S, Nhampossa T, Quintó L, Alonso PL, Roca A. Lower respiratory tract infections associated with rhinovirus during infancy and increased risk of wheezing during childhood. A cohort study. PLoS One. 2013 Jul 31;8(7):e69370. doi: 10.1371/journal.pone.0069370. PMID: 23935997; PMCID: PMC3729956.
10. Régnier SA, Huels J. Association between respiratory syncytial virus hospitalizations in infants and respiratory sequelae: systematic review and meta-analysis. Pediatr Infect Dis J. 2013 Aug;32(8):820-6. doi: 10.1097/INF.0b013e31829061e8. PMID: 23518824.
11. Lodge CJ, Zaloumis S, Lowe AJ, Gurrin LC, Matheson MC, Axelrad C, Bennett CM, Hill DJ, Hosking CS, Svanes C, Abramson MJ, Allen KJ, Dharmage SC. Early-life risk factors for childhood wheeze phenotypes in a high-risk birth cohort. J Pediatr. 2014 Feb;164(2):289-94.e1-2. doi: 10.1016/j.jpeds.2013.09.056. Epub 2013 Nov 14. PMID: 24238860.
12. Jeng MJ, Lee YS, Tsao PC, Yang CF, Soong WJ. A longitudinal study on early hospitalized airway infections and subsequent childhood asthma. PLoS One. 2015 Apr 28;10(4):e0121906. doi: 10.1371/journal.pone.0121906. PMID: 25919024; PMCID: PMC4412821.
13. Dawood FS, Fry AM, Goswami D, Sharmeen A, Nahar K, Anjali BA, Rahman M, Brooks WA. Incidence and characteristics of early childhood wheezing, Dhaka, Bangladesh, 2004-2010. Pediatr Pulmonol. 2016 Jun;51(6):588-95. doi: 10.1002/ppul.23343. Epub 2015 Nov 27. PMID: 26613245.
14. Sun H, Sun Q, Jiang W, Chen Z, Huang L, Wang M, Ji W, Shao X, Yan Y. Prevalence of rhinovirus in wheezing children: a comparison with respiratory syncytial virus wheezing. Braz J Infect Dis. 2016 Mar-Apr;20(2):179-83. doi: 10.1016/j.bjid.2015.12.005. Epub 2016 Feb 7. PMID: 26859065.
15. Bolat E, Arikoglu T, Sungur MA, Batmaz SB, Kuyucu S. Prevalence and risk factors for wheezing and allergic diseases in preschool children: A perspective from the Mediterranean coast of Turkey. Allergol Immunopathol (Madr). 2017 Jul-Aug;45(4):362-368. doi: 10.1016/j.aller.2017.01.002. Epub 2017 Apr 11. PMID: 28410872.
16. Nicolai A, Frassanito A, Nenna R, Cangiano G, Petrarca L, Papoff P, Pierangeli A, Scagnolari C, Moretti C, Midulla F. Risk Factors for Virus-induced Acute Respiratory Tract Infections in Children Younger Than 3 Years and Recurrent Wheezing at 36 Months Follow-Up After Discharge. Pediatr Infect Dis J. 2017 Feb;36(2):179-183. doi: 10.1097/INF.0000000000001385. PMID: 27798551.
17. Rinawi F, Kassis I, Tamir R, Kugelman A, Srugo I, Miron D. Bronchiolitis in young infants: is it a risk factor for recurrent wheezing in childhood? World J Pediatr. 2017 Feb;13(1):41-48. doi: 10.1007/s12519-016-0056-4. Epub 2016 Sep 15. PMID: 27650524.
18. Selby A, Munro A, Grimshaw KE, Cornelius V, Keil T, Grabenhenrich L, Clausen M, Dubakiene R, Fiocchi A, Kowalski ML, Papadopoulos NG, Reche M, Sigurdardottir ST, Sprikkelman AB, Xepapadaki P, Mills ENC, Beyer K, Roberts G. Prevalence estimates and risk factors for early childhood wheeze across Europe: the EuroPrevall birth cohort. Thorax. 2018 Nov;73(11):1049-1061. doi: 10.1136/thoraxjnl-2016-209429. Epub 2018 May 10. PMID: 29748253.
19. Dumas O, Hasegawa K, Mansbach JM, Sullivan AF, Piedra PA, Camargo CA Jr. Severe bronchiolitis profiles and risk of recurrent wheeze by age 3 years. J Allergy Clin Immunol. 2019 Apr;143(4):1371-1379.e7. doi: 10.1016/j.jaci.2018.08.043. Epub 2018 Sep 18. PMID: 30240701; PMCID: PMC6422772.
20. Hasegawa K, Mansbach JM, Bochkov YA, Gern JE, Piedra PA, Bauer CS, Teach SJ, Wu S, Sullivan AF, Camargo CA Jr. Association of Rhinovirus C Bronchiolitis and Immunoglobulin E Sensitization During Infancy With Development of Recurrent Wheeze. JAMA Pediatr. 2019 Jun 1;173(6):544-552. doi: 10.1001/jamapediatrics.2019.0384. PMID: 30933255; PMCID: PMC6547078.
21. Skirrow H, Wincott T, Cecil E, Bottle A, Costelloe C, Saxena S. Preschool respiratory hospital admissions following infant bronchiolitis: a birth cohort study. Arch Dis Child. 2019 Jul;104(7):658-663. doi: 10.1136/archdischild-2018-316317. Epub 2019 Mar 6. PMID: 30842095.
22. Coutts J, Fullarton J, Morris C, Grubb E, Buchan S, Rodgers-Gray B, Thwaites R. Association between respiratory syncytial virus hospitalization in infancy and childhood asthma. Pediatr Pulmonol. 2020 May;55(5):1104-1110. doi: 10.1002/ppul.24676. Epub 2020 Feb 10. PMID: 32040885; PMCID: PMC7187471.
23. Nguyen-Van-Tam J, Wyffels V, Smulders M, Mazumder D, Tyagi R, Gupta N, Gavart S, Fleischhackl R. Cumulative incidence of post-infection asthma or wheezing among young children clinically diagnosed with respiratory syncytial virus infection in the United States: A retrospective database analysis.

Influenza Other Respir Viruses. 2020 Nov;14(6):730-738. doi: 10.1111/irv.12770. Epub 2020 Jun 12. PMID: 32533658; PMCID: PMC7578296.

24. Mansbach JM, Hasegawa K, Geller RJ, Espinola JA, Sullivan AF, Camargo CA Jr; MARC-35 Investigators. Bronchiolitis severity is related to recurrent wheezing by age 3 years in a prospective, multicenter cohort. Pediatr Res. 2020 Feb;87(3):428-430. doi: 10.1038/s41390-019-0589-z. Epub 2019 Oct 4. PMID: 31585458; PMCID: PMC7035967.
25. Shi T, Ooi Y, Zaw EM, Utjesanovic N, Campbell H, Cunningham S, Bont L, Nair H; RESCEU Investigators. Association Between Respiratory Syncytial Virus-Associated Acute Lower Respiratory Infection in Early Life and Recurrent Wheeze and Asthma in Later Childhood. J Infect Dis. 2020 Oct 7;222(Suppl 7):S628-S633. doi: 10.1093/infdis/jiz311. PMID: 31370064.
26. Tan S, Szatkowski L, Moreton W, Fiaschi L, McKeever T, Gibson J, Sharkey D. Early childhood respiratory morbidity and antibiotic use in ex-preterm infants: a primary care population-based cohort study. Eur Respir J. 2020 Jul 30;56(1):2000202. doi: 10.1183/13993003.00202-2020. PMID: 32366482.
27. Da Silva Sena CR, Morten M, Meredith J, Kepreotes E, E Murphy V, G Gibson P, D Robinson P, D Sly P, Whitehead B, Karmaus W, Collison A, Mattes J. Rhinovirus bronchiolitis, maternal asthma, and the development of asthma and lung function impairments. Pediatr Pulmonol. 2021 Feb;56(2):362-370. doi: 10.1002/ppul.25165. Epub 2020 Nov 24. PMID: 33179407.
28. Kenmoe S, Bowo-Ngandji A, Kengne-Nde C, Ebogo-Belobo JT, Mbaga DS, Mahamat G, Demeni Emoh CP, Njouom R. Association between early viral LRTI and subsequent wheezing development, a meta-analysis and sensitivity analyses for studies comparable for confounding factors. PLoS One. 2021 Apr 15;16(4):e0249831. doi: 10.1371/journal.pone.0249831. PMID: 33857215; PMCID: PMC8049235.

Question 3. Does pollution influence the onset and the evolution of preschool wheezing?

Search strategies:

("child, preschool"[MeSH Terms] OR "toddler*"[All Fields]) AND ("respiratory sounds"[MeSH Terms] OR "wheez*"[All Fields] OR "Asthma"[MeSH Terms:noexp]) AND "Risk Factors"[MeSH Terms] AND ("Age of Onset"[MeSH Terms] OR "Onset"[All Fields]) AND "english"[Language] AND 2008/01/01:2021/12/31[Date - Publication]

("child, preschool"[MeSH Terms] OR "toddler*"[All Fields]) AND ("respiratory sounds"[MeSH Terms] OR "wheez*"[All Fields] OR "Asthma"[MeSH Terms:noexp]) AND "Risk Factors"[MeSH Terms] AND ("disease progression"[MeSH Terms] OR "disease evolution"[All Fields] OR "evolution"[All Fields]) AND "english"[Language] AND 2008/01/01:2021/12/31[Date - Publication]

**Title of the study, first author, year**	**Type of study**	**Objective**	**Population**	**N of patients, Age**	**Experiments/mechanisms assessed**	**Relative risk (RR) or odds ratio (OR) + confidence interval (CI 95%)**	**Results**

Traffic-related air pollution and childhood respiratory Symptoms, function and allergies<br><br>Nordling E, 2008<br><br>[1]	Prospective birth-cohort study	To assess the impact of exposure to source-specific air pollutants during the first year of life on wheezing, lung function and sensitization in children at the age of 4 years.	Birth cohort exposed to moderate levels of air pollution, and with detailed modeling of exposure to air pollution from local traffic and house-heating (predefined areas in 4 Swedish municipalities, representing urban and suburban environments)	N = 4089<br><br>From birth to 4 years of age	Data on parental allergic diseases, pet contact, detailed residential characteristics and socio-economic factors were collected with a postal questionnaire to the parents at recruitment (median child age = 2 months). When the children were approximately 1, 2, and 4-year-old, parents received similar questionnaires, with a main focus on the children's symptoms related to wheezing and allergic diseases, and information on exposure factors.<br><br>At approximately 4 years of age, 2965 children (73% of the full cohort) attended a clinical investigation in our department, including lung function test and blood sampling.	Exposure to air pollution from traffic during the first year of life was associated with an excess risk of persistent wheezing (OR for 44 microg/m3 [5th-95th percentile] difference in traffic-NOx = 1.60; 95% CI= 1.09-2.36).<br><br>Similar results were found for sensitization (measured as specific IgE) to inhalant allergens, especially pollen (OR for traffic-NOx = 1.67; 95% CI = 1.10-2.53), at the age of 4 years.<br><br>Results were similar using traffic-NOx and traffic-PM10 as indicators.	Exposure to moderate levels of locally emitted air pollution from traffic early in life appears to influence the development of airway disease and sensitization in preschool children

Impact of air pollution on respiratory diseases in children with recurrent wheezing or asthma<br><br>Esposito S, 2014<br><br>[2]	Prospective longitudinal study	To evaluate the effects of traffic-related pollution on the exacerbation of asthma and development of respiratory infections children suffering from asthma or wheezing compared with healthy subjects; to estimate the association between incremental increases in principal pollutants and the incidence of respiratory symptoms.	Italian children suffering from asthma or wheezing	N = 777 (375 with recurrent wheezing or asthma; 402 healthy subjects n= 155 from 2-5 years of age with wheezing/asthma)<br><br>From 2 to 18 years of age	Over 12 months, parents filled out a daily clinical diary to report information about respiratory symptoms, type of medication used and healthcare utilization.<br><br>Clinical data were combined with the results obtained using an air pollution monitoring system of the five most common pollutants.	Living close to a street with a high density of traffic was a risk factor for asthma exacerbations (OR = 1.79; 95% CI, 1.13-2.84);<br><br>Living near green areas was protective (OR = 0.50; 95% CI, 0.31 -0.80).<br><br>In the recurrent wheezing/asthmatic children, an increase of 10 μg/m^3 of PM_{10} and NO_2 increased the incidence of pneumonia (continuous RR = 1.08, 95% CI: 1.00-1.17 for PM_{10}; continuous RR = 1.08, 95% CI: 1.01-1.17 for NO_2).	Significant association between traffic-related pollution and the development of asthma exacerbations and respiratory infections in children suffering from recurrent wheezing or asthma.<br><br>Environmental control may be crucial for respiratory health in children with underlying respiratory disease

Timing and Duration of Traffic-related Air Pollution Exposure and the Risk for Childhood Wheeze and Asthma<br><br>Brunst KJ, 2015<br><br>[3]	Prospective birth cohort study	To evaluate the timing and duration of traffic-related air pollution (TRAP) exposure as a risk factor for childhood wheezing and asthma development	Children born to at least one atopic parent were recruited from 2001 to 2003 from CCAAPS (Cincinnati Childhood Allergy and Air Pollution Study)	N = 617<br><br>From birth to 7 years of age	Children completed clinical examinations annually from age 1 year through age 4 years and age 7 years. Parental-reported wheezing was assessed at each age, and longitudinal wheezing phenotypes (early-transient, late-onset, persistent) and asthma were defined at age 7 years.<br><br>Participants time-weighted exposure to TRAP, from birth through age 7 years, was estimated using a land-use regression model. The relationship between TRAP exposure and wheezing phenotypes and asthma was examined.	High TRAP exposure at birth was significantly associated with both transient and persistent wheezing phenotypes □ ([aOR] = 1.64; 95% CI 1.04-2.57 and aOR = 2.31; 95% CI, 1.28-4.15, respectively).<br><br>Exposure from birth to age 1 year and age 1 to 2 years was also associated with persistent wheeze □ ([aOR] = 1.89; 95% CI 1.05-3.40)<br><br>Average time of TRAP exposure from birth to age 4 years associated with persistent wheeze □ [aOR] = 1.98; 95% CI 1.09-3.57<br><br>Only children with high average TRAP exposure from birth through age 7 years were at significantly increased risk for asthma (aOR = 1.71; 95% CI, 1.01-2.88).	Early-life exposure to TRAP is associated with increased risk for persistent wheezing.<br><br>Only long-term exposure to high levels of TRAP throughout childhood was associated with asthma development.

Prenatal exposure to bisphenol A and phthalates and childhood respiratory tract infections and allergy.<br><br>Gascon M, 2015<br><br>[4]	Prospective birth cohort study	To evaluate whether prenatal exposure to bisphenol A (BPA) and phthalates increases the risk of respiratory and allergic outcomes in children at various ages from birth to 7 years.	Mother-child pair participating in the Infancia y Medio Ambiente-Sabadell birth cohort studyl	Pregnant women = 657<br><br>From birth to 7 years of age	Measurements of BPA and metabolites of high-molecular-weight phthalates, 4 di-(2-ethylhexyl) phthalate (DEHP) metabolites (Σ4DEHP) and mono-benzyl phthalate (MBzP), and 3 low-molecular-weight phthalate (LMWP) metabolites (Σ3LMWP) in urine samples were collected during the first and third trimesters in pregnant women. The occurrence of chest infections, bronchitis, wheeze, and eczema in children was assessed at ages 6 and 14 months and 4 and 7 years through questionnaires given to the mothers. Atopy (specific IgE measurement) and asthma (questionnaire) were assessed at ages 4 and 7 years, respectively	Relative risks (RR) associated with BPA concentration:<br>- wheeze □ RR 1.20; 95% CI, 1.03-1.40; P = 0.02);<br>- chest infections □ RR 1.15; 95% CI, 1.00-1.32; P = 0.05);<br>- bronchitis □ RR 1.18; 95% CI, 1.01-1.37; P = 0.04.<br>The abovementioned RRs increased at any age for each doubling in concentration of maternal urinary BPA.<br><br>Σ4DEHP metabolites were associated with the same outcomes:<br>- wheeze □ RR 1.25; 95% CI, 1.04-1.50, P = 0.02;<br>- chest infections □ RR, 1.15; 95% CI, 0.97-1.35; P = 0.11;<br>- bronchitis □ RR, 1.20; 95% CI, 1.01-1.43; P = 0.04).<br><br>MBzP was associated with higher risk of wheeze □ RR, 1.15; 95% CI, 1.00-1.33; P = 0.05).<br><br>The risk of asthma at age 7 years was also increased with increasing prenatal BPA, Σ4DEHP, and MBzP exposure:<br>- Σ4DEHP □ RR, 1.38; 95% CI, 1.05-1.82; *P* = 0.02<br>- MBzP □ RR, 1.26; 95% CI, 1.01-1.82; *P* = 0.02<br>- BPA □ RR, 1.21; 95% CI, 0.94-1.57), but this association was not	Prenatal exposure to BPA and high-molecular-weight phthalates might increase the risk of asthma symptoms and respiratory tract infections throughout childhood.

						statistically significant (*P* = 0.14<br><br>There were no other exposure-outcome associations.	

Urbanization factors associated with childhood asthma and prematurity: a population-based analysis aged from 0 to 5 years in Taiwan by using Cox regression within a hospital cluster model<br><br>Lin SC, 2015<br><br>[5]	Retrospective cohort study	To explore association between urbanization, asthma and prematurity among children by using a population-based analysis.	Data derived from the Longitudinal Health Insurance Database 2005 (LHID2005) in Taiwan	N = 532<br><br>< 5 years of age<br><br>(n =60.505 age-matched control patients)	Evaluation of prematurely born infants and children using Cox proportional hazard regression analysis within a hospital cluster model, during a 5-year follow-up	Variable □ adjusted HR (CI 95%, p-value) for presence of asthma:<br><br>1) Prematurity □ aHR 1.45 (1.27-1.67; p<0.001)<br><br>2) Age □ aHR 0.85 (0.64-0.86, p<0.001)<br><br>3) Gender (M) □ aHR 1.34 (1.30-1.39, p<0.001)<br><br>4) Urbanization level:<br>Rural □ aHR 1<br>Suburban □ aHR 1.25 (1.08-1.45, p<0.001)<br>Urban □ aHR 1.07 (0.93-1.23, p=0.326)	Sex, age, urbanization level, and geographic region are significantly associated with prematurity and asthma. Based on cumulative asthma-free survival curve generated using the Kaplan–Meier method, infants born prematurely should be closely monitored to see if they would develop asthma until the age of 6 years.

Respiratory hospital admissions in young children living near metal smelters, pulp mills and oil refineries in two Canadian provinces<br><br>Brand A, 2016<br><br>[6]	Case-crossover analysis between epidemiological studies	To assess the effects of community exposure to industrial air emissions of PM2.5, SO2, and NO2 on respiratory hospital admissions for wheezing diseases in young children.	Children living in communities with metal smelters, oil refineries, and pulp mills in the Canadian provinces of Quebec (QC) and British Columbia (BC).	2868 cases<br><br>2-4 years of age	Assessing and pooling associations between the following estimates of exposure and hospital admissions for asthma and bronchiolitis in children:<br>1) Crude emission exposures at the residential postal codes of children, calculated by multiplying estimated daily emissions of PM2.5, SO2, or NO2 from all nearby (<7.5 km) pulp mills, oil refineries, metal smelters emitting yearly ≥50 t and their total emissions, by the percent of the day each postal code was downwind;<br><br>2) Daily levels of these pollutants at central ambient monitoring stations nearby the industries and the children's residences.	Odds ratios (ORs) for crude refinery and smelter emissions were positive in QC but more variable in BC:<br>1) For PM2.5 in QC, ORs were 1.13 per 0.15 t/day (95% CI: 1.00–1.27) and 1.03 (95% CI: 0.99–1.07) for refinery and smelter emissions, respectively.<br><br>Pooled results of QC and BC for crude total SO2 emissions from all sources indicated a 1% increase (0–3%) in odds of hospital admissions per 1.50 t/day increase in exposure.<br><br>Associations with measured pollutant levels were only seen in BC, with SO2 and NO2.	Hospital admissions for wheezing diseases in young children were associated with community exposure to industrial air pollutant emissions. Future work is needed to better assess the risk of exposure to complex mixture of air pollutants from multiple industrial sources.

Association between traffic-related air pollution and asthma in preschool children in a national Japanese nested case-control study<br><br>Hasunuma H, 2016<br><br>[7]	Prospective longitudinal study and a subsequent two-stage nested case-control study	To evaluate the association of exposure to traffic-related air pollution with the incidence/persistence of asthma during the first 3 years of life using a population-based study.	Preschool japanes children	Baseline survey conducted in 1½-year-old children (n=63,266)<br><br>Follow-up survey at 3 years of age (n=43,343)<br><br>New-onset asthma cases (n=853, controls = 3409) and persistence of asthma (n=214).	In the baseline survey at age 1½ years, the outcome was the prevalence of asthma. In the follow-up survey at age 3 years, the incidence of asthma was identified as well as the persistence of asthmatic symptoms during the follow-up period.<br><br>In the nested case–control study, participants with new-onset asthma between the ages of 1½ and 3 years were regarded as cases, and participants randomly selected from those who did not develop asthma during this period were regarded as controls. An interview, blood examination and mite allergen test were conducted	No statistically significant association between the incidence of asthma between age 1½ and 3 years and personal exposure levels to NOx (nitrogen oxides) nor EC (elemental carbon) □ the OR of level 5 NOx compared with level 1 was 1.20 (95% CI 0.79 to 1.84), and that of level 5 EC compared with level 1 was 1.04 (95% CI 0.67 to 1.61)<br><br>The persistence of asthmatic symptoms (between 1½ and 3 years) was significantly associated with outdoor concentrations of NOx □ ORs 6.02 (95% CI 1.51 to 23.92) for the comparison between the upper 5th and lower 25th centiles of NOx	While no statistically significant association was observed for the incidence of asthma, the persistence of asthmatic symptoms in preschool children was significantly associated with traffic-related air pollution □ importance as a risk factor in childhood airway disease

The independent role of prenatal and postnatal exposure to active and passive smoking on the development of early wheeze in children<br><br>Vardavas CI, 2016<br><br>[8]	Meta-analysis and pooled analysis of 15 prospective mother-child cohorts	To examine the independent and combined role of different sources of exposure during different timeframes (prenatal maternal passive smoking, prenatal maternal active smoking and children's postnatal passive smoking exposure) on the development of wheeze symptoms up to the age of 2 years	Mother-child pairs form 15 European birth cohorts.<br><br>The 15 cohorts providing primary data included in this analysis participated in the European project Environmental Health Risks in European Birth Cohorts (ENRIECO)	27.993 mother-child pairs.<br><br>Age of children: from birth to 2 years of age	Using a multilevel mixed-effects logistic regression to evaluate the effect of exposure to tobacco smoke on the development of child wheeze during the first 2 years of life, calculating odds ratios and 95% confidence intervals.	A. Children with maternal exposure to passive smoking during pregnancy and no other smoking exposure were more likely to develop wheeze up to the age of 2years [illegible] OR 1.11, 95% CI 1.03–1.20) compared with unexposed children.<br><br>B. Risk of wheeze was further increased by children's postnatal passive smoke exposure in addition to their mothers' passive exposure during pregnancy - [illegible] OR 1.29, 95% CI 1.19–1.40.<br>B1. The risk was highest in children with both sources of passive exposure and mothers who smoked actively during pregnancy [illegible]OR 1.73, 95% CI 1.59–1.88).<br><br>C. Risk of wheeze associated with tobacco smoke exposure was higher in children with an allergic (OR 1.49, 95% CI 1.35–1.66)<br>*versus* nonallergic family history (OR 1.15, 95% CI 1.00–1.32; p-value for interaction 0.043)<br>C1. OR for exposure to maternal active smoking during pregnancy was increased in children with a parental history of allergy (OR 2.25, 95% CI 1.99–2.54) compared to those without (OR 1.28, 95% CI	Maternal passive smoking exposure during pregnancy is an independent risk factor for wheeze in children up to the age of 2 years.

						1.09–1.50; p-value for interaction 0.011).	

Differences between preschoolers with asthma and allergies in urban and rural environments<br><br>Marfortt DA, 2018<br><br>[9]	Cross-sectional-study	The aim of the present study was to evaluate if recurrent wheezing preschoolers from rural or urban areas differ in asthma, allergic diseases, and atopy.	All the children enrolled were referred to a pediatric pulmonology clinic in Rafaela.<br><br>It is a sample of 5 years of age children with recurrent wheezing in outpatient clinics located in downtown Rafaela and in five surrounding rural areas.	143 preschoolers divided in urban and rural, according to the geographic area they lived in.	A detailed questionnaire was completed and collected at enrollment.<br>This questionnaire included information about demographic characteristics, parental history of asthma and allergic diseases, use of antibiotics by the mother during pregnancy and breastfeeding, exposure to farm animals, pets and tobacco, cooking and heating appliances, bedroom ventilation index, fumigation near the house, wheezing episodes, use of antibiotics during the first year of life, and assistance to day care centers.<br><br>Anthropometric measurements and stigmata of allergic rhinitis and atopic dermatitis were also recorded during the enrollment process.<br><br>A peripheral blood sample was obtained the same day the admission questionnaire was	Preschoolers from rural settings had significantly higher prevalence of vaginal delivery, longer breastfeeding, earlier onset of wheezing, more parental smoking, siblings, shared a bedroom, and more exposure to chemicals used in plant fumigation or farm animals, and unpasteurized milk consumption, in comparison to preschoolers living in urban setting.<br><br>In contrast, preschoolers from urban areas had significantly higher prevalence of parental history of allergy, positive skin prick test, and positive API.<br><br>After multivariate analysis adjusting for covariates, maternal smoking [odds ratio (OR) = 3.44] and positive SPT (OR = 5.57) significantly increase the risk of asthma diagnosis (positive API); in contrast, living in rural setting (OR = 0.04), and having more siblings (OR = 0.51) decrease their risk.	Recurrent wheezing preschoolers from rural areas had a significant inverse odds of being diagnosed with asthma (type-2 inflammation) when compared to those from urban areas.

					completed in order to assess eosinophil counts and IgE measurements, and a skin prick test was performed.		

Household dampness related exposures in relation the childhood asthma and rhinitis in China: multicenter observational study.<br><br>Cai J, 2019<br><br>[10]	Multicenter observational study	The study is a part of the China, Children, Homes, Health (CCHH) study.<br><br>Based on the parent-administered data, the main purpose of the study is to explore the associations of household environment with lifetime-ever (from the child's birth to the survey) and current (in the last year before the survey) asthma and rhinitis in preschool children. In each city, we used a standard questionnaire that was validated by a pilot study in Chongqing, China	Preschool children from 7 major cities in China	N = 40.010<br><br>From birth to 6 years of age	The associations of six dampness-related indicators (visible mold spots, visible damp stains, damp clothing and/or bedding, water damage, condensation on windowpane, moldy odor) in the current residence and three dampness-related indicators (visible mold spots, condensation on windowpane, moldy odor) in the early residence with childhood asthma and rhinitis were assessed in a multi-level logistic regression analyses	Visible mold spots and visible damp stains in the current residence were significantly associated with the increased odds of doctor-diagnosed asthma and allergic rhinitis during lifetime-ever aORs range: 1.18-1.35 (95% CI).<br><br>All dampness-related indicators were significantly associated with increased odds of wheeze and rhinitis during lifetime-ever and in the past 12 months AORs range: 1.16-2.64 (95% CI).<br><br>The cumulative numbers of damp indicators had positively dose-response relationships with the increased odds of the studied diseases.<br><br>The associations for wheeze and rhinitis were similar between northern children and southern children	Early and lifetime exposures to household dampness indicators are risk factors for childhood asthma and rhinitis.

Early-life exposure to household chemicals and wheezing in children<br><br>Mikeš O, 2019<br><br>[11]	Longitudinal cohort study	To study the potential effects of overall exposure to home chemicals in the early life on the phenotypes of wheezing, from birth until 5 years of age.	Mother-infant pairs from the Czech part of the European Longitudinal Study of Pregnancy and Childhood (ELSPAC-CZ)	3441 mother-infant pairs.<br><br>Age of children: from 0 to 5 years of age.	The exposure was estimated by the composite household chemical score (CHCE score) from 18 chemical-based products.<br><br>Social, medical and environmental factors were taken into account as covariates in multivariable multinomial logistic regression using phenotypes of wheezing as a study outcome.	A. Statistically significant odds ratios (OR) for increasing exposures per 1 SD of CHCE score were obtained for:<br>- intermediate onset transient wheezing (OR 1.27, 95% CI 1.10-1.47);<br>- intermediated onset persistent wheezing (OR 1.23, 95% CI 1.03-1.46);<br>- early onset persistent wheezing (OR 1.36, 95% CI 1.04-1.77) in comparison to never wheezing children.<br><br>B. OR (95% CI) of doctor-diagnosed asthma at 7 years of child age by wheezing phenotypes.<br>1) Early onset transient: 0.26 (0.04-1.93, p=0.189)<br>2) intermediate onset transient: 1.63 (0.83-3.21, p=0.154)<br>**3) Early onset persistent: 5.26 (2.63-10.53, p<0.001)**<br>**4) Intermediate onset persistent: 7.52 (4.63-12.20, p<0.001)**<br>**5) Late onset: 7.58 (4.57-12.65, p<0.001)**	There is a negative role of the increased household chemicals usage on the respiratory outcomes in children up to five years of age.<br><br>Persistent phenotypes were significantly associated with school age asthma

Onset and remission of childhood wheeze and rhinitis across China - Associations with early life indoor and outdoor air pollution<br><br>Norbäck D, 2019<br><br>[12]	Longitudinal, Observational study	To study associations between indoor and outdoor environment and prevalence, onset and remission of wheeze and rhinitis	Children not moving since birth across China	N = 17.679<br><br>From 3 to 6 years of age	Data on wheeze, rhinitis and the home environment were assessed by a parental questionnaire. Prevalence in the first two years of life (baseline) and the last year (follow-up) was used to calculate onset and remission.<br><br>Outdoor $PM_{2.5}$, PM_{10}, and NO_2 at the day care centre were modelled from monitoring station data. Associations were calculated by multilevel logistic regression.	A1. Prenatal exposure to NO_2 was associated with lower remission of wheeze (OR = 0.63 per IQR; 95% CI 0.49–0.81).<br><br>A2. Postnatal $PM_{2.5}$ was associated with prevalence of wheeze (OR 1.15 per IQR; 95% CI 1.00–1.31).<br><br>A3. Postnatal PM_{10} was associated with lower remission of wheeze (OR = 0.50 per IQR; 95% CI 0.30–0.84).<br><br>A4. Postnatal NO_2 was associated with prevalence of wheeze (OR 1.12 per IQR; 95% CI 1.03–1.22) as well as lower remission of wheeze (OR = 0.70 per IQR; 95% CI 0.57–0.86).<br><br>Since the correlation between prenatal and post-natal NO_2 (on day care center level) was strong (*r* = 0.87) it was not possible to include prenatal and postnatal exposure in the same model.<br><br>B1. Home renovation was associated with prevalence of wheeze (OR 1.14, 95% CI 1.01-1.28) as well as onset of wheezing (OR 1.16, 95% CI 1.04-1.30) and lower remission (OR 0.89 95% CI 95% 0.70-1.12)<br><br>B2. Visible mould/damp	Outdoor $PM_{2.5}$, PM_{10} and NO_2 can increase childhood wheeze and rhinitis.<br><br>Dampness and mould can increase onset and decrease remission.

						stains at home:<br>- prevalence of wheezing: OR 1.22, 95% CI 1.03-1.44;<br>- onset of wheezing: OR 1.31, 95% CI 1.12-1.54<br>- remission: OR 0.71, 95% CI 0.51-1.00	

Air pollution and family related determinants of asthma onset and persistent wheezing in children: nationwide case-control study<br><br>Holst GJ, 2020<br><br>[13]	Case-control study	To identify risk factors (air pollution and family related) for the onset of asthma and persistent wheezing in children up to 15 years of age.	All Danish children born from 1997 to 2014 and followed for asthma onset and persistent wheezing	3.192.785 children were included in the study.<br><br>Of these, 122.842 children were identified as having asthma and persistent wheezing.<br><br>Most cases (83%, n=101.348) occurred among children younger than 3 years, with a mean age of 1.9 years (SD 2.2) for developing asthma and persistent wheezing.	An average of hourly air pollution concentrations during the past 3, 6, and 12 months before the index date for which the case and matched controls were identified has been used.<br><br>Air pollution concentrations were linked to the children's residential addresses with a detailed spatial resolution of 1 km×1 km grid cells, taking into account any changes in residence.	A. Exposure to particulate matter ≤2.5 µm ($PM_{2.5}$) and ≤10 µm (PM_{10}) and nitrate was associated with an increased risk of asthma and persistent wheezing, with hazard ratios per 5 µg/m^3 increase in pollutant concentrations 1.05 (1.03 to 1.07) for $PM_{2.5}$, 1.04 (1.02 to 1.06) for PM_{10}, and 1.04 (1.03 to 1.04) for nitrogen dioxide.<br><br>A1. Only the positive association of $PM_{2.5}$ with asthma and persistent wheezing remained robust across the different models and in sensitivity analyses.<br><br>A higher incidence of asthma was found in children of parents with asthma (aHR 2.29 (95% confidence interval 2.22 to 2.35) and mothers who smoked during pregnancy (1.20, 1.18 to 1.22), whereas a lower incidence was found in children of parents with high educational attainment (0.72, 0.69 to 0.75) and high incomes (0.85, 0.81 to 0.89).	Children exposed to higher levels of $PM_{2.5}$ are more likely to develop asthma and persistent wheezing than children who are not exposed.<br><br>Other risk factors associated with these outcomes were parental asthma, parental education, and maternal smoking during pregnancy.

The course of asthma: A population-based 10-year study examining asthma remission in children diagnosed with asthma in preschool.<br><br>Oluwole O, 2020<br><br>[14]	Retrospective birth cohort study	The aim is to follow-up preschool children with diagnosed asthma (with a focus on age of asthma diagnosis defined as early-onset (≤ 3 years) and late-onset (4– 6 years)) and investigate the occurrence of persistence and remission of asthma over a 10-year period following diagnosis.	Children with a diagnosis of asthma in the first 6 years of life and who had at least 10 years of follow-up after diagnosis, from Saskatchewan Ministry of Health databases.<br><br>The study includes children born between January 1, 1995 and December 31, 2014 who had either a physician visit or hospitalization for asthma during this time	22,563 children who had been diagnosed with asthma within the first 6 years of life and who had at least 10 years of follow-up after their diagnosis.	Children were categorized into one of two asthma groups: ≤ 3 years (early-onset) and > 3 years up to 6 years (late-onset).<br><br>Sex, location of residence, cohort, and allergic status were considered as covariates in this study.<br><br>At the end of the 10 year follow-up period, the following different patterns of asthma outcomes were identified : "persistent asthma", complete remission, relapse, intermittent ashtma.	Of the study participants, 87.2% had early-onset (≤ 3 years) and 12.8% had late-onset (4–6 years) asthma.<br><br>Over the 10-years of follow-up, rate of asthma remission was 37 per 100 person-years.<br><br>Factors positively associated with remission:<br>- Early-onset asthma (HR = 1.10, 95%CI: 1.01–1.20),<br>- being female (HR = 1.12, 95%CI: 1.07–1.18),<br>- living in a rural location (HR = 1.20, 95%CI: 1.14–1.27)<br>- Living in a medium urban location (HR = 1.16, 95%CI: 1.08–1.26).<br><br>History of atopy decreased likelihood of remission (HR = 0.73, 95%CI: 0.54–0.97).	Most children with asthma experienced remission, especially those with onset of symptoms within the first 3 years of life.

Association of use of cleaning products with respiratory health in a Canadian birth cohort<br><br>Parks J, 2020<br><br>[15]	Prospective birth cohort study	To examine associations between use of household cleaning products in early life and childhood respiratory and allergic disease	Data from the Canadian Healthy Infant Longitudinal Development (CHILD) Cohort Study	N = 2022<br><br>From 3 months to 3 years of age	Assessment of parental questionnaires that indicated the frequency of use of 26 household cleaning products in homes from 3–4 months of age to create a cumulative Frequency of Use Score (FUS). Using multivariable logistic regression models to assess whether frequent compared with less frequent use was associated with recurrent wheeze, atopy or asthma diagnosis, as defined by the questionnaire and clinical assessments at age 3 years	A1. Children in homes with a higher frequency of use of cleaning products in infancy, as determined by an interquartile range increase, had higher odds of:<br>- recurrent wheeze □ aOR = 1.35, (95% CI 1.11–1.64);<br>- recurrent wheeze with atopy □ aOR 1.49, (95% CI 1.02–2.16);<br>- asthma diagnosis □ aOR 1.37, (95% CI 1.09–1.70);<br><br>A2. Children in homes with a higher frequency of use of cleaning products in infancy, as determined by an interquartile range increase, had no increase in the odds of atopy at age 3 years □ aOR 1.14 (95% CI 0.96–1.35).<br><br>B. Compared with the lowest tertile of FUS exposure, infants in the highest tertile had higher odds of acquiring asthma, although this trend was nonsignificant:<br>- associations with recurrent wheeze □ aOR 1.26, 95% CI 0.85–1.88);<br>- recurrent wheeze with atopy □ aOR 1.76, 95% CI 0.81–4.06);<br>- asthma □ aOR 1.57, 95% CI 0.98–2.53);<br>- The trend wasn't confirmed for atopy alone □aOR 1.17 (95% CI 0.85–1.63)	Frequent use of household cleaning products in early life was associated with an increased risk for childhood wheeze and asthma but not atopy at age 3 years.

						C. Stratification of the results showed that females had higher ORs than males for all outcomes, although the *p* values for this sex difference did not reach statistical significance	

Proximity to Major Roads and Risks of Childhood Recurrent Wheeze and Asthma in a Severe Bronchiolitis Cohort<br><br>Freid D, 2021<br><br>[16]	Multicenter Prospective birth cohort study	To investigate the association of cumulative early-life residential proximity to the nearest major or primary road with risk of recurrent wheeze at birth to age 3 years and incidence of asthma by age 5 years among children with a history of severe bronchiolitis at infancy	Infants hospitalized for bronchiolitis and recruited from 14 U.S. states	N = 921<br><br>From birth to 5 years of age	Primary exposure was residential proximity to the nearest major road at birth through age 3 years. Residential distance from nearest major road was divided into four categories: <100, 100–200, 201–300, and >300 mt.<br><br>Outcomes were parent-reported recurrent wheeze by age 3 years and asthma by age 5 years. Associations between residential proximity to major roads and respiratory outcomes were investigated using multivariable Cox proportional hazards modeling and logistic regression, adjusted for confounders.	241 (26%) participants resided within 300 m of a major road, 296 (32%) developed recurrent wheeze by age 3, and 235 out of 858 participants (27%) developed asthma by 5 years.<br><br>Participants who resided close to a major road had the highest risk of recurrent wheeze (aHR for <100 m, 1.59, 95% CI: 1.08–2.33) and asthma (aOR for 201–300 m, 1.62, 95%CI: 1.16–2.25), compared to those residing >300 m from a major road.	Proximity to major roads is associated with increased risks of recurrent wheeze and asthma in young children.

Early-life exposure to submicron particulate air pollution in relation to asthma development in Chinese preschool children<br><br>Zhang Y, 2021<br><br>[17]	Cross-sectional survey	To investigate associations of childhood asthma and wheezing with in utero and first-year exposures to size-specific particles.	Preschool children in central China	N= 5788<br><br>From 3 to 5 years	In utero and first-year exposures to ambient PM_1, PM with aerodynamic diameter less than or equal to 2.5 µm, and PM with aerodynamic diameter less than or equal to 10 µm at 1 × 1-km resolution were assessed using machine learning-based spatiotemporal models. A time-to-event analysis was performed to examine associations between residential PM exposures and childhood onset of asthma and wheezing.	Each 10-µg/m^3 increase in in utero and first-year PM_1 exposure was accordingly associated with an asthma's HR in childhood of 1.618 (95% CI, 1.159-2.258; P = .005) and 1.543 (0.822-2.896; P = .177).<br><br>Subgroup analyses suggest that short breast-feeding duration may aggravate PM-associated risk of childhood asthma.<br><br>Each 10-µg/m^3 increase in in utero exposure to PM_1 was associated with a hazard ratio of 2.260 (1.393-3.666) among children with 0 to 5 months' breast-feeding and 1.156 (0.721-1.853) among those longer breast-fed.	Early-life size-specific PM exposures, particularly during pregnancy, were significantly associated with increased risk of asthma, whereas no evident PM-wheezing associations were observed.<br><br>Increased risk of childhood asthma in relation to early-life PM exposures, highlighting stronger associations with ambient PM_1 than with PM with aerodynamic diameter less than or equal to 2.5 µm and PM with aerodynamic diameter less than or equal to 10 µm.

References

1. Nordling E, Berglind N, Melén E, Emenius G, Hallberg J, Nyberg F, Pershagen G, Svartengren M, Wickman M, Bellander T. Traffic-related air pollution and childhood respiratory symptoms, function and allergies. Epidemiology. 2008 May;19(3):401-8. doi: 10.1097/EDE.0b013e31816a1ce3. PMID: 18379426.
2. Esposito S, Galeone C, Lelii M, Longhi B, Ascolese B, Senatore L, Prada E, Montinaro V, Malerba S, Patria MF, Principi N. Impact of air pollution on respiratory diseases in children with recurrent wheezing or asthma. BMC Pulm Med. 2014 Aug 7;14:130. doi: 10.1186/1471-2466-14-130. PMID: 25098250; PMCID: PMC4126992.
3. Brunst KJ, Ryan PH, Brokamp C, Bernstein D, Reponen T, Lockey J, Khurana Hershey GK, Levin L, Grinshpun SA, LeMasters G. Timing and Duration of Traffic-related Air Pollution Exposure and the Risk for Childhood Wheeze and Asthma. Am J Respir Crit Care Med. 2015 Aug 15;192(4):421-7. doi: 10.1164/rccm.201407-1314OC. PMID: 26106807; PMCID: PMC4595664.
4. Gascon M, Casas M, Morales E, Valvi D, Ballesteros-Gómez A, Luque N, Rubio S, Monfort N, Ventura R, Martínez D, Sunyer J, Vrijheid M. Prenatal exposure to bisphenol A and phthalates and childhood respiratory tract infections and allergy. J Allergy Clin Immunol. 2015 Feb;135(2):370-8. doi: 10.1016/j.jaci.2014.09.030. Epub 2014 Oct 30. PMID: 25445825.
5. Lin SC, Lin HW. Urbanization factors associated with childhood asthma and prematurity: a population-based analysis aged from 0 to 5 years in Taiwan by using Cox regression within a hospital cluster model. J Asthma. 2015 Apr;52(3):273-8. doi: 10.3109/02770903.2014.958854. Epub 2014 Sep 16. PMID: 25171433.
6. Brand A, McLean KE, Henderson SB, Fournier M, Liu L, Kosatsky T, Smargiassi A. Respiratory hospital admissions in young children living near metal smelters, pulp mills and oil refineries in two Canadian provinces. Environ Int. 2016 Sep;94:24-32. doi: 10.1016/j.envint.2016.05.002. Epub 2016 May 18. PMID: 27203781.
7. Hasunuma H, Sato T, Iwata T, Kohno Y, Nitta H, Odajima H, Ohara T, Omori T, Ono M, Yamazaki S, Shima M. Association between traffic-related air pollution and asthma in preschool children in a national Japanese nested case-control study. BMJ Open. 2016 Feb 25;6(2):e010410. doi: 10.1136/bmjopen-2015-010410. PMID: 26916696; PMCID: PMC4769392.
8. Vardavas CI, Hohmann C, Patelarou E, Martinez D, Henderson AJ, Granell R, Sunyer J, Torrent M, Fantini MP, Gori D, Annesi-Maesano I, Slama R, Duijts L, de Jongste JC, Aurrekoetxea JJ, Basterrechea M, Morales E, Ballester F, Murcia M, Thijs C, Mommers M, Kuehni CE, Gaillard EA, Tischer C, Heinrich J, Pizzi C, Zugna D, Gehring U, Wijga A, Chatzi L, Vassilaki M, Bergström A, Eller E, Lau S, Keil T, Nieuwenhuijsen M, Kogevinas M. The independent role of prenatal and postnatal exposure to active and passive smoking on the development of early wheeze in children. Eur Respir J. 2016 Jul;48(1):115-24. doi: 10.1183/13993003.01016-2015. Epub 2016 Mar 10. PMID: 26965294.
9. Marfortt DA, Josviack D, Lozano A, Cuestas E, Agüero L, Castro-Rodriguez JA. Differences between preschoolers with asthma and allergies in urban and rural environments. J Asthma. 2018 May;55(5):470-476. doi: 10.1080/02770903.2017.1339800. Epub 2017 Jul 21. PMID: 28605217.
10. Cai J, Li B, Yu W, Wang H, Du C, Zhang Y, Huang C, Zhao Z, Deng Q, Yang X, Zhang X, Qian H, Sun Y, Liu W, Wang J, Yang Q, Zeng F, Norbäck D, Sundell J. Household dampness-related exposures in relation to childhood asthma and rhinitis in China: A multicentre observational study. Environ Int. 2019 May;126:735-746. doi: 10.1016/j.envint.2019.03.013. Epub 2019 Mar 14. PMID: 30878869.
11. Mikeš O, Vrbová M, Klánová J, Čupr P, Švancara J, Pikhart H. Early-life exposure to household chemicals and wheezing in children. Sci Total Environ. 2019 May 1;663:418-425. doi: 10.1016/j.scitotenv.2019.01.254. Epub 2019 Jan 25. PMID: 30716632.
12. Norbäck D, Lu C, Zhang Y, Li B, Zhao Z, Huang C, Zhang X, Qian H, Sun Y, Sundell J, Juan W, Liu W, Deng Q. Onset and remission of childhood wheeze and rhinitis across China - Associations with early life indoor and outdoor air pollution. Environ Int. 2019 Feb;123:61-69. doi: 10.1016/j.envint.2018.11.033. Epub 2018 Nov 26. PMID: 30496983.

13. Holst GJ, Pedersen CB, Thygesen M, Brandt J, Geels C, Bønløkke JH, Sigsgaard T. Air pollution and family related determinants of asthma onset and persistent wheezing in children: nationwide case-control study. BMJ. 2020 Aug 19;370:m2791. doi: 10.1136/bmj.m2791. PMID: 32816747; PMCID: PMC7437497. Holst GJ, Pedersen CB, Thygesen M, Brandt J, Geels C, Bønløkke JH, Sigsgaard T. Air pollution and family related determinants of asthma onset and persistent wheezing in children: nationwide case-control study. BMJ. 2020 Aug 19;370:m2791. doi: 10.1136/bmj.m2791. PMID: 32816747; PMCID: PMC7437497.
14. Oluwole O, Rennie DC, Goodridge D, Blackburn D, Litzenberger T, Penz E, Lawson JA. The course of asthma: A population-based 10-year study examining asthma remission in children diagnosed with asthma in preschool. Pediatr Pulmonol. 2020 Aug;55(8):1924-1935. doi: 10.1002/ppul.24881. Epub 2020 Jun 9. PMID: 32478962.
15. Parks J, McCandless L, Dharma C, Brook J, Turvey SE, Mandhane P, Becker AB, Kozyrskyj AL, Azad MB, Moraes TJ, Lefebvre DL, Sears MR, Subbarao P, Scott J, Takaro TK. Association of use of cleaning products with respiratory health in a Canadian birth cohort. CMAJ. 2020 Feb 18;192(7):E154-E161. doi: 10.1503/cmaj.190819. PMID: 32071106; PMCID: PMC7030878.
16. Freid RD, Qi YS, Espinola JA, Cash RE, Aryan Z, Sullivan AF, Camargo CA Jr. Proximity to Major Roads and Risks of Childhood Recurrent Wheeze and Asthma in a Severe Bronchiolitis Cohort. Int J Environ Res Public Health. 2021 Apr 15;18(8):4197. doi: 10.3390/ijerph18084197. PMID: 33921001; PMCID: PMC8071463.
17. Zhang Y, Wei J, Shi Y, Quan C, Ho HC, Song Y, Zhang L. Early-life exposure to submicron particulate air pollution in relation to asthma development in Chinese preschool children. J Allergy Clin Immunol. 2021 Sep;148(3):771-782.e12. doi: 10.1016/j.jaci.2021.02.030. Epub 2021 Mar 5. PMID: 33684436.

Question 4. Does genetics influence the onset and the evolution of preschool wheezing?

Search strategies:

("child, preschool"[MeSH Terms] OR "toddler*"[All Fields]) AND ("respiratory sounds"[MeSH Terms] OR "wheez*"[All Fields] OR "Asthma"[MeSH Terms:noexp]) AND ("family history"[All Fields] OR "familiar history"[All Fields] OR "familiar medical history"[All Fields]) AND "english"[Language] AND 2008/01/01:2021/12/31[Date - Publication]

("child, preschool"[MeSH Terms] OR "toddler*"[All Fields]) AND ("respiratory sounds"[MeSH Terms] OR "wheez*"[All Fields] OR "Asthma"[MeSH Terms:noexp]) AND "Risk Factors"[MeSH Terms] AND ("Age of Onset"[MeSH Terms] OR "Onset"[All Fields]) AND "english"[Language] AND 2008/01/01:2021/12/31[Date - Publication]

("child, preschool"[MeSH Terms] OR "toddler*"[All Fields]) AND ("respiratory sounds"[MeSH Terms] OR "wheez*"[All Fields] OR "Asthma"[MeSH Terms:noexp]) AND "Risk Factors"[MeSH Terms] AND ("disease progression"[MeSH Terms] OR "disease evolution"[All Fields] OR "evolution"[All Fields]) AND "english"[Language] AND 2008/01/01:2021/12/31[Date - Publication]

**Title of the study, first author, year**	**Type of study**	**Objective**	**Population**	**N of patients, Age**	**Experiments/mechanisms assessed**	**Relative risk (RR) or odds ratio (OR) + confidence interval (CI 95%)**	**Results**

Risk factors associated with transient wheezing in young children.<br><br>Simon MR, 2008<br><br>[1]	Birth cohort study	A prospective analysis of a relatively large population-based<br><br>birth cohort to define the relationship of atopic risk factors and sensitization in association with transient wheezing in young children.	Childhood Allergy Study population	372 children<br>- 189 females<br>- 183 males	Data were collected by questionnaires at ages 1, 2, 3 and 4.<br>Information collected on:<br>- presence of transient wheezing,<br>- atopy risk factor,<br>- exposition to pets,<br>- early antibiotic administration,<br>- persistent breast-feeding,<br>- Daycare attendance.<br><br>Between ages 6 and 7 years the children were clinically evaluated for circulating IgE to Dermatophagoides farinae, dog, cat, short ragweed, and timothy grass antigens by skin prick tests.	A. 175 (47.0%) of the 372 subjects had never wheezed and 128 (34.4%) subjects had wheezed in the previous year at ages 1, 2, and/or 4 years, but not at age 6 years (transient wheezing).<br>A. Boys were more likely to transiently wheeze (RR 1.7; CI, 1.1–2.8; p 0.018; Table 2).<br>B. Early antibiotic use was associated also with an increased risk (RR 1.6; CI, 1.0 –2.6; p 0.048).<br>C. Breastfeeding for 4 or more months was not associated with transient wheezing compared with never having wheezed (RR 0.9; CI, 0.7–1.1; p 0.34). Breastfeeding was further evaluated by assessing transient wheezing in children who were exclusively breast-fed (RR 0.9; CI, 0.7–1.2; p 0.62) and those who received supplemental feedings as well as breast milk (RR 0.7; CI, 0.5–1.1; p 0.082), suggesting some protection from supplementation and breast-feeding.<br>D. There was no association of birth order, parental allergy, pet keeping, fever in the 1st year of life, or day care with transient wheezing.<br><br>Children with transient wheezing were not more likely to be classified as atopic by ELISA (OR 1.2; CI, 0.5–2.7; p 0.66; Table 3), and they	Transient wheezing in young children is not associated with allergy and appears to be more common in boys and among children who received treatment with<br><br>antibiotics in the first 6 months of life.

						were not more likely to have positive skin-prick test results (OR 0.8; CI, 0.4 –1.5; p 0.47; Table 3).	

Chromosome 17q21 gene variants are associated with asthma and exacerbations but not atopy in early childhood.<br><br>Bisgaard H, 2009.<br><br>[2]	Birth cohort study	To characterize the asthma and atopy phenotypes in early<br><br>childhood that associate with the 17q12-21 locus.	Copenhagen Prospective Study on Asthma in Childhood (COPSAC) birth cohort born to mothers with asthma	376 children	Clinical endpoints investigated from birth, clinical-reported every 6 months or recorded in daily diaries by parents:<br>- Recurrent wheeze,<br>- Asthma,<br>- Episodic viral wheeze,<br>- Acute severe exacerbations,<br>- Rhinitis,<br>- Eczema<br><br>The single nucleotide polymorphism (SNP), rs7216389, was genotyped, Nineteen additional SNPs in the region were genotyped in the children.	rs7216389 was significantly associated with the development of:<br>- wheeze (hazard ratio 1.64 [1.05–2.59], P value = 0.03),<br>- asthma (hazard ratio, 1.88 [1.15–3.07], P = 0.01),<br>- acute severe exacerbations (hazard ratio 2.66 [1.58–4.48], P value = 0.0002).<br><br>The effect on wheeze and asthma was observed for early onset but not late onset of disease.<br><br>The increased risk of exacerbations persisted from 1 to 6 years of age (incidence ratio 2.48 [1.42–4.32], P value = 0.001), and increased bronchial responsiveness was present in infancy and at 4 years of age, but not at 6 years.<br><br>In contrast, rs7216389 conferred no risk of eczema, rhinitis, or allergic sensitization.	Variation at the chromosome 17q12-q21 locus was associated with approximately twofold increased risk of recurrent wheeze, asthma, asthma exacerbations, and bronchial hyperresponsiveness<br><br>from early infancy to school age.

An interaction between filaggrin mutations and early food sensitization improves the prediction of childhood asthma.<br><br>Marenholz I, 2009<br><br>[3]	Birth cohort study	Evaluation of the utility of the FLG loss of function mutations for the prediction of asthma.	Prospective German Multicenter Allergy Study (MAS) birth cohort (chidren born in 1990)	871 children, followed at the ages of 1, 3, 6, 12, 18 and 24 months, and at a yearly intervals until age 13.	Clinical assessement with standardized interviews, questionnaires and physical examinations.<br>Phenotypes evaluated:<br>- eczema<br>- asthma<br>- lung function<br>- allergic sensitization<br><br>Three FLG mutations were genotyped (R501X, 2282del4 and R2247X).	In infants with eczema and sensitization to food allergens, the FLG mutations predicted childhood asthma with a positive predictive value of 100% (95% CI, 65,5% to 100%).<br><br>There is a strong synergic interaction between the FLG-null alleles and early food sensitization in disease transiting from eczema to asthma (relative excess risk due to interaction, 2.64; 95% CI, 1.70-3.98; P=.00040).	FLG mutations and food sensitization represent 2 distinct mechanism interacting in the pathogenesis of asthma before the onset of symptoms.

A sequence variant on 17q21 is associated with age at onset and severity of asthma.<br><br>Halapi E, 2010<br><br>[4]	Case–control study	Evaluation of the effect of rs7216389-T on asthma subphenotypes.<br><br>Correlation between the presence of rs7216389-T and expression levels of neighboring genes.	**Primary outcome population**: six European and one Asian study cohort (Iceland, Germany, The Netherlands, the United Kingdom, Australia and Korea, and a familial sample set from Germany).<br><br>**Secondary outcome population**: only Icelandic individuals	**Primary outcome population**:<br>- N=4917 cases<br>- N=34.589 controls<br><br>**Secondary outcome population** :<br>- N=743	For all sample sets, a diagnosis of asthma and the assessement of its severity was given on the basis of international and national asthma guidelines.<br><br>**Primary outcome method**: All genotyping procedures of SNPs in the 17q21 region were carried out using Hap300 bead-array (Illumina Inc., San Diego, CA, USA), TaqMan (Applied Biosystems Inc., Cheshire, UK) or Centaurus platforms (NanoGen Inc., San Diego, CA, USA).<br><br>**Secondary outcome method**: Expression levels for genes in the 17q21 region and correlation with the genotype status of 15 SNPs in the region were assessed as part of a separate study.	**Primary outcome**: The association of rs7216389-T with asthma severity was confined to cases with early onset of asthma, particularly in early childhood (age: 0-5 years OR=1.51, P=6.89.10(-9)) and adolescence (age: 14-17 years OR=1.71, P=5.47.10(-9)). A weaker association was observed for onset between 6 and 13 years of age (OR=1.17, P=0.035), but none for adult-onset asthma (OR=1.07, P=0.12).<br><br>**Secondary outcome:** The strongest association of rs7216389 was seen with the expression of *GSDMB* and *ORMDL3* genes (P=2.3·10−38 and P=8.8·10−58, respectively), which expression is correlated in white blood cells (observed correlation r 0.71;).<br><br>By the way, the variants with the strongest association with asthma were different from those that were most strongly associated with the expression of *ORMDL3* or *GSDMB*.	**Primary outcome**: The 17q21 locus associated with disease severity among early-onset but not adult-onset cases.<br><br>**Secondary outcome**:<br><br>the contribution of rs7216389-T to the development of asthma is unlikely to operate only through an impact on the expression of ORMDL3 or GSDMB genes.

Polymorphism of the rs1800896 IL10 promoter gene protects children from post-bronchiolitis asthma.<br><br>Koponen P, 2013<br><br>[5]	Longitudinal study	The aim of the present study was to evaluate the associations between preschool-age asthma and polymorphisms in IL10 rs1800896, IFNG rs2430561, or IL18 rs1872387 after hospitalization for bronchiolitis in early infancy.	Preschool-aged children<br><br>hospitalized for bronchiolitis at age 0–6 months., enrolled from 2001 to 2004.	135 children (mean age: 6.4 years)	Parents were interviewed using a structured questionnaire that reviewed asthma and allergic symptoms from early infancy to present.<br><br>Genotyping of the IL10 rs1800896 and IL18 rs187238 gene polymorphism was performed using the ABI PRISM 7000 Sequence Detection System<br><br>(Applied Biosystems, Carlsbad, CA).	Homozygous for rs1800896 allele G were rarely asthmatics; only 1/32 (3.1%) had doctor diagnosed asthma at 5–7 years of age (P=0.04).<br>On the other hand, the low-producing genotype A/A carried a 1.27-fold (95% CI 1.01–1.60) asthma risk compared with genotypes A/G and G/G.<br><br>IFNG rs2430561 or IL18 rs1872387 polymorphims had no significant associations with asthma at any age, or with allergic rhinitis or atopic eczema.	IL10 rs1800896 SNP was significantly<br><br>associated with preschool asthma after severe lower respiratory tract infection in early infancy.

Integrative genomic analysis identifies a role for intercellular adhesion molecule 1 in childhood asthma.<br><br>Klaassen EM, 2014<br><br>[6]	Case-control study	To employ an integrative genomic approach investigating inflammation markers on DNA, mRNA, and protein level at preschool age in relationship to asthma development.	Children from the Asthma DEtection and Monitoring (ADEM) study	252 preschool children:<br>- 202 recurrent wheezers,<br>- 50 controls,<br><br>Enrolled at 2–4 years of age, followed until age 6.	Genetic variants, mRNA expression in peripheral blood mononuclear cells, and protein levels in exhaled breath condensate for intercellular adhesion molecule 1 (ICAM1), interleukin (IL)4, IL8, IL10, IL13, and tumor necrosis factor α were analyzed at preschool age.<br><br>At six years of age, a classification (healthy, transient wheeze, or asthma) was based on symptoms, lung function, and medication use.	The ICAM1 rs5498 A allele was positively associated with asthma development ($p = 0.02$) and ICAM1 gene expression ($p = 0.01$). ICAM1 gene expression was positively associated with exhaled levels of soluble ICAM1 ($p = 0.04$) which in turn was positively associated with asthma development ($p = 0.01$).<br><br>Furthermore, rs1800872 and rs1800896 in IL10 were associated with altered IL10 mRNA expression ($p < 0.01$).<br><br>Exhaled levels of IL4, IL10, and IL13 were positively associated with asthma development ($p < 0.01$).	ICAM1 is associated with asthma development on DNA, mRNA, and protein level. Thus, ICAM1 is likely to be involved in the development of childhood asthma.

The association of genetic variants in toll-like receptor 2 subfamily with allergy and asthma after hospitalization for bronchiolitis in infancy<br><br>Koponen P, 2014<br><br>[7]	Longitudinal study	To investigate the role of polymorphisms in genes regulating TLR2 subfamily, which may play a significant role in determining which children are in elevated risk for asthma and/or atopy after bronchiolitis in early infancy.	Full-term infants <6 months of age and hospitalized due to bronchiolitis in the Department of Pediatrics, Tampere University Hospital, Finland. Recruited during December 1, 2001, and May 31, 2002, and between October 28, 2002, and May 31, 2004.	133 children	Asthmatic, other respiratory and allergic symptoms and asthma or allergy diagnosed during the 6 first years of life, were reviewed with an extensive questionnaire.<br><br>The genotyping of single nucleotide polymorphisms (SNPs) TLR1 rs5743618, TLR2 rs5743708 and TLR6 rs5743810 was performed by pyrosequencing.	Twenty-four (24%) children homozygous for major allele G at TLR1 rs5743618 were diagnosed to have asthma between 1 and 6 years of age [vs. 13(38%) of those with G/T or T/T genotypes; P = 0.04]. This association was robust to further statistical adjustments with age and sex (aOR: 0.48, 95% confidence intervals: 0.2–0.9; Table 1).<br><br>Of 60 children, 11 (18%) with TLR6 rs5743810 C/T genotype versus 36/73(49%) of other genotypes had atopic eczema at follow up. After adjustment with gender and age, the finding remained statistically significant (aOR: 0.25; 95% confidence intervals: 0.11–0.56; Table 3).<br><br>Of the 102 TLR6 rs5743810 allele C carriers, 38 (37%) had current allergic rhinitis versus 19(61%) noncarriers. After adjustment with gender and age, the finding remained statistically significant, aOR 0.36 (0.16–0.83; Table 3).	Preliminary evidence was found that TLR1 rs5743618 was associated with asthma prevalence during first 6 years of life after bronchiolitis at age <6 months.<br><br>In addition, TLR1 rs5743618 and TLR6 rs5743810 were associated with allergic rhinitis at preschool age.<br><br>TLR2 rs5743708 did not show any correlation with asthma or allergic rhinitis.

Cadherin-related family member 3, a childhood asthma susceptibility gene product, mediates rhinovirus C binding and replication<br><br>Bochkov YA, 2015<br><br>[8]	Genome-wide gene-expression analysis	To investigate the role of the expression of human cadherin-related family member 3 (CDHR3), that may enables the cells normally unsusceptible to RV-C infection to support both virus binding and replication.	HeLa cell-line cultures		Cell cultures, then recombinant RV-C15, RV-C15-GFP, RV-C2, RV-C41, RV-A16, and RV-B52 were produced. Total RNA was extracted from sinus tissue or cultured cells using the RNeasy Mini kit (Qiagen). Viral RNA concentrations were determined by RT-qPCR using Power SYBR Green PCR mix.<br><br>Finally, a gene-expression analysis was made, by generating stable HeLa Cell Line Expressing CDHR3 and by fluorescent Labeling of RV-C15 and Virus Binding Assay.<br><br>Modeling of CDHR3 structure identified potential binding sites that could impact the virus surface in regions that are highly conserved among all RV-C types.	A coding SNP (rs6967330) in CDHR3, which was previously associated with wheezing illnesses and hospitalizations in childhood asthma by genetic analysis, is also associated with increased RV-C binding and progeny yields in vitro.<br><br>If the same relationship is true in vivo, rs6967330 and the surface overexpression of CDHR3 could also be a specific risk factor for more severe RV-C illnesses	Asthma susceptibility gene product CDHR3 mediates RV-C entry into host cells, and suggest that rs6967330 mutation could be a risk factor for RV-C wheezing illnesses.

Prevalence and risk factors for atopic disease in a population of preschool children in Rome: Challenges to early intervention.<br><br>Indinnimeo L, 2016<br><br>[9]	Observational study	Evaluate the prevalence of the major allergic and respiratory diseases in a group of preschool children attending nurseries in Rome and assessing the related factors, especially in relation to environment	Children attending nursery schools	494 children<br><br>From 3 to 6 years of age	Standardized questionnaire (SIDRIA-2 protocol) for the assessment of potential risk factors and outcomes. Questionnaires were distributed at school, completed at home by parents and finally delivered in anonymous form to the teachers.<br><br>The questionnaire investigated: individual characteristics such as age, gender, medical history, family history, allergies, eating habits; environmental factors such as traffic level (defined by the parents), house crowding, presence of pets at home, nursery and daycare attendance; allergic and respiratory diseases such as rhinitis, wheezing in the past 12 months, asthma diagnosed by physician, respiratory symptoms, mouth breathing and snoring during sleep, food allergy	A1. UNIVARIATE ANALYSIS: Wheezing during the last 12 months positively associated with:<br>- siblings' history of atopy □ (odds ratio [OR], 2.42; 95% confidence interval [CI], 1.39–4.22);<br>- recurrent siblings' bronchitis □ (OR, 2.32; 95% CI, 1.26–4.27);<br>- dermatitis □ (OR, 1.69; 95% CI, 0.99–2.89).<br><br>A2. UNIVARIARE ANALYSIS: diagnosis of asthma positively associated with:<br>- nationality not being Italian □ (OR, 2.54; 95% CI, 1.09–5.92);<br>- duration of breastfeeding longer than 1 month □ (OR, 2.78; 95% CI, 0.97–7.97):<br>- daycare attendance □ (OR, 2.26; 95% CI, 1.14–4.52);<br>- mother's history of atopy □ (OR, 2.04; 95% CI, 1.13–3.66);<br>-siblings' history of atopy □(OR, 2.09; 95% CI, 1.12–3.89];<br>- recurrent siblings' bronchitis □ (OR, 1.99; 95% CI, 0.99–4.00);<br>- dermatitis □ (OR, 2.02; 95% CI, 1.13–3.61);<br>- Girls had a lower risk of developing asthma with respect to boys □ (OR, 0.50; 95% CI, 0.27–0.90).<br><br>B1. MULTIVARIATE ANALYSIS: association of wheezing in the last 12	Wheezing prevalence (15.0%) was higher compared to the SIDRIA-2 study in Rome (9%) in children aged 6–7 years.<br><br>Allergic rhinitis prevalence was lower (5.5%) compared to previous Italian studies. The increase of allergic rhinitis and decrease of atopic dermatitis with age is observed.<br><br>The diagnosis of asthma was positively associated with the breastfeeding longer than 1 month.<br><br>Family size and siblings' recurrent bronchitis could be associated with a reduced risk of wheezing and allergic sensitization, respectively. The siblings' recurrent bronchitis is a protective factor for atopy.<br><br>Repeated viral infections reduce the risk for the

					or food anaphylaxis, eczema, urticaria, otitis, diarrhea, infectious diseases; medical history including therapies (e.g. bronchodilators, antihistamines, oral corticosteroids, nasal steroids, herbal medicine) received during the past year.	months with<br>- siblings' history of atopy and correlation is even stronger (ORadj, 4.43; 95% CI, 1.95–10.0);<br>protective effect of having more than one sibling becomes statistically significant (ORadj, 0.17; 95% CI, 0.04–0.76).<br><br>B2. MULTIVARIATE ANALYSIS: The analysis of asthma onset confirms an effect of gender (girls were protected compared to boys), daycare attendance, and maternal history of atopy. No statistically significance for breastfeeding longer than 1 month.<br><br>C. Urban traffic, pets, passive smoking, and house crowding do not represent significant risk factors for respiratory symptoms (small size sample?)	development of asthma up to school age, whereas no effects were observed for other types of infection<br><br>Urban traffic, pets, passiv e smoking, and house crowding do not represent significant risk factors for atopy and respiratory symptoms.

Polymorphism in the gene encoding toll-like receptor 10 may be associated with asthma after bronchiolitis.<br><br>Törmänen S, 2017<br><br>[10]	Longitudinal study	We evaluated whether post-bronchiolitis asthma was associated with polymorphisms in the TLR3 rs3775291, TLR4 rs4986790, TLR7 rs179008, TLR8 rs2407992, TLR9 rs187084, and TLR10 rs4129009 genes.	Full-term infants hospitalised for bronchiolitis at less than 6 months of age in 2002–2004 at the Department of Paediatrics, Tampere University Hospital, Finland<br><br>Follow-up at 5 to 7 years of age.	135 children	Early-life data were collected by interviewing the parents during hospitalisation using structured questionnaires.<br><br>Data on the viral aetiology of bronchiolitis were studied on admission by antigen detection and polymerase chain reaction (PCR).<br><br>Polymorphisms of TLR3 rs3775291 (1234 C/T), TLR4 rs4986790 (1194 A/G), TLR7 rs179008 (171 A/T), TLR8 rs2407992 (2040 C/G), TLR9 rs187084 (1486 T/C), and TLR10 rs4129009 (2322 A/G) were selected	Current asthma was more common (30%) in children with the variant AG or GG genotype in the TLR10 rs4129009 gene versus those who were homozygous for the major allele A (11%) ($p = 0.03$). The adjusted odds ratio (aOR) was 4.30 (95% CI 1.30–14.29).<br><br>Asthma ever was more common (34.6%) in girls with the TLR7 variant AT or TT genotype versus those who were homozygous for the major allele A (12.5%) ($p = 0.03$). The adjusted OR was 3.93 (95% CI 1.06–14.58).<br><br>Corresponding associations were not seen in boys.	There were no significant associations between TLR3, TLR4, TLR8, or TLR9 polymorphisms and post-bronchiolitis asthma. Polymorphism in the TLR10 gene increases and in the TLR7 gene may increase the risk of asthma in preschool-aged children after infant bronchiolitis.

The IL-4 rs2070874 polymorphism may be associated with the severity of recurrent viral-induced wheeze.<br><br>Amat F, 2017<br><br>[11]	Longitudinal study	To link genetic variants of asthma candidate genes to the phenotypes of early onset wheezing.	Young children presenting with recurrent wheezing from December 2013 to September 2015, from the BIOMarkers of ASthma (BIOMAS) Study, from the Department of Allergology at the Hôpital Trousseau in Paris	317 children aged 21.5 ± 7.9 months: - **cluster 1** (nonatopic uncontrolled severe wheeze), n = 207, a severe viral-induced wheeze,<br>- **cluster 2** (atopic multiple trigger wheeze), n = 61, with multiple allergic comorbidities,<br><br>- **cluster 3** (episodic viral wheeze), n = 49, a mild viral-induced wheeze.	Data collected:<br>- Sex, BMI<br>- parental history of asthma,<br>- hystory of allergic riniti and eczema,<br>- history of IgE-mediated food allergy<br><br>Infants were classified as having either VW (wheezing only during colds and without symptoms between episodes) or MTW (wheezing triggered by colds, but also other factors).<br>Finally, participants were genotyped for 16 SNPs:<br>- ADAM33 rs528557,<br>- ORMDL3 rs4065275, rs4795405, rs12603332,<br>- TSLP rs3806933,<br>- CD14 rs2569190,<br>- CDHR3 rs6967330,<br>- IL-4 rs2070874,<br>- IL-4R rs1805010, and rs1801275,<br>- IL-13 rs1800925,<br>- GSDMB rs7216389,<br>- IL-17A rs2275913,<br>- IL-5 rs2069812,<br>- IL-5RA rs2290608,<br>- C11 orf30	According to frequency analysis, the TT genotype of IL-4 rs2070874polymorphism was more frequent in cluster 1 (16%) than in clusters 2and 3 (2% for both,P= 0.002).<br><br>Only the TT genotype of IL-4rs2070874 remained significantly associated with cluster 1 (OR7.9; CI95% [2.5-25.3];P= 0.0010).	Association between the TT-genotype of IL-4rs2070874 polymorphism and a severe phenotype of viral-induced wheeze further underlines the role IL-4 plays in the inflammation pathway leading to viral respiratory infections.

					rs2155219.		

Asthmatic/wheezing phenotypes in preschool children: Influential factors, health care and urban-rural differences<br><br>Kutzora S, 2018<br><br>[12]	Cross-sectional study	First aim: to describe characteristics and potential influential factors of different asthmatic/wheezing phenotypes in preschool children.<br><br>Second aim: to ascertain differences in health care for those phenotypes, also with regard to urban-rural differences.	Population of children enrolled from a periodical and standardized collection of health data of preschool children in order to develop and evaluate intervention and prevention strategies.<br><br>Data collected in 2014/2015 from three urban and three rural regions within Bavaria.	4732 children	Parents filled out a questionnaire concerning children's health and environmental factors.<br><br>To classify respiratory symptoms, five phenotype groups were built:<br>- episodic wheeze,<br>- unremitting and frequent wheeze,<br>- ISAAC asthma,<br>- asthma<br>- physician-diagnosed asthma.<br><br>For each phenotype, health care variables were presented and stratified for residence.	Risk factors for wheezing phenotypes were:<br>- male gender (OR = 2.02, 95%-CI = [1.65–2.48]),<br>- having older siblings (OR = 1.24, 95%-CI = [1.02–1.51]),<br>- preterm delivery (OR = 1.61, 95%-CI = [1.13–2.29]) (ORs for unremitting wheeze).<br><br>57% of children with ISAAC asthma and 74% with physician-diagnosed asthma had performed allergy tests.<br><br>Medication intake among all groups was more frequent in rural areas, and physician's asthma diagnoses were more frequent in urban areas	Study confirms that male gender, older siblings and preterm delivery are associated with several wheezing phenotypes.<br><br>Overall, low numbers of allergy tests among children with physician's diagnoses highlight a discrepancy between common practice and current knowledge and guidelines.<br><br>Residential differences in health care might encourage further research and interventions strategies.

Prevalence estimates and risk factors for early childhood wheeze across Europe: the EuroPrevall birth cohort<br><br>Selby A, 2018<br><br>[13]	Birth cohort study	To assess the prevalence of early childhood wheeze across Europe and evaluate risk factors focusing on food allergy, breast feeding and smoke exposure.	Infants from nine countries were recruited into the EuroPrevall birth cohort: Reykjavik (Iceland), Southampton (UK), Amsterdam (The Netherlands), Berlin (Germany), Lodz (Poland), Vilnius (Lithuania), Madrid (Spain), Milan (Italy) and Athens (Greece).	8805 infants with 24-months follow up data	Evaluation began at birth with follow-up of participants at 12 and 24 months using standardised questionnaires based on those used in previous epidemiological studies such as ISAAC.<br><br>At recruitment, data were collected on birth details, maternal diet, family history, maternal education (as a marker of socioeconomic status) and environmental exposures, including cigarette smoke and pet ownership. The 12-month and 24-month questionnaires included an extensive list of foods found in children's diets.<br><br>Additional assessments, including skin prick testing, measurement of specific IgE with or without a DBPCFC were performed according to a standardised protocol whenever	The prevalence of wheeze in the second year of life ranged:<br>- from <2% in Lodz (Poland) and Vilnius (Lithuania)<br>- to 13.1% (95% CI 10.7% to 15.5%) in Southampton (UK) and 17.2% (95% CI 15.0% 19.5%) in Reykjavik (Iceland).<br><br>In multivariable analysis, Factors associated with wheeze:<br>- frequent lower respiratory tract infections in the first and second years of life (incidence rate ratio (IRR) 1.9 (95% CI 1.3 to 2.6) and 2.5 (95% CI 1.9 to3.4), respectively),<br>- postnatal maternal smoking (IRR 1.6, 95% CI 1.1 to 2.4),<br>- day care attendance (IRR 1.6, 95% CI 1.1 to 2.5)<br>- male gender (IRR 1.3, 95% CI 1.0 to 1.7).<br><br>Food allergy and breast feeding were not independently associated with wheeze.	The prevalence of early childhood wheeze varied considerably across Europe. Lower respiratory tract infections, day care attendance, postnatal smoke exposure and male gender are important risk factors.

					parents reported symptoms suggestive of food allergy in their children.1		

Maternal Black Race and Persistent Wheezing Illness in Former Extremely Low Gestational Age Newborns: Secondary Analysis of a Randomized Trial<br><br>Wai KC, 2018<br><br>[14]	Secondary analysis from the randomized controlled Trial of Late Surfactant	Evaluate the relationship between maternal self-reported race/ethnicity and persistent wheezing illness in former high-risk, extremely low gestational age newborns (ELGAN), and quantify the contribution of socioeconomic, environmental, and biological factors on this relationship.	Infants<br>28 0/7 weeks' gestational age (GA), who were<br><br>mechanically ventilated between 7–14 days of life, were randomized to late surfactant and inhaled nitric oxide (iNO) versus iNO-alone.	420 infants (25.2±1.2 weeks' gestation and 714±166 grams at birth, 57% male, 34% maternal black race)	Perinatal characteristics and sociodemographic data were collected at enrollment.<br><br>1. Maternal race/ethnicity<br>2. Factors known to modify respiratory morbidity in former ELGAN (additional children <5 years in the home, furry pets in the home, anticipated day care attendance and breast milk diet, maternal educational attainment, public insurance status, and parental history of asthma)<br>3. Exposure to inhaled medications, wheeze auscultated by a medical professional and diagnosis of respiratory syncytial virus (RSV) infection.<br><br>Physician diagnosis of asthma, eczema or hay fever.	189 (45%) had persistent wheezing illness.<br>Infants of black mothers had increased odds of persistent wheeze compared with infants of non-black mothers (OR=2.9, 95% CI 1.9, 4.5).<br>Only bronchopulmonary dysplasia, breast milk diet, and public insurance status were identified as mediators.<br><br>The direct effect of race accounted for 69% of the relationship between maternal race and persistent wheeze, while breast milk diet, public insurance status, and bronchopulmonary dysplasia accounted for 8%, 12%, and 10%, respectively.	Among former high-risk ELGAN, infants of black mothers have increased odds of developing persistent wheeze. A substantial proportion of this effect is directly accounted for by race, which may reflect unmeasured environmental influences, and acquired and innate biological differences.

The course of asthma: A population-based 10-year study examining asthma remission in children diagnosed with asthma in preschool.<br><br>Oluwole O, 2020<br><br>[15]	Retrospective birth cohort study	The aim is to follow-up preschool children with diagnosed asthma (with a focus on age of asthma diagnosis defined as early-onset (≤ 3 years) and late-onset (4– 6 years)) and investigate the occurrence of persistence and remission of asthma over a 10-year period following diagnosis.	Children with a diagnosis of asthma in the first 6 years of life and who had at least 10 years of follow-up after diagnosis, from Saskatchewan Ministry of Health databases.<br><br>The study includes children born between January 1, 1995 and December 31, 2014 who had either a physician visit or hospitalization for asthma during this time	22,563 children who had been diagnosed with asthma within the first 6 years of life and who had at least 10 years of follow-up after their diagnosis.	Children were categorized into one of two asthma groups: ≤ 3 years (early-onset) and > 3 years up to 6 years (late-onset).<br><br>Sex, location of residence, cohort, and allergic status were considered as covariates in this study.<br><br>At the end of the 10 year follow-up period, the following different patterns of asthma outcomes were identified : “persistent asthma”, complete remission, relapse, intermittent ashtma.	Of the study participants, 87.2% had early-onset (≤ 3 years) and 12.8% had late-onset (4–6 years) asthma.<br><br>Over the 10-years of follow-up, rate of asthma remission was 37 per 100 person-years.<br><br>Factors positively associated with remission:<br>- Early-onset asthma (HR = 1.10, 95%CI: 1.01–1.20),<br>- being female (HR = 1.12, 95%CI: 1.07–1.18),<br>- living in a rural location (HR = 1.20, 95%CI: 1.14–1.27)<br>- Living in a medium urban location (HR = 1.16, 95%CI: 1.08–1.26).<br><br>History of atopy decreased likelihood of remission (HR = 0.73, 95%CI: 0.54–0.97).	Most children with asthma experienced remission, especially those with onset of symptoms within the first 3 years of life.

Interleukin-1 receptor-associated kinase-4 gene variation may increase post-bronchiolitis asthma risk.<br><br>Korppi M., 2020<br><br>[16]	Prospective study	To evaluate the associations of six IRAK4 gene polymorphisms with presence of asthma and allergic rhinitis and use of inhaled corticosteroids (ICSs) for asthma at 5-7 and 11-13 years of ages after hospitalization for bronchiolitis at younger than 6 months of age.	Enrolled full-term infants aged <6 months that were hospitalized, between 2001-2004, due to bronchiolitis in the department<br><br>of Pediatrics, Tampere University Hospital, Finland.12	141 former bronchiolitis patients followed up until 5-7	Data on atopic dermatitis in children and asthma or allergy and use of inhaled corticosteroids (ICSs)in parents were registered through a questionnaire during hospitalization and at the control visit at 1.5 years of age.<br><br>Later follow-ups at 5-7 years of age, that included an interview of the parents and children, and exercise challenge test using impulse oscillometry.<br><br>IRAK4 rs4251513, rs4251520, rs4251522, rs4251578, rs79154645 and rs13852554 SNPs (Figure 1) were studied by PCR-based sequencing,<br><br>carried out at the Eurofins Genomics.	The homozygous variant IRAK4 rs4251513 genotype was associated with the presence of asthma and allergic rhinitis and use of ICSs at 5-7 in univariate analyses. Statistical significance remained for the presence of asthma and use of ICSs but was lost in the case of allergic rhinitis in multivariate analyses. The adjusted odds ratios were 3.48 for asthma and 5.22 for ICS use.	The homozygous variant IRAK4 rs4251513 genotype was constantly associated with post-bronchiolitis asthma and asthma medication in school-aged children.

Chromosome 17q12-21 Variants Are Associated with Multiple Wheezing Phenotypes in Childhood.<br><br>Hallmark B., 2021<br><br>[17]	Birth cohort study	To determine whether wheezing phenotypes, defined by latent class analysis (LCA), are associated with nine 17q12-21 SNPs<br><br>and if so, whether these relationships differ by race/ancestry.	Data from seven U.S. birth cohorts from the CREW (Children's Respiratory Research and Environment Workgroup) were harmonized to represent whether subjects<br><br>wheezed in each year of life from birth until age 11 years.	3,786 children	Using the Bayesian information criterion (BIC), population was sorted in four latent classes that differ in the probability of wheezing during each year of life:<br>- infrequent,<br>- transient,<br>- late-onset,<br>- persistent wheeze.<br><br>Nine SNPs, rs2941504, rs2517955, rs12936231, rs2305840, rs7216389, rs4065275, rs8076131, rs8069202, and rs3859192, were selected from the 17q12-21 locus based on previously reported<br><br>associations and LD patterns.	Most 17q SNPs were associated with higher odds ratios (ORs) for three wheezing phenotypes (non for the infrequent type).<br>In general, for each SNP, the ORs were greatest for persistent wheeze and lowest for transient wheeze, but for some SNPs (e.g., rs7216389), the ORs for transient wheeze exceeded late-onset wheeze.<br><br>The strongest signals were with the two SNPs, rs2517955 in PGAP3 and rs2305480 in GSDMB, which had the largest ORs for all three latent class comparisons.	These results indicate that 17q12-21 is a "wheezing<br><br>locus," and this association may reflect an early life susceptibility torespiratory viruses common to all wheezing children.

References

1. Simon MR, Havstad SL, Wegienka GR, Ownby DR, Johnson CC. Risk factors associated with transient wheezing in young children. Allergy Asthma Proc. 2008 Mar-Apr;29(2):161-5. doi: 10.2500/aap.2008.29.3089. PMID: 18430313.
2. Bisgaard H, Bønnelykke K, Sleiman PM, Brasholt M, Chawes B, Kreiner-Møller E, Stage M, Kim C, Tavendale R, Baty F, Pipper CB, Palmer CN, Hakonarsson H. Chromosome 17q21 gene variants are associated with asthma and exacerbations but not atopy in early childhood. Am J Respir Crit Care Med. 2009 Feb 1;179(3):179-85. doi: 10.1164/rccm.200809-1436OC. Epub 2008 Nov 21. PMID: 19029000.
3. Marenholz I, Kerscher T, Bauerfeind A, Esparza-Gordillo J, Nickel R, Keil T, Lau S, Rohde K, Wahn U, Lee YA. An interaction between filaggrin mutations and early food sensitization improves the prediction of childhood asthma. J Allergy Clin Immunol. 2009 Apr;123(4):911-6. doi: 10.1016/j.jaci.2009.01.051. PMID: 19348926.
4. Halapi E, Gudbjartsson DF, Jonsdottir GM, Bjornsdottir US, Thorleifsson G, Helgadottir H, Williams C, Koppelman GH, Heinzmann A, Boezen HM, Jonasdottir A, Blondal T, Gudjonsson SA, Jonasdottir A, Thorlacius T, Henry AP, Altmueller J, Krueger M, Shin HD, Uh ST, Cheong HS, Jonsdottir B, Ludviksson BR, Ludviksdottir D, Gislason D, Park CS, Deichmann K, Thompson PJ, Wjst M, Hall IP, Postma DS, Gislason T, Kong A, Jonsdottir I, Thorsteinsdottir U, Stefansson K. A sequence variant on 17q21 is associated with age at onset and severity of asthma. Eur J Hum Genet. 2010 Aug;18(8):902-8. doi: 10.1038/ejhg.2010.38. Epub 2010 Apr 7. PMID: 20372189; PMCID: PMC2987388.
5. Koponen P, Nuolivirta K, Virta M, Helminen M, Hurme M, Korppi M. Polymorphism of the rs1800896 IL10 promoter gene protects children from post-bronchiolitis asthma. Pediatr Pulmonol. 2014 Aug;49(8):800-6. doi: 10.1002/ppul.22909. Epub 2013 Oct 25. PMID: 24167151.
6. Klaassen EM, van de Kant KD, Jöbsis Q, Penders J, van Schooten FJ, Quaak M, den Hartog GJ, Koppelman GH, van Schayck CP, van Eys G, Dompeling E. Integrative genomic analysis identifies a role for intercellular adhesion molecule 1 in childhood asthma. Pediatr Allergy Immunol. 2014 Mar;25(2):166-72. doi: 10.1111/pai.12187. Epub 2014 Jan 6. PMID: 24393359.
7. Koponen P, Vuononvirta J, Nuolivirta K, Helminen M, He Q, Korppi M. The association of genetic variants in toll-like receptor 2 subfamily with allergy and asthma after hospitalization for bronchiolitis in infancy. Pediatr Infect Dis J. 2014 May;33(5):463-6. doi: 10.1097/INF.0000000000000253. PMID: 24445834.
8. Bochkov YA, Watters K, Ashraf S, Griggs TF, Devries MK, Jackson DJ, Palmenberg AC, Gern JE. Cadherin-related family member 3, a childhood asthma susceptibility gene product, mediates rhinovirus C binding and replication. Proc Natl Acad Sci U S A. 2015 Apr 28;112(17):5485-90. doi: 10.1073/pnas.1421178112. Epub 2015 Apr 6. PMID: 25848009; PMCID: PMC4418890.
9. Indinnimeo L, Porta D, Forastiere F, De Vittori V, De Castro G, Zicari AM, Tancredi G, Melengu T, Duse M. Prevalence and risk factors for atopic disease in a population of preschool children in Rome: Challenges to early intervention. Int J Immunopathol Pharmacol. 2016 Jun;29(2):308-19. doi: 10.1177/0394632016635656. Epub 2016 Mar 2. PMID: 26936651; PMCID: PMC5806707.
10. Törmänen S, Korppi M, Teräsjärvi J, Vuononvirta J, Koponen P, Helminen M, He Q, Nuolivirta K. Polymorphism in the gene encoding toll-like receptor 10 may be associated with asthma after bronchiolitis. Sci Rep. 2017 Jun 7;7(1):2956. doi: 10.1038/s41598-017-03429-x. PMID: 28592890; PMCID: PMC5462783.
11. Amat F, Louha M, Benet M, Guiddir T, Bourgoin-Heck M, Saint-Pierre P, Paluel-Marmont C, Fontaine C, Lambert N, Couderc R, Gonzalez JR, Just J. The IL-4 rs2070874 polymorphism may be associated with the severity of recurrent viral-induced wheeze. Pediatr Pulmonol. 2017 Nov;52(11):1435-1442. doi: 10.1002/ppul.23834. Epub 2017 Sep 26. PMID: 28950434.

12. Kutzora S, Weber A, Heinze S, Hendrowarsito L, Nennstiel-Ratzel U, von Mutius E, Fuchs N, Herr C; GME Study Group. Asthmatic/wheezing phenotypes in preschool children: Influential factors, health care and urban-rural differences. Int J Hyg Environ Health. 2018 Mar;221(2):293-299. doi: 10.1016/j.ijheh.2017.12.001. Epub 2017 Dec 24. PMID: 29330038.
13. Selby A, Munro A, Grimshaw KE, Cornelius V, Keil T, Grabenhenrich L, Clausen M, Dubakiene R, Fiocchi A, Kowalski ML, Papadopoulos NG, Reche M, Sigurdardottir ST, Sprikkelman AB, Xepapadaki P, Mills ENC, Beyer K, Roberts G. Prevalence estimates and risk factors for early childhood wheeze across Europe: the EuroPrevall birth cohort. Thorax. 2018 Nov;73(11):1049-1061. doi: 10.1136/thoraxjnl-2016-209429. Epub 2018 May 10. PMID: 29748253.
14. Wai KC, Hibbs AM, Steurer MA, Black DM, Asselin JM, Eichenwald EC, Ballard PL, Ballard RA, Keller RL; Trial of Late Surfactant (TOLSURF) Study Group. Maternal Black Race and Persistent Wheezing Illness in Former Extremely Low Gestational Age Newborns: Secondary Analysis of a Randomized Trial. J Pediatr. 2018 Jul;198:201-208.e3. doi: 10.1016/j.jpeds.2018.02.032. Epub 2018 Apr 4. PMID: 29627188; PMCID: PMC6019148.
15. Oluwole O, Rennie DC, Goodridge D, Blackburn D, Litzenberger T, Penz E, Lawson JA. The course of asthma: A population-based 10-year study examining asthma remission in children diagnosed with asthma in preschool. Pediatr Pulmonol. 2020 Aug;55(8):1924-1935. doi: 10.1002/ppul.24881. Epub 2020 Jun 9. PMID: 32478962.
16. Korppi M, Teräsjärvi J, Lauhkonen E, Törmänen S, He Q, Nuolivirta K. Interleukin-1 receptor-associated kinase-4 gene variation may increase post-bronchiolitis asthma risk. Acta Paediatr. 2021 Mar;110(3):952-958. doi: 10.1111/apa.15607. Epub 2020 Oct 16. PMID: 33020954.
17. Hallmark B, Wegienka G, Havstad S, Billheimer D, Ownby D, Mendonca EA, Gress L, Stern DA, Myers JB, Khurana Hershey GK, Hoepner L, Miller RL, Lemanske RF, Jackson DJ, Gold DR, O'Connor GT, Nicolae DL, Gern JE, Ober C, Wright AL, Martinez FD. Chromosome 17q12-21 Variants Are Associated with Multiple Wheezing Phenotypes in Childhood. Am J Respir Crit Care Med. 2021 Apr 1;203(7):864-870. doi: 10.1164/rccm.202003-0820OC. PMID: 33535024; PMCID: PMC8017591.

Question 5. Does obesity influence the onset and the evolution of preschool wheezing?

Search strategies:

("child, preschool"[MeSH Terms] OR "toddler*"[All Fields]) AND ("respiratory sounds"[MeSH Terms] OR "wheez*"[All Fields] OR "Asthma"[MeSH Terms:noexp]) AND "Risk Factors"[MeSH Terms] AND ("Age of Onset"[MeSH Terms] OR "Onset"[All Fields]) AND "english"[Language] AND 2008/01/01:2021/12/31[Date - Publication]

("child, preschool"[MeSH Terms] OR "toddler*"[All Fields]) AND ("respiratory sounds"[MeSH Terms] OR "wheez*"[All Fields] OR "Asthma"[MeSH Terms:noexp]) AND "Risk Factors"[MeSH Terms] AND ("disease progression"[MeSH Terms] OR "disease evolution"[All Fields] OR "evolution"[All Fields]) AND "english"[Language] AND 2008/01/01:2021/12/31[Date - Publication]

Title of the study, first author, year	Type of study	Objective	Population	N of patients, Age	Experiments/mechanisms assessed	Relative risk (RR) or odds ratio (OR) + confidence interval (CI 95%)	Results

Early childhood weight status in relation to asthma development in high-risk children.<br><br>Zhang Z, 2010<br><br>[1]	Prospective birth cohort study	To investigate the associations between weight status in the first 5 years and having asthma at ages 6 and 8 years with a annual follow.up<br><br>The data used are generated from the Childhood Origin of Asthma (COAST) cohort, which comprises a birth cohort genetically at high risk of asthma because of a parental history of asthma or respiratory allergies.	High-risk newborns with at least 1 asthmatic/atopic parent enrolled in the Childhood Origin of Asthma project	N = 285<br><br>From birth to age 6-8 years	Evaluation of asthma development at age 6-8 years in a high-risk children cohort	A. The prevalence of wheezing illnesses decreased from 29% in the first year of life to 14% at age 5 years. Asthma was diagnosed in 28% of children at age 6 years and 33% of children at age 8 years.<br>The prevalence of overweight status increased from 15% at age 1 year to 22% at age 3 years and then stayed about the same between 3 and 8 years of age.<br>No significant concurrent association found between overweight status and wheezing/asthma occurrence at each year of age.<br><br>B. Longitudinal analyses revealed complex relationships between being overweight and asthma:<br>- (1) Being overweight at age 1 year was associated with a decreased risk of asthma at age 6 ([OR], 0.32; *P* = .02) and 8 (OR, 0.35; *P* = .04) years, as well as better lung function.<br><br>- (2) Being overweight beyond infancy was not associated with asthma occurrence. In fact, only children who were overweight at age 5 years but not at age 1 year had an increased risk of asthma at	Association between weight and childhood asthma changes with age

						age 6 years (OR, 5.78; *P* = .05).	

Body mass index in young children and allergic disease: gender differences in a longitudinal study<br><br>Murray CS, 2011<br><br>[2]	Prospective birth cohort study	To investigate the relationship of body mass index (BMI) at 3, 5 and 8 years of age with allergic disease within a birth cohort.	Children from the Manchester Asthma and Allergy Study, an unselected population-based birth cohort.	N = 731	Children were followed from birth and were reviewed at age 3, 5 and 8 years. Parents completed questionnaires; children were weighed, measured, skin prick tested and examined.	Increasing BMI at 3, 5 and 8 years increased the risk of current wheezing at the corresponding age:<br>1) age 3 □ OR 1.26 (95% CI, 1.04–1.53; P = 0.02);<br>2) age 5 □ OR 1.33 (95% CI, 1.06–1.67; P = 0.02);<br>3) age 8 □ OR 1.27 [95% CI, 1.0–1.62; P = 0.05).<br><br>The effect of BMI on wheeze at age 8 years differed between boys and girls, with a significant positive association in girls, but not in boys (P = 0.04 for interaction).<br><br>The effect of BMI at earlier ages on current or subsequent wheezing did not differ significantly between genders.<br><br>Increasing BMI significantly increased the risk of physician-diagnosed eczema:<br>- at age 5 □ OR 1.23 (95%CI, 1.04–1.47), P = 0.02);<br>- at age 8 □ OR 1.23 (95% CI, 1.03–1.45), P = 0.02)-<br><br>There is a significant interaction between gender and BMI at age 5 (P = 0.04).<br><br>There was no association between BMI and	Being overweight is associated with an increased risk of allergic disease in childhood. However, the strength of the association varies with the gender, age and atopic phenotype.

						sensitization.<br><br>Being overweight at age 3 years was significantly associated with:<br>- late-onset wheeze OR 3.83 [95% CI, 1.51–9.75], P = 0.005);<br>- persistent wheeze OR 4.15 [95% CI2.07–8.32], P < 0.001);<br>- persistent eczema OR 1.79 [95% CI 1.03–3.13], P = 0.04).<br><br>There was no difference in these results between boys and girls.	

Body mass index trajectory classes and incident asthma in childhood: results from 8 European Birth Cohorts – A Global Allergy and Asthma European Network initiative<br><br>Rzehak P, 2013<br><br>[3]	Data analysis from the combination of 8 different prospective birth cohort studies	To investigate whether the type of course in standardized BMI according to age- and sex-specific “World Health Organization (WHO) child growth standards” and “WHO growth standards for school aged children and adolescents” for children up to age 5 years and more than 5 years of age, respectively (BMI-SDS), affected incident asthma over the first 6 years of life.	Subjects of 8 European birth cohorts on asthma and allergies (GINIplus, LISAplus, MAS, DARC, PIAMA-NHS, PIPO, AMICS-Barcelona, AMICS-Menorca)	N = 12.050	BMI and doctor-diagnosed asthma were modeled during the first 6 years of life with latent growth mixture modeling and discrete time hazard models. Subpopulations of children were identified with similar standardized BMI trajectories.<br><br>These types of growth profiles were analyzed as predictors for incident asthma.	Children with a rapid BMI-SDS gain in the first 2 years of life had a higher risk for incident asthma up to age 6 years than children with a less pronounced weight gain slope in early childhood □ HR = 1.3 (95% CI, 1.1-1.5), after adjustment for birth weight, weight-for-length at birth, gestational age, sex, maternal smoking in pregnancy, breast-feeding, and family history of asthma or allergies.<br><br>A rapid BMI gain at 2 to 6 years of age in addition to rapid gain in the first 2 years of life did not significantly enhance the risk of asthma.	Rapid growth in BMI during the first 2 years of life increases the risk of asthma up to age 6 years.

Nutritional status and childhood wheezing in rural Bangladesh<br><br>Hawlader MD, 2014<br><br>[4]	Cross-sectional study (nested within a large-scale randomized clinical trial of nutrition interventions in pregnancy: the Maternal and Infant Nutrition Intervention in Matlab (MINIMat) Trial	To investigate the association between current childhood nutritional status and current wheezing among pre-school children in rural Bangladesh.	Children from Matlab region, rural Bangladesh	N = 912<br><br>Age= 4,5 years	Anthropometric measurements of the mothers and their children were taken during a 1-year period from December 2007 to November 2008. Current wheezing was identified using the International Study of Asthma and Allergies in Childhood questionnaire.<br>Serum total IgE was measured by human IgE quantitative ELISA. IgE specific antibody to dust mites (*Dermatophagoides pteronyssinus*) was measured by the CAP-FEIA system (Phadia AB, Uppsala, Sweden)	Wheezing at 4·5 years old was significantly associated with:<br>A) stunting □ OR = 1,58; 95 % CI 1,13 - 2,22.<br>B) underweight □ OR = 1,39; 95 % CI 1,00 – 1,94.<br><br>The association with stunting remained significant after adjustment for sex, birth weight, birth length, gestational age at birth, mother's parity, maternal BMI, family history of asthma, socio-economic status, season of birth and intervention trial arm □ OR = 1,74; 95 % CI 1,19 - 2,56.	Stunting was a significant risk factor for wheezing among rural Bangladeshi children.<br><br>Further studies are necessary to confirm the relationship between nutritional status and allergic illnesses in developing countries.

Effect of fetal and infant growth on respiratory symptoms in preterm-born children<br><br>Lowe J, 2017<br><br>[5]	Cross-sectional population study	To assess if the increased respiratory symptoms associated with altered fetal growth and infant weight gain were mediated by early factors.	Cohort of preterm- and term-born children, aged 1-10 years.	7149 children (4284 pre-term born and 2865 term-born)	Respiratory outcomes obtained from a respiratory questionnaire were regressed on measures of fetal growth and infant weight gain between birth and nine months of age, then adjusted for covariates.	Association between increased wheeze-ever in preterm-born children and accelerated fetal growth between the 1st trimester and birth (OR 2.01; 95%CI 1.25, 2.32), and between the 2nd trimester and birth (1.60; 1.15, 2.22).<br><br>Rapid infant weight gain was associated with increased wheeze-ever (1.22; 1.02, 1.45);<br><br>Children born ≤32 weeks' gestation exhibiting rapid weight gain had fivefold higher risk of wheeze-ever compared to term-born without weight gain.<br><br>Current maternal smoking and gestational age were identified as candidate mediating effects.	Antenatal and postnatal growth rates are important for future respiratory health in preterm-born children, and that their effects may be mediated by modifiable factors. Minimizing exposure to environmental pollutants, especially maternal tobacco smoking, may improve outcomes.

The effect of catch-up growth in the first year of life on later wheezing phenotypes<br><br>Kotecha SJ, 2020<br><br>[6]	Observational study	To investigate if catch-up growth in the first year of life is associated with any specific wheezing phenotypes	Cohort of term-born children	6161 children<br><br>From 3 to 11 years of age	Data on respiratory symptoms were collected at 3, 5, 7 and 11 years of age at face-to-face interviews by trained interviewers .Multinomial logistic regression was performed and relative risk ratios and corresponding 95% confidence intervals for associations between catch-up growth in the first year and wheezing phenotypes are reported. The "No wheezing" class was used as the reference group.<br><br>Adjustments were made for important early-life factors associated with wheezing in later life including sex, IUGR, antenatal and postnatal smoking, social	A. Children who were born with IUGR were associated with early wheeze but had a lower risk of developing late wheeze:<br>– IUGR and early wheeze: RR 1.15 (0.91–1.45) , p=0.17;<br>– IUGR and late wheeze: RR 0.46 (0.24–0.88) p=0.02<br><br>The association between catch-up growth and the early wheeze phenotype remained after adjustments for early-life factors (IUGR, antenatal and postnatal smoking, social economic status, breastfeeding, childcare, postnatal smoking, and cesarean delivery):<br>- RR 1.21 (1.03–1.42), p = 0.02.<br><br>Linear relationship between increase in weight gain z-scores and odds ratio of early wheeze ranging from 1.2 (95% CI 1.06–1.35) for increase of >0.4 weight gain z-score to 1.53 (1.22–1.93) for z-score	Catch-up growth is associated with early wheeze, but not with persistent or late wheeze. As catch-up growth is generally promoted by clinicians for infants born with IUGR, it is important that the longer-term effect of catch-up growth is well defined and the risks and benefits clearly delineated.

					economic status, breastfeeding, childcare, postnatal smoking, and cesarean delivery.	of >2.0.	

References

1. Zhang Z, Lai HJ, Roberg KA, Gangnon RE, Evans MD, Anderson EL, Pappas TE, Dasilva DF, Tisler CJ, Salazar LP, Gern JE, Lemanske RF Jr. Early childhood weight status in relation to asthma development in high-risk children. J Allergy Clin Immunol. 2010 Dec;126(6):1157-62. doi: 10.1016/j.jaci.2010.09.011. Epub 2010 Nov 4. PMID: 21051081; PMCID: PMC2998556.
2. Murray CS, Canoy D, Buchan I, Woodcock A, Simpson A, Custovic A. Body mass index in young children and allergic disease: gender differences in a longitudinal study. Clin Exp Allergy. 2011 Jan;41(1):78-85. doi: 10.1111/j.1365-2222.2010.03598.x. Epub 2010 Aug 16. PMID: 20718779.
3. Rzehak P, Wijga AH, Keil T, Eller E, Bindslev-Jensen C, Smit HA, Weyler J, Dom S, Sunyer J, Mendez M, Torrent M, Vall O, Bauer CP, Berdel D, Schaaf B, Chen CM, Bergström A, Fantini MP, Mommers M, Wahn U, Lau S, Heinrich J; GA²LEN-WP 1.5 Birth Cohorts. Body mass index trajectory classes and incident asthma in childhood: results from 8 European Birth Cohorts--a Global Allergy and Asthma European Network initiative. J Allergy Clin Immunol. 2013 Jun;131(6):1528-36. doi: 10.1016/j.jaci.2013.01.001. Epub 2013 Feb 10. PMID: 23403049.
4. Hawlader MD, Noguchi E, El Arifeen S, Persson LÅ, Moore SE, Raqib R, Wagatsuma Y. Nutritional status and childhood wheezing in rural Bangladesh. Public Health Nutr. 2014 Jul;17(7):1570-7. doi: 10.1017/S1368980013001262. Epub 2013 May 17. PMID: 23680045.
5. Lowe J, Kotecha SJ, Watkins WJ, Kotecha S. Effect of fetal and infant growth on respiratory symptoms in preterm-born children. Pediatr Pulmonol. 2018 Feb;53(2):189-196. doi: 10.1002/ppul.23920. Epub 2017 Nov 23. PMID: 29168321.
6. Kotecha SJ, Lowe J, Granell R, Watkins WJ, Henderson AJ, Kotecha S. The effect of catch-up growth in the first year of life on later wheezing phenotypes. Eur Respir J. 2020 Dec 24;56(6):2000884. doi: 10.1183/13993003.00884-2020. PMID: 32586886; PMCID: PMC7758542.

Question 6. Do prematurity and other perinatal factors influence the onset and the evolution of preschool wheezing?

Search strategy:

("child, preschool"[MeSH Terms] OR "toddler*"[All Fields]) AND ("respiratory sounds"[MeSH Terms] OR "wheez*"[All Fields] OR "Asthma"[MeSH Terms:noexp]) AND ("infant, premature"[MeSH Terms] OR "prematur*"[All Fields]) AND "english"[Language] AND 2008/01/01:2021/12/31[Date - Publication]

("child, preschool"[MeSH Terms] OR "toddler*"[All Fields]) AND ("respiratory sounds"[MeSH Terms] OR "wheez*"[All Fields] OR "Asthma"[MeSH Terms:noexp]) AND "Risk Factors"[MeSH Terms] AND ("Age of Onset"[MeSH Terms] OR "Onset"[All Fields]) AND "english"[Language] AND 2008/01/01:2021/12/31[Date - Publication]

("child, preschool"[MeSH Terms] OR "toddler*"[All Fields]) AND ("respiratory sounds"[MeSH Terms] OR "wheez*"[All Fields] OR "Asthma"[MeSH Terms:noexp]) AND "Risk Factors"[MeSH Terms] AND ("disease progression"[MeSH Terms] OR "disease evolution"[All Fields] OR "evolution"[All Fields]) AND "english"[Language] AND 2008/01/01:2021/12/31[Date - Publication]

Title of the study, first author, year	Type of study	Objective	Population	N of patients, Age	Experiments/mechanisms assessed	Relative risk (RR) or odds ratio (OR) + confidence interval (CI 95%)	Results

Prematurity, chorioamnionitis, and the development of recurrent wheezing: A prospective birth cohort study<br><br>Kumar R, 2008<br><br>[1]	Prospective birth cohort study	Evaluate the association between prematurity and wheezing accounting for the presence of perinatal chorioamnionitis using subjects enrolled in the Boston Birth Cohort, a large-scale, multiethnic, inner-city population–based study, which includes both term and preterm children.	Boston Birth Cohort (n= 1096) □ Term and Preterm children	Term children = 771<br><br>Preterm children = 325<br><br>From birth to a mean age of 2.2 ± 2.0 years	Perinatal and postnatal clinical data and placental pathology were collected. Preterm children were grouped by gestational age into moderately (33-36.9 weeks) and very preterm (<33 weeks) with and without chorioamnionitis and compared with term children without chorioamnionitis (reference group). Chorioamnionitis was diagnosed either by intrapartum fever or by placental histology findings. Logistic regression models were performed to investigate the independent and joint associations of degree of prematurity and chorioamnionitis.	A. Prematurity was associated with recurrent wheezing □ (odds ratio [OR], 1.7; 95% CI, 1.2-2.6).<br><br>A1. When subjects were grouped by degree of prematurity with or without chorioamnionitis, the highest risk of wheezing (OR, 4.0; 95% CI, 2.0-8.0) and physician-diagnosed asthma (OR, 4.4; 95% CI, 2.2-8.7) was present in the very preterm children with chorioamnionitis.<br><br>A2. The effect on both wheezing (OR, 5.4; 95% CI, 2.4-12.0) and asthma (OR, 5.2; 95% CI, 2.3-11.9) was greater in African Americans.<br><br>B. Neither prematurity nor chorioamnionitis was associated with food allergy or eczema.	The primary outcome □ recurrent wheezing (≥2 physician documented episodes).<br><br>Secondary outcomes □ physician-diagnosed asthma, food allergy, and eczema.

Late preterm birth and risk of developing asthma.<br><br>Abe K, 2010<br><br>[2]	Retrospective birth cohort study	To evaluate the association between gestational age at birth (late preterm vs term) and risk for physician-diagnosed asthma.	6189 singleton children born at 34- 41 completed weeks of gestation; data from the Third National Health and Nutrition Examination Survey (NHANES III, 1988-1994	Born late preterm, between 34 and 36 completed weeks of gestation n = 537<br><br>Born term, between 37 and 41 completed weeks of gestation n = 5650<br><br>age 2-83 months at the time<br><br>of survey participation		Physician-diagnosed asthma was weakly associated with late preterm birth (HR, 1.3; 95% confidence interval, 0.8-2.0).<br><br>This association was not statistically significant (*P* = 0.30).	Late preterm birth was not associated with a diagnosis of asthma in early childhood.

Pregnancy exposures and risk of childhood asthma admission in a population birth cohort<br><br>Algert CS, 2011<br><br>[3]	Retrospective population-based study	To develop a multivariable risk factor model of asthma hospitalization and to use the model to explore the strength of association with risk factors from the pregnancy and neonatal period	All singleton births to residents of New South Wales (NSW) Australia from 1 January 2001 through 31 December 2003 who survived to at least 2 yrs of age were included in the study population.<br><br>Data were available from longitudinally linked population datasets.	7245 (3.0%) with at least one asthma admission between their 2nd and 5th birthday<br><br>From 2 to 5 years of age	multivariable risk factor model of preschooler childhood asthma ammission	A. *In utero* infectious exposures associated with childhood asthma were:<br>1) maternal antenatal admission with a urinary tract infection (UTI) □ [adjusted odds ratio (aOR) = 1.49, 95% CI (1.23–1.79)]<br>2) pre-term pre-labor rupture of membranes (PROM) □ [aOR = 1.23 (1.04–1.45)].<br><br>B. No evidence that gestational age at time of first antenatal UTI admission (<28, ≥28 wks) affected the risk of asthma (homogeneity test p = 0.6).<br><br>C. Pre-term birth was a risk factor for asthma admission, with the risk decreasing by 5.3% with each extra week of gestation. Gestational age included as a continuous variable (aOR = 0.947 □ the risk decreased by 5.3% (95% CI 3.8–6.7%) for each extra week of gestation).<br><br>D. Autumn and winter conceptions were associated with an increased risk of childhood asthma admission: winter aOR = 1.15 (1.08–1.23), autumn aOR = 1.09 (1.02–1.16).	An increased risk of asthma admission in children is associated with birth factors including *in utero* exposures, even after adjustment for other maternal and pregnancy factors. Among the factors related to birth, the largest increases in risk are associated with pre-term birth and with fetal exposure to maternal UTI and PROM.

Effects of gestational age at birth on health outcomes at 3 and 5 years of age: population based cohort study<br><br>Boyle EM, 2012<br><br>[4]	Prospective longitudinal birth cohort study	Secondary analysis of data from the Millennium Cohort Study (MCS) to investigate the burden of later disease associated with moderate/late preterm (32-36 weeks) and early term (37-38 weeks) birth.	18 818 infants participated in the MCS.<br><br>Effects of gestational age at birth on health outcomes at 3 (n=14 273) and 5 years (n=14 056) of age were analysed.	N = 18.818<br><br>From birth to 5 years of age	Evaluation of growth, hospital admissions, longstanding illness/disability, **wheezing/asthma**, use of prescribed drugs, and parental rating of their children's health.<br><br>Height, weight, and body mass index were assessed at 3 and 5 years.<br><br>Parental reports of the number of hospital admissions not related to accidents since birth or the previous interview were collected at 9 months and at 3 and 5 years. Parental reports of any longstanding illness, disability, or infirmity of more than three months' duration and diagnosed by a health professional were collected at 3 and 5 years. Parental reports of wheezing within the previous 12 months and asthma were collected at 3 and 5 years. Parental reports of the use of prescribed drugs were collected at 5	A. Wheezing or whistling in chest in previous 12 months at 3 years in:<br>1) <32 weeks of gestational age □ aOR 2.6 (1.7 – 4.0)<br>2) from 32 to 33 weeks of gestational age □ aOR 1.7 (1.1 – 2.6)<br>3) from 34 to 36 weeks of gestational age □ aOR 1.3 (1.0 – 1.5)<br>4) from 37 to 38 weeks of gestational age □ aOR 1.1 (1.0 – 1.2)<br>1) from 39 to 41 weeks of gestational age □ aOR 1<br><br>B. Wheezing or whistling in chest in previous 12 months at 5 years in:<br>1) <32 weeks of gestational age □ aOR 2.9 (1.9 – 4.6)<br>2) from 32 to 33 weeks of gestational age □ aOR 1.7 (1.0 – 2.8)<br>3) from 34 to 36 weeks of gestational age □ aOR 1.5 (1.2 – 1.8)<br>4) from 37 to 38 weeks of gestational age □ aOR 1.2 (1.0 – 1.3)<br>1) from 39 to 41 weeks of gestational age □ aOR 1<br><br>Results adjusted for child's age at interview, sex, and ethnicity; maternal age at birth, marital status, education, and occupation; whether child was mother's firstborn; duration of breast feeding; and maternal	Wheezing or whistling in chest in previous 12 months □ showed a gradient of effect with increasing prematurity, but with particularly high odds in children born very preterm

					years. Parents' ratings of children's health as excellent, very good, good, fair, or poor were collected at 5 years.	smoking and alcohol intake during pregnancy.	

Maternal smoking during pregnancy, prematurity and recurrent wheezing in early childhood<br><br>Robison RG, 2012<br><br>[5]	Prospective longitudinal birth cohort study	Maternal antenatal and postnatal exposure was determined from standardized questionnaires. Gestational age was assessed by the first day of the last menstrual period and early prenatal ultrasound (preterm < 37 weeks gestation). Wheezing episodes were determined from medical record extraction of well and ill/unscheduled visits. study visits were conducted at 6 to 12 months, 2 years, 4 years and 6 years in alignment with the child's pediatric primary care visit schedule. Mothers were interviewed with a standardized postnatal health questionnaire.	Children with smoke exposure	N = 1448<br><br>From birth to 6 years of age	To evaluate the interactive effects of maternal smoking and prematurity upon the development of early childhood wheezing.<br><br>The primary outcome was recurrent wheezing, defined as ≥ 4 episodes of physician documented wheezing. Logistic regression models and zero inflated negative binomial regression (for number of episodes of wheeze) assessed the independent and joint association of prematurity and maternal antenatal smoking on recurrent wheeze, controlling for relevant covariates.	A. Prematurity ([OR] 2.0; 95% confidence interval [CI], 1.3-3.1) was associated with an increased risk of recurrent wheezing<br><br>B. In utero maternal smoking was not associated with an increased risk of recurrent wheezing (OR 1.1, 95% CI 0.5-2.4).<br><br>C. Jointly, maternal smoke exposure and prematurity caused an increased risk of recurrent wheezing (OR 3.8, 95% CI 1.8-8.0).<br><br>C1. There was an interaction between prematurity and maternal smoking upon episodes of wheezing (P = 0.049).	Demonstration of an interaction between maternal smoking during pregnancy and prematurity on childhood wheezing in a urban, multiethnic birth cohort.

Preterm birth and childhood wheezing disorders: a systematic review and meta-analysis.<br><br>Been JV, 2014<br><br>[6]	Systematic review and meta-analysis	Two reviewers independently searched seven online databases for contemporaneous (1 January 1995-23 September 2013) epidemiological studies investigating the association between preterm birth and asthma/wheezing disorders. Additional studies were identified through reference and citation searches, and contacting international experts. Quality appraisal was undertaken using the Effective Public Health Practice Project instrument. We pooled unadjusted and adjusted effect estimates using random-effects meta-analysis, investigated "dose-response" associations, and undertook subgroup,	Preterm children	1,543,639 children<br><br>6 months to 18 years of age		Preterm birth was associated with an increased risk of wheezing disorders in unadjusted (13.7% versus 8.3%; odds ratio [OR] 1.71, 95% CI 1.57-1.87; 26 studies including 1,500,916 children) and adjusted analyses (OR 1.46, 95% CI 1.29-1.65; 17 studies including 874,710 children).<br><br>The risk was particularly high among children born very preterm (<32 wk gestation; unadjusted: OR 3.00, 95% CI 2.61-3.44; adjusted: OR 2.81, 95% CI 2.55-3.12).<br><br>Findings were most pronounced for studies with low risk of bias and were consistent across sensitivity analyses. The estimated population-attributable risk of preterm birth for childhood wheezing disorders was ≥3.1%.	Preterm birth-particularly very preterm birth-increases the risk of asthma

		sensitivity, and meta-regression analyses to assess the robustness of associations. We identified 42 eligible studies from six continents. Twelve were excluded for population overlap, leaving 30 unique studies.					

Prenatal stress and childhood asthma in the offspring: role of age at onset<br><br>Liu X, 2015<br><br>[7]	Cohort study	To investigate whether there is an association between prenatal stress and asthma, and if so, whether such an association differs according to age at asthma onset.	All live singletons born during 1996–2007 in Denmark	750 058 children	Identification of a bereaved group: children born to mothers who lost a close relative 1 year prior to or during pregnancy as the bereaved group.<br><br>Using Cox proportional hazards regression model, were evaluated the hazard ratios (HRs) for asthma in children of bereaved mothers, compared with children of non-bereaved mothers.	Prenatal stress associated with a marginally increased risk of asthma events in children aged 0–3 years [HR = 1.04, 95% confidence interval (CI): 1.00–1.07].<br><br>Unexpected bereavement was associated with a higher risk (HR = 1.13, 95% CI: 1.02–1.24).<br><br>There was no association between prenatal bereavement and asthma in children aged 4–15 years (HR = 1.02, 95% CI: 0.96–1.09).	Prenatal stress is possibly associated with asthma events in children aged 0–3 years, but not with asthma in children aged 4–15 years irrespective of age at asthma onset.

Risk of Asthma in Late Preterm Infants: A Propensity Score Approach<br><br>Voge GA, 2015<br><br>[8]	Population-based birth cohort study	Asthma status during the first 7 years of life was assessed by applying predetermined criteria. The pro-pensity score was formulated using 15 covariates by fitting a logistic regression model for late preterm birth versus term birth. We applied the propensity score method to match late preterm infants (34 0/7 to 36 6/7 weeks of gestation) to term infants (37 0/7 to 40 6/7 weeks of gestation) within a caliper of 0.2 standard deviation of logit of propensity score.	Children born in Rochester, Minn, between 1976 and 1982.	7040 children □ 5915 with complete data.<br><br>From 0 to 8 years of age.<br><br>(Median duration of follow-up: 5.1 years)	Propensity score	Before propensity score matching, late preterm infants had a higher risk of asthma □ (20 of 262, 7.6%) compared with full-term infants □ (272 of 5653, 4.8%) (P = 0.039).<br><br>Significant covariate imbalance between comparison groups.<br><br>After matching with propensity scores:<br>- former late preterm infants had a similar risk of asthma to the matched full- term infants □ (6.6% vs 7.7%, respectively, P = 0.61).<br><br>The result was consistent with covariate-adjustment Cox regression models controlling for significant covariates (P = 0 .57).	A late preterm birth history is not independently associated with childhood asthma.<br><br>The previously reported risk of asthma among former late preterm infants appears to be due to covariate imbalance.

Population-Attributable Risk of Risk Factors for Recurrent Wheezing in Moderate Preterm Infants During the First Year of Life.<br><br>Blanken MO, 2016<br><br>[9]	Multicentre prospective birth cohort study	Provide population-attributable risks (PAR) of risk factors for recurrent wheezing during the first year of life in otherwise healthy moderate preterm infants.	Population of moderate preterm infants (defined as infants born at 32 weeks and 1 day to 35 weeks and 6 days' gestational age, referred to as32–35 wGA) in one university hospital and 40 regional hospitals of the Dutch RSV Neonatal Net- work in the Netherlands.	3952 one year-old moderate preterm infants	Fourteen potentially modifiable viral exposure variables were independently associated with recurrent wheezing:<br>- Day-care attendance<br>- Male sex<br>- Bronchiolitis hospitalisation<br>- RSV bronchiolitis hospitalisation<br>- Non-RSV bronchiolitis hospitalisation<br>- Non-tested bronchiolitis hospitalisation<br>- Gestational age <35 weeks<br>- Presence of siblings<br>- Low education mother<br>- Paternal asthma<br>- Maternal childhood wheezing<br>- Maternal smoking<br>- Respiratory support<br>- Maternal hay fever	Strong relationship between RSV bronchiolitis hospitalisation and RW (RR 2.6; 95% confidence interval, CI, 2.2, 3.1), but a relative modest PAR (8%; 95% CI 6, 11%) which can be explained by a low prevalence (13%).<br><br>Day-care attendance showed a strong relationship with recurrent wheezing (RR 1.9; 95% CI 1.7, 2.2) and the highest PAR (32%; 95% CI 23, 37%) due to a high prevalence (67%).<br><br>The combined adjusted PAR for the 14 risk factors associated with recurrent wheezing was 49% (95% CI 46, 52%).	In moderate preterm infants, day-care attendance has the largest PAR for recurrent wheezing.

Gestational age at birth and wheezing trajectories at 3–11 years<br><br>Leps C, 2018<br><br>[10]	Prospective, longitudinal, observational study	Assessment of wheter being born <39 weeks' gestation is a risk factor for wheeze and asthma medication use in late childhood and early adolescence (7 and 11 years);<br><br>Evaluate the association between preterm birth and different trajectories of wheezing from the ages of 3 to 11 years.	18.018 9-months children interviewed, of whom 17.207 eligibile for the study	12.198 included in 7-year analysis<br><br>11.690 included in 11-year analysis<br><br>From 9 months to 11 years of age	Wheezing trajectories evaluated:<br>- 'early-remittent' (wheezing at ages 3 and/or 5 years but not after);<br><br>- 'late' (wheezing at ages 7 and/or 11 years but not before);<br><br>-persistent/relapsing' (wheezing at ages 3 and/or 5 and 7 and/or 11 years) wheeze.	- Birth <32 weeks and at 32–33 weeks, were associated with an increased risk of wheeze and asthma medication use at ages 7 and 11, and all three wheezing trajectories. The aOR for 'persistent/relapsing wheeze' at <32 weeks was 4.30 (95% CI 2.33 to 7.91) and 2.06 (95% CI 1.16 to 2.69) at 32–33 weeks<br><br>- Birth at 34–36 weeks was not associated with asthma medication use at 7 or 11, nor late wheeze, but was associated with the other wheezing trajectories.<br><br>-Birth at 37–38 weeks was not associated with wheeze nor asthma medication use.	Adjusted ORs (aOR) were estimated for recent wheeze and asthma medication use for children born <32, 32–33, 34–36 and 37–38 weeks' gestation, compared with children born at full term (39–41 weeks) at 7 (n=12 198) and 11 years (n=11 690). aORs were also calculated for having 'early-remittent' (wheezing at ages 3 and/or 5 years but not after), 'late' (wheezing at ages 7 and/or 11 years but not before) or 'persistent/relapsing' (wheezing at ages 3 and/or 5 and 7 and/or 11 years) wheeze.

Maternal Stress During Pregnancy and Allergic Diseases in Children During the First Year of Life.<br><br>Smejda K, 2018<br><br>[11]	Multi-center prospective cohort study	To assess the association between exposure to different kinds of prenatal stress and the occurrence of atopic dermatitis, food allergy, wheezing, and recurrent respiratory tract infections in children.	Mother-child pairs from a Polish Mother and Child Cohort, restricted to women that worked at least one month during pregnancy	370 mother-child pairs followed from pregnancy to 2 y of age	Maternal psychological stress during pregnancy was assessed based on the Subjective Work Characteristics Questionnaire, the Perceived Stress Scale, and the Social Readjustment Rating Scale.	Maternal stress during pregnancy increased the risk of wheezing in children (OR 1.09, 95% CI 1.01-1.02) independently from other predictors of wheezing.<br><br>Significant positive association between maternal life stress during pregnancy and the risk of recurrent respiratory tract infections in the first year of life (not significant after adjustment for confounding variables).	Maternal stress during pregnancy increases the risk of childhood wheezing.

Comparison of the Associations of Early-Life Factors on Wheezing Phenotypes in Preterm-Born Children and Term-Born Children.<br><br>Kotecha SJ, 2018<br><br>[12]	Millennium Cohort Study	Aim: define wheezing phenotypes in preterm-born children and investigate whether the association of early-life factors and characteristics with wheezing phenotypes was similar between preterm- and term-born children.	Cohort of children born in the United Kingdom between 2000 and 2002	A total of 1,049 preterm-born children and 12,307 term-born children.	All data were collected at face-to-face interviews At 9 months of the child's age, data were collected on preg- nancy, birth and early-life factors, and characteristics and at 3, 5, 7, and 11 years of age on respiratory symptoms.<br>Four phenotypes were defined:<br>• No wheeze/infrequent wheeze: no or infrequent wheezing through the 4 time points. Wheezing at none of the time points or at 1 time point only.<br>• Early wheeze: wheezing reported at 3 years of age and dis- appearing by 7 or 11 years of age.<br>• Persistent wheeze: wheezing that persisted throughout the study period.<br><br>• Late wheeze: no wheeze reported before the age of 7 years but developing at age 7 years or beyond.	Preterm-born children were more likely to develop early wheeze (odds ratio = 1.6, 95% confidence interval: 1.3, 1.9; $P < 0.001$) and persistent wheeze (odds ratio = 1.6, 95% confidence interval: 1.3, 1.9; $P < 0.001$) but not late wheeze (odds ratio = 1.0, 95% confidence interval: 0.7, 1.5; $P = 0.90$) when compared with term-born children.	Although early-life factors and characteristics were similarly associated with the wheezing phenotypes in both groups, the preterm-born group had higher rates of early and persistent wheeze.

Recurrent wheezing in preterm infants: Prevalence and risk factors.<br><br>Simoes MCRDS, 2019<br><br>[13]	Cross-sectional study	To evaluate the prevalence and risk factors associated with progression to recurrent wheezing in preterm infants.	Preterm infants born between 2011 and 2012, with a GA <37 weeks	445 children aged 39 (18-54) months.<br>- 194 with passive immunization against RSV<br>- 251 not immunized	Research tool: a questionnaire directed at risk<br><br>factors associated with the reduced version of the EISL17 questionnaire, which investigated demographic data and risk factors for RW such as gender, ethnicity, maternal schooling, birth weight, GA at birth, breastfeeding, day-care attendance, exposure to pets, number of children in the same household, maternal smoking, caregiver smok- ing, personal history of food allergy and atopic dermatitis, parental history of food allergy or asthma, and protection against severe RSV infection through the use of Palivizumab.	- Univariate analysis: the risk factors with the greatest chance of recurrent wheezing were birth weight <1000 g, gestational age <28 weeks, living with two or more siblings, food allergy, and atopic dermatitis in the child, as well as food allergy and asthma in the parents.<br>- Multivariate analysis: there was a significant association between recurrent wheezing and gestational age at birth <28 weeks, food allergy and atopic dermatitis in the child, and living with two or more children.<br><br>The overall prevalence of recurrent wheezing was 27.4% (95% CI: 23.42-31.70), whereas in the children who received passive immunization it was 36.1% (95% CI: 29.55-43.03).	Personal history of atopy, lower gestational age, and living with two or more children had a significant association with recurrent wheezing. Children with lower gestational age who received passive immunization against the respiratory syncytial virus had a higher prevalence of recurrent wheezing than the group with higher gestational age.

Preterm birth is associated with higher prevalence of wheeze and asthma in a selected population of Japanese children aged three years<br><br>Takata N, 2019<br><br>[14]	Cross-sectional study	To investigate the associations between low birth- weight (LBW), high birthweight, preterm birth (PTB), postterm birth, small for gestational age (SGA), and large for gestational age (LGA) and the prevalence of wheeze and asthma.	Japanese children aged three years (age range, 33-54 months; mean age, 38.7 months)	6364 children:<br>- 8.8% were classified as LBW (<2500 g),<br>- 90.4% as normal birthweight,<br>- 0.8% as high birthweight (≥4000 g),<br>- 4.8% as PTB (<37 weeks),<br>- 94.8% as term birth,<br>- 0.4% as postterm birth (≥42 weeks),<br>- 7.8% as SGA (<10th percentile),<br>- 82.5% as appropriate for gestational age,<br>- 9.7% as LGA (>90th percentile)	Each subject received a physical examination at a public health center All eligible children's parents or guardians received a 38-page self-administered questionnaire, which aim is to collect all the informations regarding the potential confounding factors	The prevalence values of wheeze and asthma were 19.5% and 7.7%, respectively.<br>Compared with term birth, PTB was independently positively associated with wheeze and asthma: the adjusted ORs (95% CI) were 1.47 (1.11---1.92) and 1.52 (1.02---2.20), respectively.<br><br>No evident associations were observed between LBW, high birthweight, postterm birth, SGA, or LGA and wheeze or asthma.	PTB was signifi- cantly positively associated with childhood wheeze only in boys. Such an interaction between PTB and sex was not seen for asthma.

Effect of asthma exacerbation during pregnancy in women with asthma: a population-based cohort study<br><br>Abdullah K, 2020<br><br>[15]	Population cohort study	The objective of this study is to comprehensively evaluate the effect of asthma exacerbations in pregnant women with asthma on maternal and child health.	Mother–baby pairs identified from health administrative databases and their health outcomes, from April 1, 2003 to March 31, 2012	103424 singleton pregnancies in women with asthma.<br>- The mother cohort included women aged between 13 and 45 years with prevalent asthma during pregnancy,<br><br>- Babies born to women with prevalent asthma during pregnancy were followed from birth to 5 years of age.	The Ontario Asthma Surveillance Information System (OASIS) is a population-based, longitudinal surveillance system that uses a validated case definition of asthma to monitor its prevalence and incidence in the Ontario population.<br>Outcomes:<br>1) Maternal pregnancy-related complications;<br>2) perinatal adverse outcomes:<br><br>early childhood respiratory disorders: incidence of respiratory health outcomes (allergy, wheeze, asthma, bronchiolitis and pneumonia) during the first 5 years since birth.	Asthma exacerbation in pregnant women with asthma was associated with higher odds of pre-eclampsia (OR 1.30, 95% CI 1.12– 1.51) and pregnancy-induced hypertension (OR 1.17, 95% CI 1.02–1.33).<br><br>Babies had higher odds of low birthweight (OR 1.14, 95% CI 1.00–1.31), preterm birth (OR 1.14, 95% CI 1.01–1.29) and congenital malformations (OR 1.21, 95% CI 1.05–1.39). Children born to women with asthma exacerbation during pregnancy had elevated risk of asthma (OR 1.23, 95% CI 1.13–1.33) and pneumonia (OR 1.12, 95% CI 1.03–1.22) during the first 5 years of life.	Asthma exacerbation during pregnancy in women with asthma showed increased risk of pregnancy complications, adverse perinatal outcomes and early childhood respiratory disorders in their children

Gestational hypertension and childhood atopy: a Millennium Cohort Study analysis<br><br>Henderson I, 2021<br><br>[16]	Birth cohort-based longitudinal study	Estimate the association between hypertensive disease and asthma, hay fever, or eczema by age 5, and parentally reported early wheeze and severe wheeze.	Children born 2000–2002 in UK	12,450 mother-child pairs:<br>- 950 mothers (7.7%) had gestational hypertensive disease,<br><br>Among offspring:<br>- 4242 (35%) suffered from eczema,<br>- 1857 (15%) asthma,<br>- 1342 (10%) hay fever<br>- 3805 (31%) were symptomatic of early wheeze<br>- 924 (7.3%) with severe wheeze.	Data collection is performed in three waves collected at approximately 9 months, 3 years and 5 years of age.<br>Primary outcomes: parentally reported eczema, asthma, or hay fever at the age 5 wave.<br><br>Secondary outcomes were parentally reported symptomatic outcomes of early wheeze and severe wheeze.	Odds ratios (95% CI) for gestational hypertension and childhood asthma, hay fever, and eczema were 1.32 (1.09, 1.59), 1.22 (0.97, 1.55), and 1.12 (0.96, 1.32) respectively, adjusted for confounding.<br><br>Accounting for mediation by gestational age and caesarean delivery, odds ratios (95% CI) for the potential direct effects of gestational hyper- tension were 1.21 (0.97, 1.50), 1.17 (0.91, 1.49), and 1.11 (0.94, 1.31) for the same.	Gestational hypertension was weakly positively associated with asthma and this was partly mediated by earlier<br><br>delivery. Only a small proportion of early childhood asthma was attributable to gestational hypertensive disease in this representative UK-based birth cohort.

Better maternal quality of life in pregnancy yields better offspring respiratory outcomes: A birth cohort<br><br>Yamamoto-Hanada K, 2021<br><br>[17]	Multicenter, prospective birth cohort study	To evaluate whether maternal quality of life (QoL) and depression during pregnancy leads to wheezing, asthma, and food allergy of the offspring at 3 years of age.	Nationwide study	72,685 participants with no missing variables	All health variables were collected using the Medical Outcomes Survey Short Form-8 questionnaire with a physical component summary and a mental component summary score.	Maternal physical component summary scores of the Medical Outcomes Survey Short Form-8 questionnaire were negatively associated with offspring's asthma (adjusted odds ratio [aOR], 0.99; 95% confidence interval [CI], 0.99-1.00), current wheezing (aOR, 0.99; 95% CI, 0.99-0.99), and food allergy diagnoses (aOR, 0.99; 95% CI, 0.98-0.99) in children.<br><br>Offspring's wheezing and asthma were also associated with maternal depression and anxiety during pregnancy.	Poor maternal prenatal QoL increased the risk of wheezing, asthma, and food allergy in offspring. In addition, maternal depression and anxiety increased the risk of offspring's wheezing, asthma, and food allergy.

The Associations of Caesarean Delivery With Risk of Wheezing Diseases and Changes of T Cells in Children<br><br>Lin J, 2021<br><br>[18]	Cross-sectional study	This study aimed to assess the associations of caesarean delivery (CD) with risk of wheezing diseases and changes of immune cells in children.	Children with a mean age of 36.97 ± 40.27 months and their guardians were included in the present study in Shanghai Children's Medical Center	2079 children - 987 children (47.47%) were born by cesarean delivery (CD) - 1092 (52.53%) by vaginal delivery (VD)	Patients were divided into two groups according to the delivery mode.<br><br>As for analyses of the first episode of wheezing (FEW), children in 6 groups were extracted from total population according to the age (≥1 years old, ≥2 years old, ≥3 years old, ≥4 years old, ≥5 years old, and ≥6 years old, respectively). As for analyses of asthma, children were extracted from the total population according to the age (≥3 years old, ≥4 years old, ≥5 years old, ≥6 years old, respectively). The age-dependent association between CD and the risk of asthma, FEW and immune cells were estimated.	CD was related to increased risk of FEW by the age of 3 (adjusted OR 1.50, 95%CI 1.06, 2.12) and increased tendency to develop asthma by the age of 4 (adjusted OR 3.16, 95%CI 1.25, 9.01).<br><br>The subgroup analysis revealed that the negative effects of CD on asthma were more obvious in children without exclusive breastfeeding (adjusted OR 4.93, 95%CI 1.53, 21.96) or without postnatal smoking exposure (adjusted OR 3.58, 95%CI 1.20, 13.13).<br><br>Furthermore, compared with children born through VD, a significant change of the T cells (increased proportion of CD4+ T cells and decreased number and proportion of CD8+ T cells) were observed before the age of one in the CD group. However, the changes were insignificant in children over 1 year old.	This study showed age-dependent associations of CD with asthma and FEW in offspring. Moreover, CD appeared to have an effect on the cellular immunity in infants, the disorder of which may contribute to the development of asthma in children.

References

1. Kumar R, Yu Y, Story RE, Pongracic JA, Gupta R, Pearson C, Ortiz K, Bauchner HC, Wang X. Prematurity, chorioamnionitis, and the development of recurrent wheezing: a prospective birth cohort study. J Allergy Clin Immunol. 2008 Apr;121(4):878-84.e6. doi: 10.1016/j.jaci.2008.01.030. Epub 2008 Mar 4. PMID: 18313129; PMCID: PMC2993062.
2. Abe K, Shapiro-Mendoza CK, Hall LR, Satten GA. Late preterm birth and risk of developing asthma. J Pediatr. 2010 Jul;157(1):74-8. doi: 10.1016/j.jpeds.2010.01.008. Epub 2010 Mar 24. PMID: 20338577.
3. Algert CS, Bowen JR, Lain SL, Allen HD, Vivian-Taylor JM, Roberts CL. Pregnancy exposures and risk of childhood asthma admission in a population birth cohort. Pediatr Allergy Immunol. 2011 Dec;22(8):836-42. doi: 10.1111/j.1399-3038.2011.01206.x. Epub 2011 Sep 19. PMID: 21929593; PMCID: PMC3263424.
4. Boyle EM, Poulsen G, Field DJ, Kurinczuk JJ, Wolke D, Alfirevic Z, Quigley MA. Effects of gestational age at birth on health outcomes at 3 and 5 years of age: population based cohort study. BMJ. 2012 Mar 1;344:e896. doi: 10.1136/bmj.e896. PMID: 22381676; PMCID: PMC3291750.
5. Robison RG, Kumar R, Arguelles LM, Hong X, Wang G, Apollon S, Bonzagni A, Ortiz K, Pearson C, Pongracic JA, Wang X. Maternal smoking during pregnancy, prematurity and recurrent wheezing in early childhood. Pediatr Pulmonol. 2012 Jul;47(7):666-73. doi: 10.1002/ppul.22501. Epub 2012 Jan 30. PMID: 22290763; PMCID: PMC3756665.
6. Been JV, Lugtenberg MJ, Smets E, van Schayck CP, Kramer BW, Mommers M, Sheikh A. Preterm birth and childhood wheezing disorders: a systematic review and meta-analysis. PLoS Med. 2014 Jan 28;11(1):e1001596. doi: 10.1371/journal.pmed.1001596. PMID: 24492409; PMCID: PMC3904844.
7. Liu X, Olsen J, Agerbo E, Yuan W, Sigsgaard T, Li J. Prenatal stress and childhood asthma in the offspring: role of age at onset. Eur J Public Health. 2015 Dec;25(6):1042-6. doi: 10.1093/eurpub/ckv129. Epub 2015 Jun 25. PMID: 26116689.
8. Voge GA, Katusic SK, Qin R, Juhn YJ. Risk of Asthma in Late Preterm Infants: A Propensity Score Approach. J Allergy Clin Immunol Pract. 2015 Nov-Dec;3(6):905-10. doi: 10.1016/j.jaip.2015.03.018. Epub 2015 May 2. PMID: 25944734; PMCID: PMC4630211.
9. Blanken MO, Korsten K, Achten NB, Tamminga S, Nibbelke EE, Sanders EA, Smit HA, Groenwold RH, Bont L; Dutch RSV Neonatal Network. Population-Attributable Risk of Risk Factors for Recurrent Wheezing in Moderate Preterm Infants During the First Year of Life. Paediatr Perinat Epidemiol. 2016 Jul;30(4):376-85. doi: 10.1111/ppe.12295. Epub 2016 May 16. PMID: 27199198.
10. Leps C, Carson C, Quigley MA. Gestational age at birth and wheezing trajectories at 3-11 years. Arch Dis Child. 2018 Dec;103(12):1138-1144. doi: 10.1136/archdischild-2017-314541. Epub 2018 Jun 2. PMID: 29860226; PMCID: PMC6287557.
11. Smejda K, Polanska K, Merecz-Kot D, Krol A, Hanke W, Jerzynska J, Stelmach W, Majak P, Stelmach I. Maternal Stress During Pregnancy and Allergic Diseases in Children During the First Year of Life. Respir Care. 2018 Jan;63(1):70-76. doi: 10.4187/respcare.05692. Epub 2017 Oct 17. PMID: 29042485.
12. Kotecha SJ, Watkins WJ, Lowe J, Granell R, Henderson AJ, Kotecha S. Comparison of the Associations of Early-Life Factors on Wheezing Phenotypes in Preterm-Born Children and Term-Born Children. Am J Epidemiol. 2019 Mar 1;188(3):527-536. doi: 10.1093/aje/kwy268. PMID: 30668648; PMCID: PMC6395162.

13. Simões MCRDS, Inoue Y, Matsunaga NY, Carvalho MRV, Ribeiro GLT, Morais EO, Ribeiro MAGO, Morcillo AM, Ribeiro JD, Toro AADC. Recurrent wheezing in preterm infants: Prevalence and risk factors. J Pediatr (Rio J). 2019 Nov-Dec;95(6):720-727. doi: 10.1016/j.jped.2018.06.007. Epub 2018 Jul 20. PMID: 30031764.
14. Takata N, Tanaka K, Nagata C, Arakawa M, Miyake Y. Preterm birth is associated with higher prevalence of wheeze and asthma in a selected population of Japanese children aged three years. Allergol Immunopathol (Madr). 2019 Sep-Oct;47(5):425-430. doi: 10.1016/j.aller.2018.10.004. Epub 2018 Dec 17. PMID: 30573321.
15. Abdullah K, Zhu J, Gershon A, Dell S, To T. Effect of asthma exacerbation during pregnancy in women with asthma: a population-based cohort study. Eur Respir J. 2020 Feb 12;55(2):1901335. doi: 10.1183/13993003.01335-2019. PMID: 31772000.
16. Henderson I, Quenby S. Gestational hypertension and childhood atopy: a Millennium Cohort Study analysis. Eur J Pediatr. 2021 Aug;180(8):2419-2427. doi: 10.1007/s00431-021-04012-3. Epub 2021 Mar 26. PMID: 33770273; PMCID: PMC8285347.
17. Yamamoto-Hanada K, Pak K, Saito-Abe M, Sato M, Ohya Y; Japan Environment and Children’s Study (JECS) Group. Better maternal quality of life in pregnancy yields better offspring respiratory outcomes: A birth cohort. Ann Allergy Asthma Immunol. 2021 Jun;126(6):713-721.e1. doi: 10.1016/j.anai.2021.02.019. Epub 2021 Feb 25. PMID: 33639261.
18. Lin J, Yuan S, Dong B, Zhang J, Zhang L, Wu J, Chen J, Tang M, Zhang B, Wang H, Dai Y, Liu S, Hu Y, Qi X, Xu L, Zhao L, Yin Y. The Associations of Caesarean Delivery With Risk of Wheezing Diseases and Changes of T Cells in Children. Front Immunol. 2021 Dec 14;12:793762. doi: 10.3389/fimmu.2021.793762. PMID: 34970272; PMCID: PMC8712489.

Question 7. Does smoke exposure influence the onset and the evolution of preschool wheezing?

Search strategy:

("child, preschool"[MeSH Terms] OR "toddler*"[All Fields]) AND ("respiratory sounds"[MeSH Terms] OR "wheez*"[All Fields] OR "Asthma"[MeSH Terms:noexp]) AND "Risk Factors"[MeSH Terms] AND ("Age of Onset"[MeSH Terms] OR "Onset"[All Fields]) AND "english"[Language] AND 2008/01/01:2021/12/31[Date - Publication]

("child, preschool"[MeSH Terms] OR "toddler*"[All Fields]) AND ("respiratory sounds"[MeSH Terms] OR "wheez*"[All Fields] OR "Asthma"[MeSH Terms:noexp]) AND "Risk Factors"[MeSH Terms] AND ("disease progression"[MeSH Terms] OR "disease evolution"[All Fields] OR "evolution"[All Fields]) AND "english"[Language] AND 2008/01/01:2021/12/31[Date - Publication]

Title of the study, first author, year	Type of study	Objective	Population	N of patients, Age	Experiments/mechanisms assessed	Relative risk (RR) or odds ratio (OR) + confidence interval (CI 95%)	Results

IL13 gene polymorphisms modify the effect of exposure to tobacco smoke on persistent wheeze and asthma in childhood, a longitudinal study.<br><br>Sadeghnejad A, 2008.<br><br>[1]	Birth cohort study	Investigation to assess whether there is a combined effect of interleukin-13 gene (IL13) polymorphisms and tobacco smoke on persistent childhood wheezing and asthma.	The Isle of Wight birth cohort (UK, 1989-1990)	791 children,<br><br>followed up at the ages of 1, 2, 4 and 10 years.	Three cathegories of tobacco exposition: ETS-0, 1 and 2., according to the exposures to environmental tobacco smoke (ETS) in the household and maternal smoking during pregnancy.<br><br>Stratification: defined four wheezing phenotypes:<br>- non-wheezers<br>- early-onset persistent<br>- late-onset persistent<br>- early transient<br><br>Five SNPs from the IL13 gene were used in this study:<br>- rs1800925,<br>- rs2066960,<br>- rs1295686,<br>- rs20541,<br><br>- rs1295685	Maternal smoking during pregnancy was associated with early-onset persistent wheeze (OR 2.93, $p < 0.0001$); polymorphisms in IL13 were not (OR 1.15, $p = 0.60$ for the common haplotype pair).<br><br>The effect of maternal smoking during pregnancy was stronger in children with the common IL13 haplotype pair compared to those without it (OR 5.58 and OR 1.29, respectively; p for interaction = 0.014).<br><br>Single SNP analysis revealed a similar statistical significance for rs20541 (p for interaction = 0.02).<br><br>A. Comparable results were observed for persistent childhood asthma (p for interaction = 0.03).	It is showed<br><br>a combined effect of in utero exposure to smoking and IL13 on asthma phenotypes in childhood.

Recurrent wheezing in the first three years of life: short-term prognosis and risk factors.<br><br>Sahiner UM, 2013<br><br>[2]	Cohort study	Investigation on the short-term prognosis of recurrent wheezing in very young children in whom symptoms started developing during their first 3 years of life.<br><br>Investigation on the risk factors that predict the persistence of wheezing in these children.	Children with recurrent wheezing evaluated in the period<br><br>1 January 2007 - 31 December 2010 from pediatric allergy and asthma department of Hacettepe University, (Ankara, Turkey).	529 children - 362 males<br>- 167 females<br><br>Median age of 4.4 years and a median age of 0.6 years at symptom onset.	Histories, physical findings, and diagnostic tests were all retrieved from the patients' medical records. Characteristics evaluated: symptom duration (years), SPT positivity, atopic dermatitis, prematurity, hospitalization/ICU hospitalization during the first 3 years, maternal smoking during pregnancy, breastfeeding, family atopy, presence of older siblings, pets during the first three years, number oh household members.<br><br>A stringent API (asthma prediction index) was determined in each patient.<br><br>The rate of recovery/remission was determined as children that had not experienced any wheezing and had not used a bronchodilator within the last 12 months.<br><br>Patients were subjected to Skin Prick Testing (SPT) and tested for:<br>- house dust mite mixture	A. Remission/recovery was achieved in 1.7%, 8.0%, and 14.4% of the children within 12, 24, and 36 months, respectively.<br>A. None of the factors, including gender, age at onset of symptoms, aeroallergen sensitization, family history of atopy, IgE levels, eosinophil counts, or the presence of atopic dermatitis, appeared to affect the recovery time.<br>B. However, the negative stringent API significantly shortened the duration of the recovery in children with wheezing in univariate analysis ($p = .036$).<br>C. Maternal smoking during pregnancy [(OR = 4.35; 95% CI = 1.29–14.63); ($p = .018$)]<br>The number of emergency room admissions within the first 3 years of life [(OR = 1.10; 95% CI = 1.01–1.19); ($p = .031$)] were found to be associated with an increased risk of the persistence of symptoms independently.	Most children referred with frequent wheezing remained symptomatic 3 years after the initial wheezing episode.<br><br>None of the individual factors affect the recovery time but a stringent API was related with a shorter wheezing duration.<br><br>Maternal smoking during pregnancy and emergency<br>room admissions were found to be significant risk factors<br><br>for the persistence of wheezing symptoms independently.

					- cat, dog, and mold mixture<br>- weed mixture<br>- tree pollen mixture<br><br>- grass mixture		

Early-life risk factors for childhood wheeze phenotypes in a high-risk birth cohort.<br><br>Lodge CJ, 2014<br><br>[3]	Birth-cohort study	Definition of a longitudinal childhood wheeze phenotypes in children up 7 years of age.<br><br>Investigation over the relationships of these phenotypes with earlylife exposures and wheeze at age 12 years (not consistent with pico question).	The Melbourne Atopy Cohort Study (MACS), a high-allergy-risk birth cohort that enrolled pregnant women from Melbourne, Australia between 1990 and 1994.	620 infants	Telephone surveys conducted with the infants' mothers every 4 weeks from birth to age 15 months, once at age 18 months, yearly at age 2-7 years, and once at 12 years.<br>Mothers reported each time the<br>days of cough, rattle, and wheeze experienced during the previous 4 weeks.<br><br>Potential early-life risk factors for wheeze reported:<br>- parental asthma, education, and smoking;<br>- infant sex and 4-week weight;<br>- breastfeeding;<br>- child atopy and early eczema;<br>- exposure to cats and/or dogs;<br>- lower respiratory tract infection (LRTI) in the first year;<br>- first-born status;<br>- childcare attendance by age 12 months<br><br>SPT used to test for cow's milk, egg white, peanut, house dust mite, rye grass, and cat dander allergies at age 1 and 2 years.	- Lower respiratory tract infection (LRTI) by 1 year (relative risk [RR], 3.00; 95% CI, 1.58-5.70), childcare by 1 year (RR, 1.51; 95% CI, 1.02-2.22), and higher body mass index (RR, 2.51; 95% CI, 1.09-5.81) were associated with increased risk of early transient wheeze.<br><br>- Breastfeeding was protective (RR, 0.54; 95% CI, 0.32-0.90).<br><br>- LRTI (RR, 6.54; 95% CI, 2.55-16.76) and aeroallergen sensitization (RR, 4.95; 95% CI, 1.74-14.02) increased the risk of early persistent wheeze.<br><br>- LRTI (RR, 5.31; 95% CI, 2.71-10.41), eczema (RR, 2.77; 95% CI, 1.78-4.31), aeroallergen sensitization (RR, 5.60; 95% CI, 2.86-10.9), and food sensitization (RR, 2.77; 95% CI, 1.56-4.94) increased the risk of intermediate-onset wheeze.<br><br>- Dog exposure at baseline (RR, 0.52; 95% CI, 0.32-0.84) and first-born status (RR, 0.49; 95% CI, 0.32-0.76) were protective.<br><br>- Heavy parental smoking at birth (RR, 3.18; 95% CI,	Evidence of novel insights into potential causative or aggravating environmental exposures for different childhood wheeze phenotypes.

						1.02-9.88) increased the risk of late-onset wheeze, whereas breastfeeding reduced it (RR, 0.34; 95% CI, 0.12-0.96).	

Prevalence and risk factors for atopic disease in a population of preschool children in Rome: Challenges to early intervention.<br><br>Indinnimeo L, 2016<br><br>[4]	Observational study	Evaluate the prevalence of the major allergic and respiratory diseases in a group of preschool children attending nurseries in Rome and assessing the related factors, especially in relation to environment	Children attending nursery schools	494 children<br><br>From 3 to 6 years of age	Standardized questionnaire (SIDRIA-2 protocol) for the assessment of potential risk factors and outcomes.<br>Questionnaires were distributed at school, completed at home by parents and finally delivered in anonymous form to the teachers.<br><br>The questionnaire investigated: individual characteristics such as age, gender, medical history, family history, allergies, eating habits; environmental factors such as traffic level (defined by the parents), house crowding, presence of pets at home, nursery and daycare attendance; allergic and respiratory diseases such as rhinitis, wheezing in the past 12 months, asthma diagnosed by physician, respiratory symptoms, mouth breathing and snoring during sleep, food allergy or food anaphylaxis, eczema, urticaria, otitis, diarrhea, infectious	A1. UNIVARIATE ANALYSIS: Wheezing during the last 12 months positively associated with:<br>- siblings' history of atopy □ (odds ratio [OR], 2.42; 95% confidence interval [CI], 1.39–4.22);<br>- recurrent siblings' bronchitis □ (OR, 2.32; 95% CI, 1.26–4.27);<br>- dermatitis □ (OR, 1.69; 95% CI, 0.99–2.89).<br><br>A2. UNIVARIARE ANALYSIS: diagnosis of asthma positively associated with:<br>- nationality not being Italian □ (OR, 2.54; 95% CI, 1.09–5.92);<br>- duration of breastfeeding longer than 1 month □ (OR, 2.78; 95% CI, 0.97–7.97):<br>- daycare attendance □ (OR, 2.26; 95% CI, 1.14–4.52);<br>- mother's history of atopy □ (OR, 2.04; 95% CI, 1.13–3.66);<br>-siblings' history of atopy □(OR, 2.09; 95% CI, 1.12–3.89];<br>- recurrent siblings' bronchitis □ (OR, 1.99; 95% CI, 0.99–4.00);<br>- dermatitis □ (OR, 2.02; 95% CI, 1.13–3.61);<br>- Girls had a lower risk of developing asthma with respect to boys □ (OR, 0.50; 95% CI, 0.27–0.90).	Wheezing prevalence (15.0%) was higher compared to the SIDRIA-2 study in Rome (9%) in children aged 6–7 years.<br><br>Allergic rhinitis prevalence was lower (5.5%) compared to previous Italian studies. The increase of allergic rhinitis and decrease of atopic dermatitis with age is observed.<br><br>The diagnosis of asthma was positively associated with the breastfeeding longer than 1 month.<br><br>Family size and siblings' recurrent bronchitis could be associated with a reduced risk of wheezing and allergic sensitization, respectively. The siblings' recurrent bronchitis is a protective factor for atopy.<br><br>Repeated viral infections reduce the risk for the development of asthma up to school age, whereas no

					diseases; medical history including therapies (e.g. bronchodilators, antihistamines, oral corticosteroids, nasal steroids, herbal medicine) received during the past year.	B1. MULTIVARIATE ANALYSIS: association of wheezing in the last 12 months with<br>- siblings' history of atopy and correlation is even stronger (OR$_{adj}$, 4.43; 95% CI, 1.95–10.0);<br>protective effect of having more than one sibling becomes statistically significant (OR$_{adj}$, 0.17; 95% CI, 0.04–0.76).<br><br>B2. MULTIVARIATE ANALYSIS: The analysis of asthma onset confirms an effect of gender (girls were protected compared to boys), daycare attendance, and maternal history of atopy. No statistically significance for breastfeeding longer than 1 month.<br><br>C. Urban traffic, pets, passive smoking, and house crowding do not represent significant risk factors for respiratory symptoms (small size sample?)	effects were observed for other types of infection<br><br>Urban traffic, pets, passive smoking, and house crowding do not represent significant risk factors for atopy and respiratory symptoms.

Risk factors for recurrent wheezing in infants: a case-control study.<br><br>De Sousa RB, 2016<br><br>[5]	Case-Control study	To evaluate the association between recurrent wheezing and atopy, the Asthma Predictive Index, exposure to risk factors, and total serum IgE levels as potential factors to predict recurrent wheezing.	Infants treated at a specialized outpatient clinic from November 2011 to March 2013.	N = 113<br><br>From 6 to 24 months of age.<br><br>- 65 infants with recurrent wheezing (63.0% male) with a mean age of 14.8 (SD = 5.2) months;<br><br>- 48 healthy infants (44.0% male) with a mean age of 15.2 (SD = 5.1) months	Multiple analysis model included sensitivity to inhalant and food antigens, positive Asthma Predictive Index (API), and other risk factors for recurrent wheezing (smoking during pregnancy, presence of indoor smoke, viral infections, and total serum IgE levels).<br>A logistic regression model was applied to the independent variables presenting p < 0.10 in the univariate analysis. The strength of the association between wheezing condition and the various outcomes was evaluated by odds ratio. Pearson's Chi-square test was used for the categorical variables. Fisher's exact test was used when the expected values were lower than five. Student's t-test was used to compare the mean age between both groups. A significance level of 5% was considered.	A. Risk factors for recurrent wheezing:<br>- antigen sensitivity (OR = 12.45; 95%CI 1.28–19.11);<br>- positive Asthma Predictive Index (OR = 5.57; 95%CI 2.23–7.96);<br>- exposure to environmental smoke (OR = 2.63; 95%CI 1.09–6.30).<br><br>B. Eosinophilia ≥ 4.0% e total IgE ≥ 100 UI/mL were more prevalent in the wheezing group but failed to remain in the model.<br><br>C. Smoking during pregnancy was identified in a small number of mothers, and secondhand smoke at home was higher in the control group.	Presence of atopy, positive Asthma Predictive Index and exposure to environmental smoke are associated to recurrent wheezing. Identifying these factors enables the adoption of preventive measures, especially for children susceptible to persistent wheezing and future asthma onset

The independent role of prenatal and postnatal exposure to active and passive smoking on the development of early wheeze in children<br><br>Vardavas CI, 2016<br><br>[6]	Meta-analysis and pooled analysis of 15 prospective mother-child cohorts	To examine the independent and combined role of different sources of exposure during different timeframes (prenatal maternal passive smoking, prenatal maternal active smoking and children's postnatal passive smoking exposure) on the development of wheeze symptoms up to the age of 2 years	Mother-child pairs form 15 European birth cohorts.<br><br>The 15 cohorts providing primary data included in this analysis participated in the European project Environmental Health Risks in European Birth Cohorts (ENRIECO)	27.993 mother-child pairs.<br><br>Age of children: from birth to 2 years of age	Using a multilevel mixed-effects logistic regression to evaluate the effect of exposure to tobacco smoke on the development of child wheeze during the first 2 years of life, calculating odds ratios and 95% confidence intervals.	A. Children with maternal exposure to passive smoking during pregnancy and no other smoking exposure were more likely to develop wheeze up to the age of 2years □ OR 1.11, 95% CI 1.03–1.20) compared with unexposed children.<br><br>B. Risk of wheeze was further increased by children's postnatal passive smoke exposure in addition to their mothers' passive exposure during pregnancy - □ OR 1.29, 95% CI 1.19–1.40.<br>B1. The risk was highest in children with both sources of passive exposure and mothers who smoked actively during pregnancy □OR 1.73, 95% CI 1.59–1.88).<br><br>C. Risk of wheeze associated with tobacco smoke exposure was higher in children with an allergic (OR 1.49, 95% CI 1.35–1.66)<br>*versus* nonallergic family history (OR 1.15, 95% CI 1.00–1.32; p-value for interaction 0.043)<br><br>C1. OR for exposure to maternal active smoking during pregnancy was increased in children with a parental history of allergy (OR 2.25, 95% CI 1.99–	Maternal passive smoking exposure during pregnancy is an independent risk factor for wheeze in children up to the age of 2 years.

						2.54) compared to those without (OR 1.28, 95% CI 1.09–1.50; p-value for interaction 0.011).	

Prevalence and risk factors for wheezing and allergic diseases in preschool children: A perspective from the Mediterranean coast of Turkey<br><br>Bolat E, 2017<br><br>[7]	Phase 1: prevalence study<br><br>Phase 2: case-control study	Determine the prevalence and risk Factors of respiratory And allergic diseases Using a modified ISAAC questionnaire In preschool children Attending daycare Centres in the city<br><br>Of Mersin.	All children Attending day-care Centres in the City centre Who were Randomly selected from The list of all Daycare centres in Mersin from January to December<br><br>2011.	396 preschool children<br>- 206 males<br>- 190 females<br><br>Mean age: 4.4± 0.9years.	**Phase 1.**<br>A modified ISAAC Questionnaire was used to assess the Symptoms of allergic And respiratory diseases, and the potential risk factors For the outcomes. The questionnaire Included questions About the symptoms And diagnosis of Respiratory diseases, eczema, food allergy And risk factors such As demographic characteristics, gestational factors, Family history, feeding practices, household Characteristics such As house crowding, Presence of pets, dampness, and tobacco Smoke exposure.<br><br>**Phase 2.**<br>Serum food and inhalant specific IgE, And skin tests were Performed in 45 children with frequent Wheezing and 28 Children with no<br><br>wheezing.	Significant risk factors for physician-diagnosed asthma:<br>- family history of atopy (OR=2.5, 95% CI: 1.3-4.7, p=0.004),<br>- dampness at home (OR=2.4, 95% CI: 1.2-4.8, p=0.008),<br>- a history of intestinal parasites (OR=4.3, 95% CI: 1.7-10.9, p=0.002),<br>- previous history of pneumonia (OR=6.9, 95% CI: 1.9-25.9, p=0.004),<br>- initiation of complementary foods before the age of three months (OR=6.1, 95%CI: 1.4-26.9, p=0.02)<br>- presence of food allergy (OR=3.1, 95% CI: 1.1-9.2, p=0.03)<br><br>The risk factors for frequent wheezing were:<br>- maternal smoking during pregnancy (OR=5.2, 95% CI: 0.9-28.7, p=0.05)<br><br>- high serum IgE levels (OR=2.9, 95% CI: 0.9-9.0, p=0.05) at borderline significance.	A high prevalence of asthma and allergic diseases, probably related to humid climatic properties in addition to other environmental and genetic factors was demonstrated.

Prevalence estimates and risk factors for early childhood wheeze across Europe: the EuroPrevall birth cohort<br><br>Selby A, 2018<br><br>[8]	Birth cohort study	To assess the prevalence of early childhood wheeze across Europe and evaluate risk factors focusing on food allergy, breast feeding and smoke exposure.	Infants from nine countries were recruited into the EuroPrevall birth cohort: Reykjavik (Iceland), Southampton (UK), Amsterdam (The Netherlands), Berlin (Germany), Lodz (Poland), Vilnius (Lithuania), Madrid (Spain), Milan (Italy) and Athens (Greece).	8805 infants with 24-months follow up data	Evaluation began at birth with follow-up of participants at 12 and 24 months using standardised questionnaires based on those used in previous epidemiological studies such as ISAAC.<br><br>At recruitment, data were collected on birth details, maternal diet, family history, maternal education (as a marker of socioeconomic status) and environmental exposures, including cigarette smoke and pet ownership. The 12-month and 24-month questionnaires included an extensive list of foods found in children's diets.<br><br>Additional assessments, including skin prick testing, measurement of specific IgE with or without a DBPCFC were performed according to a standardised protocol whenever parents reported symptoms suggestive of food allergy in their	The prevalence of wheeze in the second year of life ranged:<br>- from <2% in Lodz (Poland) and Vilnius (Lithuania)<br>- to 13.1% (95% CI 10.7% to 15.5%) in Southampton (UK) and 17.2% (95% CI 15.0% 19.5%) in Reykjavik (Iceland).<br><br>In multivariable analysis, Factors associated with wheeze:<br>- frequent lower respiratory tract infections in the first and second years of life (incidence rate ratio (IRR) 1.9 (95% CI 1.3 to 2.6) and 2.5 (95% CI 1.9 to3.4), respectively),<br>- postnatal maternal smoking (IRR 1.6, 95% CI 1.1 to 2.4),<br>- day care attendance (IRR 1.6, 95% CI 1.1 to 2.5)<br>- male gender (IRR 1.3, 95% CI 1.0 to 1.7).<br><br>Food allergy and breast feeding were not independently associated with wheeze.	The prevalence of early childhood wheeze varied considerably across Europe. Lower respiratory tract infections, day care attendance, postnatal smoke exposure and male gender are important risk factors.

					children.		

References

1. Sadeghnejad A, Karmaus W, Arshad SH, Kurukulaaratchy R, Huebner M, Ewart S. IL13 gene polymorphisms modify the effect of exposure to tobacco smoke on persistent wheeze and asthma in childhood, a longitudinal study. Respir Res. 2008 Jan 10;9(1):2. doi: 10.1186/1465-9921-9-2. PMID: 18186920; PMCID: PMC2265286.
2. Sahiner UM, Buyuktiryaki B, Cavkaytar O, Arik Yılmaz E, Soyer O, Sackesen C, Tuncer A, Sekerel BE. Recurrent wheezing in the first three years of life: short-term prognosis and risk factors. J Asthma. 2013 May;50(4):370-5. doi: 10.3109/02770903.2013.770013. Epub 2013 Feb 27. PMID: 23363237.
3. Lodge CJ, Zaloumis S, Lowe AJ, Gurrin LC, Matheson MC, Axelrad C, Bennett CM, Hill DJ, Hosking CS, Svanes C, Abramson MJ, Allen KJ, Dharmage SC. Early-life risk factors for childhood wheeze phenotypes in a high-risk birth cohort. J Pediatr. 2014 Feb;164(2):289-94.e1-2. doi: 10.1016/j.jpeds.2013.09.056. Epub 2013 Nov 14. PMID: 24238860.
4. Indinnimeo L, Porta D, Forastiere F, De Vittori V, De Castro G, Zicari AM, Tancredi G, Melengu T, Duse M. Prevalence and risk factors for atopic disease in a population of preschool children in Rome: Challenges to early intervention. Int J Immunopathol Pharmacol. 2016 Jun;29(2):308-19. doi: 10.1177/0394632016635656. Epub 2016 Mar 2. PMID: 26936651; PMCID: PMC5806707.
5. de Sousa RB, Medeiros D, Sarinho E, Rizzo JÂ, Silva AR, Bianca AC. Risk factors for recurrent wheezing in infants: a case-control study. Rev Saude Publica. 2016;50:15. doi: 10.1590/S1518-8787.2016050005100. Epub 2016 May 3. PMID: 27143615; PMCID: PMC4904490.
6. Vardavas CI, Hohmann C, Patelarou E, Martinez D, Henderson AJ, Granell R, Sunyer J, Torrent M, Fantini MP, Gori D, Annesi-Maesano I, Slama R, Duijts L, de Jongste JC, Aurrekoetxea JJ, Basterrechea M, Morales E, Ballester F, Murcia M, Thijs C, Mommers M, Kuehni CE, Gaillard EA, Tischer C, Heinrich J, Pizzi C, Zugna D, Gehring U, Wijga A, Chatzi L, Vassilaki M, Bergström A, Eller E, Lau S, Keil T, Nieuwenhuijsen M, Kogevinas M. The independent role of prenatal and postnatal exposure to active and passive smoking on the development of early wheeze in children. Eur Respir J. 2016 Jul;48(1):115-24. doi: 10.1183/13993003.01016-2015. Epub 2016 Mar 10. PMID: 26965294.
7. Bolat E, Arikoglu T, Sungur MA, Batmaz SB, Kuyucu S. Prevalence and risk factors for wheezing and allergic diseases in preschool children: A perspective from the Mediterranean coast of Turkey. Allergol Immunopathol (Madr). 2017 Jul-Aug;45(4):362-368. doi: 10.1016/j.aller.2017.01.002. Epub 2017 Apr 11. PMID: 28410872.
8. Selby A, Munro A, Grimshaw KE, Cornelius V, Keil T, Grabenhenrich L, Clausen M, Dubakiene R, Fiocchi A, Kowalski ML, Papadopoulos NG, Reche M, Sigurdardottir ST, Sprikkelman AB, Xepapadaki P, Mills ENC, Beyer K, Roberts G. Prevalence estimates and risk factors for early childhood wheeze across Europe: the EuroPrevall birth cohort. Thorax. 2018 Nov;73(11):1049-1061. doi: 10.1136/thoraxjnl-2016-209429. Epub 2018 May 10. PMID: 29748253.

SECTION 3 - PROTECTIVE FACTORS FOR WHEEZE DEVELOPMENT

Question 1. Are probiotics protective for preschool wheezing development?

Search strategy:

(“child, preschool"[MeSH Terms] OR "toddler*"[All Fields]) AND ("respiratory sounds"[MeSH Terms] OR "wheez*"[All Fields] OR "Asthma"[MeSH Terms:noexp]) AND ("Probiotics"[MeSH Terms:noexp] OR "Probiotics"[All Fields] OR "gut commensals"[All Fields] OR "bifidobacteria"[All Fields] OR "gut microbiota"[All Fields] OR "virome"[All Fields] OR "microbiome"[All Fields] OR "synbiotics"[All Fields] OR "prebiotics"[All Fields]) AND "english"[Language] AND 2008:2021 [pdat]

Are probiotics protective for preschool wheezing development?

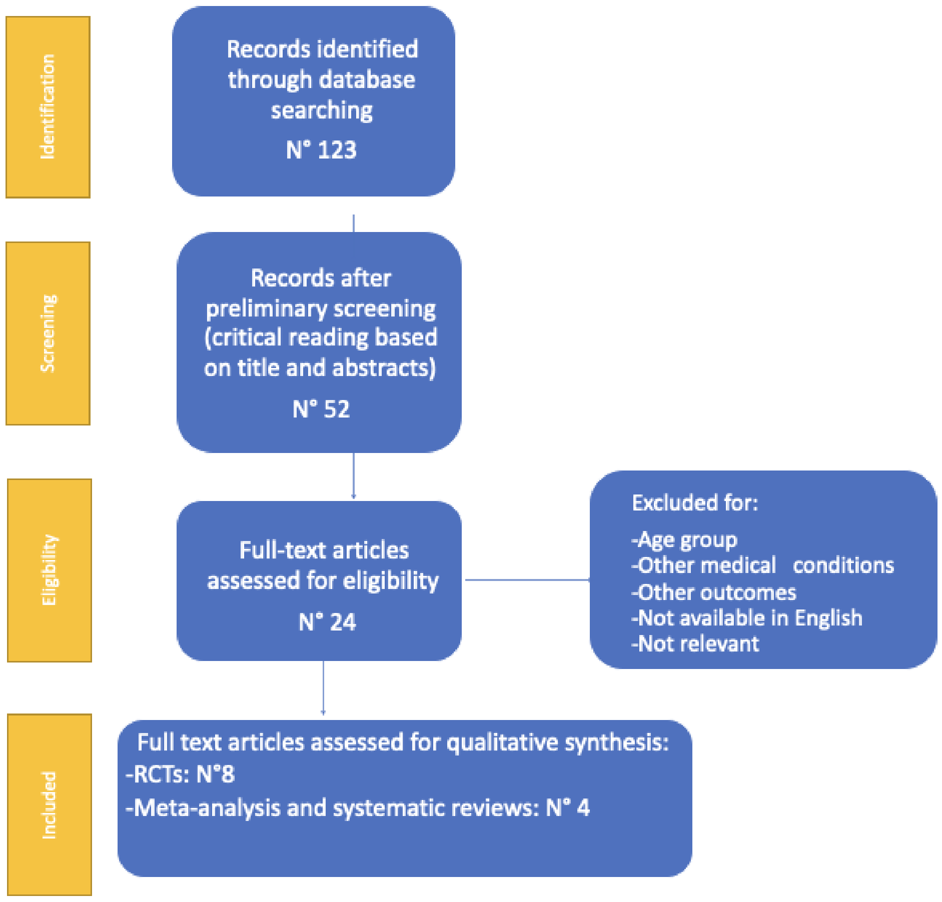

Modified from: Moher D, Liberati A, Tetzlaff J, Altman DG, The PRISMA Group (2009). Preferred Reporting Items for Systematic Reviews and Meta-Analyses: The PRISMA Statement. PLoS Med 6(7): e1000097

**Title of the study, first author, year [ref]**	**Type of study**	**Study design**	**N of patients (age)**	**Type of probiotic**	**Outcomes**	**Results**	**Relative risk (RR) or odds ratio (OR) + confidence interval (CI 95%)**
Early markers of allergic disease in a primary prevention study using probiotics: 2.5-year follow-up phase.<br><br>Prescott SL, 2008<br><br>[1]	Randomized double-blind controlled trial	Follow-up at 2.5 years after 6 months of daily administration of L. Acidophilus (n 77) or placebo (n 76) to infants at high risk for atopy	153 (2.5 years)	*Lactobacillus acidophilus*	-Atopic dermatitis<br><br>-Asthma (wheeze in the last year, wheeze ever, recurrent wheeze ≥ 2 episodes)<br>-IgE-mediated food allergy	No difference<br><br>No difference<br><br>No difference	/
Efficacy of probiotic *Lactobacillus* GG on allergic sensitization and asthma in infants at risk.<br><br>Rose MA, 2010<br><br>[2]	Randomized double-blind controlled trial	Administration of *L. rhamnosus* GG (n 65) or placebo (n 66) daily to infants at high risk for atopy* for 6 months<br><br>*with at least two wheezing episodes and a first-degree family history of atopic disease	131 (6-24 months)	*Lactobacillus rhamnosus* GG (LGG)	Before, at the end of intervention, and after 6 months of follow-up assessment of:<br><br>-atopic dermatitis<br><br>-asthma-related events (n. of wheezing episodes, days with wheeze, days on inhalative betamimetics, days on inhalative steroids, number of rescue-free days, number of symptom-free days, asthma symptom score)<br><br>-total and specific IgE, eosinophils, eosinophilic cationic protein, and TGF-	No difference<br><br>No difference<br>*In the subgroup sensitized to aeroallergenes higher asthma symptom score after probiotic supplementation (P = 0.04)*<br><br>Fewer sensitization	/

					beta	s towards aeroallergen s after 6 months of LGG (p = 0.027) and after 6 months of follow-up (p = 0.03)	
Follow-up of probiotic Lactobacillus GG effects on allergic sensitization and asthma in infants at risk.<br><br>Rose MA, 2011<br><br>[3]	Randomize d double-blind controlled trial	Follow-up for 26 months after 6 months of daily administration of LGG (n 39) or placebo (n 17) to infants at high risk for atopy	56 (42-44 months)	*Lactobacillus rhamnosus* GG	-Asthma-related events (asthma exacerbations numbers, use of beta-mimetic, use of inhalative steroids)<br><br>-Rhinoconjuntivitis<br><br>-Atopic eczema<br><br>-Allergic sensitizazion	No difference<br><br>Difference. *Higher prevalence in placebo (23.5% vs 10.3%, p = 0.03)*<br><br>No difference<br><br>Difference *Higher prevalence in placebo (52% vs 17%, p = 0.09)*	/
Early probiotic supplementation for allergy prevention: long-term outcomes.<br><br>Jensen MP, 2012	Randomize d double-blind controlled trial	Administration of L. *acidophilus* (n 66) or maltodextrin placebo (n 57) daily to infants at	123 (5 years)	*Lactobacillus acidophilus* (LAFTI L10/LAVRI-A1)	At 5 years of age diagnosis of:<br>-Eczema<br>-IgE-mediated food allergy<br>-Allergic rhinitis	 <br>No difference<br>No difference<br>No difference	 <br>1.95 (0.76-5.03)<br>1.83 (0.43-7.78)<br>0.506 (0.16-1.61)<br>1.40 (0.39-5.00)

[4]		high risk for atopy from birth to 6 months of life and follow-up at 5 years of age			-Asthma<br><br>-Sensitization to common food and aeroallergens	No difference<br>No difference<br>*Subite trend in the probiotic recipients of developing more recurrent wheezing (P:0.06)*<br><br>No difference	1.27 (0.55-2.93)
Treatment and secondary prevention effects of the probiotics Lactobacillus paracasei or Bifidobacterium lactis on early infant eczema: randomized controlled trial with follow-up until age 3 years.<br><br>Gore C, 2012<br><br>[5]	Randomized double-blind controlled trial	Administration of L. *Paracaseii* (n 45) or B. *Lactis* (n 45) or maltodextrin placebo (n 47) daily to infants with eczema for 3 months	137 (3-6 months)	*Lactobacillus paracaseii* CNCM I-2116 or *Bifidobacterium lactis* CNCM I-3446	Primary outcome:<br>Eczema 3 months post-intervention (primary outcome)<br><br>Then until 36 months of life:<br>-eczema<br>-gastrointestinal permeability<br>-urinary eosinophilic protein X<br>-allergen-sensitization<br>-allergic symptoms (included wheeze)	No difference<br><br>No difference<br>No difference<br>No difference<br>No difference<br>No difference	/
Probiotic supplementation during pregnancy or infancy for the prevention of asthma and wheeze: Systematic review and meta-analysis.	Systematic review and meta-analysis	Two reviewers independently identified randomised controlled trials evaluating probiotics administered to mothers during pregnancy or to infants during the	4866 (<8 years)<br><br>*Only 1 RCT meets our inclusion criteria (early life vitamin D supplementation and*	*Lactobacillus* spp and *Bifidobacterium* spp	-Doctor diagnosed asthma (primary outcome)<br><br>-Wheeze<br><br>-Lower respiratory tract infections	No difference<br><br>No difference<br><br>No difference	RR for asthma: 0.99 (0.81–1.21)<br><br>RR for wheeze: 0.97 (0.87–1.09)<br><br>RR for lower respiratory tract infection: 1.26 (0.99–1.61)

Azad MB, 2013<br><br>[6]		first year of life.	*wheezing offsprings in preschool children) [5]*				
Probiotic administration in early life, atopy, and asthma: a meta-analysis of clinical trials.<br><br>Elazab N,2013<br><br>[7]	Meta-analysis	Meta-analysis of double-blinded randomized placebo-controlled trials, evaluating the use of probiotics during pregnancy or early infancy for prevention of allergic diseases.	4031, (<18 yr)<br><br>*Only 6 RCTs meet our inclusion criteria (early life vitamin D supplementat ion and wheezing offsprings in preschool children), of these only 4 have been published after 2008 [1,3,4,5]*	*Lactobacillus* spp and *Bifidobacterium* spp	-Atopic sensitization<br><br>-Total IgE<br><br>-Asthma and wheeze development	Difference. *Lower risk of atopic sensitization after probiotics given prenatally (p = 0.035) or postnatally (p = 0.027)*<br><br>Difference. *Lower IgE level in the probiotic group than in the placebo group Mean reduction: –7.59 U/mL (–14.96 to –0.22); p = .044*<br><br>No difference	RR for atopic sensitization: 0.86 (0.75-0.98)<br><br>/<br><br>RR for asthma and wheeze: 0.96 (0.75–0.98)
Supplementation with Probiotics in the First 6 Months of Life Did Not Protect against Eczema and Allergy in At-Risk Asian Infants: A 5-Year	Randomized clinical trial	Follow-up at 5 years after 6 months of daily administration of of L. *Rhamnosus* + B. Long BL99 + cow milk (n 124)	220 (5 years)	L. *rhamnosus* LPR + B. *longum* BL999	-Asthma<br><br>-Allergic rhinitis	No difference<br><br>No difference	aRR for asthma at 5 years: 0.4 (95% CI): 0.2–0.7<br><br>aRR for allergic rhinitis at 5 years:0.4 (95% CI): 0.2-0.8<br><br>aRR for eczema: 0.8 (95%

Follow-Up.<br><br>Loo EXL, 2014<br><br>[8]		or cow milk alone (n 121) to infants at high risk for atopy from the first day of life to the 6 months of age			-Eczema<br><br>-Food allergy<br>-Sensitization to inhalant allergens	No difference<br><br>No difference<br>No difference	CI): 0.3–1.9
Early Probiotic Supplementation for Eczema and Asthma Prevention- A Randomized Controlled Trial.<br><br>Cabana MD, 2017<br><br>[9]	Randomized double-blind controlled trial	Administration of L. rhamnosus GG (n 92) or inulin placebo (n 92) daily to infants at high risk for atopy from birth to 6 months and then followed until 5 years	184 (<6 months)	Lactobacillus rhamnosus GG	-Eczema at 2 years (primary outcome)<br><br>-Asthma at 5 years	No difference<br><br>No difference	Hazard radio (HR) for eczema at 2 years: 0.95 (0.59-1.53)<br><br>HR for asthma at 5 years: 0,88 (0,41- 1,87)
Extensively hydrolysed casein formula containing L. rhamnosus GG reduces the occurrence of other allergic manifestations in children with cow's milk allergy: 3-year randomized controlled trial.<br><br>Berni Canani R, 2017<br><br>[10]	Parallel-arm randomized controlled trial	Administration of EHCF (extensively hydrolysed casein formula) +LGG (n 110) or EHCF alone (n 110) daily to infants with cow milk allergy (CMA) and followed for 36 months	220 (3-8 months)	*Lactobacillus rhamnosus* GG	Primary outcome:<br><br>Allergic manifestations (eczema, urticaria, asthma and rhinoconjunctivitis)over 36 months follow-up<br><br>-Tollerance acquisition (i.e. negativization of a double-blind food challenge) at 12, 24, 36 months	Significative difference (p <0.001) *lower allergic manifestations in the intervention group*<br><br>Significative difference at 36 months (P<0.001) *higher development of oral tolerance in the*	ARD (absolute risk difference) for allergic manifestations :- 0.23 (95%CI): -0.36- -0.10<br><br>ARD for tollerante acquistino at 36 months: 0.27 (95% CI): 0.11-0.43

						intervention group	
Probiotics supplementation in children with asthma: a systematic review and meta-analysis<br><br>Lin J, 2018<br><br>[11]	Systematic review and meta-analysis	Meta-analysis of randomized placebo-controlled trials, evaluating the use of probiotics during early infancy for prevention of asthma	910 (<16 years)<br><br>*Only 2 RCTs meet our inclusion criteria (early life vitamin D supplementation and wheezing offsprings in preschool children), of these only 1 has been published after 2008 [2]*	*Lactobacillus* spp, *Bifidobacterium* spp	Asthma development<br><br>Childhood asthma control test<br><br>Asthmatic symptom in the day and night<br><br>Number of symptom-free days<br><br>Pulmonary function (FEV1 and PEF)<br><br>Changes in the laboratory data (reduction of IL-4/increasing INF-γ / changes in IgE levels)	Difference *fewer episodes of asthma in the intervention group (p=0.01)*<br><br>No difference<br><br>No difference<br><br>No difference<br><br>No difference<br><br>No difference	RR for asthma episodes: 1.3 (1.06–1.59)
Association between probiotic supplementation and asthma incidence in infants: A meta-analysis of randomized controlled trials<br><br>Wei X, 2020<br><br>[12]	Systematic review and meta-analysis	Meta-analysis of randomized placebo-controlled trials, evaluating the use of probiotics during pregnancy or early infancy for prevention of asthma	6542 (<8 years<br><br>*Only 6 RCTs meet our inclusion criteria (early life vitamin D supplementation and wheezing offsprings in preschool*	*Lactobacillus* spp and *Bifidobacterium* spp	-Asthma<br><br>-Wheeze	No difference<br><br>No difference *However in the subgroup of atopic infants lower wheezing*	RR for asthma: 0.94 (0.82–1.09)<br><br>RR for wheeze: 0.97 (0.88–1.06)<br><br>RR for wheeze in infants with atopy disease: 0.61 (0.42-0.90)

			children) [1,4,5,8-10]			*events after probiotics (p <0.05)*	

LGG: Lactobacillus rhamnosus GG, EHCF:extensively hydrolysed casein formula, FEV1: Forced expiratory volume in the first second, PEF: Peak expiratory flow, IL-4: Interleukin 4, IFN-γ: interferon-gamma, IgE: immunoglobulin E, RCT: randomized placebo-control trial

References – Section 3, Question 1

1. Prescott SL, Wiltschut J, Taylor A, Westcott L, Jung W, Currie H, Dunstan JA. Early markers of allergic disease in a primary prevention study using probiotics: 2.5-year follow-up phase. Allergy. 2008 Nov;63(11):1481-90. doi: 10.1111/j.1398-9995.2008.01778.x. PMID: 18925885.
2. Rose MA, Stieglitz F, Köksal A, Schubert R, Schulze J, Zielen S. Efficacy of probiotic Lactobacillus GG on allergic sensitization and asthma in infants at risk. Clin Exp Allergy. 2010 Sep;40(9):1398-405. doi: 10.1111/j.1365-2222.2010.03560.x. Epub 2010 Jun 28. PMID: 20604800.
3. Rose MA, Schubert R, Schulze J, Zielen S. Follow-up of probiotic Lactobacillus GG effects on allergic sensitization and asthma in infants at risk. Clin Exp Allergy. 2011 Dec;41(12):1819-21. doi: 10.1111/j.1365-2222.2011.03876.x. PMID: 22107145.
4. Jensen MP, Meldrum S, Taylor AL, Dunstan JA, Prescott SL. Early probiotic supplementation for allergy prevention: long-term outcomes. J Allergy Clin Immunol. 2012 Nov;130(5):1209-1211.e5. doi: 10.1016/j.jaci.2012.07.018. Epub 2012 Sep 6. PMID: 22958946.
5. Gore C, Custovic A, Tannock GW, Munro K, Kerry G, Johnson K, Peterson C, Morris J, Chaloner C, Murray CS, Woodcock A. Treatment and secondary prevention effects of the probiotics Lactobacillus paracasei or Bifidobacterium lactis on early infant eczema: randomized controlled trial with follow-up until age 3 years. Clin Exp Allergy. 2012 Jan;42(1):112-22. doi: 10.1111/j.1365-2222.2011.03885.x. Epub 2011 Oct 18. PMID: 22092692.
6. Azad MB, Coneys JG, Kozyrskyj AL, Field CJ, Ramsey CD, Becker AB, Friesen C, Abou-Setta AM, Zarychanski R. Probiotic supplementation during pregnancy or infancy for the prevention of asthma and wheeze: systematic review and meta-analysis. BMJ. 2013 Dec 4;347:f6471. doi: 10.1136/bmj.f6471. PMID: 24304677; PMCID: PMC3898421.
7. Elazab N, Mendy A, Gasana J, Vieira ER, Quizon A, Forno E. Probiotic administration in early life, atopy, and asthma: a meta-analysis of clinical trials. Pediatrics. 2013 Sep;132(3):e666-76. doi: 10.1542/peds.2013-0246. Epub 2013 Aug 19. PMID: 23958764.
8. Loo EX, Llanora GV, Lu Q, Aw MM, Lee BW, Shek LP. Supplementation with probiotics in the first 6 months of life did not protect against eczema and allergy in at-risk Asian infants: a 5-year follow-up. Int Arch Allergy Immunol. 2014;163(1):25-8. doi: 10.1159/000356338. Epub 2013 Nov 16. PMID: 24247661.
9. Cabana MD, McKean M, Caughey AB, Fong L, Lynch S, Wong A, Leong R, Boushey HA, Hilton JF. Early Probiotic Supplementation for Eczema and Asthma Prevention: A Randomized Controlled Trial. Pediatrics. 2017 Sep;140(3):e20163000. doi: 10.1542/peds.2016-3000. Epub 2017 Aug 7. PMID: 28784701; PMCID: PMC5574725.
10. Berni Canani R, Di Costanzo M, Bedogni G, Amoroso A, Cosenza L, Di Scala C, Granata V, Nocerino R. Extensively hydrolyzed casein formula containing Lactobacillus rhamnosus GG reduces the occurrence of other allergic manifestations in children with cow's milk allergy: 3-year randomized controlled trial. J Allergy Clin Immunol. 2017 Jun;139(6):1906-1913.e4. doi: 10.1016/j.jaci.2016.10.050. Epub 2016 Dec 30. PMID: 28043872.
11. Lin J, Zhang Y, He C, Dai J. Probiotics supplementation in children with asthma: A systematic review and meta-analysis. J Paediatr Child Health. 2018 Sep;54(9):953-961. doi: 10.1111/jpc.14126. Epub 2018 Jul 27. PMID: 30051941.
12. Wei X, Jiang P, Liu J, Sun R, Zhu L. Association between probiotic supplementation and asthma incidence in infants: a meta-analysis of randomized controlled trials. J Asthma. 2020 Feb;57(2):167-178. doi: 10.1080/02770903.2018.1561893. Epub 2019 Jan 18. PMID: 30656984.

Question 2. Is vitamin D supplementation protective for preschool wheezing development?

Search strategy:

("child, preschool"[MeSH Terms] OR "toddler*"[All Fields]) AND ("respiratory sounds"[MeSH Terms] OR "wheez*"[All Fields] OR "Asthma"[MeSH Terms:noexp]) AND ("Vitamin D"[Mesh:NoExp] OR "vitamin D" OR “25-hydroxyvitamin D”) AND “english" [Language] AND 2008:2021[pdat]

Is vitamin D supplementation protective for preschool wheezing development?

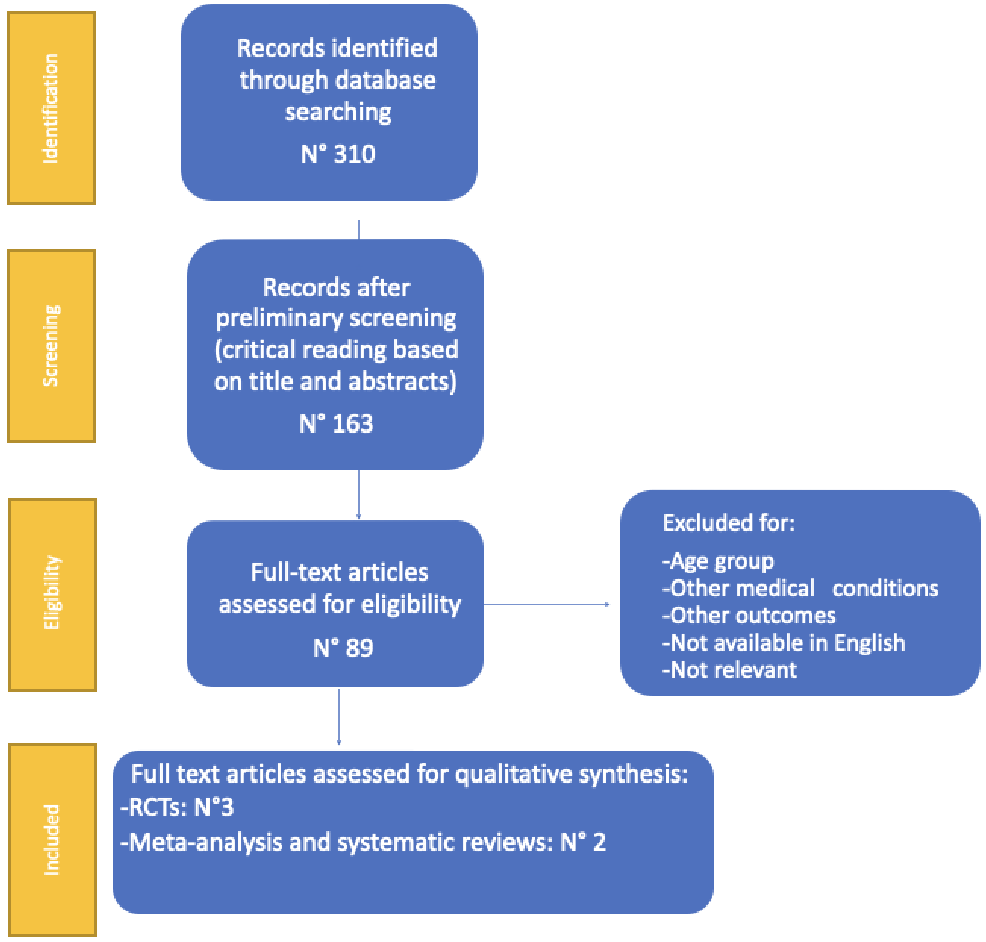

Modified from: Moher D, Liberati A, Tetzlaff J, Altman DG, The PRISMA Group (2009). Preferred Reporting Items for Systematic Reviews and Meta-Analyses: The PRISMA Statement. PLoS Med 6(7): e1000097

**Title of the study, first author, year [ref]**	**Type of study**	**Study design**	**N of patients (age)**	**Outcomes**	**Results**	**Relative risk (RR) or odds ratio (OR) + confidence interval (CI 95%)**
Vitamin D intervention in preschoolers with viral-induced asthma (DIVA).<br><br>Jensen ME, 2016<br><br>[1]	Randomized double-blind controlled trial	Administration of 100.000 IU vitamin D followed by 400 IU daily (n 11) or placebo followed by 400 IU vitamin D daily (n 11) for 6 months to preschool children with asthma	22<br>(1-5 years)	At 3 months after intervention assessment of:<br>-25(OH)D level<br><br>-children with 25(OH)D ≥75 nmol/L<br><br>-total 25(OH)D values over 6 months<br><br>-children with hypercalciuria at any time point<br><br>-rate of URTIs (upper respiratory tract infections)<br><br>-oral-corticosteroids use	No difference<br><br>Difference<br>*100% in the intervention group VS 54,5% in the control group, p=0.035*<br><br>Difference (p<0.001) *with a significant group, time, and group*time interaction effect*<br><br>No difference<br><br>No difference *70.8% in the intervention group vs 76% in the control group confirmed positive for at least one virus*<br><br>No difference. *0.82 in the intervention group vs 1.18 in the control group*<br><br>No difference *0.64 in the intervention group vs 0.73 in the control group*	RR: 0.88 (95 % CI): 0.32, 2.41<br><br>RR:0.68 (95 %CI: 0.30, 1.62)]

				-acute-care visits		
Vitamin D supplementation to prevent asthma exacerbations: a systematic review and meta-analysis of individual participant data.<br><br>Joliffe DA, 2017<br><br>[2]	Systematic review and meta-analysis	Meta-analysis of randomized placebo-controlled trials, evaluating the use of vitamin D supplementation or placebo to prevent atopic outcomes	955<br>(Adults and children)<br><br>*Only 1 RCT [1] meets our inclusion criteria (early life vitamin D supplementation and wheezing offsprings in preschool children)*	-asthma exacerbation requiring systemic corticosteroids<br><br>-proportion of participants with at least one exacerbation<br>-time to first exacerbation	Difference<br>*Lower rate in the intervention group (p=0.03)*<br>*Subgroup analyses show that protective effects are seen in children with baseline 25(OH)D < 25 nmol/l (p=0.046) but not in children with higher baseline 25(OH)d levels (p=0.08)*<br><br>No difference<br><br>No difference	adjusted incidence rate ratio [aIRR] 0.74 (0.56–0.97)<br><br>aIRR: 0.33 (0.11-0.98)
Impact of two oral doses of 100,000 IU of vitamin D in preschoolers with viral-induced asthma.<br><br>Ducharme FM, 2019<br><br>[3]	Randomized triple-blind controlled trial	Administration of 100.000 IU vitamin D x 2 doses, 14 weeks apart ± daily ICS (n 23) or placebo ± daily ICS (n 24) for 7 months to preschool children with viral-induced asthma	47<br>(1-5 years)	-25(OH)D over time<br><br>-25(OH)D ≥ 75 nmol/L 10 days after the first and second bolus	Difference (p<0.001) *higher 25(OH)D levels over time in the intervention group. However at 3.5 months and 7 months there is not a significantly difference in serum 25(OH)D level between cases and controls.*<br><br>Difference<br>*100% in the intervention group vs 48% in the control group (p=0.0003) after the first bolus and*<br>*100% in the intervention group vs 35 % in the control group (p<0.0001) after the second bolus*	

				Over a 7 months follow-up assessment of:<br><br>-hypercalciuria episodes<br>-URTIs<br><br>-asthma exacerbations<br><br>-oral-corticosteroids use<br><br>-acute care visits	No difference<br><br>No difference<br><br>No difference<br><br>No difference	RR:1.24 (95% CI): 0.88, 1.75<br><br>RR:1.78 (95% CI): 1.10, 2.90)<br><br>RR:1.21 (95% CI):0.57, 2.57<br><br>RR:1.34 (95% CI):0.69, 2.63
In “High-Risk” Infants with Sufficient Vitamin D Status at Birth, Infant Vitamin D Supplementation Had No Effect on Allergy Outcomes: A Randomized Controlled Trial.<br><br>Rueter K, 2020<br><br>[4]	Randomized double-blind controlled trial	Administration of 400 IU daily (n 97) or placebo (n 98) for the first 6 months of life to infants at high risk for atopy with normal 25(OH)D at birth	165 (0-1 year)	-25(OH)D levels over time<br><br>At a 1-year follow-up assessment of:<br>-eczema<br>-food allergy<br>-wheeze<br>-allergen sensitization<br><br>At a 2.5 years follow-up assessment of:<br>-eczema<br>-food allergy	Difference *higher at 3 months (p<0.01) and 6 months (p=0.02) of age in the vitamin D group BUT these differences not persist beyond the intervention period*<br><br>No difference<br>No difference<br>No difference No difference<br><br>No difference<br>No difference<br>No difference<br>No difference<br>No difference<br>*there are no statistically significant differences in incidence for any of the medically diagnosed allergic disease*	RR:1.45 (0.90–2.32)<br>RR:1.21 (0.39–3.83)<br>RR: 1.66 (0.92–3.01)<br>RR: 0.60 (0.29-1.25)<br><br>RR: 1.19 (0.79–1.80)<br>RR: 0.48 (0.12-1.84)<br>RR: 1.32 (0.79-2.23)<br>RR: 1.37 (0.68-2.76)<br>RR: 1.18 (0.63-2.20)

				-asthma/wheeze<br>-allergic rhinitis<br>-allergen sensitization	*outcomes or allergen sensitization rates between the vitamin D-supplemented and placebo groups at either 1 year or at 2.5 years of age.*	
Vitamin D supplementation in childhood asthma: a systematic review and meta-analysis of randomised controlled trials.<br><br>Kumar J, 2021<br><br>[5]	Systematic review and meta-analysis	Meta-analysis of randomized placebo-controlled trials, evaluating the use of vitamin D supplementation or placebo to prevent atopic outcomes	1579(1-18 years)<br><br>*Only 2 RCTs [1,3] included preschool children with asthma*	-asthma exacerbation requiring systemic corticosteroids<br><br>-proportion of children with asthma attacks of any severity<br><br>-emergency visits<br><br>-hospitalizations<br><br>-FEV1<br><br>-FeNo<br><br>-asthma control (GINA, ACT, C-ACT, ATAQ)<br>-number of children with adverse events	No difference (p=0.7)<br><br>No difference (p=0.007)<br><br>No difference (p=0.4)<br><br>No difference (p=0.8)<br><br>No difference<br>*mean difference -2.64 (-7.04 + 1.77)*<br><br>No difference<br>*mean difference -2.87 (-24.66 + 18.91)*<br><br>No difference<br><br>No difference (p=0.9)	RR:1.13 (95% CI): 0.86–1.48<br><br>RR: 0.84 (95% CI): 0.65–1.09<br><br>RR:0.97 (95% CI):0.89–1.07<br><br>RR:1.38 (95% CI): 0.52–3.66

URTIs: upper respiratory tract infections, FEV1:Forced expiratory volume in the first second, FeNO: exhaled nitric oxide fraction, GINA: Global Initiative for Asthma, C-ACT: Childhood Asthma Control Test, ACT: Asthma Control Test, ATAQ: Asthma Therapy Assessment Questionnaire

References – Section 3, Question 2

1. Jensen ME, Mailhot G, Alos N, Rousseau E, White JH, Khamessan A, Ducharme FM. Vitamin D intervention in preschoolers with viral-induced asthma (DIVA): a pilot randomised controlled trial. Trials. 2016 Jul 26;17(1):353. doi: 10.1186/s13063-016-1483-1. PMID: 27456232; PMCID: PMC4960871.
2. Jolliffe DA, Greenberg L, Hooper RL, Griffiths CJ, Camargo CA Jr, Kerley CP, Jensen ME, Mauger D, Stelmach I, Urashima M, Martineau AR. Vitamin D supplementation to prevent asthma exacerbations: a systematic review and meta-analysis of individual participant data. Lancet Respir Med. 2017 Nov;5(11):881-890. doi: 10.1016/S2213-2600(17)30306-5. Epub 2017 Oct 3. Erratum in: Lancet Respir Med. 2018 Jun;6(6):e27. PMID: 28986128; PMCID: PMC5693329.
3. Ducharme FM, Jensen M, Mailhot G, Alos N, White J, Rousseau E, Tse SM, Khamessan A, Vinet B. Impact of two oral doses of 100,000 IU of vitamin D_3 in preschoolers with viral-induced asthma: a pilot randomised controlled trial. Trials. 2019 Feb 18;20(1):138. doi: 10.1186/s13063-019-3184-z. PMID: 30777118; PMCID: PMC6379931.
4. Rueter K, Jones AP, Siafarikas A, Lim EM, Prescott SL, Palmer DJ. In "High-Risk" Infants with Sufficient Vitamin D Status at Birth, Infant Vitamin D Supplementation Had No Effect on Allergy Outcomes: A Randomized Controlled Trial. Nutrients. 2020 Jun 11;12(6):1747. doi: 10.3390/nu12061747. PMID: 32545250; PMCID: PMC7353265.
5. Kumar J, Kumar P, Goyal JP, Thakur C, Choudhary P, Meena J, Charan J, Singh K, Gupta A. Vitamin D supplementation in childhood asthma: a systematic review and meta-analysis of randomised controlled trials. ERJ Open Res. 2021 Feb 7;8(1):00662-2021. doi: 10.1183/23120541.00662-2021. PMID: 35141325; PMCID: PMC8819253

Question 3. Is breastfeeding protective for preschool wheezing development?

Search strategy:

((child, preschool[MeSH Terms]) OR (toddler*)) AND ((Respiratory Sounds[Mesh]) OR (wheez*) OR ("Asthma"[Mesh:NoExp])) AND ((“Breast Feeding") OR (“Lactation“) OR (“Polyunsaturated fatty acid”) OR ("Breast Feeding"[Mesh:NoExp]) OR (“Lactation”[Mesh:NoExp])) AND (english[Filter]) AND (2008:2021[pdat]

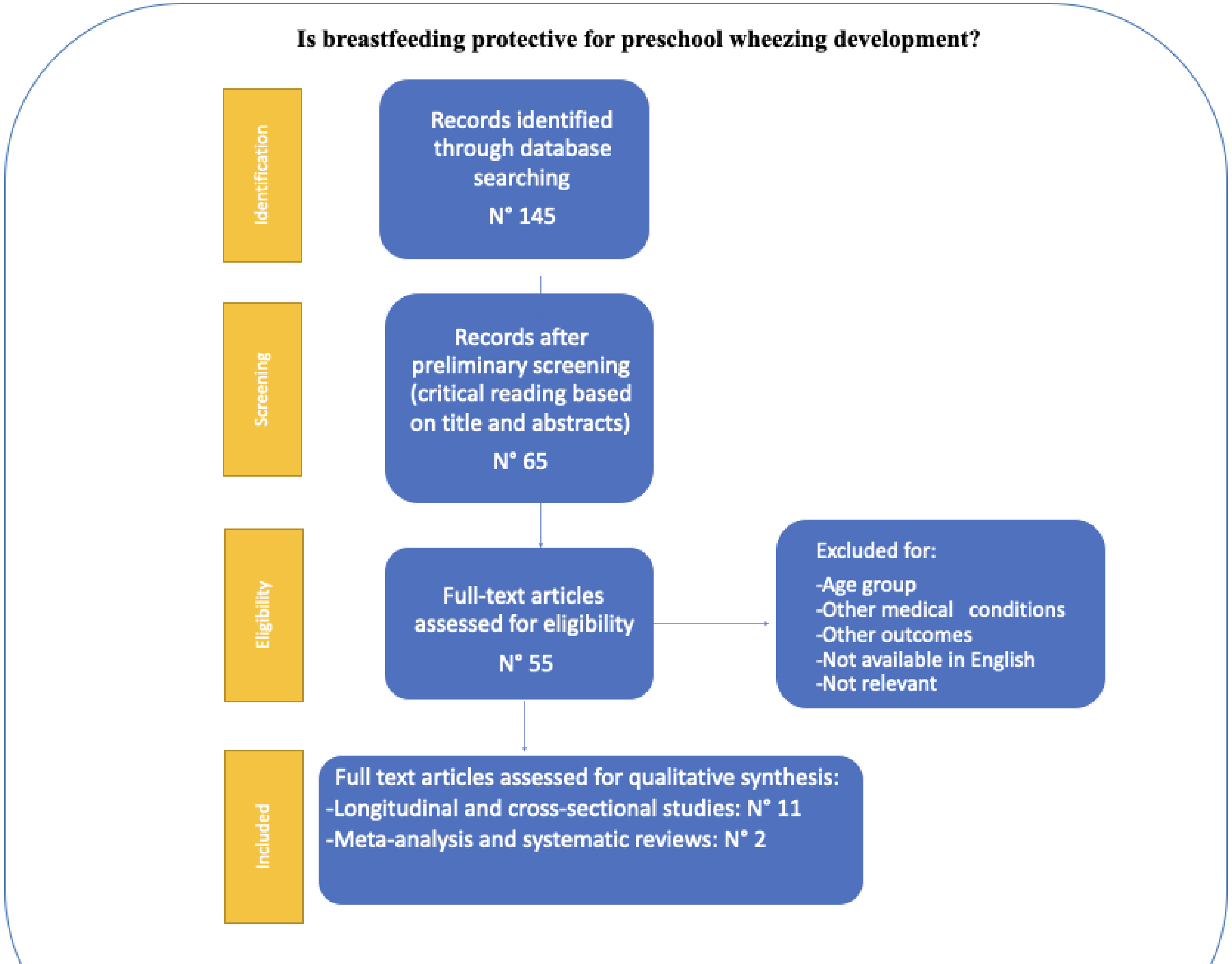

Modified from: Moher D, Liberati A, Tetzlaff J, Altman DG, The PRISMA Group (2009). Preferred Reporting Items for Systematic Reviews and Meta-Analyses: The PRISMA Statement. PLoS Med 6(7): e1000097

Title of the study, first author, year [ref]	Type of study	Study design	N of patients (age)	Outcomes	Duration and exclusivity of breastfeeding (BF)	Results	Relative risk (RR) or odds ratio (OR) + confidence interval (CI 95%)
Prospective study of breast-feeding in relation to wheeze, atopy, and bronchial hyperresponsiveness in the Avon Longitudinal Study of Parents and Children (ALSPAC).<br><br>Elliott L, 2009<br><br>[1]	Prospective birth cohort study	The study investigates the impact of breastfeeding modes on the incidence of atopy, wheezing and bronchial responsiveness to methacoline until 8 years	8131<br>(birth)	Wheezing in the first 3 yr<br><br>At ages 7-8 years risk of:<br>-Wheezing<br>-Atopy<br>-Bronchial responsiveness	BF >6 mo vs never BF	Difference<br>*Lower wheezing episodes in breastfeeding for at least 6 months children*<br><br>No difference<br>No difference<br>No difference	OR: 0.80 (95% CI): 0.70-0.90
Breast feeding, parental allergy and asthma in children followed for 8 years. The PIAMA birth cohort study.<br><br>Scholtens S, 2009<br><br>[2]	Prospective birth cohort study	The study investigates the impact of breastfeeding modes on the diagnosis of asthma (based on wheeze, dyspnoea and prescription of inhaled steroids) until 8 years of age	3115<br>[2238 in the subsample with nonallergic mothers, 877 in the subsample with allergic mothers]<br><br>(Birth)	-Asthma at 3 yr in the full sample<br><br>-Asthma at 3 years in subsample with nonallergic mothers<br><br>-Asthma at 3 years in subsample with allergic mothers<br><br>-Asthma at 4 years in the full sample, in subsample with allergic mother and in subsample with nonallergic mother<br><br>-Asthma at 5 yr in the full sample	BF> 16 we vs no BF	Difference ($p<0.05$)<br><br>Difference ($p<0.05$)<br><br>Difference ($p<0.05$)<br><br>No difference<br><br>Difference ($p<0.05$)	OR: 0.65 (95%CI):0.50-0.85<br><br>OR: ~0.70 (95% CI): ~0.50, ~1.00<br><br>OR: ~0.55 (95% CI): ~0.35, ~0.90<br><br>OR:0.50 (95%CI): 0.55-

				-Asthma at 5 years in subsample with nonallergic mothers<br><br>-Asthma at 5 years in subsample with allergic mothers<br><br>-Asthma at 6-7-8 years in the full sample or in subsample with non allergic mothers<br><br>-Asthma at 6-7-8 years in subsample with allergic mothers		No Difference<br><br>Difference (p<0.05)<br><br>Difference (p<0.05)<br><br>No Difference<br>*Breast feeding is associated with a lower asthma risk in children until 8 years of age without evidence of attenuation and regardless of the family history of allergy*	0.90<br><br>OR ~0.70 (95% CI: ~0.50, ~1.00)
Nutritional errors in the first months of life and their association with asthma and atopy in preschool children.<br><br>Strassburger SZ, 2010<br><br>[3]	Prospective birth cohort study*<br><br>*The cohort was sampled from an RCT; however, the data of interest for this SR are unrelated to randomization	The study investigates the impact of exclusive breastfeeding and introduction of cow's milk in the first year of life on the diagnosis of asthma, wheezing, and atopy at 3-4	347 (birth)	Asthma at 3-4 yr<br><br>Atopy<br><br>Asthma at 3-4 yr<br><br>Atopy	Cow's milk <4 mo<br><br>Exclusively BF< 6 mo VS exclusively BF ≥ 6 mo<br>mo	Difference (p<0.05)<br>*The early introduction of cow's milk is an important risk factor for triggering asthma/wheeze symptoms at the age of 4 years.*<br><br>No difference<br><br>No difference<br><br>Difference<br>*Exclusive breastfeeding*	OR 3.22 (95%CI): 1.05-9.80<br><br>OR 1.55 (95% CI): 0.61-

		years				*for longer than six months was potentially associated with protection against the development of atopy.*	3.92
Early Life Factors Associated with Incidence of Physician-diagnosed Asthma in Preschool Children: Results from the Canadian Early Childhood Development Cohort Study.<br><br>Midodzi WK, 2010<br><br>[4]	Prospective cohort study	The study investigates the associations between early life factors (included breastfeeding patterns) and the incidence of asthma at preschool age	8499<br>(<2 yr)	Asthma at 2-5 yr<br><br>Asthma at 2-5 yr	BF >3 mo vs never BF<br><br>BF 0-3 mo vs never BF	Difference<br>*BF more than 3 months is protective for the development of asthma*<br><br>No difference	HR: 0,82 (95% CI 0,69-0,97)<br><br>HR: 0,85 (95% CI 0,70-1,00)
Breastfeeding Protects against Current Asthma up to 6 Years of Age.<br><br>Silvers KM, 2012<br><br>[5]	Prospective birth cohort study	The study investigates the associations between breastfeeding patterns and wheezing and current asthma until 6 years of age	892<br>(birth)	Current asthma at 2 yr<br>Current asthma at 3 yr<br>Current asthma at 4 yr<br><br>Current asthma at 5 yr<br>Current asthma at 6 yr<br><br>Current asthma at 2 yr<br>Current asthma at 3 yr<br>Current asthma at 4 yr<br>Current asthma at 5 yr	Ever BF vs never BF<br><br>Exclusive BF vs partially BF	Difference<br><br>Difference<br><br>Difference<br>*Current asthma at 2, 3, and 4 years is reduced by each month of any breastfeeding (P <.005),the degree of protection beyond 3 years is more pronounced in atopic children.*<br><br>No difference<br><br>No difference	OR: 0,94 (95% CI: 0,90-0,97)<br>OR: 0,94 (95% CI: 0,91-0,97)<br>OR: 0,96 (95% CI: 0,92-0,99)<br><br>OR: 0,98 (95% CI: 0,94-1,00)<br>OR: 0,99 (95% CI: 0,96-1,03)

				Current asthma at 6 yr		Difference<br><br>Difference<br><br>Difference<br><br>Difference<br><br>Difference<br>*Each month of exclusive breastfeeding was associated with significant reductions in current asthma from 2 to 6 years (P < .03).*	OR: 0,85 (95% CI: 0,76-0,94)<br>OR: 0,88 (95% CI: 0,80-0,97)<br>OR: 0,92 (95% CI: 0,84-1,00)<br>OR: 0,88 (95% CI: 0,80-0,96)<br>OR: 0,92 (95% CI: 0,84-1,01)
Breastfeeding and wheeze prevalence in pre-schoolers and pre-adolescents: the Genesis and Healthy Growth studies.<br><br>Guibas GV, 2013<br><br>[6]	Meta-analysis of two different cross-sectional studies	Wheeze ever/in the last 12 months (current) and doctor-diagnosed asthma have been retrospectively reported by parents of the participants of two cross-sectional studies	1871<br>(1-5 yr)<br>And 1884 (9-13 yr)	-current/ever wheeze<br><br>-doctor-diagnosed asthma	Not exclusive BF-containing regimes vs exclusive BF-containing regimes	In preschool children:<br><br>Difference<br>*higher incidence of wheezing in regimes that do not entail exclusive BF*<br><br>No difference<br><br>In pre-adolescent: no association of infantile feeding patterns with the wheeze/asthma outcomes are observed.	OR(current wheeze): 2.25 (95% CI): 1.35-3.77<br><br>aOR(ever wheeze): 2.17 (95% CI): 1.33-3.55
Breastfeeding and Childhood Asthma: Systematic Review and Meta-Analysis.<br><br>Dogaru MC,	Systematic review and meta-analysis.	Meta-analysis of both cohort (longitudinal) and noncohort studies (cross-sectional or case- control),	50–168,283 children*<br><br>*** Values expressed as range**	-Asthma ever (analyzed by 75 studies)[1]	Ever BF vs. never BF<br><br>≥3 vs. <3 months<br><br>-≥6 vs. <6 months<br><br>Exclusive BF	 <br>Difference<br><br>Difference<br><br>Difference	0-2 years<br><br>OR: 0.65 (95% CI): 0.51-0.82<br><br>OR: 0.59 (95% CI): 0.50-0.70

2014<br><br>[7]		evaluating the relationship between BF and the development of pediatric asthma	(0 - >7 years)	-Recent asthma (analyzed by 46 studies)[2]	≥3 vs. <3 months<br>≥6 vs. <6 months<br><br>Ever BF vs. never BF<br>≥3 vs. <3 months<br>-≥6 vs. <6 months<br>Exclusive BF<br>≥3 vs. <3 months<br>≥6 vs. <6 months<br><br>Ever BF vs. never BF<br>≥3 vs. <3 months<br>-≥6 vs. <6 months<br>Exclusive BF<br>≥3 vs. <3 months<br>≥6 vs. <6 months<br><br>Ever BF vs. never BF<br>≥3 vs. <3 months<br>-≥6 vs. <6 months<br>Exclusive BF<br>≥3 vs. <3 months<br>≥6 vs. <6 months	Difference<br>Difference<br><br>Difference<br>Difference<br>Difference<br>Difference<br>Difference<br><br>Difference<br>Difference<br>Difference<br>Difference<br>Difference<br><br>Difference<br>Difference<br>Difference<br>Difference<br>Difference<br><br>Reduced risk with longer breastfeeding for all outcomes at 0–2 years, 3–6 years, and 7 or more years of age,	OR: 0.61 (95% CI): 0.50-0.74<br>OR: 0.62 (95% CI): 0.51-0.74<br>OR: 0.69 (95% CI): 0.58-0.81<br>3-6 years<br>OR: 0.79 (95% CI): 0.68-0.91<br>OR: 0.84 (95% CI): 0.76-0.92<br>OR: 0.57 (95% CI): 0.38-0.86<br>OR: 0.81 (95% CI): 0.59-1.11<br>OR: 0.51 (95% CI): 0.24-1.08<br><br>0-2 years<br>OR: 0.65 (95% CI): 0.51-0.82<br>OR: 0.59 (95% CI): 0.50-0.70<br>OR: 0.61 (95% CI): 0.50-0.74<br>OR: 0.62 (95% CI): 0.51-0.74<br>OR: 0.69 (95% CI): 0.58-0.81<br>3-6 years

							OR: 0.86 (95% CI): 0.65-1.13<br>OR: 0.79 (95% CI): 0.70-0.88<br>OR: 0.45 (95% CI): 0.30-0.69<br>OR: 0.83 (95% CI): 0.56-1.23<br>OR: 0.71 (95% CI): 0.53-0.94
Breastfeeding and childhood hospitalizations for asthma and other wheezing disorders.<br>Leung YJJ, 2016<br>[8]	Prospective cohort study	The study investigates the associations between breastfeeding duration and patterns and public hospital admissions for asthma, bronchitis, and bronchiolitis until 12 years of age	8301, (3mo)	From 2 to 6 years of age risk of:<br>-Pubblic hospital admission for asthma<br>-Bronchitis<br>-Bronchiolitis<br>-Pubblic hospital admission for asthma<br>-Bronchitis<br>-Bronchiolitis	Exclusive BF ≥ 3 months vs never BF<br>Partially BF for any length of time or exclusive BF < 3 months vs never BF	No difference<br>No difference<br>No difference<br>No difference<br>No difference<br>No difference	HR for asthma at 2-6 years of a age:<br>1.27 (95% CI: 0.82-1.98)<br>1,11 (95% CI: 0,64-1,90)
Breastfeeding and timing of first dietary introduction in relation to childhood	Cross-sectional study	The study investigates by a self-administrated questionnaire the	13335 (4-6 yr)	Asthma<br>Wheeze	Exclusively BF for 3-6 mo vs never BF	Difference<br>Difference<br>*children who are exclusively breastfed for*	OR: 0.81 (95% CI):0,72-0,91<br>OR: 0,93 (95% CI):0,87-0,99

asthma, allergies, and airway diseases: A cross-sectional study.<br><br>Huang C, 2016<br><br>[9]		associations between breastfeeding duration and patterns and timing of other dietary introduction and the prevalence of wheeze, asthma, hay fever, rhinitis, pneumonia and eczema		Hay fever, pneumonia, rhinitis and eczema<br><br>Hay fever, pneumonia, rhinitis and eczema<br><br>Asthma, wheeze, hay fever, pneumonia, rhinitis and eczema	Exclusively BF for more than 6 mo vs never BF<br><br>Longer duration BF	*three-six months have the lowest risk of asthma*<br><br>No differences<br><br>Differences (all p<0.05) *children who are exclusively breastfed for more than six months have the lowest risk of hay fever, pneumonia, rhinitis and eczema*<br><br>No differences if there is no family history of atopy	
Modes of Infant Feeding and the Risk of Childhood Asthma: A Prospective Birth Cohort Study.<br><br>Klopp A, 2017<br><br>[10]	Longitudinal development birth cohort study	The study investigates the associations between infant feeding mode at 3 months and the prevalence asthma at 3 years of age	3296 (birth)	At 3 months of age, the distribution of feeding modes was 27% direct breastfeeding, 32% breastfeeding with some expressed breast milk, 26% breast milk and formula, and 15% formula only. At 3 years of age, 12% of children were diagnosed with possible or probable asthma<br><br>Asthma at 3 years<br><br>Asthma at 3 years<br><br>Asthma at 3 years	-exclusively BF vs partially BF<br><br>-exclusively BF vs BF +	Difference<br><br>Difference	OR:1.64 (95% CI): 1.12-2.39<br><br>OR:1.73 (95% CI): 1.17-

					formula feeding<br><br>-formula feeding only	 <br>Difference<br>*Compared with direct breastfeeding, any other mode of infant feeding is associated with an increased risk of asthma*	2.57<br><br>OR:2.14 (95% CI): 1.37-3.35
Breastfeeding duration is inversely associated with asthma in Japanese children aged 3 years.<br><br>Watanabe JI,2018<br><br>[11]	Cross-sectional study	The study investigates the associations between breastfeeding duration and the prevalence of wheeze (defined according to the criteria of the international study of asthma and allergies in childhood) and asthma (considered present if the child had been diagnosed by a physician as having asthma)in Japanese children by a self-administrated questionnaire	6412<br>(3 yr)	-wheeze<br><br>-doctor-diagnosed asthma<br><br>-wheeze<br><br>-doctor-diagnosed asthma<br><br>-wheeze<br><br>-doctor-diagnosed asthma<br><br>-wheeze<br><br>-doctor-diagnosed asthma	Exclusive<br>BF≥ 4 mo<br><br>10 mo<BF< 14 mo vs BF< 10 mo<br><br>14mo<BF<19 mo vs BF <10 mo<br><br>BF ≥19 vs<br>BF <10 mo	No difference<br><br>No difference<br><br>No difference<br><br>Difference (p<0.05)<br><br>No difference<br><br>Difference (p<0.05)<br><br>No difference<br><br>Difference (p<0.05)<br><br>*>10 months of breastfeeding duration regardless of exclusivity are inversely related to doctor-diagnosed asthma*	OR: 0,69 (95%CI):0.52–0.91<br><br>OR: 0,73(95%CI):0.56–0.97<br><br>OR: 0,67(95%CI):0.51–0.88

Breastfeeding and childhood wheeze: Age-specific analyses and longitudinal wheezing phenotypes as complementary approaches to the analysis of cohort data.<br><br>Quingley MA, 2018<br><br>[12]	Longitudinal study	Using UK Millenium cohort data the authors examined the association between BF duration and wheezing in the previous yerar first for each age group separately (ages 9 months, 3 years, 5 years, 7 years, and 11 years) and then in terms of a longitudinal wheezing phenotype: "early transient" (wheezing any time up to age 5 years but not thereafter), "late onset" (any time from age 7 years but not beforehand), and "persistent" (any time up to age 5 years and any time from age 7 years)	10.126<br>(9 mo-11years)	Wheezing at 9 months<br><br>Wheezing at 3 years<br><br>Wheezing at 5 years<br><br><br><br><br><br><br><br><br>Wheezing at 7 years<br><br>Wheezing at 9 years<br><br><br>Early transient wheeze<br><br><br><br><br><br><br>Late-onset Wheeze<br><br>Persistent wheeze	BF for 6-9mo<br><br><br><br><br><br><br><br><br><br><br><br><br><br><br><br><br><br><br><br>Longer BF per month	Difference ($p<0.05$)<br><br><br>Difference ($p<0.05$)<br>Difference ($p<0.05$)<br><br>At 9 months, 3 and 5 years *there is a statistically significant association between breastfeeding duration and wheezing*<br><br><br>No Difference<br><br>No Difference<br><br>Difference ($p<0.05$)<br>*There is a strong dose-response relationship for breastfeeding per month and early transient wheeze*<br><br>No Difference<br><br><br>No Difference	OR: 0.73 (95% CI): 0.55-0.98<br><br>OR: 0.78 (95% CI): 0.65-0.94<br><br>OR: 0.79 (95% CI): 0.65-0.97<br><br><br><br><br><br><br><br><br><br><br><br><br><br><br><br>OR: 0.961 (95% CI): 0.942- 0.980

Breastfeeding and risk of childhood asthma: a systematic review and meta-analysis.<br><br>Xue M, 2021<br><br>[13]	Systematic review and meta-analysis.	Meta-analysis of retrospective/ prospective cohort studies, evaluating the relationship between BF and the development of pediatric asthma	Not available (<18 years)	-Asthma risk<br><br>-Asthma risk	Longer BF vs shorter BF<br><br>More esclusive BF vs less exclusive BF	Difference *Longer BF duration is associated with lower risk of asthma*<br><br>Difference *More esclusive BF is associated with lower risk of asthma*<br><br>Analysis of different age groups demonstrated a lower risk of asthma in the 0–2-years age group and the 3–6-years age group	OR 0.84, 95% CI 0.75–0.93<br><br>OR 0.81, 95% CI 0.72–0.91<br><br>**0-2 years** OR 0.73, 95% CI 0.63–0.83<br><br>**3-6 years** OR 0.69, 95% CI 0.55–0.87

BF: breastfeeding
1 refers to a condition that occurred at any time in the past, including asthma diagnosis retrieved from medical records and/or parent reports of doctor diagnosis, use of asthma medication, or wheeze accompanied by bronchial
2 those who met the "asthma ever" criteria within the last 12 months

References

1. Elliott L, Henderson J, Northstone K, Chiu GY, Dunson D, London SJ. Prospective study of breast-feeding in relation to wheeze, atopy, and bronchial hyperresponsiveness in the Avon Longitudinal Study of Parents and Children (ALSPAC). J Allergy Clin Immunol. 2008 Jul;122(1):49-54, 54.e1-3. doi: 10.1016/j.jaci.2008.04.001. Epub 2008 May 12. PMID: 18468669; PMCID: PMC2632970.
2. Scholtens S, Wijga AH, Brunekreef B, Kerkhof M, Hoekstra MO, Gerritsen J, Aalberse R, de Jongste JC, Smit HA. Breast feeding, parental allergy and asthma in children followed for 8 years. The PIAMA birth cohort study. Thorax. 2009 Jul;64(7):604-9. doi: 10.1136/thx.2007.094938. PMID: 19561280.
3. Strassburger SZ, Vitolo MR, Bortolini GA, Pitrez PM, Jones MH, Stein RT. Nutritional errors in the first months of life and their association with asthma and atopy in preschool children. J Pediatr (Rio J). 2010 Sep-Oct;86(5):391-9. English, Portuguese. doi: 10.2223/JPED.2031. PMID: 20938590.

4. Midodzi WK, Rowe BH, Majaesic CM, Saunders LD, Senthilselvan A. Early life factors associated with incidence of physician-diagnosed asthma in preschool children: results from the Canadian Early Childhood Development cohort study. J Asthma. 2010 Feb;47(1):7-13. doi: 10.3109/02770900903380996. PMID: 20100014.

5. Silvers KM, Frampton CM, Wickens K, Pattemore PK, Ingham T, Fishwick D, Crane J, Town GI, Epton MJ; New Zealand Asthma and Allergy Cohort Study Group. Breastfeeding protects against current asthma up to 6 years of age. J Pediatr. 2012 Jun;160(6):991-6.e1. doi: 10.1016/j.jpeds.2011.11.055. Epub 2012 Jan 30. PMID: 22289356.

6. Guibas GV, Xepapadaki P, Moschonis G, Douladiris N, Filippou A, Tsirigoti L, Manios Y, Papadopoulos NG. Breastfeeding and wheeze prevalence in pre-schoolers and pre-adolescents: the Genesis and Healthy Growth studies. Pediatr Allergy Immunol. 2013 Dec;24(8):772-81. doi: 10.1111/pai.12169. Epub 2013 Dec 2. PMID: 24298900.

7. Dogaru CM, Nyffenegger D, Pescatore AM, Spycher BD, Kuehni CE. Breastfeeding and childhood asthma: systematic review and meta-analysis. Am J Epidemiol. 2014 May 15;179(10):1153-67. doi: 10.1093/aje/kwu072. Epub 2014 Apr 11. PMID: 24727807.

8. Leung JY, Kwok MK, Leung GM, Schooling CM. Breastfeeding and childhood hospitalizations for asthma and other wheezing disorders. Ann Epidemiol. 2016 Jan;26(1):21-7.e1-3. doi: 10.1016/j.annepidem.2015.10.001. Epub 2015 Oct 23. PMID: 26559328.

9. Huang C, Liu W, Cai J, Weschler LB, Wang X, Hu Y, Zou Z, Shen L, Sundell J. Breastfeeding and timing of first dietary introduction in relation to childhood asthma, allergies, and airway diseases: A cross-sectional study. J Asthma. 2017 Jun;54(5):488-497. doi: 10.1080/02770903.2016.1231203. Epub 2016 Sep 7. PMID: 27603296.

10. Klopp A, Vehling L, Becker AB, Subbarao P, Mandhane PJ, Turvey SE, Lefebvre DL, Sears MR; CHILD Study Investigators, Azad MB. Modes of Infant Feeding and the Risk of Childhood Asthma: A Prospective Birth Cohort Study. J Pediatr. 2017 Nov;190:192-199.e2. doi: 10.1016/j.jpeds.2017.07.012. PMID: 29144244.

11. Watanabe JI, Tanaka K, Nagata C, Furukawa S, Arakawa M, Miyake Y. Breastfeeding duration is inversely associated with asthma in Japanese children aged 3 years. J Asthma. 2018 May;55(5):511-516. doi: 10.1080/02770903.2017.1349793. Epub 2017 Aug 16. PMID: 28759280.

12. Quigley MA, Carson C, Kelly Y. Breastfeeding and Childhood Wheeze: Age-Specific Analyses and Longitudinal Wheezing Phenotypes as Complementary Approaches to the Analysis of Cohort Data. Am J Epidemiol. 2018 Aug 1;187(8):1651-1661. doi: 10.1093/aje/kwy057. PMID: 29617923; PMCID: PMC6070068.

13. Xue M, Dehaas E, Chaudhary N, O'Byrne P, Satia I, Kurmi OP. Breastfeeding and risk of childhood asthma: a systematic review and meta-analysis. ERJ Open Res. 2021 Dec 13;7(4):00504-2021. doi: 10.1183/23120541.00504-2021. PMID: 34912884; PMCID: PMC8666625.
